# The Disorazole Z Family of Highly Potent Anticancer Natural Products from Sorangium cellulosum: Structure, Bioactivity, Biosynthesis, and Heterologous Expression

**DOI:** 10.1128/spectrum.00730-23

**Published:** 2023-06-15

**Authors:** Yunsheng Gao, Joy Birkelbach, Chengzhang Fu, Jennifer Herrmann, Herbert Irschik, Bernd Morgenstern, Kerstin Hirschfelder, Ruijuan Li, Youming Zhang, Rolf Jansen, Rolf Müller

**Affiliations:** a Department of Microbial Natural Products, Helmholtz-Institute for Pharmaceutical Research Saarland, Helmholtz Centre for Infection Research and Department of Pharmacy at Saarland University, Saarbrücken, Germany; b Department of Microbial Drugs, Helmholtz Centre for Infection Research, Braunschweig, Germany; c Helmholtz International Lab for Anti-Infectives, Shandong University-Helmholtz Institute of Biotechnology, State Key Laboratory of Microbial Technology, Shandong University, Qingdao, China; d Helmholtz International Lab for Anti-Infectives, Helmholtz Center for Infection Research, Braunschweig, Germany; e Department of Inorganic Chemistry, Saarland University, Saarbrücken, Germany; Shenzhen Bay Laboratory

**Keywords:** myxobacteria, secondary metabolites, disorazole Z, anticancer natural products, biosynthetic gene cluster, direct cloning, heterologous expression

## Abstract

Myxobacteria serve as a treasure trove of secondary metabolites. During our ongoing search for bioactive natural products, a novel subclass of disorazoles termed disorazole Z was discovered. Ten disorazole Z family members were purified from a large-scale fermentation of the myxobacterium Sorangium cellulosum So ce1875 and characterized by electrospray ionization–high-resolution mass spectrometry (ESI-HRMS), X-ray, nuclear magnetic resonance (NMR), and Mosher ester analysis. Disorazole Z compounds are characterized by the lack of one polyketide extension cycle, resulting in a shortened monomer in comparison to disorazole A, which finally forms a dimer in the bis-lactone core structure. In addition, an unprecedented modification of a geminal dimethyl group takes place to form a carboxylic acid methyl ester. The main component disorazole Z1 shows comparable activity in effectively killing cancer cells to disorazole A1 via binding to tubulin, which we show induces microtubule depolymerization, endoplasmic reticulum delocalization, and eventually apoptosis. The disorazole Z biosynthetic gene cluster (BGC) was identified and characterized from the alternative producer *S. cellulosum* So ce427 and compared to the known disorazole A BGC, followed by heterologous expression in the host Myxococcus xanthus DK1622. Pathway engineering by promoter substitution and gene deletion paves the way for detailed biosynthesis studies and efficient heterologous production of disorazole Z congeners.

**IMPORTANCE** Microbial secondary metabolites are a prolific reservoir for the discovery of bioactive compounds, which prove to be privileged scaffolds for the development of new drugs such as antibacterial and small-molecule anticancer drugs. Consequently, the continuous discovery of novel bioactive natural products is of great importance for pharmaceutical research. Myxobacteria, especially *Sorangium* spp., which are known for their large genomes with yet-underexploited biosynthetic potential, are proficient producers of such secondary metabolites. From the fermentation broth of Sorangium cellulosum strain So ce1875, we isolated and characterized a family of natural products named disorazole Z, which showed potent anticancer activity. Further, we report on the biosynthesis and heterologous production of disorazole Z. These results can be stepping stones toward pharmaceutical development of the disorazole family of anticancer natural products for (pre)clinical studies.

## INTRODUCTION

Myxobacteria are soil-dwelling Gram-negative delta proteobacteria and known as prolific sources of natural products often exhibiting novel chemical scaffolds and unique biological modes of action (1–4). Disorazoles are a family of macrocyclic dilactones with dimeric or pseudodimeric structures, and they represent one group of myxobacterial secondary metabolites showing promising cytotoxic activity (5, 6). Furthermore, this family of compounds was shown to block the invasion of human epithelial cells by group A streptococci via targeting of the host factor ezrin (7). Therefore, disorazoles have the potential to be developed as anticancer or anti-infective drugs.

Previously, 29 disorazole A family compounds (disorazole A1 and 28 variants) were isolated from the fermentation culture of the myxobacterium Sorangium cellulosum So ce12 (8). The main component, disorazole A1 (compound 1) showed extremely high cytotoxicity, with half-maximal inhibitory concentrations (IC_50_s) in the range of 2 to 42 pmol/L against a panel of cancer cell lines, including a multidrug-resistant KB line by inhibiting tubulin polymerization and inducing apoptosis (9). In 2005, the disorazole A biosynthetic gene cluster from *S. cellulosum* So ce12 (*dis12* gene cluster) was identified by transposon mutagenesis (10, 11). The core biosynthetic gene cluster encodes a multifunctional megasynthetase consisting of polyketide synthases (PKS), a nonribosomal peptide synthetase (NRPS), and a dedicated acyltransferase (AT) and thus belongs to the family of *trans*-AT PKS-NRPS hybrid gene clusters (12, 13). In 2016, the *dis12* gene cluster was subcloned and heterologously expressed in Myxococcus xanthus DK1622 leading to the production of disorazole A2 (compound 2) as the major compound (14).

In the course of our ongoing screening for biologically active natural products from myxobacteria, a novel subclass of disorazole, i.e., disorazole Z, was discovered to be produced by several *S. cellulosum* strains, such as So ce1875 and So ce427 (15). Structure elucidation and characterization of disorazole Z have not been published to date. Nevertheless, and due to its outstanding anticancer activity, disorazole Z was subjected to preclinical evaluation as a cytotoxic component in a drug-targeting approach for the treatment of luteinizing hormone-releasing hormone (LHRH) receptor-overexpressing cancers by conjugation with the receptor-targeting moiety d-Lys6-LHRH (16–19). Compared to doxorubicin-d-Lys6-LHRH, the disorazole Z conjugate demonstrated an increased cytotoxicity *in vitro* in HCC 1806 and MDA-MB-231 triple-negative breast cancer cells (20). In the meantime, disorazole Z was also identified as a maytansine site ligand, which was confirmed by solving the crystal structure of the tubulin-disorazole Z complex (21).

Due to the novelty of these compounds’ structures, their intriguing biological activities, and their promising potential for clinical application, massive efforts were made to develop chemical synthesis routes. Total synthesis has been achieved for some disorazole congeners and nonnatural analogs, for example, disorazole A1 (compound 1) and also a simplified disorazole Z (22, 23). Nevertheless, biotechnological methods still present significant advantages for obtaining these types of complex natural products (24). Furthermore, taking advantage of synthetic biotechnology, rational engineering of the biosynthetic pathway in an advantageous heterologous host could also facilitate high-efficiency production of certain target components or generate nonnatural compounds (25, 26).

In this work, large-scale fermentation afforded the isolation of 10 disorazole Z family members featuring two-carbon shorter monomers and unprecedented modification of a geminal dimethyl group compared to disorazole A. We report the first full structure elucidation of the disorazole Z congeners using nuclear magnetic resonance (NMR), Mosher ester, and X-ray analysis. Furthermore, we characterized the bioactivity of disorazole Z1 (compound 3). In addition, the disorazole Z biosynthetic pathway was identified by comparative analysis and heterologous expression. Promoter engineering and gene deletion experiments were carried out using the heterologous expression system, which allowed the function of a methyltransferase involved in disorazole Z biosynthesis to be assigned.

## RESULTS AND DISCUSSION

### Isolation and full structure elucidation of the disorazole Z family of compounds.

**FIG 1:**
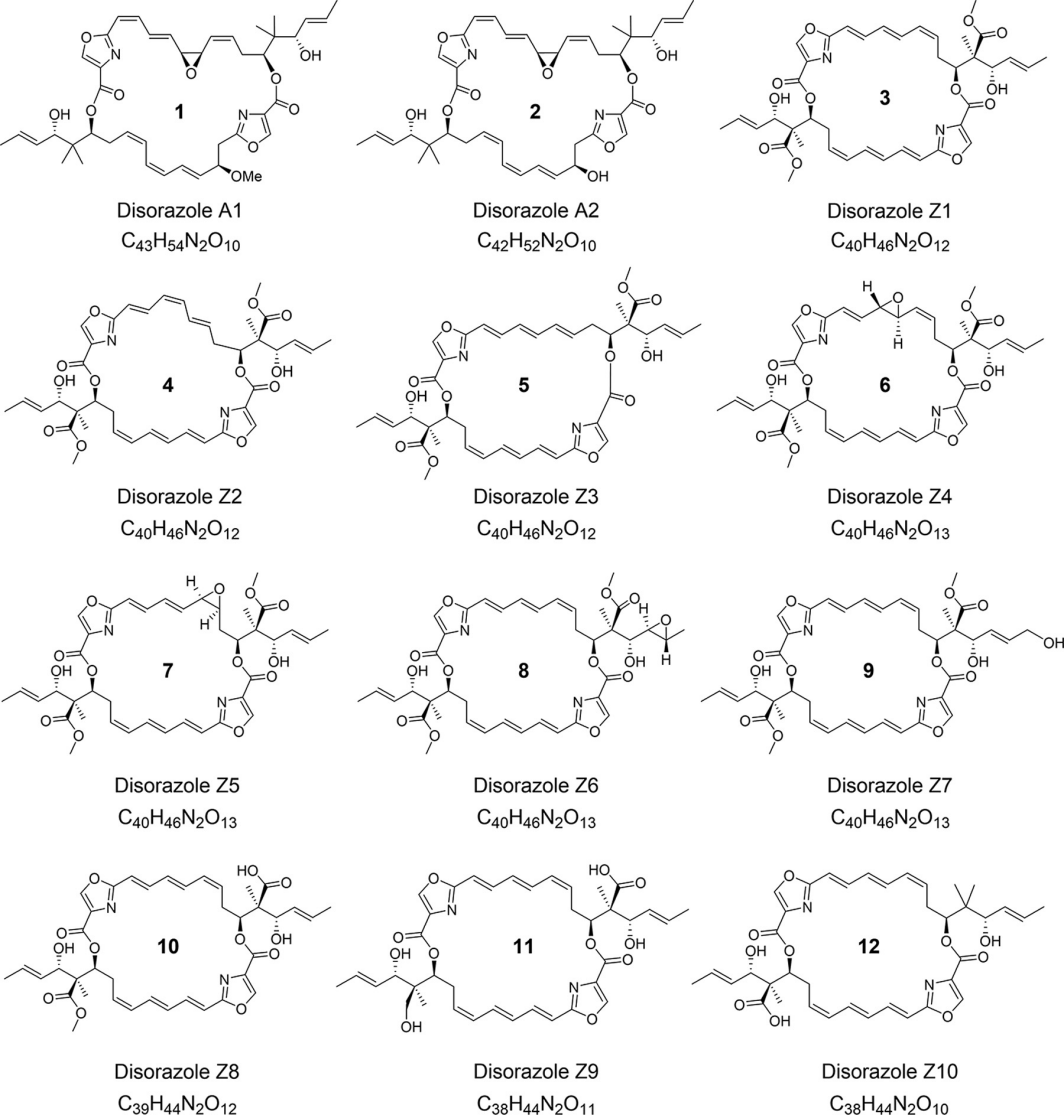
Chemical structures of disorazole Z family compounds and disorazole A for comparison.

After large-scale fermentation of *S. cellulosum* So ce1875, we were able to isolate 10 disorazole Z congeners (compounds 3 to 12), achieving a production titer of 60 to 80 mg/L disorazole Z1 (compound 3). An overview of the chemical structures of these compounds is given in Fig. 1. Details of fermentation and purification are provided in Materials and Methods.

**FIG 2:**
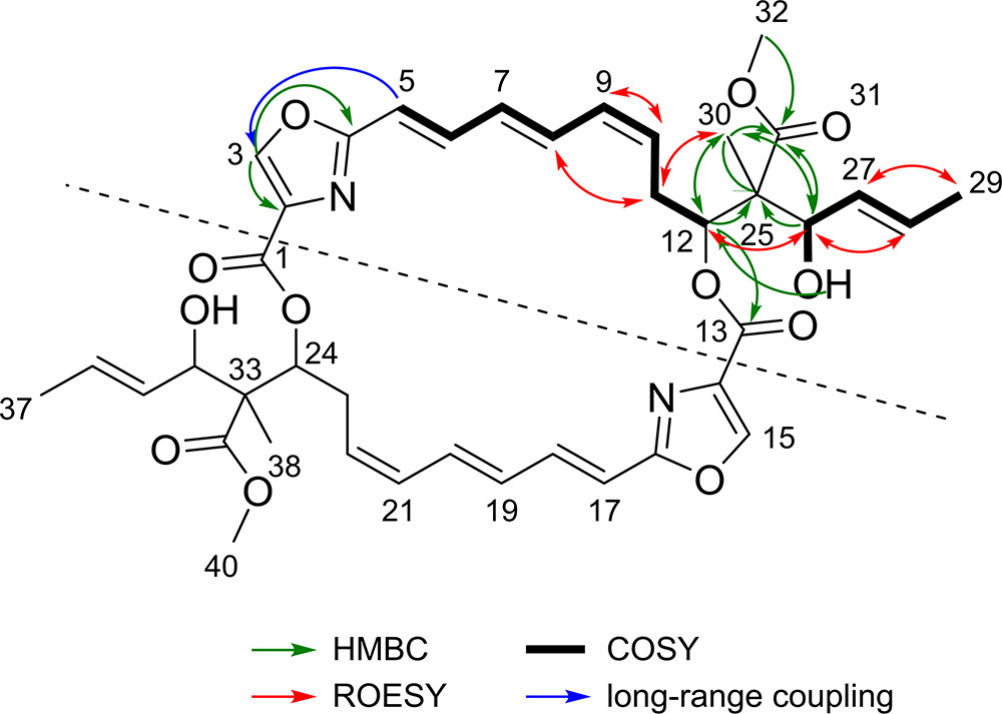
NMR correlations of structural parts A and B of disorazole Z1. ^1^H,^1^H-COSY correlations (thick bonds) and their interconnection by selected ^1^H,^13^C-HMBC correlations (green arrows), ^1^H,^1^H-rotating frame Overhauser effect spectroscopy (^1^H,^1^H-ROESY) correlations (red arrows) and ^1^H,^1^H-long-range coupling (blue arrow). ROESY, rotating-frame nuclear Overhauser effect spectroscopy.

Molecular ion clusters [M+H]^+^ (*m/z* 747.3132), [M+Na]^+^ and [M−H_2_O+H]^+^ in electrospray ionization–high-resolution mass spectrometry (ESI-HRMS) revealed the elemental formula C_40_H_46_N_2_O_12_ of disorazole Z1 (compound 3) from which 19 double-bond equivalents were calculated. Surprisingly, the ^13^C NMR spectrum showed only half the number of carbon signals solely explicable for a completely symmetrical dimer. The ^1^H NMR spectrum in acetone-*d*_6_ presented 23 protons, which were correlated with their corresponding carbons in a ^1^H,^13^C heteronuclear single quantum coherence (HSQC) NMR spectrum with the exception of one doublet of a secondary alcohol proton (OH-26) at 4.16 ppm. The ^1^H,^1^H correlation spectroscopy (COSY) NMR spectrum allowed us to assign two main structural parts, A and B (Fig. 2). H-5 of part A and a ^1^H singlet (3-H) at 8.53 ppm were connected by a long-range coupling. The corresponding carbon C-3 had no correlation in the heteronuclear multiple-bond correlation (HMBC) NMR spectrum. However, the direct ^1^*J*_C,H_ coupling of 213 Hz indicated its role in a hetero-aromatic ring (27). The further ring carbons C-2 and C-4 were identified by their HMBC correlations with H-3, while the exact connection of the oxazole ring to structural part A was shown by the HMBC correlation between C-4 and H-5. However, H-6 did not show any HMBC correlation, appearing as a flat and broad proton signal. The continuation of structural part A at the opposite oxymethine C-12 (δ_C_ 76.28) was indicated by an apparent low-field acylation shift (δ_H_ 5.45) of the H-12 proton signal that provided the only HMBC correlation of the carboxyl carbon C-13 (δ_C_ 159.65). By HMBC correlations with H-12, H-26, and the hydroxyl group OH-26, the quaternary sp^3^ carbon C-25 (δ_C_ 56.26) was revealed as the interconnection of parts A and B. Further, the HMBC correlations showed the direct attachment of the methyl group C-30 to C-25. The last open position was filled by a carboxymethyl ester at C-25, as indicated by three HMBC correlations of the carboxyl carbon C-31 (δ_C_ 173.81) with the methyl group C-30, the alcohol methine H-26, and the acylated oxymethine H-12. The stereochemistry of the *cis* and *trans* double bonds was derived from vicinal coupling constants of about 11 Hz and 15 Hz, respectively. With all NMR structural elements assigned, 18 double-bond equivalents were consumed for two identical structural parts. Using the last equivalent, both halves were connected to give the point symmetrical dilactone ring of compound 3, in which all identical structural elements have the same vicinity.

X-ray analysis revealed the relative configuration of disorazole Z1 (compound 3). In order to elucidate the absolute configuration, Mosher’s method based upon ^1^H and ^13^C NMR chemical shift differences was applied (28–31). Consequently, all asymmetric centers (12, 25, 26) of compound 3 are in the *S* configuration, as shown in Fig. 1. A full description of the stereo-chemical analyses is given in the supplemental material. The structural assignment is in full agreement with the structure of the tubulin-disorazole Z complex published recently (21).

Besides disorazole Z1 (compound 3), nine more variants have also been isolated and characterized. Two of them turned out to be isomers of compound 3 with different double bond geometries, i.e., disorazole Z2 (compound 4, Δ^7,8^-*cis*-disorazole Z) and disorazole Z3 (compound 5, Δ^9,10^-*trans*-disorazole Z). Four more polar disorazole Z variants were identified as isomers with the ESI-HRMS-derived elemental formula C_40_H_46_N_2_O_13_. They contained one additional oxygen, either incorporated as an epoxide group replacing different double bonds, i.e., disorazole Z4 (compound 6, 7,8-epoxy-disorazole Z), disorazole Z5 (compound 7, 9,10-epoxy-disorazole Z), and disorazole Z6 (compound 8, 27,28-epoxy-disorazole Z), or incorporated as primary unsaturated alcohol at the end of a side chain, i.e., disorazole Z7 (compound 9, 29-hydoxy-disorazole Z). Three disorazole Z variants with smaller molecular weight were proposed to be biosynthetic intermediates featuring different degrees of modification on geminal methyl groups, i.e., disorazole Z8 (compound 10, 31-*O*-desmethyl-disorazole Z), disorazole Z9 (compound 11, 31-*O*-desmethyl-39-hydroxy-disorazole Z) and disorazole Z10 (compound 12, 39-*O*-desmethyl-25,25-dimethyl-disorazole Z). Details of the structure elucidation of these analogues are given in the supplemental material.

### Discovery and comparative analysis of disorazole Z biosynthetic gene cluster.

**FIG 3:**
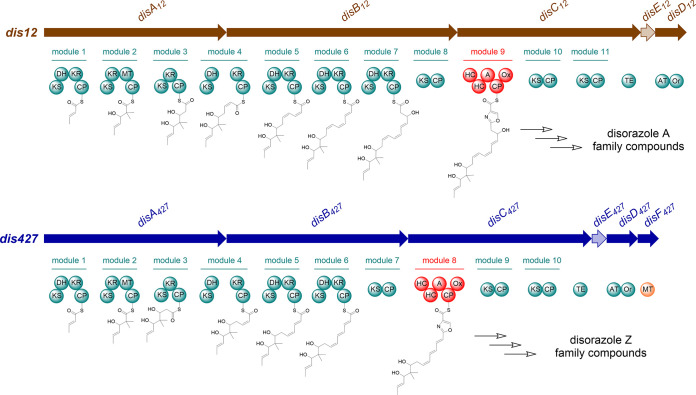
Comparison of proposed biosynthetic pathways between disorazole A and Z. *dis12*, the disorazole A biosynthetic gene cluster from *S. cellulosum* So ce12; *dis427*, the disorazole Z biosynthetic gene cluster from *S. cellulosum* So ce427; KS, ketosynthase; DH, dehydratase; KR, ketoreductase; CP, carrier protein; MT, methyltransferase; HC, heterocyclization domain; A, adenylation domain; Ox, oxidation domain; TE, thioesterase; AT, acyl transferase; Or, oxidoreductase. Oxidation of the methyl group and formation of the methyl ester may occur prior to or after release of the bis-lactone.

The significant structural differences between the disorazole Z and A family compounds motivated us to investigate the disorazole Z biosynthetic pathway. The draft genome sequence of the alternative producer strain *S. cellulosum* So ce427 was obtained by Illumina sequencing and subjected to antiSMASH analysis for annotation of biosynthetic gene clusters (32). A *trans*-AT PKS-NRPS gene cluster (*dis427* gene cluster; GenBank accession number OQ408282) which exhibited significant overall similarity to the known *dis12* gene cluster (GenBank accession number DQ013294 or AJ874112) was speculated to be responsible for the biosynthesis of disorazole Z (Fig. 3). Intriguingly, this gene cluster has two main distinguishing features: on the one hand, one PKS module is lacking in the second polyketide synthase gene, *disB_427_*, which corresponds nicely with the two-carbon shortened monomeric unit of disorazole Z compared to one half-side of the disorazole A congener; on the other hand, the methyltransferase gene *disF_427_* was found downstream of *disD_427_*, which is not present in the *dis12* gene cluster and could be assigned to the *O*-methylation function needed for the methylation of the carboxyl group found in disorazole Z. The carboxyl group most likely arises from the oxidation of one of the methyl groups at positions C-25 and C-33. However, no gene flanking the corresponding biosynthetic gene cluster (BGC) encoding an expected oxidative function could be assigned for this hypothetical step. The organization of the remaining part of the disorazole Z pathway is quite similar to that of the disorazole A pathway. The biosynthesis of the half-side of the disorazole Z dilactone begins with condensing one malonyl coenzyme A (malonyl-CoA) with the starter acetate. After five additional rounds of extension with malonyl-CoA, the ketosynthase (KS) domain in module 7 is not expected to extend the polyketide chain, because the catalytic histidine of KS7 is substituted with alanine (Fig. S7). The mutation of the conserved motif C-H-H to C-A-H most likely causes malfunction of this KS domain, as the histidine residue is known to play an essential role in decarboxylation and condensation reactions (33). However, this type of KS was proven to be a gatekeeper and still capable of transferring the polyketide chain between different domains in FR901464 biosynthesis (34). The adenylation domain of DisC_427_ activates serine, also found in the disorazole A pathway. Similar tandem heterocyclization (HC) domains were proven to be essential for vibriobactin and anguibactin biosynthesis (35, 36). It is assumed likely that the HC domains of DisC_427_ work in the same fashion. However, it is also possible that one of the HC domains condenses the serine with the polyketide chain, and the other HC domain might perform the cyclization of the serine moiety to form the oxazoline ring, which is finally oxidized to its oxazole form by the oxidation domain. The extension of the nascent intermediate bound to the carrier protein of module 9 is supposed to stop here, because the two following PKS modules are most likely nonextending ones, which are suggested by annotation to be noncondensing KS9 and KS10 domains similar to KS7 (see Fig. S7 in the supplemental material). Finally, the termination of the assembly line and the cyclization of the two monomeric subunits are likely to be similar to the mechanism described for enterobactin, elaiophylin, and conglobatin biosynthesis (37–39), eventually forming the bis-lactone structure. The carboxylic acid methyl ester in disorazole Z might be introduced before or after release from the assembly line by stereospecific oxidation of a methyl group, giving rise to the free carboxylic acid and subsequent methyl ester formation. Nevertheless, the oxidation process involved remains enigmatic and needs further study. Intriguingly, after expression of the *dis427* gene cluster, disorazole Z1 is also formed, being the major heterologous product in the host M. xanthus DK1622 (see below), which indicates that the missing functionalities are encoded within either the BGC or the heterologous host to harbor similar genomic functions capable of oxidizing the precursor. The final step of methyl ester formation could be connected to DisF, encoded in the corresponding BGC.

### Heterologous production and biosynthesis of disorazole Z.

**FIG 4:**
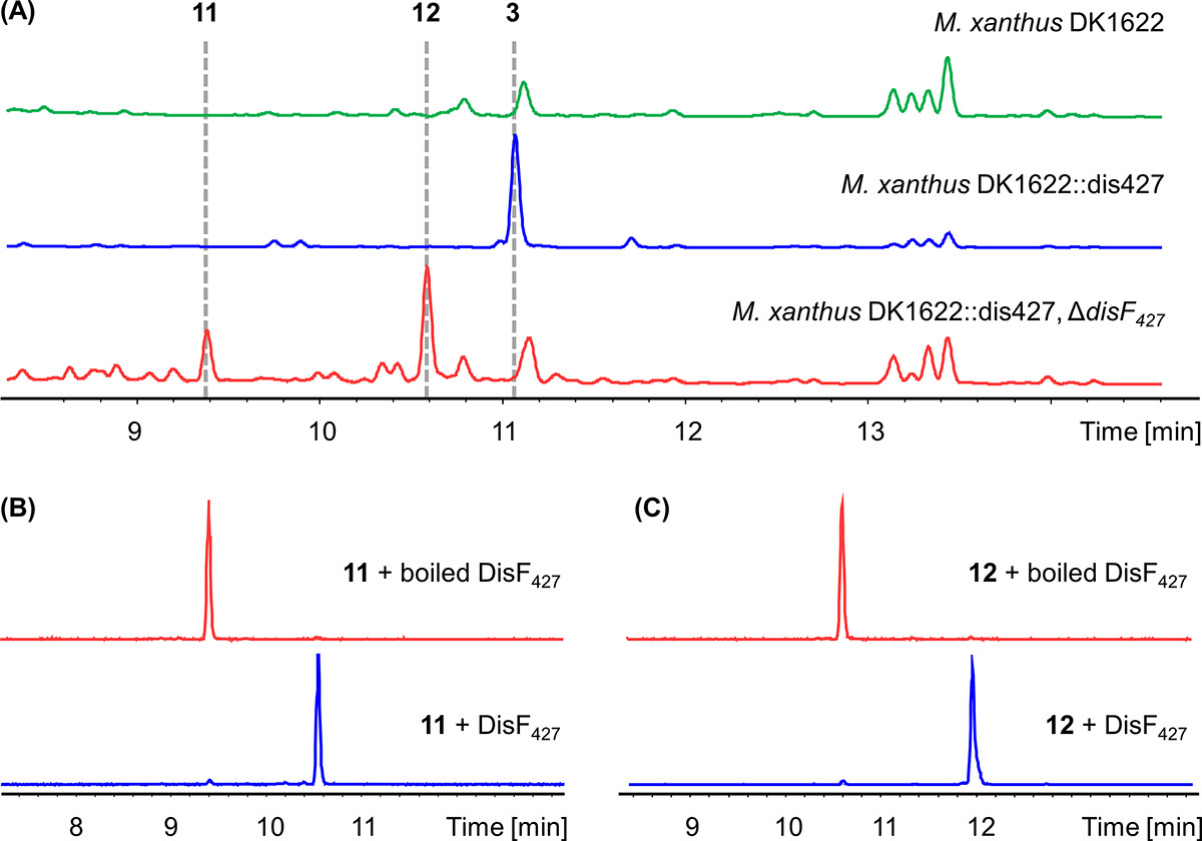
HPLC-MS analysis of disorazole Z formation in M. xanthus and *in vitro*. (A) HPLC chromatogram analyzed by UV detection at 320 nm. Blue trace, heterologous production of disorazole Z1 (compound 3) in M. xanthus DK1622 mutant harboring the *dis427* gene cluster. Red trace, heterologous production of disorazole Z9 (compound 11) and Z10 (compound 12) after deletion of *disF_427_*. Green trace, M. xanthus DK1622 wild type. (B) *In vitro* reaction of compound 11 with DisF_427_ (blue trace) and boiled DisF_427_ (red trace), shown as extracted ion chromatograms (EIC) of *m/z* 689.31 ± 0.05 and *m/z* 703.31 ± 0.05. (C) *In vitro* reaction of 12 with DisF_427_ (blue trace) and boiled DisF_427_ (red trace), shown as EIC of *m/z* 705.30 ± 0.05 and *m/z* 719.30 ± 0.05.

Since Sorangium cellulosum strains are slow growing and unsuitable for *in situ* genetic manipulation, we cloned the disorazole Z biosynthetic gene cluster (from *disA_427_* to *disF_427_*) from the genomic DNA of So ce427 for heterologous expression and functional verification. One-step capture of large gene clusters is usually challenging, especially for myxobacteria, due to their complex genomes. Therefore, the gene cluster was divided into three smaller parts for cloning and then assembled into a p15A-cm vector using RecET-mediated linear-linear homologous recombination (LLHR) (Fig. S9) (40). After that, the chloramphenicol resistance gene was replaced by a *km-int* cassette coding for phage Mx8 integrase, which could be employed for site-specific integration of the gene cluster into the genome of the heterologous host M. xanthus DK1622 (41). The *dis427* gene cluster under the control of its native promoters was successfully expressed in M. xanthus DK1622, leading to the production of disorazole Z1 (compound 3) as the primary compound, which was confirmed by HPLC-MS and NMR analysis (Fig. 4A; Table S3; Fig. S17 and S18). However, the production yield is only about 0.2 mg/L in the standard fermentation procedure (see the supplemental material). Replacement of the native promoter before *disA_427_* with a tetracycline-inducible P*tet* promoter resulted in at least a 4-fold increase in heterologous production of compound 3 (Fig. S10). Furthermore, inserting a strong constitutive P*apr* promoter before *disD_427_* led to a further improved yield but not by much. When the gene cluster was under the control of a vanillate-inducible P*van* promoter, a nearly 9-fold increase was achieved (Fig. S10). Nevertheless, the production yield of compound 3 (about 1.8 mg/L) is still much lower than that obtained by using the original producer strain under optimized fermentation conditions, which may be due to the low level of phylogenetic similarity between *Sorangium* and *Myxococcus*. Further systematic genetic engineering to improve the yield using heterologous expression is therefore still required to achieve competitiveness. Nevertheless, this system, for the first time, allows for genetic manipulation of the disorazole Z biosynthesis, as the native host was found to be genetically intractable despite significant efforts.

As mentioned above, the methyltransferase gene was found exclusively in type Z disorazole gene clusters and was thus supposed to be involved in methyl ester formation. In order to verify its function in disorazole Z biosynthesis, we replaced *disF_427_* with a gentamicin resistance gene using Redαβ-mediated linear-circular homologous recombination (LCHR; see the supplemental material) (42). Deletion of *disF_427_* completely abolished disorazole Z1 (compound 3) production in M. xanthus DK1622, whereas the compounds disorazole Z9 (compound 11) and disorazole Z10 (compound 12) accumulated in the culture broth, which was confirmed by HPLC-MS and NMR analysis (Fig. 4A; Tables S12 and S14; Fig. S55 and S61). The methyltransferase DisF_427_ was then expressed and purified as N-terminal His-tagged recombinant protein using E. coli BL21(DE3). Incubating purified DisF_427_ with compound 11 or compound 12 and *S*-adenosyl methionine resulted in almost complete conversion to the corresponding methylated compounds *in vitro* at 30°C in 1 h (Fig. 4B and C). These results clearly demonstrated the methyltransferase DisF to be responsible for methyl ester formation in disorazole Z biosynthesis. The accumulation of compound 12 as the major component in the absence of *disF_427_* also indicated that stereospecific oxidation and subsequent methylation might occur initially on one side of the symmetric substrate. However, it remains unclear how the geminal methyl group was oxidized to the hydroxy group and furthermore to the carboxyl group, which implies novel tailoring biochemical steps and motivates further investigation, which is ongoing in our laboratory.

### Biological activity of disorazole Z.

Disorazole Z1 (compound 3) was tested on a small panel of human cancer cell lines and displayed very pronounced cytotoxic activity, with IC_50_s in the range of 0.07 to 0.43 nM (Table S16). In comparison to the previously described disorazole A1 (compound 1), it showed similar activity, although it tended to be less potent (by a factor of 4 to 5) when tested on hepatocellular carcinoma (HepG2) and osteosarcoma (U-2 OS) cells.

**FIG 5:**
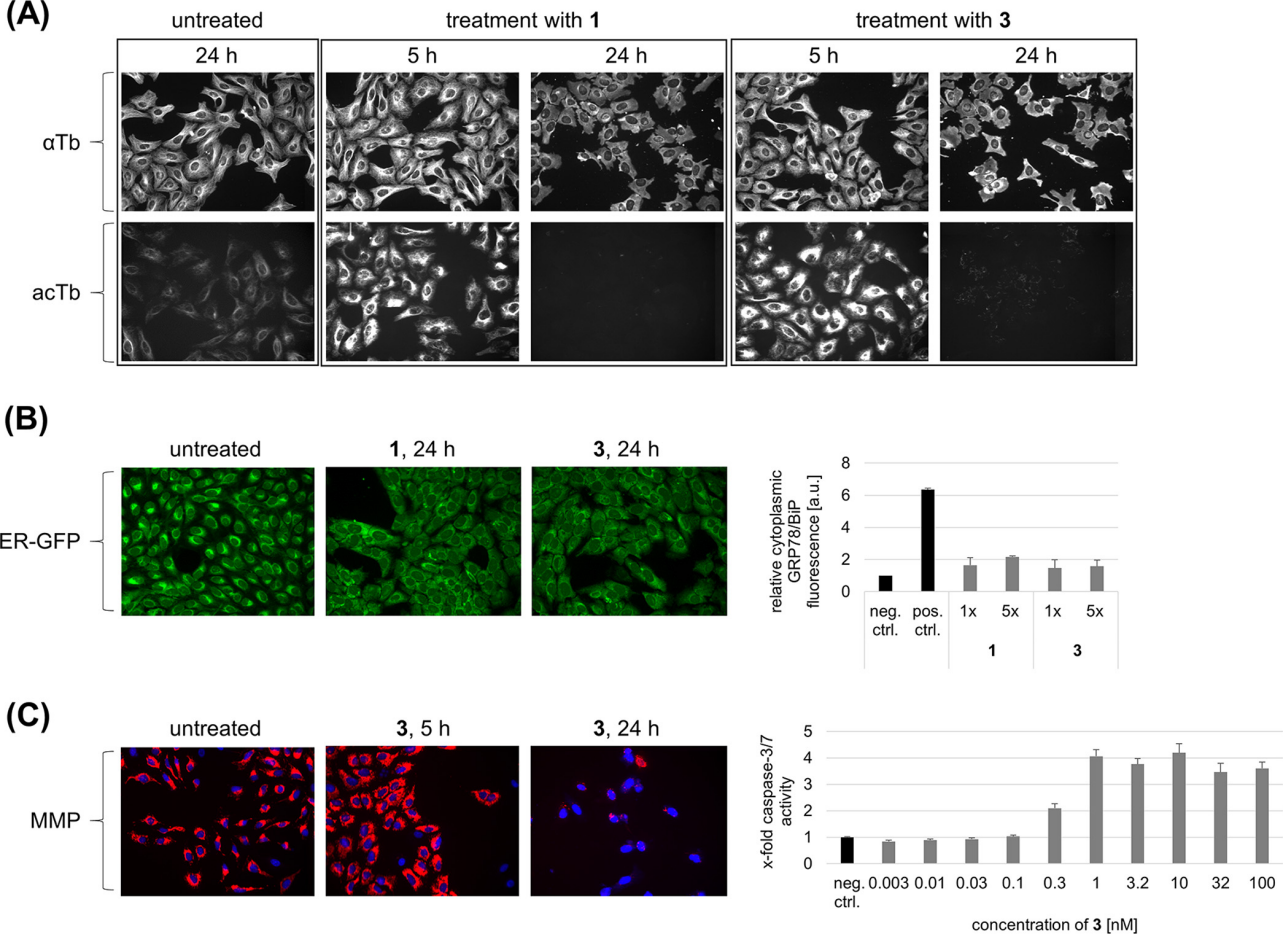
Biological characterization of disorazole Z. (A) Influence on microtubules. U-2 OS cells were treated with disorazole A1 (compound 1) and disorazole Z1 (compound 3) at their respective IC_50_s. α-Tubulin (αTb) and acetylated tubulin (acTb) were visualized by immunofluorescence. The appearance of any artifact lines in these images is a result of cropping these images from a larger microscopy image with built-in stitching (see Materials and Methods). (B) Influence on the ER. (Left) U-2 OS cells were transfected with CellLight ER-GFP for live cell imaging of the ER after treatment with compounds 1 and 3 at their respective IC_50_s. (Right) Relative immunofluorescence quantification of high-content images of the ER stress marker protein GRP78/BiP after 24 h treatment of U-2 OS cells with 1 and 3 at their respective 1-fold and 5-fold IC_50_s. The positive control was 1 μM thapsigargin, and the negative control was untreated (set to 1). Data are means and standard deviations (SD). (C) Induction of the intrinsic apoptosis pathway. (Left) U-2 OS ells were stained with TMRM as the fluorescence marker of the MMP after treatment of U-2 OS with 3 at its 5-fold IC_50_. (Right) Quantification of caspase-3/7 activity in HepG2 cells after treatment with compound 3 in serial dilution. The negative control was untreated (set to 1). Data are means and SD.

In order to explore the effects of disorazole Z on microtubule dynamics, U-2 OS cells were treated with disorazoles followed by immunostaining of α-tubulin and fluorescence microscopy. After 5 h treatment with compound 1 or compound 3, a slightly higher density of interphase microtubules around the nuclear periphery was observed, which resembles a local destabilization. The same effect was already described for, e.g., the microtubule-destabilizing agent vinblastine (43). Interestingly, the acetylated microtubule population, which plays an important role in dynamic cellular processes, was much more affected. This might be caused by the ability of disorazoles to preferentially suppress dynamic mechanisms at the binding sites at the end of microtubules. After prolonged treatment, microtubules were completely depolymerized and the low-abundance acetylated tubulin population was no longer detectable by immunostaining (Fig. 5A). In particular, endoplasmic reticulum (ER) dynamics are directly associated with acetylated microtubules, an effect termed ER sliding (44). Thus, we studied whether specific responses of the ER can be observed after treatment of cells with disorazoles. Both compound 1 and compound 3 induced a delocalization of the ER structure, which coaligns with (depolymerized) microtubules. However, this event does not trigger ER stress, as determined by applying GRP78/BiP immunostaining to disorazole-treated cells (Fig. 5B). The 78-kDa glucose-regulated protein (GRP78) functions as a chaperone and is a master regulator of the unfolded-protein response (UPR) (45), and it is found to be upregulated after treatment of cells with the ER calcium ATPase inhibitor thapsigargin. However, disorazoles did not directly induce ER stress, although dynamics of the ER are probably greatly impaired due to the delocalization of the complex.

To further assess compound 3 and its ability to induce apoptotic processes, the mitochondrial membrane potential (MMP) and caspase activation were determined. Following microtubule depolymerization and a concurrent cell cycle arrest at the G_2_/M checkpoint, many tubulin-binding agents are described as inducing a loss of MMP followed by cytochrome *c* release and activation of the caspase cascade (46). Here, we could demonstrate that treatment of cells with compound 3 at nanomolar concentrations results in mitochondrial swelling (5 h), followed by a complete loss of MMP (24 h). In line with these findings, we found caspase-3/7 activation upon 24 h treatment of cells with compound 3 at concentrations as low as 0.3 nM (Fig. 5C).

### Conclusion and outlook.

In this study, we describe the full structural elucidation of 10 novel disorazole congeners exhibiting a significantly modified basic structure compared to disorazole A and thus grouped as a new subclass of disorazole anticancer drugs termed disorazole Z. This family of compounds possesses a shortened polyketide chain in each half-side of the bis-lactone ring and a carboxymethyl ester at the position where a geminal methyl group is installed in disorazole A. The discovery of the disorazole Z biosynthetic gene cluster and comparison to the disorazole A biosynthetic pathway allowed us to understand the structural differences between these two types of disorazoles. The successful heterologous expression of the disorazole Z gene cluster in an amenable host organism paved the way for detailed biosynthesis studies, e.g., elucidating the intriguing biosynthetic steps involving the oxidation of one methyl center in the geminal dimethyl group, and rational biosynthetic engineering to further improve the yield of disorazole Z. The system will also allow us to generate nonnatural disorazole family compounds through combinatorial biosynthesis. Activity assays of disorazole Z1 and disorazole A1 revealed similar biological activities in cancer cell lines and thus great potential for this family of compounds to be employed as antitumor drugs, a possibility which is being explored by using a peptide-drug conjugate.

## MATERIALS AND METHODS

### General experimental procedures.

Melting points were measured on a Büchi-510 melting point apparatus. UV data were recorded on a Shimadzu UV/Vis-2450 spectrophotometer in methanol (UVASOL, Merck). Infrared (IR) data were recorded on a Bruker Tensor 27 IR spectrophotometer. ^1^H NMR and ^13^C NMR spectra were recorded on Bruker Avance III 700, DMX 600, UltraShield 500 or DPX 300 NMR spectrometers, locked to the deuterium signal of the solvent. Data acquisition, data processing, and spectral analysis were performed with standard Bruker software and ACD/NMRSpectrus. Chemical shifts are given in parts per million, and coupling constants are in hertz. Analytical reverse-phase high-performance liquid chromatography (RP-HPLC) was carried out with an Agilent 1260 HPLC system equipped with a diode-array UV detector (DAD) and a Corona Ultra detector (Dionex) or a maXis ESI time-of-flight (TOF) mass spectrometer (ESI-HRMS; Bruker Daltonics). HPLC was carried out with a Waters Acquity C_18_ column, 100 by 2 mm, 1.7 μm; solvent A was H_2_O–0.1% formic acid, and solvent B was acetonitrile–0.1% formic acid. The gradient was 5% B for 1 min, increasing to 95% B in 18 min; the flow rate was 0.6 mL/min; and the column temperature was 45°C. All elemental formulae were assigned using the high-resolution data of molecular ion clusters measured with a Bruker maXis ESI-TOF mass spectrometer and calculated with the SmartFormula tool of the Compass DataAnalysis program (Bruker). The myxobacterial strain Sorangium cellulosum So ce1875 was isolated in 2001 from soil with plant residues collected near Holbrook, AZ, in 1996 and can be obtained from the DSMZ (German Collection of Microorganisms and Cell Cultures) under the depository number DSM 53600.

### Fermentation of *S. cellulosum* So ce1875.

A fermentation medium (300 L) was inoculated with 10 L precultured *S. cellulosum* So ce1875 in 2-L Erlenmeyer flasks. The fermentation medium contained 0.8% starch (Cerestar), 0.3% soy meal, 0.05% Casitone, 0.02% soy peptone, 0.1% MgSO_4_ · 7H_2_O, 0.075% CaCl_2_ · 2H_2_O, 8 mg/L Na-Fe-EDTA, and 1% Amberlite XAD-16 resin. The pH was 7.3 before autoclaving. Glucose (0.25%) in H_2_O was added after autoclaving. The strain was cultivated at 30°C with a pO_2_ level at 20% for 14 days. At the end of fermentation, the XAD resin was collected from the culture by sieving. The production of 76 mg/L disorazole Z1 (compound 3) was analyzed by HPLC.

### Extraction and isolation of disorazole Z from *S. cellulosum* So ce1875.

The XAD adsorber resin (3.03 kg) from a large-scale (300-L) fermentation of *S. cellulosum* strain So ce1875 was separated from adhering cells by flotation with water before it was eluted in a glass column with 2 bed volumes of 30% aqueous methanol followed by 3 bed volumes of 100% methanol. The methanol eluate was evaporated to an aqueous mixture, diluted with water, and extracted twice with equal portions of ethyl acetate. After evaporation, the aqueous oil was subjected to a 90% methanol-heptane partitioning removing lipid products with three equal portions of heptane. The aqueous oil was diluted with water and extracted with dichloromethane (DCM) to give 72 g crude extract after evaporation of the solvent. Crystallization from ethanol provided 36.7 g of raw crystalline disorazole Z1 (compound 3), including several minor structural variants and about 1.7 equivalents of the solvent.

A portion of 10.7 g raw crystals was dissolved in DCM and toluene and evaporated to dryness twice before the material was subjected to Si flash chromatography (Reveleris silica cartridge, 330 g, 61 by 223 mm [Grace], equilibrated with DCM; flow rate, 90 mL/min; solvent A, DCM; solvent B, acetone; gradient, 0% B for 15 min, to 10% B in 15 min, 50 min at 10% B, to 20% B in 5 min, 10 min at 20% B, to 100% B in 35 min, and 10 min at 100% B). A total of 8.2 g of disorazole compound 3 was obtained in the first peak (UV at 280 nm; evaporative light scattering detector) between 40 and 85 min and crystalized from ethanol to give 6.8 g of disorazole compound 3 as crystals containing 1.7 equivalents of ethanol after drying in a vacuum. Further fractions with disorazole variants were collected.

Fraction 3 (91 to 96 min), 760 mg, was separated by reverse-phase medium-pressure liquid chromatography (RP-MPLC; ODS-AQ column, 30 by 480 mm, 25 μm YMC Ltd.; solvent A, 50% aqueous methanol; solvent B, 100% methanol; gradient, 20% to 34% B in 120 min, to 45% B in 20 min, to 100% B in 10 min; flow rate, 30 mL/min; UV detection, 310 nm) to give fractions according to UV peaks. The fractions were evaporated, and the remaining water was extracted with ethyl acetate, yielding disorazole Z5 (compound 7) (387 mg), disorazole Z6 (compound 8), and disorazole Z1 (compound 3) (35 mg) after evaporation to dryness.

Fraction 4 (97 to 109 min) (428 mg) was separated similarly with a gradient of 20% B to 32% B in 105 min, to give disorazole Z4 (compound 6) enriched (113 mg, amorphous) and 234 mg which was crystallized from ethanol and furnished compound 6 (195 mg, crystallized, containing 1.2 equivalents of ethanol).

Fraction 6 (628 mg) was separated similarly with a gradient of 10% B to 25% B in 130 min to give a main peak of disorazole Z7 (compound 9) (387 mg), which was crystallized from ethanol, yielding 310 mg of white crystals.

Si flash chromatography of mother liquor (38 g) of a disorazole Z crystallization provided further fractions, which were separated by RP-MPLC.

Fraction 12 (2.7 g) was separated by RP-MPLC (column, 60 by 500 mm; YMC ODS-AQ, 120 Å and 21 μm; solvent A, 50% methanol; solvent B, methanol; gradient, 15% B to 26% in 225 min, to 50% B in 120 min; flow rate, 65 mL/min; UV detection, 313 nm) to give disorazole Z8 (compound 10) (593 mg) and *O*-desmethyl-dimethyl-disorazole Z10 (compound 12) (503 mg) as main peaks, recovered after evaporation of the methanol and extraction with ethyl acetate.

Fraction 15 (500 mg) was separated be RP-MPLC (column, 30 by 480 mm, ODS-AQ, 120 Å, 16 μm [YMC]; solvent A, 50% aqueous methanol; solvent B, 100% methanol, gradient, 15% for 3 min, 15% B to 30% B in 173 min; flow rate, 30 mL/min; UV detection, 310 nm) to give a main peak at 125 min with 80 mg of disorazole Z9 (compound 11).

A sample of 6 g disorazole Z from Si flash chromatography was further separated by RP-MPLC in two runs (column, 60 by 500 mm, YMC ODS-AQ, 120 Å, 21 μm; solvent A, 50% methanol; solvent B, methanol; gradient, 20% B for 160 min, to 30% B in 240 min; flow rate, 60 mL/min; UV detection, 313 nm) to give a mixture of disorazoles (680 mg) eluting in front of compound 3. This mixture was again separated by RP-MPLC (column, 30 by 480 mm, ODS-AQ, 15 μm [YMC]; solvent A, 50% aqueous methanol; solvent B, 100% methanol; gradient, 30% B for 47 min, 30% B to 40% B in 40 min, to 100% B in 30 min; flow rate, 30 mL/min; UV detection, 310 nm) to give disorazole Z3 (compound 5) (284 mg). Stepwise crystallization from methanol-water gave about 200 mg of compound 5.

Enriched disorazole Z1 (compound 3) (2.33 g) from Si flash chromatography was separated by RP-MPLC (column, 60 by 500 mm, YMC ODS-AQ, 120 Å, 21 μm; solvent A, 50% methanol; solvent B, methanol; gradient, 20% B for 160 min, to 30% B in 240 min; flow rate, 60 mL/min; UV detection, 313 nm) to give compound 3 and a mixture of disorazoles (48 mg) eluting after compound 3. This fraction was separated by preparative thin-layer chromatography (four plates; 20 by 20 cm; silica gel, 0.5 mm; solvent, DCM-methanol [MeOH] [19:1], UV detection, 254 nm) to give compound 3 after elution with the same solvent mixture and compound 5 at a higher *R*_f_ value (19 mg).

Disorazole Z1 (compound 3): C_40_H_46_N_2_O_12_ M = 746.80; *t_R_* = 11.09 min; crystallized from ethanol with 1.7 equivalent ethanol C_40_H_46_N_2_O_12_–1.7 C_2_H_6_O_1_ M = 825.12; mp.128 to 130°C; [α]^22^_D_ +169.8 (*c *= 1.01, CH_3_OH); UV (MeOH) (amorph): λ_max_ (log ε) 314 (4.948), 340 (sh) nm; UV (chloroform) (crystallized): λ_max_ (log ε) 315 (4.969), 322, 340 (sh) nm; IR (KBr) (amorph): ν_max_ 3486 (m), 2950 (m), 1730 (s), 1614 (m), 1312 (m), 1234 (m), 1153 (m), 1115 (s), 996 (s), 973 (m) cm^−1^; NMR data, see Table S1; ESI-HRMS *m/z* 747.3132 [M+H]^+^ (calculated for C_40_H_47_N_2_O_12_, 747.3123), *m/z* 769.2949 [M+Na]^+^ (calculated for C_40_H_46_N_2_O_12_Na, 769.2943), *m/z* 729.3022 [M-H_2_O+H]^+^ (calculated for C_40_H_45_N_2_O_11_, 729.3018).

Disorazole Z2 (Δ^7,8^-*cis*-disorazole Z) (compound 4): C_40_H_46_N_2_O_12_ M = 746.80; *t_R_* = 11.17 min; crystallized from methanol; mp.133 to 135°C.; [α]^22^_D_ +305 (*c *= 0.41, CHCl_3_); UV (chloroform) (crystallized): λ_max_ (log ε) 313 (5.014), 299, 342 (sh) nm; NMR data, see Table S4; ESI-HRMS *m/z* 747.3128 [M+H]^+^ (calculated for C_40_H_47_N_2_O_12_, 747.3124), *m/z* 769.2944 [M+Na]^+^ (calculated for C_40_H_46_N_2_O_12_Na, 769.2943), *m/z* 729.3020 [M-H_2_O+H]^+^ (calculated for C_40_H_45_N_2_O_11_, 729.3018).

Disorazole Z3 (Δ^9,10^-*trans*-disorazole Z) (compound 5): C_40_H_46_N_2_O_12_ M = 746.80; *t_R_* = 10.85 min; [α]^22^_D_ –59.6 (*c *= 0.12, CHCl_3_); UV (CHCl_3_): λ_max_ (log ε) 314 (4.948), 340 (sh) nm; NMR data, see Table S5; ESI-HRMS *m/z* 747.3123 [M+H]^+^ (calculated for C_40_H_47_N_2_O_12_, 747.3124), *m/z* 769.2935 [M+Na]^+^ (calculated for C_40_H_46_N_2_O_12_Na, 769.2943), *m/z* 729.3012 [M-H_2_O+H]^+^ (calculated for C_40_H_45_N_2_O_11_, 729.3018).

Disorazole Z4 (7,8-epoxy-disorazole Z) (compound 6): C_40_H_46_N_2_O_13_ M = 762.80; *t_R_* = 10.09 min; m.p.132 to 138°C; [α]^22^_D_ +170.9 (*c *= 0.23, CHCl_3_); UV (CHCl_3_): λ_max_ (log ε) 266 (4.601), 328 (4.652), 344 (4.558), 276, 314 (sh) nm; NMR data, see Table S6; ESI-HRMS *m/z* 763.3074 [M+H]^+^ (calculated for C_40_H_47_N_2_O_13_ 763.3073), *m/z* 785.2896 [M+Na]^+^ (calculated for C_40_H_46_N_2_O_13_Na, 785.2892), *m/z* 745.2975 [M-H_2_O+H]^+^ (calculated for C_40_H_45_N_2_O_12_, 745.2967), *m/z* 727.2866 [M-2H_2_O+H]^+^ (calculated for C_40_H_43_N_2_O_11_, 727.2861), *m/z* 807.2991 [M+HCOOH-H]^−^ (calculated for C_41_H_47_N_2_O_15_, 807.2982).

Disorazole Z5 (9,10-epoxy-disorazole Z) (compound 7): C_40_H_46_N_2_O_13_ M = 762.80; *t_R_* = 9.87 min; m.p.137 to 148°C; [α]^22^_D_ +135.1 (*c *= 0.28, CHCl_3_); UV (CHCl_3_): λ_max_ (log ε) 301 (4.823), 345 (4.416), 291, 323 (sh) nm; NMR data, see Table S7; ESI-HRMS *m/z* 763.3085 [M+H]^+^ (calculated for C_40_H_47_N_2_O_13_ 763.3073), *m/z* 780.3351 [M+NH_4_]^+^ (calculated for C_40_H_50_N_3_O_13_, 780.3338), *m/z* 785.2903 [M+Na]^+^ (calculated for C_40_H_46_N_2_O_13_Na, 785.2892), *m/z* 1542.6339 [2M+NH_4_]^+^ (calculated for C_80_H_96_N_5_O_26_ 1542.6338), *m/z* 745.2977 [M+H-H_2_O]^+^ (calculated for C_40_H_45_N_2_O_12_ 745.2967).

Disorazole Z6 (27,28-epoxy-disorazole Z) (compound 8): C_40_H_46_N_2_O_13_ M = 762.80; *t_R_* = 10.04 min; m.p.121 to 125°C; [α]^22^_D_ +20.3 (*c *= 0.272, CHCl_3_); UV (CHCl_3_): λ_max_ (log ε) 315 (4.924), 300, 322, 342 (sh) nm; NMR data, see Table S8; ESI-HRMS *m/z* 763.3079 [M+H]^+^ (calculated for C_40_H_47_N_2_O_13_ 763.3073), *m/z* 785.2898 [M+Na]^+^ (calculated for C_40_H_46_N_2_O_13_Na, 785.2892), *m/z* 745.2971 [M-H_2_O+H]^+^ (calculated for C_40_H_45_N_2_O_12_, 745.2967).

Disorazole Z7 (29-hydroxy-disorazole Z) (compound 9): C_40_H_46_N_2_O_13_ M = 762.80; *t_R_* = 8.96 min; [α]^22^_D_ −11.6 (*c *= 0.23, CHCl_3_); UV (CHCl_3_): λ_max_ (log ε) 316 (4.893), 300, 342 (sh) nm; NMR data, see Table S9; ESI-HRMS *m/z* 763.3075 [M+H]^+^ (calculated for C_40_H_47_N_2_O_13_ 763.3073), *m/z* 785.2883 [M+Na]^+^ (calculated for C_40_H_46_N_2_O_13_Na, 785.2892).

Disorazole Z8 (31-*O*-desmethyl-disorazole Z) (compound 10): C_39_H_44_N_2_O_12_ M = 732.77; *t_R_* = 9.38 min; [α]^22^_D_ +305.2 (*c *= 0.41, CHCl_3_); UV (MeOH) (amorph): λ_max_ (log ε) 316 (4.954), 300, 343 (sh) nm; NMR data, see Table S10; ESI-HRMS *m/z* 733.2958 [M+H]^+^ (calculated for C_39_H_45_N_2_O_12_, 733.2967), *m/z* 755.2779 [M+Na]^+^ (calculated for C_39_H_44_N_2_O_12_Na, 755.2786), *m/z* 715.2866 [M-H_2_O+H]^+^ (calculated for C_39_H_43_N_2_O_11_, 715.2861).

Disorazole Z9 (31-*O*-desmethyl-39-hydroxy-disorazole Z) (compound 11): C_38_H_44_N_2_O_11_ M = 704.76; *t_R_* = 9.35 min; [α]^22^_D_ +55.8 (*c *= 0.26, CHCl_3_); UV (CHCl_3_): λ_max_ (log ε) 317 (4.942), 300, 344 (sh) nm; NMR data, see Table S11; ESI-HRMS *m/z* 705.3023 [M+H]^+^ (calculated for C_38_H_45_N_2_O_11_ 705.3018), *m/z* 687.2920 [M-H_2_O+H]^+^ (calculated for C_38_H_43_N_2_O_10_, 687.2912), *m/z* 1409.5975 [2M+H]^+^ (calculated for C_76_H_89_N_4_O_22_, 1409.5963).

Disorazole Z10 (*O*-desmethyl-dimethyl-disorazole Z) (compound 12): C_38_H_44_N_2_O_10_ M = 688.76; *t_R_* = 10.59 min; [α]^22^_D_ +25.4 (*c *= 0.24, CHCl_3_); UV (CHCl_3_): λ_max_ (log ε) 316 (4.825), 300, 342 (sh) nm; NMR data, see Table S13; ESI-HRMS *m/z* 689.3057 [M+H]^+^ (calculated for C_38_H_44_N_2_O_10_ 689.3069), *m/z* 671.2952 [M-H_2_O+H]^+^ (calculated for C_38_H_43_N_2_O_9_, 671.2963).

### Preparation of disorazole Z Mosher esters.

To prepare disorazole Z (*S*)-Mosher ester (13), 20 mg of crystalline disorazole Z1 (compound 3) was twice dissolved in pyridine and toluene and evaporated to dryness. The residue was dissolved in 0.2 mL of dry pyridine. Twenty-five microliters of (*R*)-(−)-α-methoxy-α-trifluoromethylphenylacetyl chloride was added in three portions to the stirred solution for 24 h. The mixture was dissolved with pyridine and sodium hydrogen carbonate solution (1%), extracted with DCM, washed with water twice, and dried by evaporation with toluene. The residue was purified by Si flash chromatography (12 g silica gel, 40 μm; Reveleris [Grace]; solvent A, petroleum ether; solvent B, ethyl acetate; gradient, 0% B for 1 min, in 1 min to 9% B, 9% B for 1 min, in 4.7 min to 36% B, for 2.5 min 36% B; flow rate, 36 mL/min) The main peak was collected and evaporated to dryness, yielding 39 mg of compound 13. C_60_H_60_F_6_N_2_O_16_ M was 1179.1; for NMR data, see Table S2.

The disorazole Z (*R*)-Mosher ester (14) was prepared analogously using (*S*)-(+)-α-methoxy-α-trifluoromethylphenylacetyl chloride, yielding 19 mg of compound 14. C_60_H_60_F_6_N_2_O_16_ M was 1179.1; for NMR data, see Table S2.

### Crystallography and X-ray analysis.

**(i) Crystallographic data of compounds 3, 6, and 7.** Crystals suitable for single-crystal X-ray analysis were obtained from ethanol. The data set for all structures was collected using a Bruker X8 Apex diffractometer. Graphite-monochromated Mo_Kα_ radiation (λ = 0.71073 Å) was used throughout. Data were collected at 133 (2) K (disorazole Z1 [compound 3] = sh3137_a_sq, disorazole Z5 [compound 7] = sh3279) or 152 (2) K (disorazole Z4 [compound 6] = sh3191) and corrected for absorption effects using the multiscan method. The structures were solved by direct methods using SHELXS-97 (47) and were refined by full matrix least-squares calculations on F^2^ (SHELXL2018 [48]) in the graphical user interface ShelXle (49).

**(ii) Refinement.** All non-H atoms were located in the electron density maps and refined anisotropically. C-bound H atoms were placed in positions of optimized geometry and treated as riding atoms. Their isotropic displacement parameters were coupled to the corresponding carrier atoms by a factor of 1.2 (CH, CH_2_) or 1.5 (CH_3_, OH) for compound 3 (sh3137_a_sq) and compound 6 (sh3191). Restraints of 0.84 (0.01) Å were used for O-H bond lengths. For compound 7 (sh3279), the O-bonded H atoms were located in the electron density maps. Their positional parameters were refined using isotropic displacement parameters which were set at 1.5 times the equivalent isotropic displacement parameter (U_eq_) value of the parent atom. Regarding disorder, for compound 7 (sh3279), each oxirane atom is not fully occupied (O4a, 0.76; O4b, 0.24); furthermore, the propylene group of the main compound as well as one solvent ethanol molecule is split over two positions. Their occupancy factors refined to 0.87 and 0.76, respectively, for the major components. For compound 6 (sh3191), two of the solvent ethanol molecules and the propylene residue of the structure were split over two positions. Their occupancy factors refined to 0.55, 0.86, and 0.81 for the major components, respectively. Regarding SQUEEZE, for compound 3 (sh3137_a_sq), the unit cell contains approximately 2 solvent ethanol molecules (occupancy factor less than 1.0 for each ethanol molecule), which were treated as a diffuse contribution to the overall scattering without specific atom positions by SQUEEZE/PLATON.

### Cloning and engineering of the *dis427* gene cluster.

The myxobacterium Sorangium cellulosum So ce427 was cultivated using CYH medium (1.5 g/L Casitone, 1.5 g/L yeast extract, 4 g/L starch, 1 g/L soy meal, 1 g/L glucose, 1 g/L calcium chloride dihydrate, 0.5 g/L magnesium sulfate heptahydrate, 5.96 g/L HEPES, 4 mg/L Na-Fe-EDTA; pH 7.3) at 30°C. Clumpy cells were collected by centrifugation and then homogenized to become a suspension for isolation of genomic DNA according to the published protocol (42). The genomic DNA was treated with RNase A and the appropriate DNA restriction endonuclease (MluCI or BstXI) to eliminate RNA contamination and to release the gene cluster. The digested DNA was recovered by phenolic chloroform extraction and ethanol precipitation and finally dissolved in autoclaved Milli-Q water. The linear cloning vectors (p15A-cm or pBR322-amp) containing homology arms with homology to the end of the released genomic DNA fragment were achieved by PCR. The digested DNA and corresponding cloning vector were then used for electroporation of Escherichia coli GB05-dir expressing RecET recombinase for linear-linear DNA homologous recombination (LLHR) (42). Recombinant plasmids were isolated from antibiotic-resistant colonies and verified by restriction digestion and Sanger/Illumina sequencing. The correct recombinant plasmids p15A-cm-MluCI-dis427 and pBR322-amp-BstXI-dis427 were then digested with MluCI and BstXI, respectively, to release the cloned fragments. These two fragments were then assembled with a PCR-amplified fragment and a p15A-cm vector by LLHR, leading to the generation of plasmid p15A-cm-dis427. To construct p15A-km-int-dis427 and p15A-km-int-Ptet-dis427, the *km-int* or *km-int-*P*tet* cassette (Table S15) was amplified by PCR and electroporated into E. coli GB05-red harboring p15A-cm-dis427 and expressing Redαβ recombinase for linear-circular homologous recombination (LCHR) (42) to replace the *cm* cassette. Similarly, the expression plasmid p15A-km-int-P*van*-dis427 was constructed based on p15A-km-int-dis427 by LCHR using the *apr-*P*van-disA* cassette. p15A-km-int-Ptet-Papr-dis427 and p15A-km-int-Ptet-dis427-gent-delF were constructed based on p15A-km-int-Ptet-dis427 by LCHR using P*apr-disD* and *gent-delF* cassettes, respectively.

### Electroporation of M. xanthus DK1622.

M. xanthus DK1622 was inoculated into CTT medium (10 g/L Casitone, 10 mM Tris-HCl, 8 mM magnesium sulfate, 1 mM potassium phosphate; pH 7.6) and incubated at 30°C with shaking until exponential phase. For preparation of electrocompetent cells, 1.8 mL culture was transferred into a 2-mL microcentrifuge tube with a hole punched in the cap. Cells were centrifuged, washed twice, and finally resuspended in 50 μL autoclaved Milli-Q water. After addition of 1 to 3 μg plasmid DNA, the mixture was then transferred into a 1-mm cuvette. Electroporation was performed at 650 V, 400 Ω, and 25 μF using a Bio-Rad Gene Pulser Xcell electroporation system. The pulsed cells were mixed with 1.6 mL fresh CTT medium and transferred back into the 2-mL microcentrifuge tube. After recovery at 30°C for 6 h in an Eppendorf thermomixer, the cells were mixed with 10 mL CTT soft agar (0.6% agar, supplemented with 50 μg/mL kanamycin) and poured onto a CTT agar plate (1.2% agar, supplemented with 50 μg/mL kanamycin). The plate was incubated at 30°C, and colonies appeared after 4 to 7 days. Kanamycin-resistant colonies were picked out and inoculated into a new CTT agar plate supplemented with 50 μg/mL kanamycin. In order to verify intact integration of the biosynthetic gene cluster, three pairs of primers located at different positions of the gene cluster were used for colony PCR. Cells were scraped from the agar plate, suspended in 50 μL autoclaved Milli-Q water, and incubated at 100°C for 20 min. After centrifugation, 1 to 2 μL of the supernatant was used as the PCR template.

### Heterologous production of disorazole Z in M. xanthus DK1622.

M. xanthus DK1622 mutants that had the integrated disorazole biosynthetic gene cluster were inoculated in 1.6 mL CTT medium supplemented with 50 μg/mL kanamycin in a 2-mL microcentrifuge tube with a hole punched in the cap and incubated at 30°C in an Eppendorf thermomixer for 1 day. After that, 1 mL of the culture was inoculated into 50 mL CTT medium supplemented with 50 μg/mL kanamycin in a 300-mL baffled flask and incubated at 30°C, 180 rpm for 2 days. After addition of inducer (0.5 μg/mL anhydrotetracycline when the P*tet* promoter was used, 1 mM vanillate when the P*van* promoter was used) and 1 mL XAD-16 resin, incubation was continued for 2 more days. Cells and XAD-16 resin were collected by centrifugation and resuspended in methanol for extraction. After filtration, the extracts were dried by rotary evaporation *in vacuo* and redissolved in 1 mL methanol for HPLC-MS analysis using the method described above.

### Isolation of disorazole Z from M. xanthus DK1622 mutants.

For heterologous production of disorazole Z1 (compound 3), the fully grown culture of M. xanthus DK1622::km-int-Ptet-Papr-dis427 or M. xanthus DK1622::km-int-Pvan-dis427 was inoculated 1:100 into 2 L CTT medium supplemented with 50 μg/mL kanamycin in 5-L unbaffled flasks and cultivated at 30°C and 180 rpm for 2 days. After addition of inducer (0.5 μg/mL anhydrotetracycline when the P*tet* promoter was used, 1 mM vanillate when the P*van* promoter was used) and 2% XAD-16 resin, incubation was continued for 3 more days. Cells and XAD-16 resin were collected by centrifugation, lyophilized to dryness, and extracted stepwise with methanol. The methanol extract was concentrated and partitioned with *n*-hexane to remove nonpolar impurity. After evaporation *in vacuo*, the methanol extract was then dissolved in Milli-Q water and extracted twice with equal portions of ethyl acetate. The ethyl acetate extract was evaporated to dryness, redissolved in methanol, and fractionated using Si flash chromatography on a Biotage system. The gradient was set as follows: 0 to 20 column volume (CV), hexane to ethyl acetate; 20 to 40 CV, ethyl acetate to MeOH; 40 to 45 CV, MeOH. Fractions containing targeted compounds were combined and further separated by semipreparative HPLC on a Waters XSelect peptide BEH C_18_ column (250 by 10 mm; 5 μm) using A (Milli-Q water plus 0.1% formic acid) and B (acetonitrile plus 0.1% formic acid) as mobile phases at a flow rate of 5 mL/min and a column temperature of 45°C. Gradient conditions were set as follows: 0 to 4 min, 5% B; 4 to 5 min, to 53% B; 5 to 20 min, 53% B; 20 to 21 min, to 95% B; 21 to 25 min, 95% B; 25 to 26 min, to 5% B; 26 to 30 min, 5% B. Fractions were detected by UV detection at 320 nm and were collected using an AFC-3000 fraction collector based on a retention time of 17.7 min. Heterologous production and purification of disorazole Z9 (compound 11) and Z10 (compound 12) were done in a similar manner using the strain M. xanthus DK1622::km-int-Ptet-dis427-delF. For purification of compound 11 using semipreparative HPLC, separation was achieved using a Waters XBridge peptide BEH C_18_ column (250 by 10 mm; 5 μm) using the following gradient: 0 to 5 min, 5% B; 5 to 15 min, to 40% B; 15 to 25 min, 40% B; 25 to 26 min, to 95% B; 26 to 31 min, 95% B; 31 to 32 min, to 5% B; 32 to 35 min, 5% B. Fractions were detected by UV detection at 320 nm and were collected using an AFC-3000 fraction collector based on a retention time of 21.8 min. Similarly, compound 12 was purified using a modified gradient:0 to 5 min, 5% B; 5 to 15 min, to 33% B; 15 to 50 min, 33% B; 50 to 51 min, to 95% B; 51 to 56 min, 95% B; 56 to 57 min, to 5% B; 57 to 60 min, 5% B. Fractions were detected by UV detection at 320 nm and were collected using an AFC-3000 fraction collector based on a retention time of 46.0 min.

### Purification and *in vitro* reaction of recombinant protein DisF_427_.

The DNA fragment containing *disF_427_* was amplified by PCR using So ce427 genomic DNA as the template and oligonucleotides HisTEV-disF-F and HisTEV-disF-R. The plasmid pHis-TEV (50) was linearized by NcoI/XhoI and assembled with the PCR fragment by Gibson assembly, generating the protein expression construct pHisTEV-disF427. After Sanger sequencing, this construct was electroporated into E. coli BL21(DE3). The resulting strain was cultivated in 300-mL flasks at 37°C overnight in 50 mL LB medium (10 g/L tryptone, 5 g/L yeast extract, 5 g/L sodium chloride; pH 7.0) supplemented with 50 μg/mL kanamycin. Twenty milliliters of overnight culture was used to inoculate 2 L fresh LB medium supplemented with 50 μg/mL kanamycin in a 5-L flask. After cultivation at 37°C until the optical density at 600 nm (OD_600_) was about 0.6, the culture was cooled to 16°C, induced with IPTG (isopropyl-β-d-thiogalactopyranoside) at a final concentration of 0.1 mM and then further cultivated at 16°C overnight. Cells were harvested by centrifugation at 4°C, resuspended in ice-cold lysis buffer (25 mM Tris, 200 mM NaCl, 10% glycerol, 20 mM imidazole; pH 8.0) and lysed using a continuous-flow cell disrupter (Constant Systems) at 25,000 lb/in^2^ and 4°C. After centrifugation at 23,500 rpm and 4°C for 30 min, the cell debris was removed and the supernatant was loaded onto a 5-mL HisTrap HP column (GE Healthcare) for nickel affinity chromatography using the ÄKTA protein purification system. Fractions containing recombinant protein in elution buffer (25 mM Tris, 200 mM NaCl, 10% glycerol, 250 mM imidazole; pH 8.0) were collected and loaded onto a HiPrep 26/10 desalting column (GE Healthcare) to remove imidazole using desalting buffer (25 mM Tris, 200 mM NaCl, 10% glycerol; pH 8.0). The eluents were collected, concentrated, and stored at −80°C.

The *in vitro* reaction was carried out at 30°C for 1 h in a 50-μL mixture containing 25 mM Tris, 200 mM NaCl, 2 mM MgCl_2_, 2 mM *S*-adenosyl methionine, 5 μL (about 1 mg/mL) recombinant protein, 0.5 μL (1 mg/mL in MeOH) disorazole Z9 (compound 11) or Z10 (compound 12). Boiled (100°C for 10 min) recombinant protein was used as a negative control. After addition of 50 μL MeOH, the mixture was vortexed and centrifuged at 15,000 rpm for 15 min. Two microliters of the supernatant was used for HPLC-MS analysis.

### Biological characterization.

**(i) IC_50_.** Cell lines were obtained from the German Collection of Microorganisms and Cell Cultures (DSMZ) or the American Type Culture Collection (ATCC) and were handled according to standard procedures as recommended by the depositor. Cells were seeded in 96-well plates and treated with disorazole A1 (compound 1) and disorazole Z1 (compound 3) at serial dilutions for 48 h. Viability was determined by adding resazurin sodium salt for 3 h. Fluorescence measurements were performed using a SpectraMax T5 plate reader (Molecular Devices). Readouts were referenced and IC_50_s were determined by sigmoidal curve fitting using OriginPro software. Data for compound 3 were determined as duplicates in three independent experiments.

**(ii) Immunostaining and high-content imaging.** U-2 OS cells were seeded in 96-well imaging plates. After overnight equilibration, the cells were treated with compound 1 and compound 3 as assigned and incubated for up to 24 h. Cells were fixed with cold (−20°C) acetone-MeOH (1:1) for 10 min. After being washed with phosphate-buffered saline (PBS), the cells were permeabilized with 0.01% Triton X-100 in PBS. The following primary antibodies (Sigma) were used: α-tubulin monoclonal antibody (MAb), acetylated tubulin MAb, and GRP78/BiP MAb. For labeling, cells were incubated with primary antibody for 45 min at 37°C, followed by incubation with the secondary antibody (Alexa Fluor 488 goat anti-mouse or anti-rabbit immunoglobulin; Molecular Probes) under the same conditions. After cells were washed with PBS, the nuclear stain Hoechst 33342 (5 μg/mL) was applied for 10 min. Samples were imaged on an automated microscope (BD Pathway 855) suitable for high-content screening. In order to capture full-width pictures of a larger area, a built-in stitching technique was used to combine multiple frames. A representative part of the larger microscopy image was cropped for illustrations. In case of GRP78 immunostaining, fluorescence intensity was determined in the cytoplasmic segment as defined by a ring around nuclei. The relative intensity of GRP78 fluorescence in this region of interest was used as a measure for ER stress/UPR.

**(iii) Live-cell imaging of the ER.** U-2 OS cells were seeded in 96-well imaging plates and transfected using CellLight ER-GFP (Thermo Fisher Scientific) according to the manufacturer’s protocol. After treatment with compound 1 and compound 3 in triplicate as indicated, live cells were imaged on an automated microscope (BD Pathway 855).

**(iv) MMP.** U-2 OS cells were seeded at 8 × 10^3^ cells/well in 96-well imaging plates and were treated after overnight equilibration with compound 3 as assigned. Following treatment, the cells were washed twice with PBS and 100 μL of a staining solution (50 nM tetramethyl rhodamine methyl ester [TMRM] and 5 μg/mL Hoechst 33342 in assay buffer) was added. The cells were stained for 30 min at 37°C, and after they were washed with assay buffer, the samples were examined on an automated microscope (BD Pathway 855).

**(v) Determination of caspase-3/7 activity.** HepG2 cells were treated with compound 3 as indicated and lysed in buffer containing 25 mM HEPES, 5 mM MgCl_2_, 1 mM EGTA and 0.1% (vol/vol) Triton X-100. Protein lysates were mixed with substrate buffer containing 50 mM HEPES, 0.1% (wt/vol) CHAPS {3-[(3-cholamidopropyl)-dimethylammonio]-1-propanesulfonate}, 1% (wt/vol) sucrose, 0.2% (wt/vol) dithiothreitol (DTT) and 0.05 mM Ac-DEVD-7-amino-4-trifluoro-methylcoumarin (AFC). Generation of free AFC was determined after 2 h at 37°C by measurement of fluorescence using a SpectraMax T5 plate reader (Molecular Devices). For normalization, protein concentrations were determined by Bradford assay (absorption, 595 nm) using an Ultra microplate reader EL808 (BioTek Instruments). Data were determined in three independent experiments.

### Data availability.

Crystallographic data for the structure have been deposited with the Cambridge Crystallographic Data Centre (CCDC), Cambridge, UK. Copies of the data can be obtained free of charge by referencing the depository number: 2236753 (disorazole Z1 [compound 3]; data block data_sh3137_a_sq; unit cell parameters, a 8.5672 [3] b 20.1022 [6] c 25.8711 [9] P212121), 1834868 (disorazole Z4 [compound 6]; data block data_sh3191; unit cell parameters, a 8.6801 [6] b 20.1469 [16] c 26.259 [2] P212121), and 1834869 (disorazole Z5 [compound 7]; data block data_sh3279; unit cell parameters, a 8.7058 [6] b 20.2194 [15] c 26.000 [2] P212121).
