# Supplementary material for: The Disorazole Z Family of Highly Potent Anticancer Natural Products from Sorangium cellulosum: Structure, Bioactivity, Biosynthesis, and Heterologous Expression
Source: Microbiol Spectr. 2023 Jun 15;11(4):e00730-23. doi: 10.1128/spectrum.00730-23 (PMC10434194; doi:10.1128/spectrum.00730-23)
Supplement: Supplemental file 1 — Supplemental material. Download spectrum.00730-23-s0001.pdf, PDF file, 8.2 MB [file spectrum.00730-23-s0001.pdf]

**Supporting Information**

# The Disorazole Z Family of Highly Potent Anticancer Natural Products from *Sorangium cellulosum*: Structure, Bioactivity, Biosynthesis and Heterologous Expression

Yunsheng Gao+,[a,c,d] Joy Birkelbach+,[a] Chengzhang Fu,[a,d] Jennifer Herrmann,[a] Herbert Irschik,[b] Bernd Morgenstern,[e] Kerstin Hirschfelder,[a] Ruijuan Li,[c] Youming Zhang,[c] Rolf Jansen*,[b] and Rolf Müller*[a,d]

[a] Department of Microbial Natural Products, Helmholtz-Institute for Pharmaceutical Research Saarland (HIPS), Helmholtz Centre for Infection Research and Department of Pharmacy at Saarland University, Campus E8.1, 66123 Saarbrücken, Germany

[b] Department of Microbial Drugs, Helmholtz Centre for Infection Research (HZI), Inhoffenstraße 7, 38124 Braunschweig, Germany

[c] Helmholtz International Lab for Anti-Infectives, Shandong University-Helmholtz Institute of Biotechnology, State Key Laboratory of Microbial Technology, Shandong University, 266237 Qingdao, China

[d] Helmholtz International Lab for Anti-Infectives, Helmholtz Center for Infection Research (HZI), 38124 Braunschweig, Germany

[e] Department of Inorganic Chemistry, Saarland University, Campus C4.1, 66123 Saarbrücken, Germany

[+] These authors contributed equally to this work

[*] To whom correspondence should be addressed:

rolf.mueller@helmholtz-hips.de and rolf.jansen@helmholtz-hzi.de

## Table of Contents

1. **Structure elucidation of disorazole Z congeners** ..... 5

1.1 Structure elucidation of disorazole Z1 ..... 5

1.2 Structure elucidation of disorazole *cis*/*trans*-isomers Z2 and Z3 ..... 9

1.3 Structure elucidation of disorazole epoxides Z4, Z5 and Z6 ..... 12

1.4 Structure elucidation of disorazole carbon skeleton variants Z7-Z10 ..... 18

2. **Supplemental tables** ..... 25

3. **Supplemental figures** ..... 50

4. **References** ..... 104

## List of Tables

**Table S1** NMR data of disorazole Z1 (**3**) in acetone-$d_6$. ..... 5

**Table S2** $^{1}$H- and $^{13}$C-NMR data ($^{1}$H 600 MHz; $^{13}$C 150 MHz) of the (*S*)- and (*R*)-Mosher esters of **3** in $CDCl_3$. ..... 8

**Table S3** Comparison of $^{1}$H and $^{13}$C NMR data of **3** in acetone-$d_6$ isolated from *S. cellulosum* and *M. xanthus*. ..... 9

**Table S4** NMR data of disorazole Z2 (**4**) in acetone-$d_6$[a]. ..... 10

**Table S5** NMR data of disorazole Z3 (**5**) in methanol-$d_4$. ..... 11

**Table S6** NMR data of disorazole Z4 (**6**) in methanol-$d_4$. ..... 13

**Table S7** NMR data of disorazole Z5 (**7**) in methanol-$d_4$. ..... 15

**Table S8** NMR data of disorazole Z6 (**8**) in methanol-$d_4$. ..... 17

**Table S9** NMR data disorazole Z7 (**9**) in methanol-$d_4$. ..... 18

**Table S10** NMR data of disorazole Z8 (**10**) in methanol-$d_4$. ..... 19

**Table S11** NMR data of disorazole Z9 (**11**) in methanol-$d_4$. ..... 20

**Table S12** Comparison of $^{1}$H and $^{13}$C NMR data of **11** in methanol-$d_4$ isolated from *S. cellulosum* and *M. xanthus*. ..... 21

**Table S13** NMR data of disorazole Z10 (**12**) in methanol-$d_4$. ..... 23

**Table S14** Comparison of $^{1}$H and $^{13}$C NMR data of **12** in methanol-$d_4$ isolated from *S. cellulosum* and *M. xanthus*. ..... 24

**Table S15** DNA oligos used in this study. ..... 25

**Table S16** Half-inhibitory concentrations ($IC_{50}$, mean ± SD) of **3** in comparison of **1** on selected human cancer cell lines. ..... 26

**Table S17** Crystal data and structure refinement for sh3137_a_sq (**3**). ..... 27

**Table S18** Atomic coordinates (x $10^4$) and equivalent isotropic displacement parameters (Å$^2$ x $10^3$) for sh3137_a_sq (**3**). ..... 28

**Table S19** Bond lengths [Å] and angles [°] for sh3137_a_sq (**3**). ..... 29

**Table S20** Anisotropic displacement parameters (Å$^2$ x $10^3$) for sh3137_a_sq (**3**). ..... 31

**Table S21** Hydrogen coordinates (x $10^4$) and isotropic displacement parameters (Å$^2$ x $10^3$) for sh3137_a_sq (**3**). ..... 32

**Table S22** Torsion angles [°] for sh3137_a_sq (**3**). ..... 33

**Table S23** Hydrogen bonds for sh3137_a_sq (**3**) [Å and °]. 33
**Table S24** Crystal data and structure refinement for sh3191 (**6**). 34
**Table S25** Atomic coordinates (x $10^4$) and equivalent isotropic displacement parameters ($Å^2$ x $10^3$) for sh3191 (**6**). 35
**Table S26** Bond lengths [Å] and angles [°] for sh3191 (**6**). 36
**Table S27** Anisotropic displacement parameters ($Å^2$ x $10^3$) for sh3191 (**6**). 39
**Table S28** Hydrogen coordinates (x $10^4$) and isotropic displacement parameters ($Å^2$ x $10^3$) for **6**. 40
**Table S29** Torsion angles [°] for sh3191 (**6**). 41
**Table S30** Hydrogen bonds for sh3191 (**6**) [Å and °]. 41
**Table S31** Crystal data and structure refinement for sh3279 (**7**). 42
**Table S32** Atomic coordinates (x $10^4$) and equivalent isotropic displacement parameters ($Å^2$ x $10^3$) for sh3279 (**7**). 43
**Table S33** Bond lengths [Å] and angles [°] for sh3279 (**7**). 44
**Table S34** Anisotropic displacement parameters ($Å^2$x $10^3$) for sh3279 (**7**). 47
**Table S35** Hydrogen coordinates (x $10^4$) and isotropic displacement parameters ($Å^2$ x $10^3$) for **7**. 48
**Table S36** Torsion angles [°] for sh3279 (**7**). 49
**Table S37** Hydrogen bonds for sh3279 (**7**) [Å and °]. 49

## List of Figures

**Figure S1** Crystal structure of **3**-$(EtOH)_2$. 6
**Figure S2** Side chain of disorazol Z1 (**3**) after conformational search and PM3 optimization (right side). 7
**Figure S3** Mosher ester analysis of disorazole Z1 (**3**). 8
**Figure S4** Crystal structure of **6**-$(EtOH)_2$. 14
**Figure S5** Crystal structure of **7**-$(EtOH)_2$. 16
**Figure S6** Model and structure of disorazole Z6 (**8**) with 25*R*,26*R*,27*R*,28*R*-configuration. 16
**Figure S7** The aligned KS domains. 50
**Figure S8** The aligned CP domains. 50
**Figure S9** Cloning and engineering of the *dis427* gene cluster for heterologous expression. 51
**Figure S10** Quantitative analysis of disorazole Z1 (**3**) using HPLC-UV-MS. 52
**Figure S11** $^1$H NMR spectrum of disorazole Z1 (**3**) in acetone-$d_6$ (500 MHz). 53
**Figure S12** $^{13}$C NMR spectrum of disorazole Z1 (**3**) in acetone-$d_6$ (100 MHz). 54
**Figure S13** $^1$H,$^1$H-COSY NMR spectrum of disorazole Z1 (**3**) in acetone-$d_6$. 55
**Figure S14** HSQC-NMR spectrum of disorazole Z1 (**3**) in acetone-$d_6$. 56
**Figure S15** HMBC NMR spectrum of disorazole Z1 (**3**) in acetone-$d_6$. 57
**Figure S16** $^1$H,$^1$H-ROESY NMR spectrum of disorazole Z1 (**3**) in acetone-$d_6$. 58
**Figure S17** Comparison of $^1$H NMR spectra of **3** in acetone-$d_6$ isolated from *S. cellulosum* and *M. xanthus*. 59
**Figure S18** Comparison of $^{13}$C NMR spectra of **3** in acetone-$d_6$ isolated from *S. cellulosum* and *M. xanthus*. 60
**Figure S19** $^1$H NMR spectrum of $\Delta^{7,8}$-*cis*-disorazole Z (**4**) in acetone-$d_6$ (500 MHz). 61
**Figure S20** $^{13}$C NMR spectrum of $\Delta^{7,8}$-*cis*-disorazole Z (**4**) in acetone-$d_6$ (100 MHz). 62
**Figure S21** $^1$H,$^1$H-ROESY NMR spectrum of $\Delta^{7,8}$-*cis*-disorazole Z (**4**) in acetone-$d_6$. 63

**Figure S22** $^{1}H$ NMR spectrum of *Δ*$^{9,10}$-*trans*-disorazole Z (**5**) in methanol-$d_4$ (700 MHz). 64

**Figure S23** $^{13}C$ NMR spectrum of *Δ*$^{9,10}$-*trans*-disorazole Z (**5**) in in methanol-$d_4$ (175 MHz). 65

**Figure S24** $^{1}H$,$^{1}H$-ROESY NMR spectrum of *Δ*$^{9,10}$-*trans*-disorazole Z (**5**) in methanol-$d_4$. 66

**Figure S25** $^{1}H$ NMR spectrum of 7,8-epoxy-disorazole Z (**6**) in methanol-$d_4$ (600 MHz). 67

**Figure S26** $^{13}C$ NMR spectrum of 7,8-epoxy-disorazole Z (**6**) in in methanol-$d_4$ (150 MHz). 68

**Figure S27** $^{1}H$,$^{1}H$-COSY NMR spectrum of 7,8-epoxy-disorazole Z (**6**) in in methanol-$d_4$. 69

**Figure S28** HMQC-NMR spectrum of 7,8-epoxy-disorazole Z (**6**) in in methanol-$d_4$. 70

**Figure S29** HMBC NMR spectrum of 7,8-epoxy-disorazole Z (**6**) in in methanol-$d_4$. 71

**Figure S30** $^{1}H$,$^{1}H$-ROESY NMR spectrum of 7,8-epoxy-disorazole Z (**6**) in methanol-$d_4$. 72

**Figure S31** $^{1}H$ NMR spectrum of 9,10-epoxy-disorazole Z (**7**) in methanol-$d_4$ (600 MHz). 73

**Figure S32** $^{13}C$ NMR spectrum of 9,10-epoxy-disorazole Z (**7**) in in methanol-$d_4$ (150 MHz). 74

**Figure S33** $^{1}H$,$^{1}H$-ROESY NMR spectrum of 9,10-epoxy-disorazole Z (**7**) in methanol-$d_4$. 75

**Figure S34** $^{1}H$ NMR spectrum of 27,28-epoxy-disorazole Z (**8**) in methanol-$d_4$ (600 MHz). 76

**Figure S35** $^{13}C$ NMR spectrum of 27,28-epoxy-disorazole Z (**8**) in in methanol-$d_4$ (150 MHz). 77

**Figure S36** $^{1}H$,$^{1}H$-COSY NMR spectrum of 27,28-epoxy-disorazole Z (**8**) in in methanol-$d_4$. 78

**Figure S37** HMQC-NMR spectrum of 27,28-epoxy-disorazole Z (**8**) in in methanol-$d_4$. 79

**Figure S38** HMBC NMR spectrum of 27,28-epoxy-disorazole Z (**8**) in in methanol-$d_4$. 80

**Figure S39** $^{1}H$,$^{1}H$-ROESY NMR spectrum of 27,28-epoxy-disorazole Z (**8**) in methanol-$d_4$. 81

**Figure S40** $^{1}H$ NMR spectrum of 29-hydroxy-disorazole Z (**9**) in methanol-$d_4$ (600 MHz). 82

**Figure S41** $^{13}C$ NMR spectrum of 29-hydroxy-disorazole Z (**9**) in in methanol-$d_4$ (150 MHz). 83

**Figure S42** $^{1}H$,$^{1}H$-COSY NMR spectrum of 29-hydroxy-disorazole Z (**9**) in in methanol-$d_4$. 84

**Figure S43** HMQC-NMR spectrum of 29-hydroxy-disorazole Z (**9**) in in methanol-$d_4$. 85

**Figure S44** HMBC NMR spectrum of 29-hydroxy-disorazole Z (**9**) in in methanol-$d_4$. 86

**Figure S45** $^{1}H$ NMR spectrum of 31-*O*-desmethyl-disorazole Z (**10**) in methanol-$d_4$ (600 MHz). 87

**Figure S46** $^{13}C$ NMR spectrum of 31-*O*-desmethyl-disorazole Z (**10**) in methanol-$d_4$ (150 MHz). 88

**Figure S47** $^{1}H$,$^{1}H$-COSY NMR spectrum of 31-*O*-desmethyl-disorazole Z (**10**) in in methanol-$d_4$. 89

**Figure S48** HMQC-NMR spectrum of 31-*O*-desmethyl-disorazole Z (**10**) in in methanol-$d_4$. 90

**Figure S49** HMBC NMR spectrum of 31-*O*-desmethyl-disorazole Z (**10**) in in methanol-$d_4$. 91

**Figure S50** $^{1}H$ NMR spectrum of 31-*O*-desmethyl-39-hydroxy-disorazole Z (**11**) in methanol-$d_4$ (600 MHz). 92

**Figure S51** $^{13}C$ NMR spectrum of 31-*O*-desmethyl-39-hydroxy-disorazole Z (**11**) in methanol-$d_4$ (150 MHz). 93

**Figure S52** $^{1}H$,$^{1}H$-COSY NMR spectrum of 31-*O*-desmethyl-39-hydroxy-disorazole Z (**11**) in in methanol-$d_4$. 94

**Figure S53** HMQC-NMR spectrum of 31-*O*-desmethyl-39-hydroxy-disorazole Z (**11**) in in methanol-$d_4$. 95

**Figure S54** HMBC NMR spectrum of 31-*O*-desmethyl-39-hydroxy-disorazole Z (**11**) in in methanol-$d_4$. 96

**Figure S55** Comparison of $^{1}H$ NMR spectra of **11** methanol-$d_4$ isolated from *S. cellulosum* and *M. xanthus*. 97

**Figure S56** $^{1}H$ NMR spectrum of *O*-desmethyl-dimethyl-disorazole Z (**12**) in methanol-$d_4$ (600 MHz). 98

**Figure S57** $^{13}C$ NMR spectrum of *O*-desmethyl-dimethyl-disorazole Z (**12**) in methanol-$d_4$ (150 MHz). 99

**Figure S58** $^{1}H$,$^{1}H$-COSY NMR spectrum of *O*-desmethyl-dimethyl-disorazole Z (**12**) in in methanol-$d_4$. 100

**Figure S59** HMQC-NMR spectrum of *O*-desmethyl-dimethyl-disorazole Z (**12**) in in methanol-$d_4$. 101

**Figure S60** HMBC NMR spectrum of *O*-desmethyl-dimethyl-disorazole Z (**12**) in in methanol-$d_4$. 102

**Figure S61** Comparison of $^{1}H$ NMR spectra of **12** in methanol-$d_4$ isolated from *S. cellulosum* and *M. xanthus*. 103

# 1. Structure elucidation of disorazole Z congeners

## 1.1 Structure elucidation of disorazole Z1

NMR based structure elucidation of disorazole Z1 is explained in the main text.

**Table S1** NMR data of disorazole Z1 (**3**) in acetone-$d_6$.[a]

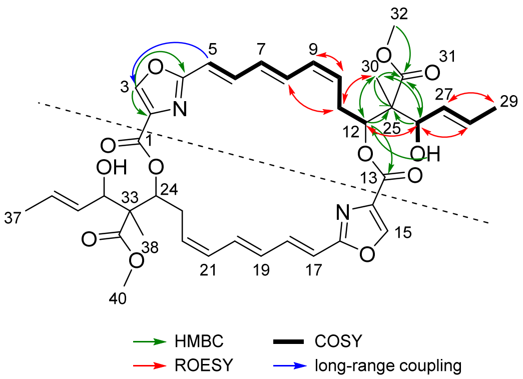


| Pos. | $\delta_H$, m ($J$ [Hz]) | $\delta_C$, type | COSY[b] | ROESY[b] | HMBC[b,c] |
|---|---|---|---|---|---|
| 1/13 | - | 159.76, C | - | - | 12 |
| 2/14 | - | 135.84, C | - | - | 3 |
| 3/15 | 8.53, s | 145.16, CH | 5 | - | ($^1J_{H,C}$ 213 Hz) |
| 4/16 | - | 162.45, C | - | - | 3, 5 |
| 5/17 | 6.19, d (15.5) | 116.61, CH | 3, 6 | overlap with 7 | 7 |
| 6/18 | 6.81, m br. | 137.77, CH | 5, 7 | overlap with 8 | 7, 8 |
| 7/19 | 6.17, dd (14.9, 11.1) | 131.92, CH | 6, 8 | - | 5, 9 |
| 8/20 | 6.77, dd (14.5, 11.9) | 135.10, CH | 7, 9 | 11 | 9, 10 |
| 9/21 | 6.11, t (11.2) | 132.89, CH | 8, 10 | 10 | 7, 11 |
| 10/22 | 5.69, dt br. (11.2, 9.5, 9.5) | 131.19, CH | 11a, 11b, 9 | 9, 11, 12 | 11, 12 |
| 11a/23a | 2.71, m | 30.31, $CH_2$ | 10, 11b, 12 | 8, 30 | 9, 10, 12 |
| 11b/23b | 2.68, m | 30.31, $CH_2$ | 10, 11a, 12 | 8, 10, 12, 26, 30 | 9, 10, 12 |
| 12/24 | 5.45, dd (9.4, 1.5) | 76.28, CH | 11a, 11b | 10, 26, 30 | 9, 11, 30 |
| 25/33 | - | 56.26, C[d]<br>56.24 | - | - | OH, 12, 26, 30 |
| 26/34 | 4.45, dd br. (4.9, 7.6[e]) | 75.65, CH[d]<br>75.52 | OH, 27 | OH, 11, 12, 28, 30 | OH, 27, 28, 29, 30 |
| 27/35 | 5.65, ddquin (15.4, 7.2, 1.3) | 131.69, CH[d]<br>131.66 | 26, 28, 29 | 29 | OH, 26, 29 |
| 28/36 | 5.74, dqd (15.4, 6.5, 0.9) | 128.84, CH | 27, 29 | 26, 29 | 26, 27, 29 |
| 29/37 | 1.70, dd (6.5, 0.9) | 18.06, $CH_3$ | 27, 28 | 27, 28 | 27, 28 |
| 30/38 | 1.36, s | 13.66, $CH_3$ | - | OH, 11, 26 | 12, 26 |
| 31/39 | - | 173.92, C | - | - | 12, 26, 30, 32 |
| 32/40 | 3.60, s | 51.97, $CH_3$ | - | 30 | - |
| 26/34-OH | 4.16, d (4.9) | - | 26 | 26, 30 | - |

[a] $^1H/^{13}C$ at 700/175 MHz; [b] For overview, correlations are given for northern part only, although they are valid for the southern part, too; [c] Carbon showing HMBC correlations to indicated protons; [d] signal doubling ratio 1:1; [e] 7.2 Hz after H/D exchange.

**Relative configuration.** The crystal structure determination shows the constitution of the cyclic molecule with the relative configuration of the stereo centers C-12, C-25 and C-26 (Figure S1, Table S17-Table S23).

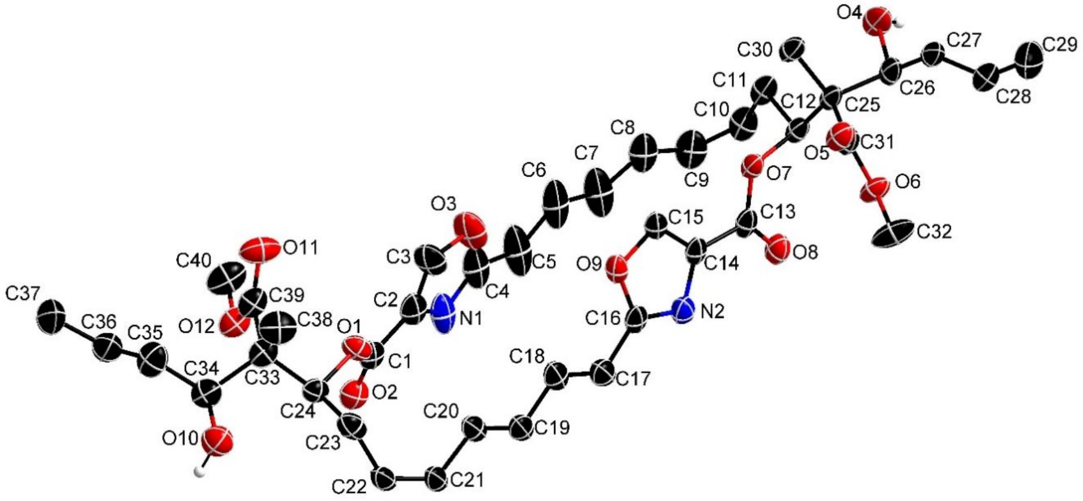


**Figure S1** Crystal structure of **3**-(EtOH)$_2$. **3** = $C_{40}H_{46}N_2O_{12}$ in the crystal (displacement ellipsoids are drawn at 50% probability level). The C bonded hydrogen atoms are omitted for clarity. Both EtOH solvent molecules are squeezed by the program PLATON.

In view of the relative configuration in the crystal, the stereo chemical NMR data, i.e. proton coupling constants and NOE correlations, were accessed critically. Indeed, with a dihedral angle of 179° the X-ray structure was not compatible with the strong NOE between methyl group C-30 and H-26 observed in solution and also not with the absence of a strong NOE between the methyl group C-30 and methine H-27 (Table S1).

In the $^{13}$C NMR spectra in acetone-$d_6$ a signal doubling of C-25 to C-27 revealed the presence of at least two rotamers in a ratio near 1:1 while the $^{1}$H NMR signal of H-26 still appeared as a broad doublet after H/D exchange. Thus, the side chain conformation was explored using the “Conformational Search” module in HyperChem and finally calculated by PM3. The optimized conformation (Figure S2, side chain on the right side) with a dihedral angel of 66° between methyl group C-30 and H-26 now accounted for the observed NOEs (Table S1) by distances of 2.4 or 2.6 Å between methyl C-30 and H-26 or H-11, respectively. Contrary to the conformation in the crystal (Figure S1), the hydroxyl group is freely exposed in the predominant conformer in solution (Table S1, the NOE 30/26 was also the most intensive in DMSO-$d_6$ and $CD_3OD$).

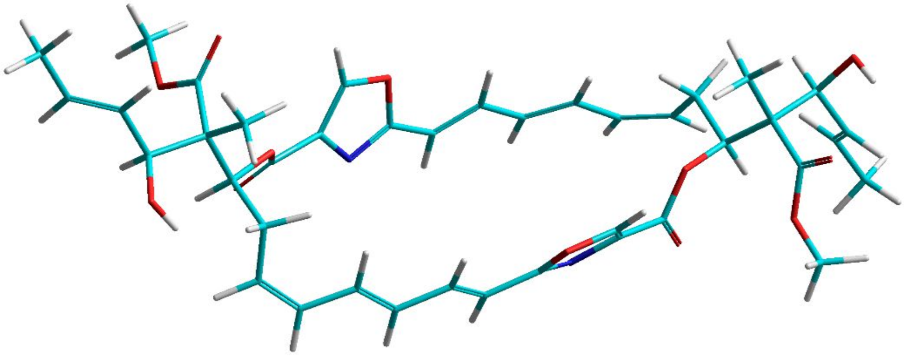

**Figure S2** Side chain of disorazol Z1 (**3**) after conformational search and PM3 optimization (right side).

**Absolute configuration.** Since the X-ray analysis provided only the relative configuration of **3**, the absolute configuration was determined by chemical derivatization and NMR comparison of the α-methoxy-α-trifluoromethylphenyl acetic (MTPA) ester diastereomers, i.e. the application of the Mosher method based upon the $^{1}H$ and $^{13}C$ NMR chemical shift differences.(1–4) The (*S*)- and (*R*)-bis-methoxy-trifluoromethylphenyl acetic acid (MTPA) esters of **3** were easily prepared using an excess of the (*R*)- and (*S*)-MTPA chlorides in pyridine even without any catalyst.

Defined by convention the signs of the $\Delta\delta^{SR} = \delta_S - \delta_R$ values for protons and carbons residing in $R^1$ will be positive and those in $R^2$ will be negative in Figure S3A. Both, the $^{1}H$- and $^{13}C$-NMR shift differences of the disorazole Z (*S*)- and (*R*)-Mosher esters (Table S2) unambiguously indicated that the negative $\Delta\delta^{SR}$ values are located at the end of side-chain (C-27 to C-29), which therefore was assigned as $R^2$. Positive values were observed at positions 10, 11, 12, 30, and 31, all residing in the molecular part $R^1$ (Figure S3B). Consequently, all asymmetric centers (12, 25, 26) of 3 are in the (*S*)-configuration as shown in (Figure S3C).

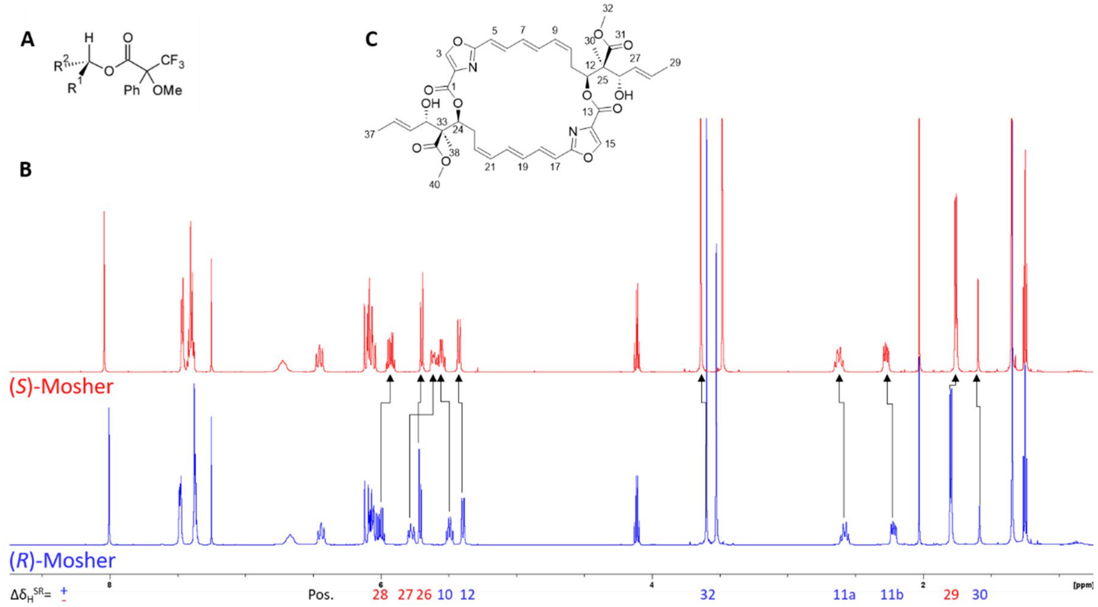


**Figure S3** Mosher ester analysis of disorazole Z1 (**3**). A: Configuration of Mosher esters. B: $^{1}$H NMR spectra and shift differences of (*S*)- and (*R*)-Mosher esters of **3**. C: Absolute configuration of **3**.

**Table S2** $^{1}$H- and $^{13}$C-NMR data ($^{1}$H 600 MHz; $^{13}$C 150 MHz) of the (*S*)- and (*R*)-Mosher esters of **3** in $CDCl_3$.

| Pos. | $\delta_S$ | - | $\delta_R$ | = | $\Delta\delta^{SR}$ | $\delta_{CS}$ | - | $\delta_{CR}$ | = | $\Delta\delta^{SR}$ |
|---|---|---|---|---|---|---|---|---|---|---|
| 10 | 5.57 | | 5.51 | | +0.06 | 129.19 | | 129.15 | | +0.04 |
| 11a | 2.64 | | 2.60 | | +0.04 | 29.47 | | 29.25 | | +0.22 |
| 11b | 2.29 | | 2.24 | | +0.05 | | | | | |
| 12 | 5.44 | | 5.41 | | +0.03 | 74.19 | | 74.12 | | +0.07 |
| 25 | - | | | | | 53.42 | | 53.54 | | -0.12 |
| 26 | 5.72 | | 5.73 | | -0.01 | 80.02 | | 80.05 | | -0.03 |
| 27 | 5.63 | | 5.80 | | -0.17 | 123.86 | | 123.92 | | -0.06 |
| 28 | 5.95 | | 6.02 | | -0.07 | 135.32 | | 135.98 | | -0.66 |
| 29 | 1.78 | | 1.81 | | -0.03 | 17.95 | | 18.03 | | -0.08 |
| 30 | 1.37 | | 1.36 | | +0.01 | 16.29 | | 15.85 | | +0.44 |
| 31 | - | | | | | 171.93 | | 171.71 | | +0.22 |
| 32 | 3.66 | | 3.62 | | +0.04 | 52.24 | | 52.20 | | +0.04 |

Disorazole Z1 (**3**) isolated from the heterologous producer *M. xanthus* DK1622::km-int-Ptet-dis427 was characterized by ESI-HRMS and NMR, accordingly. Comparison of $^{1}$H and $^{13}$C NMR data of **3** isolated from both producers revealed shift differences <0.1 ppm ($\Delta\delta_H{}^b$-$\delta_H{}^c$) and <0.3 ppm ($\Delta\delta_C{}^b$-$\delta_C{}^c$). Thus, both compounds are identical (Table S3, Figure S17 and Figure S18).

**Table S3** Comparison of $^1$H and $^{13}$C NMR data of **3** in acetone-$d_6$ isolated from *S. cellulosum* and *M. xanthus*.[a]

| Pos. | $\delta_H$[b] | $\delta_C$[b] | $\delta_H$[c] | $\delta_C$[c] | $\Delta\delta_H$[b]-$\delta_H$[c] | $\Delta\delta_C$[b]-$\delta_C$[c] |
|---|---|---|---|---|---|---|
| 1/13 | - | 159.76 | - | 159.60 | - | 0.16 |
| 2/14 | - | 135.84 | - | 135.69 | - | 0.15 |
| 3/15 | 8.53 | 145.16 | 8.56 | 145.16 | 0.03 | 0.00 |
| 4/16 | - | 162.45 | - | 162.30 | - | 0.15 |
| 5/17 | 6.19 | 116.61 | 6.17 | 116.49 | 0.02 | 0.12 |
| 6/18 | 6.81 | 137.77 | 6.80 | 137.60 | 0.01 | 0.17 |
| 7/19 | 6.17 | 131.92 | 6.15 | 131.78 | 0.02 | 0.14 |
| 8/20 | 6.77 | 135.10 | 6.78 | 135.02 | 0.01 | 0.08 |
| 9/21 | 6.11 | 132.89 | 6.10 | 132.74 | 0.01 | 0.15 |
| 10/22 | 5.69 | 131.19 | 5.68 | 131.17 | 0.01 | 0.02 |
| 11a/23a | 2.71 | 30.31 | 2.68 | 30.17 | 0.03 | 0.14 |
| 11b/23b | 2.68 | 30.31 | - | - | - | - |
| 12/24 | 5.45 | 76.28 | 5.45 | 76.14 | 0.00 | 0.14 |
| 25/33 | - | 56.26/ 56.24[d] | - | 56.13 | - | 0.13/ 0.1[d] |
| 26/34 | 4.45 | 75.65/ 75.52[d] | 4.43 | 75.60 | 0.02 | 0.05/ 0.08[d] |
| 27/35 | 5.65 | 131.69/ 131.66[d] | 5.64 | 131.58 | 0.01 | 0.11/ 0.08[d] |
| 28/36 | 5.74 | 128.84 | 5.74 | 128.77 | 0.00 | 0.07 |
| 29/37 | 1.70 | 18.06 | 1.70 | 18.00 | 0.00 | 0.06 |
| 30/38 | 1.36 | 13.66 | 1.36 | 13.51 | 0.00 | 0.15 |
| 31/39 | - | 173.92 | - | 173.81 | - | 0.11 |
| 32/40 | 3.61 | 51.97 | 3.61 | 51.88 | 0.00 | 0.09 |
| 26/34-OH | 4.16 | - | 4.22 | - | 0.08 | - |

[a] $^1$H/$^{13}$C at 700/175 MHz; [b] isolated from *S. cellulosum* So ce1875; [c] isolated from *M. xanthus* DK1622::km-int-Ptet-dis427; [d] signal doubling.

## 1.2 Structure elucidation of disorazole *cis*/*trans*-isomers Z2 and Z3

In the analytical RP-HPLC two disorazole Z variants were observed eluting shortly in front and behind disorazole Z1 (**3**). Since ESI-HRMS provided the elemental formulae $C_{40}H_{46}N_2O_{12}$ similar to the mayor compound **3** for both, they were recognized as isomers. Their $^1$H NMR spectra showed two well separated pairs of oxazole signals at about 8.5 ppm, which revealed the asymmetry of their structures. In the NMR data of disorazole Z2 (**4**) (Table S4) the signals of the side chains were nearly unchanged compared to **3** as well as the signals of the south part of the lactone ring (Table S1, Figure S11). In the $^1$H,$^1$H COSY spectrum the full correlation sequence of H-5 to H-12 of the north part could be assigned unambiguously which characterized **4** as a $\Delta^{5,6}$-*trans*, $\Delta^{7,8}$-*cis*, $\Delta^{9,10}$-*trans* isomer. This stereo chemistry was indicated by the vicinal coupling constants of about 15 Hz for the *trans* double bonds and 11 Hz for the *cis* double bond. The $\Delta^{7,8}$-*cis* geometry was additionally shown by the strong NOE correlation between H-6 and H-9 in the $^1$H,$^1$H ROESY spectrum.

**Table S4** NMR data of disorazole Z2 (**4**) in acetone-$d_6$[a].

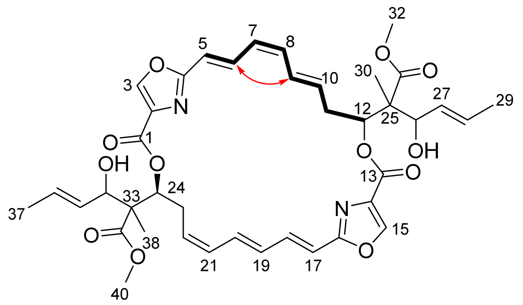

| Pos. | $\delta_H$, m (*J* [Hz]) | COSY | ROESY | $\delta_C$, type | HMBC[b] |
|---|---|---|---|---|---|
| 1 | - | - | - | 159.56, C | 24 |
| 2[c] | - | - | - | 135.59, C | 3 |
| 3 | 8.58, s | 5 | 18, 20 | 145.19, CH | - |
| 4 | - | - | - | 162.13, C | 3, 5, 6 |
| 5 | 6.24, d (15.4) | 3, 6 | 7 | 117.98, CH | 6, 7 |
| 6[c] | 6.81, dd (15.4, 11.7) | 5, 7 | 9 | 131.64, CH | 5, 8 |
| 7 | 5.93, t (11.4) | 6, 8 | 5, 8 | 127.42, CH | 5, 6, 9 |
| 8 | 6.08, t (11.0) | 7, 9 | 7, 10 | 135.13, CH | 6, 9, 10 |
| 9 | 6.49, dd (14.9, 11.6) | 8, 10, 11b | 6, 11b | 130.44, CH | 7, 11ab |
| 10 | 5.83, ddd (15.2, 9.5, 6.1) | 9, 11ab | 8, 11a, 12 | 134.98, CH | 8, 11ab, 12 |
| 11a | 2.79, m | 10, 11b | 10, 11b, 12, 26 | 35.06, $CH_2$ | 9, 10, 12 |
| 11b | 2.31, dt (12.8, 10.6) | 9, 10, 11a | 9, 11a | | |
| 12 | 5.42, dd (11.2, 1.3) | 11ab | 10, 11a, 26, 30 | 76.25, CH | 11ab, 26, 30 |
| 13 | - | - | - | 159.91, C | 12 |
| 14[c] | - | - | - | 135.86, C | 15 |
| 15 | 8.64, s | 17 | 6, 9 | 145.21, CH | - (213.7 Hz) |
| 16 | - | - | - | 162.73, C | 15, 17, 18 |
| 17 | 6.11, d (15.8) | 15, 18 | - | 115.81, CH | 18, 19 |
| 18 | 6.96, dd (15.8, 11.0) | 17, 19 | 20 | 138.83, CH | 17, 19, 20 |
| 19 | 6.13, dd (14.9, 11.4[g]) | 18 | - | 130.83, CH | 17, 21 |
| 20 | 6.87, dd (14.9, 11.6) | 21 | 18, 23 | 135.94, CH | 18, 22 |
| 21 | 6.13, t (11.4[g]) | 20, 22 | 22 | 132.59, CH | 19, 20, 23 |
| 22[d] | 5.70, m | 21, 23 | 21, 23, 24 | 131.52, CH | 20, 21, 23, 24 |
| 23 | 2.71, m | 22, 24 | 20, 24, 34, 38 | 30.11, $CH_2$ | 21, 22, 24 |
| 24 | 5.46, dd (9.9, 1.5) | 23 | 22, 23, 34, 38 | 76.31, CH | 23, 34, 38 |
| 25 | - | - | - | 55.88, C | 11b, 12, 26, 26-OH, 30 |
| 26 | 4.45, m (6.6, 4.4) | 27 | 11a, 12, 28, 30 | 75.22, CH | 12, 27, 28, 30 |
| 26-OH | 4.13, d (4.8) | - | - | - | - |
| 27[d] | 5.61, ddq (15.4, 7.0, 1.5) | 26, 28, 36 | 12, 29, 30 | 131.63, CH | 24, 28 |
| 28[e] | 5.74, m | 27, 29 | 26, 29 | 128.77, CH | 26, 27, 29 |
| 29 | 2.06, m | 28 | 27 | 18.04, $CH_3$ | 27, 28 |
| 30 | 1.35, m | - | 11ab, 12, 26, 27 | 13.46, $CH_3$ | 12, 26 |
| 31 | - | - | - | 173.91, C | 12, 26, 30, 32 |
| 32[f] | 3.64, s | - | 12, 27, 28 | 51.98, $CH_3$ | 12, 26 |
| 33 | - | - | - | 56.33, C | 24, 34, 34-OH, 38 |
| 34 | 4.43, t (6.2) | 34-OH | 23, 24, 36, 38 | 75.64, CH | 24, 35, 36, 38 |
| 34-OH | 4.15, d (4.2) | 34 | - | - | - |
| 35[d] | 5.63, ddq (15.8, 7.0, 1.5) | - | 37, 38 | 131.59, CH | 36, 37 |
| 36[e] | 5.74, m | 37 | 34, 37 | 128.85, CH | 35, 34, 37 |
| 37 | 1.70, d (5.5) | 36 | 35, 36 | 18.04, $CH_3$ | 35, 36 |
| 38 | 1.69, d (5.5) | - | 23, 24, 34, 35 | 13.57, $CH_3$ | 24, 34 |
| 39 | - | - | | 173.91, C | 24, 34, 38, 40 |
| 40[f] | 3.60, s | - | 24, 35 | 51.96, $CH_3$ | - |

[a] 1H/13C at 600/150 MHz; [b] Carbon showing HMBC correlations to indicated protons; [c- f] interchangeable $^{13}$C assignments; [g] after H/D exchange.

In the $^{1}H$ NMR spectrum of $\Delta^{9,10}$-*trans*-disorazole Z (**5**) the clearly separated signals of 17-, 18-, and 20-H with coupling constants of about 15 Hz as well as the shifts of 23-Ha and -Hb suggested a carbon skeleton similar to disorazole Z1 (**3**) for the southern part (Table S1, Figure S11). Although the signals of 21- and 22-H of the double bond were hidden in multiplets, the NOE between 20-H and the methylene group protons at C-23 unambiguously indicated the expected $\Delta^{21,22}$-*cis* double bond geometry. In the northern part a $^{1}H,^{1}H$ COSY sequence of 5-H to 10-H could be established. While 10-H was completely overlapping with H-12 and H-22, the large coupling constants of about 15 Hz in the signals of H-5, -6, -8 and -9 suggested the all-*trans* geometry for the triene. The assignment of the $\Delta^{9,10}$-*trans* bond was supported by a strong NOE between 9-H and the 11-Hb proton of the methylene group, which was shifted high-field to 2.29 ppm.

**Table S5** NMR data of disorazole Z3 (**5**) in methanol-$d_4$.[a]

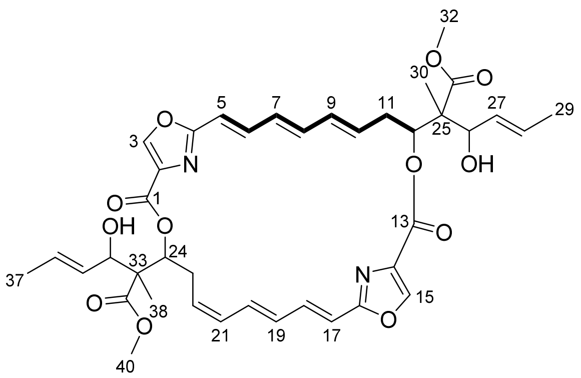


| Pos | $\delta_C$ | type | $\delta_H$ | m (*J* [Hz]) | COSY | ROESY | H in HMBC |
|---|---|---|---|---|---|---|---|
| 1 | 161.26 | C | | | | | 24 |
| 2 | 135.72 | C | | | | | 3 |
| 3 | 146.08 | CH | 8.32 | s | 5 | | - |
| 4 | 163.00 | C | | | | | 3, 5, 6 |
| 5 | 117.49 | CH | 6.30 | d (15.4) | 3, 6 | 7 | 7 |
| 6 | 137.86 | CH | 6.81 | dd (15.4, 11.1) | 5, 7 | 8 | 5, 8 |
| 7 | 133.01 | CH | 6.03 | br dd (14.4, 11.0) | 6, 8 | 5[b] | 5, 9[d] |
| 8 | 137.35 | CH | 6.16 | dd (14.8, 10.8) | 7, 9 | 6, 10 | 6, 9 |
| 9 | 139.01 | CH | 6.06 | br dd (15.3, 10.3) | 8, 10 | 11b[b] | 7, 8, 11ab |
| 10 | 131.64 | CH | 5.56 | br m | 9, 11b | 8, 11a | 8, 11ab |
| 11a | 36.48 | $CH_2$ | 2.71 | m | 11b, 12 | 10, 11b, 12, 26, 30 | 9, 10 |
| 11b | | | 2.29 | td (11.9, 11.2) | 11a, 12 | 9, 11a, 30 | |
| 12 | 76.87 | CH | 5.57 | dd (11.6, 3.2) | 11ab | 11a, 26, 30 | 11b, 26, 30 |
| 13 | 161.80 | C | | | | | 12 |
| 14 | 134.99 | C | | | | | 15 |
| 15 | 145.86 | CH | 8.43 | s | 17 | | - |

| | | | | | | | |
|---|---|---|---|---|---|---|---|
| 16 | 163.35 | C | | | | | 15, 17, 18 |
| 17 | 116.88 | CH | 6.14 | d (15.3) | 15, 18 | | 19 |
| 18 | 139.10 | CH | 6.96 | dd (15.4, 11.3) | 17, 19 | 20 | 17, 19, 20 |
| 19 | 133.01 | CH | 6.05 | dd (14.3, 11.3) | 18, 20 | [c] | 17, 18[d], 21 |
| 20 | 135.06 | CH | 6.71 | br dd (14.8, 11.4) | 19, 21 | 18, 23b | 18, 21, 22 |
| 21 | 133.57 | CH | 6.03 | br t (10.3) | 20, 22 | 22[c] | 19, 20, 23b |
| 22 | 131.78 | CH | 5.56 | t (10.5) | 21, 23a | 21, 23a | 20, 23ab, 24 |
| 23a | 30.93 | $CH_2$ | 2.72 | br d (3.4) | 22, 23b, 24 | 20, 22, 23b, 38 | 21, 22, 24 |
| 23b | | | 2.59 | br dd (13.7, 6.6) | 22, 23a, 24 | 23a, 24, 34, 38 | |
| 24 | 78.01 | CH | 5.52 | dd (10.9, 1.2) | 23ab | 23a, 34, 38 | 23ab, 34, 38 |
| 25 | 56.58 | C | | | | | 11b, 12, 26, 30 |
| 26 | 75.91 | CH | 4.32 | d (7.5) | 27 | 11a, 12, 27, 28, 30 | 27, 28, 29, 30 |
| 27 | 131.27 | CH | 5.60 | ddq (15.3, 7.6, 1.5) | 26, 28 | 26, 29, 30 | 26, 28, 29 |
| 28 | 130.41 | CH | 5.72 | br m | 27, 29 | 26, 29 | 27, 29 |
| 29 | 18.17 | $CH_3$ | 1.71 | dd (6.5, 1.3) | 28 | 27, 28 | 27, 28 |
| 30 | 13.55 | $CH_3$ | 1.33 | s | | 11ab, 12, 26, 27, 32 | 12, 26 |
| 31 | 175.21 | C | | | | | 12, 26, 30, 32 |
| 32 | 52.47 | $OCH_3$ | 3.67 | s | | 30 | - |
| 33 | 56.72 | C | | | | | 24, 34, 38 |
| 34 | 77.32 | CH | 4.30 | d (7.7) | 35 | 23b, 24, 35, 36 38 | 24, 35, 36, 37, 38 |
| 35 | 131.45 | CH | 5.68 | ddq (15.1, 7.7, 1.2) | 34, 36 | 38, 37, 34 | 34, 36, 37 |
| 36 | 130.30 | CH | 5.74 | m | 37, 35 | 37, 34 | 34, 36, 37 |
| 37 | 18.14 | $CH_3$ | 1.74 | dd (6.2, 1.3) | 36 | 35, 36 | 35, 36 |
| 38 | 13.41 | $CH_3$ | 1.45 | s | | 23ab, 34, 24, 35, 40 | 24, 34 |
| 39 | 175.11 | C | | | | | 24, 34, 38, 40 |
| 40 | 52.35 | $OCH_3$ | 3.59 | m | | 38 | - |

[a] $^1$H 700 MHz, $^{13}$C 175 MHz; [b] 7-H close to 9-H; [c] H-19 and H-21 overlap; [d] C-7 and C-19 overlap.

## 1.3 Structure elucidation of disorazole epoxides Z4, Z5 and Z6

A group of three more polar disorazole Z variants with retention times ~ 15-16 min also were isomers which according to their ESI-HRMS-derived elemental formulas $C_{40}H_{46}N_2O_{13}$ contained one additional oxygen. While the NMR data of the mayor structural part of the main representative disorazole Z4 (**6**) (Table S6) were similar to disorazole Z1 (**3**) (Table S1), the COSY NMR spectrum presented a new sequence of correlations for the northern halve which connected completely visible $^1$H signals between 5-H and 12-H. Thus, instead of a triene the isomer **6** contained a 7,8-epoxide ($\delta_C$ 60.4 and 56.5 ppm) flanked by a $\Delta^{5,6}$-*trans* and a $\Delta^{9,10}$-*cis* double bond. The proton signals of H-7 and H-8 ($\delta_H$ 3.25 and 3.76) had a small vicinal coupling constant of 1.7 Hz suggesting a *trans* configuration of the epoxide. This was supported by the strong NO effects between 7-H with 5-H and 9-H on one side and between 8-H and 6-H and 11-$H_2$ on the other side. Assuming the epoxide might be generated by an oxidation of **3** in a late stage of the biosynthesis, the epoxide ring should be directed to the

outside of the bislactone ring and the absolute configuration of both epoxide carbons in **6** should be *R.*

**Table S6** NMR data of disorazole Z4 (**6**) in methanol-$d_4$.[a]

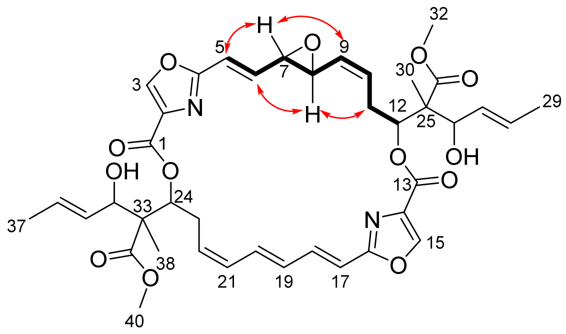


| Pos | $\delta_C$ | type | $\delta_H$ | m (*J* [Hz]) | COSY | ROESY | H in HMBC |
|---|---|---|---|---|---|---|---|
| 1 | 160.61 | C | - | - | - | - | 24 |
| 2 | 135.59 | C | - | - | - | - | 3 |
| 3 | 145.92 | CH | 8.50 | s | 5 | 18, 20 | |
| 4 | 161.84 | C | | - | - | - | 3, 5, 6 |
| 5 | 121.37 | CH | 6.46 | d (15.8) | 3, 6 | 7 | 6, 7 |
| 6 | 137.71 | CH | 6.06 | dd (15.9, 9.4) | 5, 7 | 7, 8, 15 | 5, 7, 8 |
| 7 | 60.42 | CH | 3.21 | dd (9.4, 1.8) | 6, 8 | 5, 6, 8, 9, 10 | 5, 9 |
| 8 | 56.49 | CH | 3.72 | dd (9.7, 1.8) | 7, 9 | 6, 7, 11, 15 | 6, 7, 10 |
| 9 | 131.08 | CH | 5.06 | dd (10.7, 9.9) | 8, 10 | 7, 8 | 7, 8, 11 |
| 10 | 134.04 | CH | 5.85 | ddd (11.0, 10.0, 7.7) | 9, 11 | 7, 11, 12 | 8, 11, 12 |
| 11 | 30.53 | $CH_2$ | 2.66 | m | 10, 12 | 8, 10, 12, 26, 30 | 9, 10, 12 |
| 12 | 77.16 | CH | 5.47 | dd (8.4, 4.0) | | 10, 11, 26, 30 | 9, 11, 26, 30 |
| 13 | 160.65 | C | - | - | - | - | 12 |
| 14 | 135.00 | C | - | - | - | - | 15 |
| 15 | 145.75 | CH | 8.48 | s | - | 6, 8, 26, 40 | - |
| 16 | 163.60 | C | - | - | - | - | 15, 17, 18 |
| 17 | 115.93 | CH | 6.18 | br d (15.4)[c] | 18 | [c] | 18, 19 |
| 18 | 139.36 | CH | 6.58 | dd (15.6, 11.2) | 17, 19 | 3, 20 | 19, 20 |
| 19 | 131.82[b] | CH | 6.19 | br dd (14.7, 11.4)[c] | 18, 20 | [c] | 17, 20, 22 |
| 20 | 136.56 | CH | 6.67 | dd (15.0, 11.4) | 19 | 3, 18, 23 | 18, 19, 22 |
| 21 | 133.29 | CH | 6.17 | br t (11.0)[c] | 22 | 22[c] | 19, 23 |
| 22 | 131.82[b] | CH | 5.76 | br t (9.5) | 21, 23 | 21, 23, 24 | 23, 24 |
| 23 | 30.27 | $CH_2$ | 2.63 | m | 22, 24 | 20, 22, 24, 34, 38 | 21, 22 |
| 24 | 77.77 | CH | 5.42 | dd (8.4, 2.2) | 23 | 22, 23, 34, 38 | 23, 34, 38 |
| 25 | 56.88 | C | - | - | - | - | 12, 26, 30 |
| 33 | 57.02 | C | - | - | - | - | 24, 34, 38 |
| 26, 34 | 76.56 | CH | 4.33 | d (7.3) | 27/35 | 11, 12, 23, 24, 27/35, 28/36, 30/38 | 12, 24, 28/36 |
| 27, 35 | 131.39 | CH | 5.61 | br ddq (15.4, 7.7, 1.60) | 26/34, 28/36 29/37 | 26/34, 29/37, 32/40, 38/35 | 26/34, 28/36, 29/37 |
| 28, 36 | 130.34 | CH | 5.72 | dq (15.3, 6.6) | 27/35, 29/37 | 26/34, 29/37, 32/40, 38/35 | 26/34, 27/35, 29/37 |
| 29, 37 | 18.12 | $CH_3$ | 1.72 | dt (6.2, 1.8) | 27/35, 28/36 | 27/35, 28/36 | 27/35, 28/36 |
| 30, 38 | 13.68 | $CH_3$ | 1.37 | s | - | 11/23, 12, 24, 26/34, 27/35, 28/36 | 12, 24, 26/34, |
| 30, 38 | 13.61 | $CH_3$ | 1.35 | s | - | - | - |
| 31, 39 | 175.16 | C | - | - | - | - | 12, 24, 26/34, 30/38, 32/40 |
| 32, 40 | 52.46 | $CH_3$ | 3.61 | s | - | 26/34, 27/35, 28/36 | - |
| 32, 40 | 52.45 | $CH_3$ | 3.61 | s | - | - | - |

[a] $^1H/^{13}C$ = 600/150 MHZ; [b] double intensity; [c] H-17, -19, -21 overlapping multipletts.

This configuration was proven by X-ray analysis of the 7,8-epoxy-disorazole Z (**6**) after crystallization from ethanol. The crystals contained about two solvent molecules per one molecule of **6**, which are not presented in Figure S4. The X-ray analysis provided a relative configuration shown in Figure S4 (Table S24-Table S30).

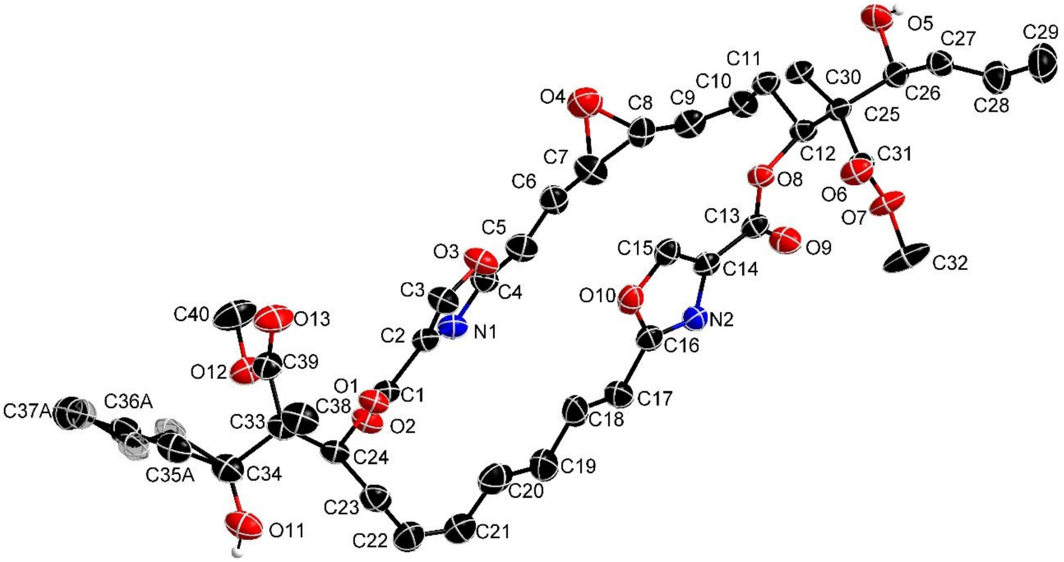


**Figure S4** Crystal structure of **6**-$(EtOH)_2$. **6** = $C_{40}H_{46}N_2O_{13}$ in the crystal (displacement ellipsoids are drawn at 50 % probability level). The C bonded hydrogen atoms and both solvent ethanol molecules are omitted for clarity. The disordered atoms are highlighted with transparency.

The second isomer with the elemental formula $C_{40}H_{46}N_2O_{13}$ was easily recognized as 9,10-epoxide disorazole Z (**7**), because the methylene protons H-11a at 2.76 ppm and the conspicuously high-field shifted H-11b at 1.50 ppm showed COSY correlation with the epoxide proton H-10 (3.27 ppm) (Table S7). The vicinal coupling $J_{9,10}$ ~ 4 Hz was large for epoxides and indicated a *cis* geometry. As proof, a strong NOE between H-8 and 11-Hb was observed, very similar to the NOE between H-20 and the methylene group protons H-23ab, which characterized the *cis* configuration of the $\Delta^{21.22}$- double bond in the southern part of **7**. On the other side of the *cis*-epoxide three clearly visible dd-signals of H-8, H-7 and H-6, which included a vicinal coupling of ~ 15 Hz, showed the *trans* configuration of the diene in the northern part of **7**.

The relative configuration of disorazole Z5 (**7**) was unambiguously shown by the X-ray structure of **7** after crystallization from ethanol (Figure S5, Table S31-Table S37).

**Table S7** NMR data of disorazole Z5 (**7**) in methanol-$d_4$.[a]

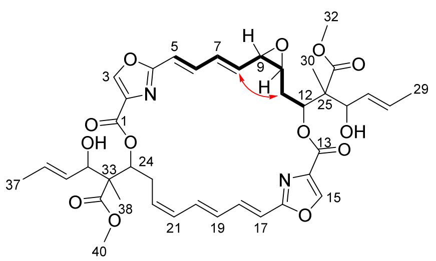

| Pos. | $\delta_H$, m ($J$ [Hz]) | COSY | ROESY | $\delta_C$ | H in HMBC |
|---|---|---|---|---|---|
| 1 | - | - | - | 160.66, C | 24 |
| 2 | - | - | - | 135.58, C | - |
| 3 | 8.42, br s | - | 18, 20, 26/34, 30/38, 32/40 | 145.52, CH | - |
| 2 | - | - | - | 163.00, C | 3, 5, 10 |
| 5 | 6.14, d (15.8) | 10 | 7 | 117.87, CH | 7, 10 |
| 6 | 6.72, dd (15.4, 11.0) | 5, 7 | 8 | 136.82, CH | 7, 8 |
| 7 | 6.41, dd (15.0, 11.0) | 8, 10 | 5, 9 | 136.16, CH | 9[b], 17/5, 18, 22 |
| 8 | 5.68, dd (15.0, 9.9) | 7, 9 | 10, 11b | 135.46, CH | 9, 10 |
| 9 | 3.34, dd (9.9, 4.2) | 8, 10 | 7 | 58.91, CH | 7, 8, 9, 11ab, 12 |
| 10 | 3.27, dt (10.5, 3.8) | 9, 11a/11b | 11a, 12 | 58.91, CH | 7, 8, 9, 11ab, 12 |
| 11a | 2.76, dd (14.7, 3.7) | 10, 11b | 10, 11b, 12, 26 | 32.11, $CH_2$ | 10, 12 |
| 11b | 1.50, dt (14.8, 10.6) | 10, 11a, 12 | 8, 11a | | |
| 12 | 5.57, d (10.6) | 11b | 10, 11a, 26/34 | 74.69, CH | 12, 26, 30 |
| 13 | - | - | - | 160.98, C | 12 |
| 14 | - | | - | 135.85, C | - |
| 15 | 8.37, s | | 8, 10, 26/34, 30/38, 32/40 | 145.63, CH | - |
| 16 | - | - | - | 163.56, C | 15, 17, 18 |
| 17[c] | 6.15, d (15.4) | 18 | 19[c] | 116.28, CH | 18, 19[c] |
| 18 | 6.64, dd (15.6, 10.8) | 17, 19c | - | 139.06, CH | 19[c], 20, 21 |
| 19[c] | 6.16, dd (11.0, 15.0) | 20, 22 | 17[c], 21 | 132.10, CH | 18/20, 21[c] |
| 20 | 6.61, dd (11.2, 6.4) | 18, 19, 21[c] | 23ab | 136.22, CH | 9[b], 17/5, 18, 22 |
| 21[c] | 6.15, t (11.0) | 18, 20 | 19[c], 22 | 133.36, CH | 19[c], 23ab |
| 22 | 5.72, m | 19, 23ab | 21[c], 23ab | 131.69, CH | 20, 23ab, 24 |
| 23 | 2.62, m | 22, 24 | 20, 22, 24, 26/34 | 30.37, $CH_2$ | 21[c], 22, 24 |
| 24 | 5.41, dd (6.4, 4.6) | 23ab | 22, 23ab, 26/34, 27/35, | 77.52, CH | 23ab, 34, 38 |
| 25/33 | - | - | - | 56.90, C | 12/24, 26/34, 30/38 |
| 25/33 | - | - | - | 56.86, C | 12/24, 26/34, 30/38 |
| 26/34 | 4.32, d (7.3) | 27/35 | 23ab, 24, 28/36, 30/38 | 76.70, CH | 27/35, 28/36, 30/38 |
| 26/34 | 4.33, d (7.3) | 27/35 | 11a, 12, 28/36, 30/38 | 76.51, CH | 27/35, 28/36, 30/38 |
| 27/35 | 5.61, ddt (15.2, 7.9, 1.7) | 26/34, 28/36, 29/37 | 24, 29/37 | 131.42, CH | 28/36, 29/37 |
| 27/35 | 5.61, ddt (15.2, 7.9, 1.7) | | | 131.37, CH | 28/36, 29/37 |
| 28/36 | 5.74, m | 27/35, 29/37 | 26/34, 29/37 | 130.42, CH | 10, 26/34, 27/35, 29/37 |
| 28/36 | 5.74, m | | | 130.31, CH | 10, 26/34, 27/35 |
| 29/37 | 1.73, br s | 27/35, 28/36 | 27/35 | 18.12, $CH_3$ | 27/35, 28/36 |
| 30/38 | 1.37, s | - | 23ab, 24, 26/34, 27/35, 28/36 | 13.67, $CH_3$ | 12/24, 26/34 |
| 30/38 | 1.35, s | - | 11ab, 12, 26/34, 27/35, 28/36 | 13.50, $CH_3$ | 12/24, 26/34 |
| 31/39 | - | - | - | 175.18, C | 12/24, 26/34, 30/38, 32/40 |
| 31/39 | - | - | - | 175.04, C | 12/24, 26/34, 30/38, 32/40 |
| 32/40 | 3.62, s | - | 27/35, 28/36 | 52.49, CH | - |
| 32/40 | 3.59, s | - | 27/35, 28/36 | 52.44, CH | - |

[a] $^1$H 600 MHz, $^{13}$C 150 MHz; Because the $^1$H signals of the side chains closely overlap, they are assigned pairwise, and direct CH assignments may be interchanged; [b] C-20/C-18 overlap; [c] H-17, -19, -21 overlap.

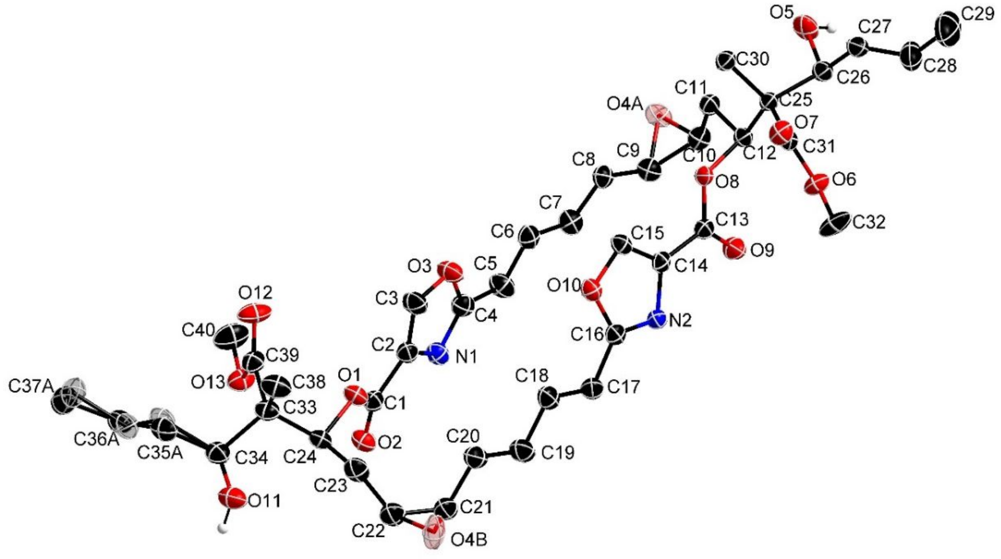


**Figure S5** Crystal structure of **7**-(EtOH)$_2$. **7** = $C_{40}H_{46}$ $N_2O_{13}$ in the crystal (displacement ellipsoids are drawn at 50 % probability level). The C bonded hydrogen atoms and both solvent ethanol molecules are omitted for clarity. The disordered atoms are highlighted with transparency.

The NMR data of the third isomer disorazole Z6 (**8**) with the elemental formula $C_{40}H_{46}N_2O_{13}$ were nearly identical to disorazole Z1 (**3**) except those of one side chain. There, the former $\Delta^{27,28}$ double bond was replaced by an epoxide with characteristic $^1$H ($\delta_H$ 2.87 and 2.92 ppm) and $^{13}$C NMR shifts ($\delta_C$ 61.34 and 54.54 pppm) (Table S8). The vicinal $^1$H coupling constant $J_{27,28}$ = 2.1 Hz was small and thus suggested the trans configuration of the epoxide. This finding was supported by the ROESY correlations observed between methyl group (C-29) and H-27 and between H-28 and H-26. Considering, that the oxidation of the former double bond would occur from the least hindered side, i.e. opposite to the hydroxyl group, the absolute configuration of the epoxide **8** was suggested as 27*S*,28*R* (Figure S6).

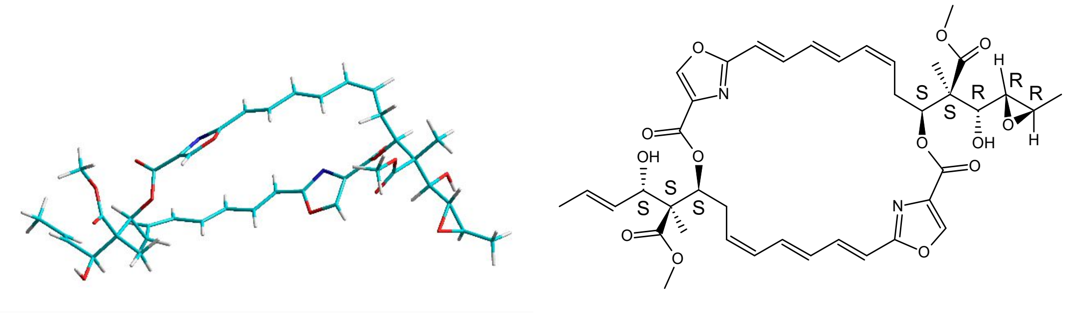


**Figure S6** Model and structure of disorazole Z6 (**8**) with 25*R*,26*R*,27*R*,28*R*-configuration.

**Table S8** NMR data of disorazole Z6 (**8**) in methanol-$d_4$.[a]

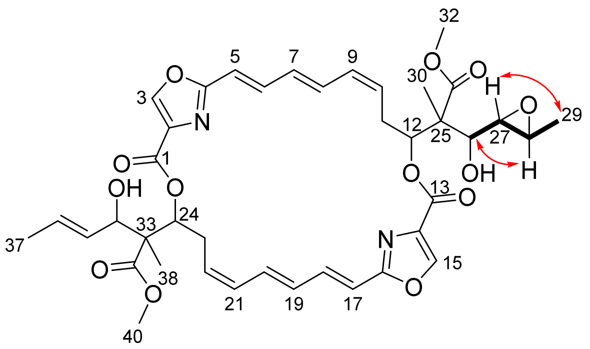


| Pos. | $\delta_H$, m (*J* [Hz]) | COSY | ROESY | $\delta_C$, type | H in HMBC |
|---|---|---|---|---|---|
| 1/13 | - | - | - | 161.03, C | 12/24 |
| 2/14 | - | - | - | 136.00, C | 3/15, 5/17 |
| 2/14 | - | - | - | 135.92, C | 3/15, 5/17 |
| 3 | 8.48, s | - | 34, 40, 38 | 145.94, CH | -[c] |
| 15 | 8.49, s | - | 27, 30, 32 | 146.05, CH | -[c] |
| 4/16 | - | - | - | 163.88, C | n.a. |
| 4/16 | - | - | - | 163.85, C | n.a. |
| 5/17 | 6.13, m | 6/18 | n.a. | 116.59, CH | n.a. |
| 5/17 | - | - | n.a. | 116.52, CH | n.a. |
| 6/18 | 6.79, m | 5/17 | n.a. | 139.41, CH | n.a. |
| 6/18 | - | - | n.a. | 139.47, CH | n.a. |
| 7/19 | 6.13, m | | n.a. | 132.58, CH | n.a. |
| 7/19 | - | - | n.a. | 132.49, CH | n.a. |
| 8/20 | 6.68, m | 7/19, 9/21 | n.a. | 136.29, CH | n.a. |
| 8/20 | - | - | n.a. | 136.34, CH | n.a. |
| 9/21 | 6.13, m | | n.a. | 133.79, CH | n.a. |
| 9/21 | - | - | n.a. | 133.63, CH | n.a. |
| 10/22 | 5.71, m | | n.a. | 131.99, CH | 12/24 |
| 10/22 | - | - | - | 131.72, CH | 12/24 |
| 11a | 2.70, m | | 30 | 31.13, $CH_2$ | 7/19, 10/22, 12 |
| 11b | 2.64, m | 10/22, 12 | 26, 30 | - | - |
| 12 | 5.46, d (10.1) | 11 | 10, 11b, 26[b], 27, 30 | 77.36, CH | 26 |
| 25 | - | - | - | 56.39, C | 12, 26, 30 |
| 26 | 3.70, d (5.9) | 27 | 11b[b], 12, 27, 28, 30 | 75.24, CH | 12, 27, 30 |
| 27 | 2.87, dd (5.9, 2.1) | 26, 28 | 15, 26, 29, 30 | 61.34, CH | 26, 29 |
| 28 | 2.92, qd (5.9, 2.1) | 27, 29 | 26[b], 29 | 54.54, CH | 29 |
| 29 | 1.29, d (5.0) | 28 | 26[b], 27, 28 | 17.86, $CH_3$ | 28 |
| 30 | 1.45, s | - | 11ab, 12, 26, 27 | 14.41, $CH_3$ | 12, 26 |
| 31 | - | - | - | 175.21, C | 12, 26, 30, 32/26 |
| 32 | 3.69, m | - | 11b[b], 12, 27, 28, 30 | 52.99, $CH_3$ | - |
| 23 | 2.61, m | 10/22, 24 | 24, 34, 38 | 30.79, $CH_2$ | 9/21, 24 |
| 24 | 5.44, d (10.2) | 23 | 22, 23, 34, 38 | 77.70, CH | 23, 34, 38 |
| 33 | - | - | - | 57.18, C | 24, 34, 38 |
| 34 | 4.33, d (7.6) | 35 | 3, 23, 24, 35, 36, 38 | 76.92, CH | 24, 30/38 |
| 35 | 5.63, m | | 34, 37, 38 | 131.81, CH | 36, 37 |
| 36 | 5.74, m | 37 | 34, 37, 38, 40 | 130.59, CH | 34, 35, 37 |
| 37 | 1.73, dd (6.3, 1.3) | 10/22, 35, 36 | 35, 36 | 18.43, $CH_3$ | 35, 36 |
| 38 | 1.39, s | - | 3, 23, 24, 34, 35 | 13.98, $CH_3$ | 24, 34 |
| 39 | - | | - | 175.46, C | 24, 34, 38, 40 |
| 40 | 3.69, s | - | 3, 34, 35, 36 | 52.71, $CH_3$ | - |

[a] $^1$H 600 MHz, $^{13}$C 150 MHz; [b] methoxy-32 and H-26 $^1$H signals overlap, however methoxy ROESY correlation signals are expected as very small, like those of methoxy group C-40; [c] carbon signals may be interchanged; n.a. not analyzed due to $^1$H signal overlap.

## 1.4 Structure elucidation of disorazole carbon skeleton variants Z7-Z10

Considerably more polar ($t_R$ = 8.96 min), a fourth isomer disorazole Z7 (**9**) of the elemental formula $C_{40}H_{46}N_2O_{13}$ was identified by HPLC-UV-ESI-HRMS. The NMR data of the isolated compound again only differed from disorazole Z1 (**3**) (Table S1, Figure S11) in one side chain, which instead of a methyl group ended in a primary unsaturated alcohol ($\delta_H$ 4.11, $\delta_C$ 63.0).

**Table S9** NMR data disorazole Z7 (**9**) in methanol-$d_4$.[a]

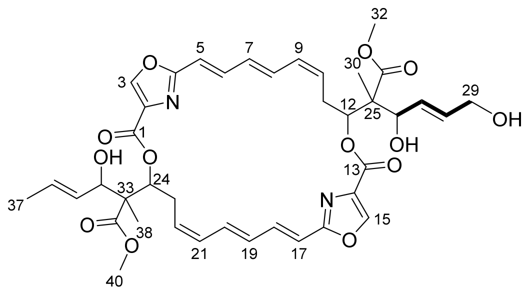


| Pos. | $\delta_H$, m (*J* [Hz]) | COSY | $\delta_C$, type | H in HMBC |
|---|---|---|---|---|
| 1/13 | - | - | 160.82, C | 12[b] |
| 1/13 | - | - | 160.75, C | 12[b] |
| 2/14 | - | - | 135.70, C | - |
| 2/14 | - | - | 135.68, C | - |
| 3/15 | 8.52, s | - | 145.75, CH | - |
| 3/15 | 8.48, s | - | 145.65, CH | - |
| 4/16 | - | - | 163.57, C | 3, 5[b] |
| 5/17 | 6.12, d (15.0) | 6/18 | 116.25, CH | 7[b] |
| 6/18 | 6.79, t br[d] (12.5) | 5/17 | 139.15, CH | 5, 7, 8[b] |
| 7/19 | 6.13, dd (15.0, 9.5) | 8/20 | 132.20, CH | 9[b] |
| 8/20 | 6.68, t br[e] (12.7) | 7/19 | 136.06, CH | 9, 10[b] |
| 9/21 | 6.13, t (10.6) | 10/22 | 133.33, CH | 7, 11[b] |
| 10/22 | 5.70, m | 9/21 | 131.75, CH | 8, 10, 11[b] |
| 10/22 | - | - | 131.70, CH | 8, 10, 11[b] |
| 11/23 | 2.65, m | 10/22, 12/24 | 30.65, $CH_2$ | 9, 10, 12[b] |
| 11/23 | - | - | 30.51, $CH_2$ | 9, 10, 12[b] |
| 12/24 | 5.45, d br (9.1) | 11/23 | 77.43, CH | 9[b], 11, 26, 30, 34 |
| 12/24 | 5.43, d br (9.1) | 11/23 | 77.34, CH | 9[b], 11, 26, 30, 34 |
| 25/33 | - | - | 56.89, C | 12[b], 25[b], 26, 34 |
| 25/33 | - | - | 56.75, C | 12[b], 25[b], 26, 34 |
| 26 | 4.45, d (5.5) | 27, 28 | 75.87, CH | 12, 27, 28 |
| 27 | 5.86, m (15.7, 5.4)[c] | 26, 28 | 130.49, CH | 26, 28, 29 |
| 28 | 5.86, m (15.3, 3.8)[c] | 26, 29 | 134.18, CH | 26, 27, 29 |
| 29 | 4.11, d (3.7) | 26, 27 | 63.00, $CH_2$ | 27, 28 |
| 30 | 1.39, s | - | 14.01, $CH_3$ | 12[b], 26 |
| 34 | 4.33, d (7.7) | 35 | 76.66, CH | 24, 35, 36, 38 |
| 35 | 5.63, ddq (15.4, 7.7, 1.5) | 34, 36, 37 | 131.43, CH | 34, 36, 37 |
| 36 | 5.74, dq (15.4, 6.2) | 35, 37 | 130.34, CH | 34, 35, 37 |
| 37 | 1.73, dd (6.2, 1.1) | 35, 36 | 18.14, $CH_3$ | 35, 36 |
| 38 | 1.38, s | - | 13.69, $CH_3$ | 12[b], 34 |
| 31/39 | - | - | 175.17, C | 12[b], 26, 30[b], 32[b], 34 |
| 32/40 | 3.66, s | - | 52.55, $CH_3$ | - |
| 32/40 | 3.62, s | - | 52.44, $CH_3$ | - |

[a] $^1H$ 600 MHz, $^{13}C$ 150 MHz; [b] correlations are given for the northern part only, although they are valid for the southern part too; [c] from *J*-resolved NMR spectrum; [d] not observed in *J*-resolved NMR spectrum; [e] from *J*-resolved NMR spectrum 11.0, 15.6 Hz.

With a RP-HPLC retention time of 9.38 min, disorazole Z8 (**10**) was the second most polar variant. The elemental formula $C_{39}H_{44}N_2O_{12}$ showed a loss of a methylene compared to disorazole Z1 (**3**). The NMR data of **10** (Table S10) were most similar to **3** for the bislactone ring and one side chain (C-33 - C-40) (Table S1, Figure S11). However, the $^1$H and $^{13}$C spectra contained only one methoxy signal which correlated in the HMBC spectrum with the ester carbon C-39 ($\delta_C$ 175.17). The respective carbon C-31 ($\delta_C$ 176.66) of the second side chain was assigned as carboxylic acid, which only had HMBC correlations with the oxymethines C-12 and C-26 and methyl group C-30.

**Table S10** NMR data of disorazole Z8 (**10**) in methanol-$d_4$.[a]

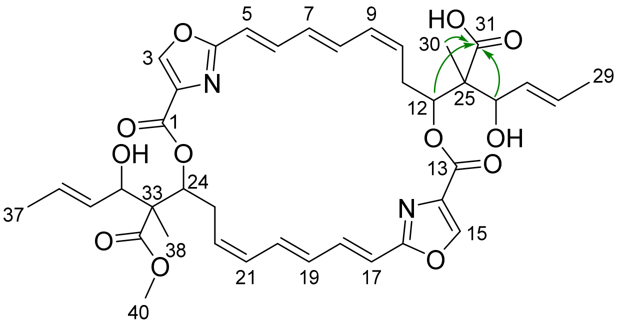


| H | $\delta_H$ | m | J[Hz] | COSY | $\delta_C$, type | H in HMBC |
|---|---|---|---|---|---|---|
| 1/13 | - | | | - | 160.85, C | 12/24 |
| 1/13 | - | | | - | 160.72, C | 12/24 |
| 2/14 | - | | | - | 135.90, C | 3/15 |
| 2/14 | - | | | - | 135.68, C | 3/15 |
| 3/15 | 8.52 | s | - | - | 145.62, CH | - |
| 3/15 | 8.51 | s | - | - | 145.56, CH | - |
| 4/16 | - | | | - | 163.57, C | 3/15, 5/17 |
| 4/16 | - | | | - | 163.49, C | 3/15, 5/17 |
| 5/17 | 6.16 | d | 15.0 | 6/18 | 116.28, CH | 7/19 |
| 5/17 | - | | | 6/18 | 116.20, CH | 7/19 |
| 6/18 | 6.81 | t | 11.7 br. | 5/17, 7/19 | 139.07, CH | 7/19, 8/20 |
| 6/18 | - | | | 5/17, 7/19 | 139.14, CH | 7/19, 8/20 |
| 7/19 | 6.17 | dd | 15.0, 11.0 | 6/18, 8/20 | 133.34, CH | 5/17, 8/20, 9/21 |
| 7/19 | - | | | 6/18, 8/20 | 133.29, CH | 5/17, 8/20, 9/21 |
| 8/20 | 6.72 | dt | 14.7, 11.7 | 7/19, 9/21 | 136.13, CH | 10/22 |
| 8/20 | - | | | 7/19, 9/21 | 136.00, CH | 10/22 |
| 9/21 | 6.16 | t | 11.0 | 8/20, 10/22 | 132.11, CH | 5/17, 7/19, 11b/23b |
| 9/21 | - | | | 8/20, 10/22 | 132.19, CH | 5/17, 7/19, 11b/23b |
| 10/22 | 5.74 | m[b] | - | 9/21, 11/23 | 131.95, CH | 8/20, 11b/23b, 12/24 |
| 10/22 | - | | - | - | 131.67, CH | 8/20, 11b/23b, 12/24 |
| 11/23 | 2.73 | m | - | 10/22, 12/24 | 30.71, $CH_2$ | 7/19, 10/22 |
| 11/23 | 2.66 | m | - | 10/22, 12/24 | 30.50, $CH_2$ | 7/19, 10/22 |
| 12/24 | 5.47 | d | 10.3 | 11/23 | 77.41, CH | 11ab/23ab, 30, 38 |
| 12/24 | - | | | - | 77.38, CH | 11ab/23ab, 30, 38 |
| 25 | - | | | - | 56.31, C | 26, 30 |
| 33 | - | | | - | 56.89, C | 34, 38 |
| 26 | 4.41 | d | 7.3 | 27 | 76.34, CH | 28, 30 |
| 34 | 4.37 | d | 7.7 | 35 | 76.64, CH | 36, 38 |
| 35 | 5.67 | ddq | 15.4, 7.7, 1.6 | 34, 36, 37 | 131.59, CH | 26/34, 28/36, 29/37 |
| 27 | 5.72 | m[b] | | 26, 28 | 131.43, CH | 26/34, 28/36, 29/37 |
| 28 | 5.79 | m[b] | | 27, 29 | 129.99, CH | 26, 29/37 |

| | | | | | | |
|---|---|---|---|---|---|---|
| 36 | 5.79 | m[b] | | 35, 37 | 130.33, CH | 29/37, 34 |
| 29/37 | 1.77 | d | 6.6 | 28, 35, 36 | 18.16, $CH_3$ | 27/35, 28/36 |
| 30 | 1.38 | s | - | - | 13.91, $CH_3$ | 12/24, 26 |
| 31 | - | | | - | 176.66, C | 12/24, 26, 30 |
| 38 | 1.42 | s | - | - | 13.69, $CH_3$ | 12/24, 34 |
| 39 | - | | | - | 175.17, C | 12/24, 34, 38, 40 |
| 40 | 3.66 | s | - | - | 52.45, $CH_3$ | - |

[a] $^1$H 600 MHz, $^{13}$C 150 MHz; [b] with 27, 35, 36, 10/22-H. For comparability reasons side chain numbering starts with position 25 and 33, respectively. Disorazole Z8 (**10**) misses $CH_3$-32.

The variant disorazole Z9 (31-*O*-desmethyl-39-hydroxy-disorazole Z) (**11**) with a retention time of 9.35 min had the elemental formula $C_{38}H_{44}N_2O_{11}$, which showed the formal loss of $C_2H_2O$. The absence of any methoxy group in the NMR data of **11** (Table S11) instantly indicated that both side chains were affected. The data of the lactone core were comparable to disorazole Z1 (**3**), although the symmetry of the molecule and the complete overlap of the respective NMR signals were lost (Table S11, Table S1 and Figure S11). In the side chain of the northern halve a free carboxylic acid at C-31 (176.78 ppm) comparable to the mono ester of **10** was assigned from the NMR data. A second carboxyl carbon was also absent in the $^{13}$C NMR spectrum of **11**. Instead, a new primary alcohol C-39 ($\delta_{H/C}$ 3.61/66.34 ppm) was assigned to its position from the HMBC correlations observed with lactone C-24, quaternary carbon C-33, secondary alcohol C-34, and methyl group C-38. The exchange of a carboxyl substituent against a primary alcohol group resulted in a high-field shift of 10 ppm for the quaternary carbon C-33 ($\delta_C$ 46.91 ppm).

**Table S11** NMR data of disorazole Z9 (**11**) in methanol-$d_4$.[a]

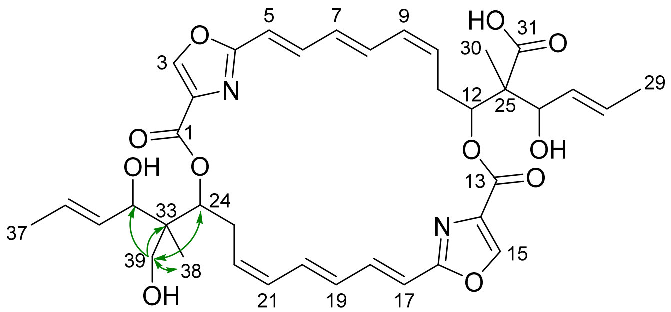


| H | $\delta_H$ | m | *J* [Hz] | ROESY | $\delta_C$, type | H in HMBC |
|---|---|---|---|---|---|---|
| 1 | - | | - | - | 161.70, C | 24 |
| 2/14 | - | | - | - | 136.10, C | 3/15 |
| 2/14 | - | | - | - | 135.95, C | 3/15 |
| 3 | 8.53 | s | - | 34, 39 | 145.58, CH | - |
| 4/16 | - | | - | - | 163.61, C | 3/15, 5/17 |
| 4/16 | - | | - | - | 163.52, C | 3/15, 5/17 |
| 5/17 | 6.13 | m | - | - | 116.20, CH | 7/19 |

| | | | | | | |
|---|---|---|---|---|---|---|
| 5/17 | 6.13 | m | - | - | 115.99, CH | 7/19 |
| 6/18 | 6.84 | m | - | - | 139.31, CH | 7/19, 8/20 |
| 6/18 | 6.84 | m | - | - | 139.17, CH | 7/19, 8/20 |
| 7/19 | 6.13 | m | - | - | 133.27, CH | [b] |
| 7/19 | 6.13 | m | - | - | 132.95, CH | [b] |
| 8/20 | 6.78 | dd | 14.5, 11.9 | - | 136.45, CH | [b] |
| 8/20 | 6.71 | dd | 14.3, 12.1 | - | 136.07, CH | [b] |
| 9/21 | 6.13 | m | - | - | 132.78[b], CH | [b] |
| 10/22 | 5.7 | m | - | - | 132.12[b], CH | [b] |
| 27/35 | 5.7 | m | - | - | 132.05[b], CH<br>131.95[b], CH<br>131.88[b], CH<br>131.63[b], CH | [b]<br>[b]<br>[b]<br>[b] |
| 28/36 | 5.7 | m | - | - | 129.62, CH | 26/34 |
| 28/36 | 5.7 | m | - | - | 129.96, CH | 26/34 |
| 29/37 | 1.75 | br d | 6 | - | 18.18, $CH_3$ | [b] |
| 29/37 | 1.74 | br d | 6.6 | - | 18.15, $CH_3$ | [b] |
| 11a | 2.72 | br dt | 13.7, 10.4 | 11b, 30 | 30.76, $CH_2$ | 12 |
| 11b | 2.62 | dd | 13.9, 7.3 | 11a, 12, 26, 30 | | 12 |
| 12 | 5.45 | d | 10.3 | 11b, 26, 30 | 77.41, CH | 11ab, 26, 30 |
| | - | | | - | 56.29, C | 12, 26, 30 |
| 26 | 4.37 | d | 7.0 | 11, 12, 30 | 76.42, CH | 12, 30 |
| 30 | 1.35 | s | - | 11ab, 12, 26 | 14.06, $CH_3$ | 12, 26 |
| 31 | - | | - | - | 176.78, C | 12, 26, 30 |
| 13 | - | | - | - | 161.03, C | 12 |
| 15 | 8.47 | s | - | 26 | 145.58, CH | - |
| 23a | 2.88 | br dt | 13.4, 10.5 | 23b | 30.40, $CH_2$ | 24 |
| 23b | 2.53 | dd | 13.9, 7.3 | 23a, 24, 34, 38, 39 | | 24 |
| 24 | 5.28 | d | 10.3 | 23b, 34, 38, 39 | 77.52, CH | 23ab, 34, 38, 39 |
| 33 | - | | | - | 46.91, C | 24, 34, 38, 39 |
| 34 | 4.13 | d | 6.6 | 23b, 24, 38, 39 | 76.90, CH | 24, 38, 39 |
| 38 | 1.02 | s | - | 23b, 24, 34, 39 | 15.53, $CH_3$ | 24, 34, 39 |
| 39 | 3.61 | m | - | 23b, 24, 34, 38 | 66.34, $CH_2$ | 24, 34, 38 |

[a] $^1H/^{13}C$ at 600/150 MHz; [b] overlapping signals. For comparability reasons side chain numbering starts with position 25 and 33, respectively. Disorazole Z9 (**11**) misses $CH_3$-32 and $CH_3$-40.

Comparison of $^1H$ and $^{13}C$ NMR data of disorazol Z9 (**11**) isolated from *S. cellulosum* So ce1875 and *M. xanthus* DK1622::km-int-Ptet-dis427-gent-delF revealed a shift differences <0.1 ppm ($\Delta\delta_H^b$-$\delta_H^c$). Thus, both compounds are identical (Table S12, Figure S55). Greater differences of carbon shifts can be explained due to their extraction from HMBC experiments.

**Table S12** Comparison of $^1H$ and $^{13}C$ NMR data of **11** in methanol-$d_4$ isolated from *S. cellulosum* and *M. xanthus*.[a]

| Pos. | $\delta_H^b$ | $\delta_C^b$ | $\delta_H^c$ | $\delta_C^c$ | $\Delta\delta_H^b$-$\delta_H^c$ | $\Delta\delta_C^b$-$\delta_C^c$ |
|---|---|---|---|---|---|---|
| 2/14 | - | 136.1 | - | 136.02 | - | 0.08 |
| 2/14 | - | 135.95 | - | 136.02 | - | 0.07 |
| 4/16 | - | 163.61 | - | 163.53 | - | 0.08 |
| 4/16 | - | 163.52 | - | 163.53 | - | 0.01 |
| 5/17 | 6.13 | 116.2 | 6.12 | 116.02 | 0.01 | 0.18 |
| 5/17 | 6.13 | 115.99 | 6.12 | 116.02 | 0.01 | 0.03 |
| 6/18 | 6.84 | 139.31 | 6.82 | 139.21 | 0.02 | 0.10 |

| | | | | | | |
|---|---|---|---|---|---|---|
| 6/18 | 6.84 | 139.17 | 6.82 | 139.21 | 0.02 | 0.04 |
| 7/19 | 6.13 | 133.27 | 6.12 | 133.17 | 0.01 | 0.10 |
| 7/19 | 6.13 | 132.95 | 6.12 | 133.17 | 0.01 | 0.22 |
| 8/20 | 6.78 | 136.45 | 6.78 | 136.48 | 0.00 | 0.03 |
| 8/20 | 6.71 | 136.07 | 6.73 | 136.36 | 0.02 | 0.29 |
| 9/21 | 6.13 | 132.78[d] | 6.12 | 132.05 | 0.01 | [d] |
| 10/22 | 5.7 | 132.12[d] | 5.72 | 131.97 | 0.02 | [d] |
| 27/35 | 5.7 | 132.05[d]<br>131.95[d]<br>131.88[d]<br>131.63[d] | 5.72 | 131.97 | 0.02 | [d]<br>[d]<br>[d]<br>[d] |
| 28/36 | 5.7 | 129.62 | 5.72 | 129.43 | 0.02 | 0.19 |
| 28/36 | 5.7 | 129.96 | 5.72 | 129.43 | 0.02 | 0.53 |
| 29/37 | 1.75 | 18.18 | 1.74 | 18.24 | 0.01 | 0.06 |
| 29/37 | 1.74 | 18.15 | 1.74 | 18.24 | 0.00 | 0.09 |
| 11a | 2.72 | 30.76 | 2.75 | 31.04 | 0.03 | 0.28 |
| 11b | 2.62 | - | 2.60 | - | 0.02 | - |
| 12 | 5.45<br>- | 77.41<br>56.29 | 5.40<br>- | 77.53<br>56.09 | 0.05<br>- | 0.12<br>0.20 |
| 26 | 4.37 | 76.42 | 4.38 | 76.69 | 0.01 | 0.27 |
| 30 | 1.35 | 14.06 | 1.29 | 15.21 | 0.06 | 1.15 |
| 31 | -<br>- | 176.78<br>161.03 | -<br>- | 178.19<br>161.71 | -<br>- | 1.41<br>0.68 |
| 15 | 8.47 | 145.58 | 8.57 | 145.74 | 0.10 | 0.16 |
| 23a | 2.88 | 30.4 | 2.87 | 30.37 | 0.01 | 0.03 |
| 23b | 2.53 | - | 2.53 | - | 0.00 | - |
| 24 | 5.28<br>- | 77.52<br>46.91 | 5.28 | 77.43<br>46.89 | 0.00 | 0.09<br>0.02 |
| 34 | 4.13 | 76.9 | 4.13 | 76.77 | 0.00 | 0.13 |
| 38 | 1.02 | 15.53 | 1.01 | 15.47 | 0.01 | 0.06 |
| 39 | 3.61<br>- | 66.34<br>161.7 | 3.61 | 66.21<br>161.71 | 0.00 | 0.14<br>0.01 |
| 3 | 8.53 | 145.58 | 8.57 | 145.74 | 0.04 | 0.16 |

[a] $^1H/^{13}C$ at 700/175 MHz; [b] isolated from *S. cellulosum* So ce1875; [c] isolated from *M. xanthus* DK1622::km-int-Ptet-dis427-gent-delF; [d] overlapping signals.

The elemental formula $C_{38}H_{44}N_2O_{10}$ of disorazole Z10 (39-*O*-desmethyl-25,25-dimethyl-disorazole Z) (**12**) with a retention time of 10.59 min in analytic HPLC showed the formal loss of $C_2H_2O_2$. In the NMR spectra both methoxy signals were absent. Similar to variants **10** and **11** only one carboxyl group C-39 at 176.75 ppm remained as free carboxylic acid on one side chain. Its position was retrieved from HMBC correlations with C-38, -34, and -24. The second side chain featured two geminal methyl groups C-30 and C-31 similar to disorazole A1 (**1**).(5) These were identified from their mutual HMBC correlations and positioned from their HMBC correlations with C-12, C-25 and C-26 together with their NOE interactions.

**Table S13** NMR data of disorazole Z10 (**12**) in methanol-*d*$_4$.[a]

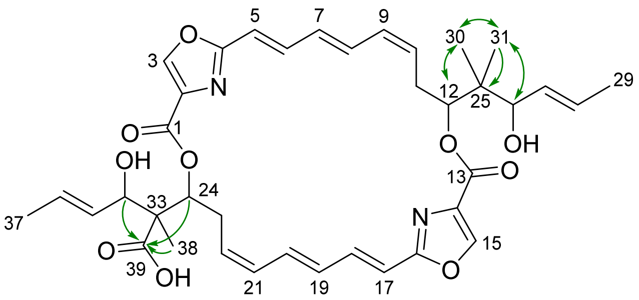


| Pos. | $\delta_H$ | m (J [Hz]) | COSY | ROESY | $\delta_C$ | type | H to C HMBC |
|---|---|---|---|---|---|---|---|
| 1 | - | - | - | - | 160.99 | C | 24 |
| 2 | - | - | - | - | 135.95 | C | 3 |
| 3 | 8.47 | s | - | - | 145.60 | CH | |
| 4/16 | - | - | - | - | 163.59<br>163.54 | C<br>C | 3/15, 5/17<br>3/15, 5/17 |
| 5/17 | 6.13c | m | 6/18 | - | 116.05<br>116.22 | CH<br>CH | 7/19<br>7/19 |
| 6/18 | 6.83 | m | 5/17 | - | 139.27<br>139.15 | CH | 7/19, 8/20 |
| 7/19 | 6.13[c] | m | - | - | 133.28[b]<br>132.88[b] | CH<br>CH | |
| 8/20 | 6.72 | dd (15.5, 12.3) | 9/21 | 11a/23a | 136.26<br>136.07 | CH<br>CH | 7/19, 9/21, 10/22<br>7/19, 9/21, 10/22 |
| 9/21 | 6.13[c] | m | 8/20, 10/22 | - | 132.63[b]<br>132.13[b] | CH<br>CH | |
| 10 | 5.69[d] | m | 9/21, 23ab | - | 131.62[b] | CH | 12, 26 |
| 11a | 2.71 | m | 10, 12 | 8/20, 11b, 30, 31 | 29.81 | CH$_2$ | 9, 10, 12 |
| 11b | 2.43 | dd (13.9, 7.3) | 10, 12 | 11a, 26, 30, 31 | | | |
| 12 | 5.13 | d (10.3) | 11ab | 11b, 26, 27/28, 30, 31 | 79.15 | CH | 11a/b, 26, 30, 31 |
| 13 | - | - | - | - | 161.75 | C | 12 |
| 14 | - | - | - | - | 136.07 | C | 15 |
| 15 | 8.56 | s | - | - | 145.53 | CH | |
| 22 | | | 9/21, 23ab | - | 131.93[b] | CH | 24, 34 |
| 23a | 2.72 | m | 22, 24 | 8/20, 23b, 38 | 30.76 | CH$_2$ | 21, 22, 24 |
| 23b | 2.62 | dd (13.6, 7.3) | 22, 24 | 22, 23a, 24, 34, 38 | | | |
| 24 | 5.45 | d (9.9) | 23ab | 23b, 34, 38 | 77.41 | CH | 34, 23a/b, 38 |
| 25 | - | - | - | - | 42.94 | C | 12, 26, 30, 31 |
| 26 | 3.89 | d (7.7) | 29 | 15, 11b, 12, 27/28,<br>30, 31 | 77.99 | CH | 12, 27, 28, 30, 31 |
| 27 | 5.59 | ddq (15.4, 8.1,<br>1.5) | 28, 29 | 11b, 12, 26, 29/37,<br>30, 31 | 131.93[b] | CH | |
| 28 | 5.69[d] | m | 27, 29 | - | 129.78 | CH | |
| 29/37 | 1.72 | dd (6.6, 1.5) | 26/34, 27/35, 28/36 | 27/35, 28/36 | 18.14<br>18.18 | CH$_3$<br>CH$_3$ | 27/35, 28/36<br>27/35, 28/36 |
| 30 | 0.98 | s | 31 | 11a, 12, 26, 36, 31 | 19.66 | CH$_3$ | 12, 26, 31 |
| 31 | 1.05 | s | 30 | 11a, 12, 26, 36, 30 | 19.44 | CH$_3$ | 12, 26, 30 |
| 33 | - | - | - | - | 56.30 | C | 23b, 24, 34, 38 |
| 34 | 4.37 | d (7.3) | 37 | 23b, 24, 3, 38 | 76.39 | CH | 24, 35, 36, 38 |
| 35 | 5.76 | m | 36, 37 | - | 129.96 | CH | |
| 36 | 5.69[d] | m | 37 | - | 131.62[b] | CH | |
| 38 | 1.35 | s | - | 23ab, 24, 34, 27/35 | 14.02 | CH$_3$ | 24, 34 |
| 39 | - | - | - | - | 176.75 | C | 24, 34, 38 |

[a] $^1$H/$^{13}$C 600/150 MHz; [b, c, d] interchangeable. For comparability reasons side chain numbering starts with position 25 and 33, respectively. Disorazole Z10 (**12**) misses CH$_3$-32 and CH$_3$-40.

Comparison of $^1H$ and $^{13}C$ NMR data of disorazol Z10 (**12**) isolated from *S. cellulosum* So ce1875 and *M. xanthus* DK1622::km-int-Ptet-dis427-gent-delF revealed a shift differences <0.2 ppm ($\Delta\delta_H^b$-$\delta_H^c$). Thus, both compounds are identical (Table S14 and Figure S61). Greater differences of carbon shifts can be explained due to their extraction from HMBC experiments.

**Table S14** Comparison of $^1H$ and $^{13}C$ NMR data of **12** in methanol-$d_4$ isolated from *S. cellulosum* and *M. xanthus*.[a]

| Pos. | $\delta H^b$ | $\delta C^b$ | $\delta H^c$ | $\delta C^{c,d}$ | $\Delta\delta H^b$-$\delta H^c$ | $\Delta\delta C^b$-$\delta C^{c,d}$ |
|---|---|---|---|---|---|---|
| 1 | - | 160.99 | - | 160.30 | - | 0.69 |
| 2 | - | 135.95 | - | 135.26 | - | 0.69 |
| 3 | 8.47 | 145.6 | 8.30 | 144.68 | 0.17 | 0.92 |
| 4/16 | - | 163.59 | - | 162.19 | - | 1.40 |
| 4/16 | - | 163.54 | - | 162.19 | - | 1.35 |
| 5/17 | 6.13 | 116.05 | 6.11 | 114.31 | 0.02 | 1.74 |
| 5/17 | - | 116.22 | 6.11 | 114.31 | - | 1.91 |
| 6/18 | 6.83 | 139.27 | 6.81 | 137.42 | 0.02 | 1.85 |
| 6/18 | - | 139.15 | 6.81 | 137.42 | - | 1.73 |
| 7/19 | 6.13 | 133.28 | 6.12 | 131.21 | 0.01 | 2.07 |
| 7/19 | - | 132.88 | 6.12 | 131.21 | - | 1.67 |
| 8/20 | 6.72 | 136.26 | 6.74 | 134.66 | 0.02 | 1.60 |
| 8/20 | - | 136.07 | 6.74 | 134.66 | - | 1.41 |
| 9/21 | 6.13 | 132.63 | 6.12 | 130.29 | 0.01 | 2.34 |
| 9/21 | - | 132.13 | 6.12 | 130.29 | - | 1.84 |
| 10 | 5.69 | 131.62 | 5.72 | 130.91 | 0.03 | 0.71 |
| 11a | 2.71 | 29.81 | 2.71 | 28.48 | 0.00 | 1.34 |
| 11b | 2.43 | 29.81 | 2.42 | 28.48 | 0.01 | 1.34 |
| 12 | 5.13 | 79.15 | 5.13 | 77.34 | 0.00 | 1.81 |
| 13 | - | 161.75 | - | 160.30 | - | 1.45 |
| 14 | - | 136.07 | - | 135.26 | - | 0.81 |
| 15 | 8.56 | 145.53 | 8.62 | 144.36 | 0.06 | 1.17 |
| 22 | - | 131.93 | 5.72 | 130.91 | - | 1.02 |
| 23a | 2.72 | 30.76 | 2.79 | 29.92 | 0.07 | 0.85 |
| 23b | 2.62 | 30.76 | 2.58 | 29.92 | 0.04 | 0.85 |
| 24 | 5.45 | 77.41 | 5.36 | 76.08 | 0.09 | 1.33 |
| 25 | - | 42.94 | - | 41.54 | - | 1.40 |
| 26 | 3.89 | 77.99 | 3.89 | 76.42 | 0.00 | 1.57 |
| 27 | 5.59 | 131.93 | 5.59 | 130.29 | 0.00 | 1.64 |
| 28 | 5.69 | 129.78 | 5.73 | 127.14 | 0.04 | 2.64 |
| 29/37 | - | 18.14 | 1.73 | 16.89 | - | 1.25 |
| 29/37 | 1.72 | 18.18 | 1.73 | 16.89 | 0.01 | 1.29 |
| 30 | 0.98 | 19.66 | 1.05 | 18.14 | 0.06 | 1.52 |
| 31 | 1.05 | 19.44 | 0.98 | 18.30 | 0.08 | 1.14 |
| 33 | - | 56.3 | - | 54.46 | - | 1.84 |
| 34 | 4.37 | 76.39 | 4.39 | 75.46 | 0.02 | 0.93 |
| 35 | 5.76 | 129.96 | 5.69 | 128.08 | 0.07 | 1.88 |
| 36 | 5.69 | 131.62 | 5.59 | 130.29 | 0.10 | 1.33 |
| 38 | 1.35 | 14.02 | 1.24 | 14.99 | 0.11 | 0.97 |
| 39 | - | 176.75 | - | 178.23 | - | 1.48 |

[a] $^1H/^{13}C$ at 700/175 MHz; [b] isolated from *S. cellulosum* So ce1875; [c] isolated from *M. xanthus* DK1622::km-int-Ptet-dis427-gent-delF; [d] $\delta_C$ extracted from HMBC.

## 2. Supplemental tables

**Table S15** DNA oligos used in this study.

| Name | Sequence (5’ to 3’) | Application |
|---|---|---|
| p15A-cm-MluCI-dis427-F | CCTGGCCGAGGATCGCGCCCACCGCCTGATCAGCGGCCCCCGCATGCTCCAGCAGCTCGCCAGGAGATCCCGCCAGCGGCAATTAGATCCGAAAACCCCAAGTTACG | for amplification of the p15A-cm-MluCI vector |
| p15A-cm-MluCI-dis427-R | AGTCAGGCTATGCCCTCGTTTTATAGACCACAAAATCGCGGTGTCAACGCGGTGCGCGAGAATACAAAGAGTCCGCGCAAAATTAGATCCTTTCTCCTCTTTAGATC | |
| pBR322-amp-BstXI-dis427-F | GCTGGGGAGCTCGGCCTTCCGGGAGGACTACGGCCTGCGCTACGCCTACGTCGCCGGATCCATGGTCGAGGGCATCTCGTCCAGCGAGCTGGAGATCCGAAAACCCCAAGTTACG | for amplification of the pBR322-amp-BstXI vector |
| pBR322-amp-BstXI-dis427-R | GGCGAGGTGAATGGCGGTGAGGGACGACGCGCACGCGGTGTCCACCGCCATGCAGGGCCCGTGCAGGTTGAGGAAATACGCCACATGATTGGGATCTAAAGAGGAGAAAGGATCT | |
| dis427-F3-F | CTGGGGAGCTCGGCCTTCCGGGAGGACTAC | for amplification of a BGC fragment |
| dis427-F3-R | GCTGTCCGCCGAGAGGTGGTTCCGTGTGGG | |
| p15A-cm-dis427-F | GAGCGGCTCGTTCGCCCTCGCGGAGCTCCGCGGCCGCGCCCCCGCGACGGACCGGTGGGTCCCACACGGAACCACCTCTCAGATCCGAAAACCCCAAGTTACG | for amplification of the p15A-cm vector |
| km-int-F | ACTAGTGCTTGGATTCTCACCAA | for amplification of the *km-int* cassette |
| km-int-R | CAACGCGGTGCGCGAGAATACAAAGAGTCCGCGCAAAATTAAGACGGACGAGGTGCTCACTCA | |
| Ptet-disA-R | ATGCCGATGATGGCAATGCCGTCCTGCTCTACGTGATTCCCGCTGCTCATAGATCCTTTCTCCTCTTTAGATC | for amplification of the *km-int-Ptet* cassette |
| Papr-disD-F | ATCACGCAGGTCGCAGGCACGAGCAGGGCTCTCTCATCTAGGAGGCGCTTACGCTCAGTGGAACGAGGTT | for amplification of the *Papr-disD* cassette |
| Papr-disD-R | CCTCCCATCCCTGGCGACTGCGACCCCTGCCCCGGAAACATGACGGCCTTCATAATCTGTACCTCCTTAAGTCAGCCAATCGACTGGCGAGC | |
| gent-delF-F | AGGCCTCGGCCGATCTCCTGGACCACCGCCTGCGCGCGCTGTCGCGGTAAGAAGGCACGAACCCAGTTGAC | for amplification of the *gent-delF* cassette |
| gent-delF-R | CTGAGCCTTTCGTTTTATTTGATGCCTGGAGATCCTTAAGATCCGTAACTTGGGGTTTTCGGATCTTTAGGTGGCGGTACTTGGGTC | |
| apr-Pvan-disA-F | AATTTTGCGCGGACTCTTTGTATTCTCGCGCACCGCGTTGACACCGCGATTCAGCCAATCGACTGGCGAGCGG | for amplification of the *apr-Pvan-disA* cassette |
| apr-Pvan-disA-R | GCCATGCCGATGATGGCAATGCCGTCCTGCTCTACGTGATTCCCGCTGCTCATATGCGTTTCCTCGCATCGTG | |
| dis427-chk01-F | GGATCGTGAGTACCTGGAGAAG | for colony PCR |
| dis427-chk01-R | GAGCGTCCGGGAGGTCGTGGGC | |
| dis427-chk02-F | GCAGAAGTACGTGGGCCTCAGC | |
| dis427-chk02-R | CGACGAGCAGGGTGGCGTATCC | |
| dis427-chk03-F | CGACCTCTACCTGAAGCACGATG | |
| dis427-chk03-R | CTGCACCTGATAATCGACCTGGC | |
| HisTEV-disF-F | TTACGATATCCCAACGACCGAAAACCTGTATTTTCAGGGCCTTGCAAAACTCATGTTGTCTCA | for cloning *disF*$_{427}$ into pHis-TEV |
| HisTEV-disF-R | CTTTGTTAGCAGCCGGATCTCAGTGGTGGTGGTGGTGGTGCTACGCGCTCCGCTGGGCGATGG | |

**Table S16** Half-inhibitory concentrations ($IC_{50}$, mean ± SD) of **3** in comparison of **1** on selected human cancer cell lines.

| Cell line | Origin | disorazole A1 (**1**) $IC_{50}$ [nM] | disorazole Z1 (**3**) $IC_{50}$ [nM] |
|---|---|---|---|
| A549 | lung carcinoma | 0.35 ± 0.15 | 0.21 ± 0.11 |
| HCT-116 | colon carcinoma | 0.24 ± 0.02 | 0.25 |
| HepG2 | hepatocellular carcinoma | 0.28 ± 0.07 | 0.06 |
| HL-60 | promyelocytic leukemia | 0.07 ± 0.01 | nd |
| KB-3.1 | cervix carcinoma | 0.15 ± 0.02 | 0.07 ± 0.02 |
| KB-V1 | cervix carcinoma, MDR | 0.17 ± 0.01 | 0.11 ± 0.02 |
| U-2 OS | osteosarcoma | 0.43 ± 0.16 | 0.09 |

nd: not determined; MDR: multi-drug resistant.

**Table S17** Crystal data and structure refinement for sh3137_a_sq (**3**).

| | | |
|---|---|---|
| Identification code | sh3137_a_sq | |
| Empirical formula | $C_{44}H_{58}N_2O_{14}$ | |
| Formula weight | 838.92 | |
| Temperature | 133(2) K | |
| Wavelength | 0.71073 Å | |
| Crystal system | Orthorhombic | |
| Space group | $P2_12_12_1$ | |
| Unit cell dimensions | a = 8.5672(3) Å | α= 90° |
| | b = 20.1022(6) Å | β = 90° |
| | c = 25.8711(9) Å | γ = 90° |
| Volume | 4455.5(3) $Å^3$ | |
| Z | 4 | |
| Density (calculated) | 1.251 $mg/m^3$ | |
| Absorption coefficient | 0.093 $mm^{-1}$ | |
| F(000) | 1792 | |
| Crystal size | 0.436 x 0.241 x 0.140 $mm^3$ | |
| Theta range for data collection | 1.283 to 27.134° | |
| Index ranges | -10<=h<=10, -21<=k<=25, -33<=l<=32 | |
| Reflections collected | 30846 | |
| Independent reflections | 9363 [R(int) = 0.0376] | |
| Completeness to theta = 25.242° | 94.3% | |
| Absorption correction | Semi-empirical from equivalents | |
| Max. and min. transmission | 0.7455 and 0.6616 | |
| Refinement method | Full-matrix least-squares on $F^2$ | |
| Data / restraints / parameters | 9363 / 2 / 495 | |
| Goodness-of-fit on $F^2$ | 1.053 | |
| Final R indices [I>2sigma(I)] | R1 = 0.0586, wR2 = 0.1356 | |
| R indices (all data) | R1 = 0.0921, wR2 = 0.1606 | |
| Absolute structure parameter | -0.2(3) | |
| Extinction coefficient | n/a | |
| Largest diff. peak and hole | 0.316 and -0.269 e.$Å^{-3}$ | |

**Table S18** Atomic coordinates (x $10^4$) and equivalent isotropic displacement parameters ($Å^2$ x $10^3$) for sh3137_a_sq (**3**). U(eq) is defined as one third of the trace of the orthogonalized $U^{ij}$ tensor.

| | x | y | z | U(eq) |
|---|---|---|---|---|
| N(1) | 5726(5) | 4725(2) | 2853(1) | 54(1) |
| N(2) | 4427(4) | 4759(2) | 807(1) | 39(1) |
| O(1) | 2985(3) | 3983(2) | 3736(1) | 37(1) |
| O(2) | 3959(4) | 5035(2) | 3755(1) | 43(1) |
| O(3) | 6092(4) | 3700(3) | 2539(1) | 69(1) |
| O(4) | 9420(4) | 2849(2) | -1292(1) | 55(1) |
| O(5) | 4487(4) | 2578(2) | -695(1) | 44(1) |
| O(6) | 4898(3) | 3611(2) | -975(1) | 41(1) |
| O(7) | 6711(3) | 3660(2) | 8(1) | 34(1) |
| O(8) | 6178(4) | 4751(2) | -132(1) | 42(1) |
| O(9) | 4243(3) | 3913(2) | 1360(1) | 36(1) |
| O(10) | 165(4) | 3948(2) | 5131(1) | 53(1) |
| O(11) | 4675(4) | 2947(2) | 4444(2) | 59(1) |
| O(12) | 4803(3) | 4002(2) | 4712(1) | 43(1) |
| C(1) | 3910(4) | 4474(3) | 3575(2) | 37(1) |
| C(2) | 4872(5) | 4262(3) | 3139(2) | 46(1) |
| C(3) | 5084(6) | 3652(3) | 2948(2) | 57(1) |
| C(4) | 6427(5) | 4381(4) | 2504(2) | 60(2) |
| C(5) | 7383(6) | 4606(4) | 2094(2) | 79(2) |
| C(6) | 7976(5) | 4231(4) | 1704(2) | 75(2) |
| C(7) | 8863(6) | 4502(4) | 1283(2) | 73(2) |
| C(8) | 9251(5) | 4168(3) | 852(2) | 53(1) |
| C(9) | 9922(5) | 4492(3) | 403(2) | 54(1) |
| C(10) | 9968(5) | 4256(3) | -74(2) | 46(1) |
| C(11) | 9395(4) | 3587(3) | -254(2) | 42(1) |
| C(12) | 7713(4) | 3617(2) | -450(1) | 33(1) |
| C(13) | 6085(4) | 4251(2) | 126(1) | 35(1) |
| C(14) | 5233(4) | 4216(2) | 617(1) | 32(1) |
| C(15) | 5130(4) | 3702(2) | 952(1) | 33(1) |
| C(16) | 3856(4) | 4559(2) | 1244(2) | 36(1) |
| C(17) | 2913(5) | 4912(3) | 1611(2) | 43(1) |
| C(18) | 2360(5) | 4633(3) | 2042(2) | 43(1) |
| C(19) | 1401(5) | 4956(3) | 2420(2) | 43(1) |
| C(20) | 905(5) | 4661(3) | 2853(2) | 41(1) |
| C(21) | 28(5) | 4982(3) | 3254(2) | 45(1) |
| C(22) | -248(5) | 4745(3) | 3725(2) | 46(1) |
| C(23) | 265(5) | 4089(2) | 3942(2) | 42(1) |
| C(24) | 1917(5) | 4104(2) | 4169(2) | 34(1) |
| C(25) | 7149(4) | 2997(2) | -743(1) | 33(1) |
| C(26) | 7789(5) | 2997(3) | -1308(2) | 43(1) |
| C(27) | 6950(6) | 2524(3) | -1657(2) | 48(1) |
| C(28) | 6173(8) | 2692(3) | -2074(2) | 63(2) |
| C(29) | 5375(10) | 2234(4) | -2434(2) | 91(2) |
| C(30) | 7566(5) | 2348(2) | -456(2) | 39(1) |
| C(31) | 5371(5) | 3016(2) | -795(1) | 36(1) |
| C(32) | 3225(6) | 3667(3) | -1039(3) | 65(2) |
| C(33) | 2276(5) | 3555(2) | 4582(2) | 39(1) |
| C(34) | 1811(5) | 3839(3) | 5125(2) | 45(1) |
| C(35) | 2252(6) | 3376(3) | 5557(2) | 55(1) |
| C(36) | 3303(6) | 3493(3) | 5902(2) | 56(1) |
| C(37) | 3673(9) | 3048(4) | 6359(2) | 78(2) |
| C(38) | 1468(6) | 2906(3) | 4469(2) | 54(1) |
| C(39) | 4029(5) | 3455(3) | 4572(2) | 42(1) |
| C(40) | 6478(6) | 3966(3) | 4686(2) | 59(1) |

**Table S19** Bond lengths [Å] and angles [°] for sh3137_a_sq (**3**).

| | |
|---|---|
| N(1)-C(4) | 1.284(7) |
| N(1)-C(2) | 1.396(6) |
| N(2)-C(16) | 1.297(5) |
| N(2)-C(14) | 1.382(6) |
| O(1)-C(1) | 1.333(5) |
| O(1)-C(24) | 1.467(5) |
| O(2)-C(1) | 1.221(6) |
| O(3)-C(3) | 1.369(6) |
| O(3)-C(4) | 1.403(8) |
| O(4)-C(26) | 1.428(5) |
| O(4)-H(14) | 0.8400 |
| O(5)-C(31) | 1.190(5) |
| O(6)-C(31) | 1.346(5) |
| O(6)-C(32) | 1.447(5) |
| O(7)-C(13) | 1.340(5) |
| O(7)-C(12) | 1.464(4) |
| O(8)-C(13) | 1.208(5) |
| O(9)-C(15) | 1.368(5) |
| O(9)-C(16) | 1.375(5) |
| O(10)-C(34) | 1.427(6) |
| O(10)-H(29) | 0.8400 |
| O(11)-C(39) | 1.207(6) |
| O(12)-C(39) | 1.334(6) |
| O(12)-C(40) | 1.438(6) |
| C(1)-C(2) | 1.459(6) |
| C(2)-C(3) | 1.335(8) |
| C(3)-H(3) | 0.9500 |
| C(4)-C(5) | 1.415(7) |
| C(5)-C(6) | 1.357(9) |
| C(5)-H(5) | 0.9500 |
| C(6)-C(7) | 1.435(7) |
| C(6)-H(6) | 0.9500 |
| C(7)-C(8) | 1.345(8) |
| C(7)-H(7) | 0.9500 |
| C(8)-C(9) | 1.452(6) |
| C(8)-H(8) | 0.9500 |
| C(9)-C(10) | 1.320(7) |
| C(9)-H(9) | 0.9500 |
| C(10)-C(11) | 1.506(7) |
| C(10)-H(10) | 0.9500 |
| C(11)-C(12) | 1.528(5) |
| C(11)-H(11A) | 0.9900 |
| C(11)-H(11B) | 0.9900 |
| C(12)-C(25) | 1.537(6) |
| C(12)-H(12) | 1.0000 |
| C(13)-C(14) | 1.467(5) |
| C(14)-C(15) | 1.351(6) |
| C(15)-H(15) | 0.9500 |
| C(16)-C(17) | 1.433(6) |
| C(17)-C(18) | 1.334(6) |
| C(17)-H(17) | 0.9500 |
| C(18)-C(19) | 1.434(6) |
| C(18)-H(18) | 0.9500 |
| C(19)-C(20) | 1.336(6) |
| C(19)-H(19) | 0.9500 |
| C(20)-C(21) | 1.435(6) |
| C(20)-H(20) | 0.9500 |
| C(21)-C(22) | 1.328(6) |
| C(21)-H(21) | 0.9500 |
| C(22)-C(23) | 1.500(7) |
| C(22)-H(22) | 0.9500 |
| C(23)-C(24) | 1.532(6) |
| C(23)-H(23A) | 0.9900 |
| C(23)-H(23B) | 0.9900 |
| C(24)-C(33) | 1.566(6) |
| C(24)-H(24) | 1.0000 |
| C(25)-C(31) | 1.530(5) |
| C(25)-C(30) | 1.544(6) |
| C(25)-C(26) | 1.563(5) |
| C(26)-C(27) | 1.494(7) |
| C(26)-H(26) | 1.0000 |
| C(27)-C(28) | 1.312(6) |
| C(27)-H(27) | 0.9500 |
| C(28)-C(29) | 1.478(9) |
| C(28)-H(28) | 0.9500 |
| C(29)-H(29A) | 0.9800 |
| C(29)-H(29B) | 0.9800 |
| C(29)-H(29C) | 0.9800 |
| C(30)-H(30A) | 0.9800 |
| C(30)-H(30B) | 0.9800 |
| C(30)-H(30C) | 0.9800 |
| C(32)-H(32A) | 0.9800 |
| C(32)-H(32B) | 0.9800 |
| C(32)-H(32C) | 0.9800 |
| C(33)-C(38) | 1.507(7) |
| C(33)-C(39) | 1.516(6) |
| C(33)-C(34) | 1.567(7) |
| C(34)-C(35) | 1.502(7) |
| C(34)-H(34) | 1.0000 |
| C(35)-C(36) | 1.289(8) |
| C(35)-H(35) | 0.9500 |
| C(36)-C(37) | 1.516(7) |
| C(36)-H(36) | 0.9500 |
| C(37)-H(37A) | 0.9800 |
| C(37)-H(37B) | 0.9800 |
| C(37)-H(37C) | 0.9800 |
| C(38)-H(38A) | 0.9800 |
| C(38)-H(38B) | 0.9800 |
| C(38)-H(38C) | 0.9800 |
| C(40)-H(40A) | 0.9800 |
| C(40)-H(40B) | 0.9800 |
| C(40)-H(40C) | 0.9800 |

| | |
|---|---|
| C(4)-N(1)-C(2) | 105.1(5) |
| C(16)-N(2)-C(14) | 104.6(4) |
| C(1)-O(1)-C(24) | 119.1(3) |
| C(3)-O(3)-C(4) | 104.3(4) |
| C(26)-O(4)-H(14) | 109.5 |
| C(31)-O(6)-C(32) | 114.0(4) |
| C(13)-O(7)-C(12) | 118.1(3) |
| C(15)-O(9)-C(16) | 105.0(3) |
| C(34)-O(10)-H(29) | 109.5 |
| C(39)-O(12)-C(40) | 116.3(4) |
| O(2)-C(1)-O(1) | 125.8(4) |
| O(2)-C(1)-C(2) | 123.1(4) |
| O(1)-C(1)-C(2) | 111.1(4) |
| C(3)-C(2)-N(1) | 110.1(4) |
| C(3)-C(2)-C(1) | 129.1(5) |
| N(1)-C(2)-C(1) | 120.8(5) |
| C(2)-C(3)-O(3) | 107.9(5) |
| C(2)-C(3)-H(3) | 126.1 |
| O(3)-C(3)-H(3) | 126.1 |
| N(1)-C(4)-O(3) | 112.6(4) |
| N(1)-C(4)-C(5) | 128.8(7) |
| O(3)-C(4)-C(5) | 118.5(6) |
| C(6)-C(5)-C(4) | 126.7(7) |
| C(6)-C(5)-H(5) | 116.7 |
| C(4)-C(5)-H(5) | 116.7 |
| C(5)-C(6)-C(7) | 123.5(7) |
| C(5)-C(6)-H(6) | 118.3 |
| C(7)-C(6)-H(6) | 118.3 |
| C(8)-C(7)-C(6) | 124.8(6) |
| C(8)-C(7)-H(7) | 117.6 |
| C(6)-C(7)-H(7) | 117.6 |
| C(7)-C(8)-C(9) | 122.6(6) |
| C(7)-C(8)-H(8) | 118.7 |
| C(9)-C(8)-H(8) | 118.7 |
| C(10)-C(9)-C(8) | 126.7(5) |
| C(10)-C(9)-H(9) | 116.6 |
| C(8)-C(9)-H(9) | 116.6 |
| C(9)-C(10)-C(11) | 126.9(4) |
| C(9)-C(10)-H(10) | 116.5 |
| C(11)-C(10)-H(10) | 116.5 |
| C(10)-C(11)-C(12) | 112.0(4) |
| C(10)-C(11)-H(11A) | 109.2 |
| C(12)-C(11)-H(11A) | 109.2 |

| | |
|---|---|
| C(10)-C(11)-H(11B) | 109.2 |
| C(12)-C(11)-H(11B) | 109.2 |
| H(11A)-C(11)-H(11B) | 107.9 |
| O(7)-C(12)-C(11) | 106.7(3) |
| O(7)-C(12)-C(25) | 105.2(3) |
| C(11)-C(12)-C(25) | 115.3(4) |
| O(7)-C(12)-H(12) | 109.8 |
| C(11)-C(12)-H(12) | 109.8 |
| C(25)-C(12)-H(12) | 109.8 |
| O(8)-C(13)-O(7) | 125.8(4) |
| O(8)-C(13)-C(14) | 123.5(4) |
| O(7)-C(13)-C(14) | 110.8(4) |
| C(15)-C(14)-N(2) | 110.1(3) |
| C(15)-C(14)-C(13) | 128.7(4) |
| N(2)-C(14)-C(13) | 121.2(4) |
| C(14)-C(15)-O(9) | 107.2(4) |
| C(14)-C(15)-H(15) | 126.4 |
| O(9)-C(15)-H(15) | 126.4 |
| N(2)-C(16)-O(9) | 113.1(4) |
| N(2)-C(16)-C(17) | 129.6(4) |
| O(9)-C(16)-C(17) | 117.3(4) |
| C(18)-C(17)-C(16) | 123.0(5) |
| C(18)-C(17)-H(17) | 118.5 |
| C(16)-C(17)-H(17) | 118.5 |
| C(17)-C(18)-C(19) | 125.7(5) |
| C(17)-C(18)-H(18) | 117.1 |
| C(19)-C(18)-H(18) | 117.1 |
| C(20)-C(19)-C(18) | 123.6(5) |
| C(20)-C(19)-H(19) | 118.2 |
| C(18)-C(19)-H(19) | 118.2 |
| C(19)-C(20)-C(21) | 125.0(5) |
| C(19)-C(20)-H(20) | 117.5 |
| C(21)-C(20)-H(20) | 117.5 |
| C(22)-C(21)-C(20) | 126.6(5) |
| C(22)-C(21)-H(21) | 116.7 |
| C(20)-C(21)-H(21) | 116.7 |
| C(21)-C(22)-C(23) | 127.3(4) |
| C(21)-C(22)-H(22) | 116.4 |
| C(23)-C(22)-H(22) | 116.4 |
| C(22)-C(23)-C(24) | 113.4(4) |
| C(22)-C(23)-H(23A) | 108.9 |
| C(24)-C(23)-H(23A) | 108.9 |
| C(22)-C(23)-H(23B) | 108.9 |
| C(24)-C(23)-H(23B) | 108.9 |
| H(23A)-C(23)-H(23B) | 107.7 |
| O(1)-C(24)-C(23) | 106.3(3) |
| O(1)-C(24)-C(33) | 106.4(3) |
| C(23)-C(24)-C(33) | 115.4(4) |
| O(1)-C(24)-H(24) | 109.5 |
| C(23)-C(24)-H(24) | 109.5 |
| C(33)-C(24)-H(24) | 109.5 |
| C(31)-C(25)-C(12) | 109.7(3) |
| C(31)-C(25)-C(30) | 107.0(3) |
| C(12)-C(25)-C(30) | 112.1(3) |
| C(31)-C(25)-C(26) | 105.5(3) |
| C(12)-C(25)-C(26) | 110.6(3) |
| C(30)-C(25)-C(26) | 111.6(4) |
| O(4)-C(26)-C(27) | 110.9(4) |
| O(4)-C(26)-C(25) | 108.4(3) |
| C(27)-C(26)-C(25) | 113.3(4) |
| O(4)-C(26)-H(26) | 108.0 |
| C(27)-C(26)-H(26) | 108.0 |
| C(25)-C(26)-H(26) | 108.0 |
| C(28)-C(27)-C(26) | 125.2(5) |
| C(28)-C(27)-H(27) | 117.4 |
| C(26)-C(27)-H(27) | 117.4 |
| C(27)-C(28)-C(29) | 126.4(6) |
| C(27)-C(28)-H(28) | 116.8 |
| C(29)-C(28)-H(28) | 116.8 |
| C(28)-C(29)-H(29A) | 109.5 |
| C(28)-C(29)-H(29B) | 109.5 |
| H(29A)-C(29)-H(29B) | 109.5 |
| C(28)-C(29)-H(29C) | 109.5 |
| H(29A)-C(29)-H(29C) | 109.5 |
| H(29B)-C(29)-H(29C) | 109.5 |
| C(25)-C(30)-H(30A) | 109.5 |
| C(25)-C(30)-H(30B) | 109.5 |
| H(30A)-C(30)-H(30B) | 109.5 |
| C(25)-C(30)-H(30C) | 109.5 |
| H(30A)-C(30)-H(30C) | 109.5 |
| H(30B)-C(30)-H(30C) | 109.5 |
| O(5)-C(31)-O(6) | 122.8(4) |
| O(5)-C(31)-C(25) | 126.7(4) |
| O(6)-C(31)-C(25) | 110.6(4) |
| O(6)-C(32)-H(32A) | 109.5 |
| O(6)-C(32)-H(32B) | 109.5 |
| H(32A)-C(32)-H(32B) | 109.5 |
| O(6)-C(32)-H(32C) | 109.5 |
| H(32A)-C(32)-H(32C) | 109.5 |
| H(32B)-C(32)-H(32C) | 109.5 |
| C(38)-C(33)-C(39) | 109.6(4) |
| C(38)-C(33)-C(24) | 112.7(4) |
| C(39)-C(33)-C(24) | 106.1(3) |
| C(38)-C(33)-C(34) | 111.9(4) |
| C(39)-C(33)-C(34) | 108.4(4) |
| C(24)-C(33)-C(34) | 107.8(4) |
| O(10)-C(34)-C(35) | 109.6(4) |
| O(10)-C(34)-C(33) | 108.5(4) |
| C(35)-C(34)-C(33) | 112.2(4) |
| O(10)-C(34)-H(34) | 108.8 |
| C(35)-C(34)-H(34) | 108.8 |
| C(33)-C(34)-H(34) | 108.8 |
| C(36)-C(35)-C(34) | 125.3(5) |
| C(36)-C(35)-H(35) | 117.4 |
| C(34)-C(35)-H(35) | 117.4 |
| C(35)-C(36)-C(37) | 125.3(6) |
| C(35)-C(36)-H(36) | 117.3 |
| C(37)-C(36)-H(36) | 117.3 |
| C(36)-C(37)-H(37A) | 109.5 |
| C(36)-C(37)-H(37B) | 109.5 |
| H(37A)-C(37)-H(37B) | 109.5 |
| C(36)-C(37)-H(37C) | 109.5 |
| H(37A)-C(37)-H(37C) | 109.5 |
| H(37B)-C(37)-H(37C) | 109.5 |
| C(33)-C(38)-H(38A) | 109.5 |
| C(33)-C(38)-H(38B) | 109.5 |
| H(38A)-C(38)-H(38B) | 109.5 |
| C(33)-C(38)-H(38C) | 109.5 |
| H(38A)-C(38)-H(38C) | 109.5 |
| H(38B)-C(38)-H(38C) | 109.5 |
| O(11)-C(39)-O(12) | 122.9(4) |
| O(11)-C(39)-C(33) | 124.9(4) |
| O(12)-C(39)-C(33) | 112.2(4) |
| O(12)-C(40)-H(40A) | 109.5 |
| O(12)-C(40)-H(40B) | 109.5 |
| H(40A)-C(40)-H(40B) | 109.5 |
| O(12)-C(40)-H(40C) | 109.5 |
| H(40A)-C(40)-H(40C) | 109.5 |
| H(40B)-C(40)-H(40C) | 109.5 |

**Table S20** Anisotropic displacement parameters ($Å^2 \times 10^3$) for sh3137_a_sq (**3**). The anisotropic displacement factor exponent takes the form: $-2\pi^2[ h^2 a^{*2}U^{11} + ... + 2 h k a^* b^* U^{12} ]$.

| | $U^{11}$ | $U^{22}$ | $U^{33}$ | $U^{23}$ | $U^{13}$ | $U^{12}$ |
|---|---|---|---|---|---|---|
| N(1) | 36(2) | 91(3) | 35(2) | -3(2) | -6(2) | -21(2) |
| N(2) | 34(2) | 51(2) | 32(2) | 2(2) | -3(1) | 4(2) |
| O(1) | 26(1) | 42(2) | 44(2) | -8(1) | 4(1) | -2(1) |
| O(2) | 34(2) | 49(2) | 47(2) | 1(2) | -4(1) | -5(2) |
| O(3) | 33(2) | 118(4) | 55(2) | -14(2) | 12(2) | 0(2) |
| O(4) | 37(2) | 86(3) | 43(2) | 22(2) | 19(1) | 18(2) |
| O(5) | 35(2) | 51(2) | 45(2) | 17(2) | 6(1) | 4(2) |
| O(6) | 24(1) | 47(2) | 51(2) | 14(2) | 0(1) | 7(1) |
| O(7) | 25(1) | 47(2) | 29(1) | 12(1) | 6(1) | 7(1) |
| O(8) | 43(2) | 49(2) | 35(2) | 11(1) | 4(1) | 0(2) |
| O(9) | 26(1) | 55(2) | 27(1) | 3(1) | 1(1) | -2(1) |
| O(10) | 45(2) | 59(2) | 54(2) | 4(2) | 6(2) | 1(2) |
| O(11) | 38(2) | 45(2) | 94(3) | -4(2) | -5(2) | 5(2) |
| O(12) | 30(2) | 43(2) | 55(2) | 6(2) | -5(1) | -9(1) |
| C(1) | 18(2) | 55(3) | 37(2) | 1(2) | -7(2) | -2(2) |
| C(2) | 25(2) | 71(4) | 41(2) | 0(2) | -7(2) | -2(2) |
| C(3) | 35(2) | 81(4) | 53(3) | -9(3) | 11(2) | 5(3) |
| C(4) | 29(2) | 111(5) | 41(2) | -7(3) | -7(2) | -25(3) |
| C(5) | 40(3) | 155(7) | 43(3) | -12(3) | -2(2) | -41(4) |
| C(6) | 27(2) | 159(7) | 39(2) | 1(3) | -5(2) | -30(3) |
| C(7) | 39(3) | 134(6) | 46(3) | -5(3) | 0(2) | -43(3) |
| C(8) | 26(2) | 88(4) | 45(2) | 6(3) | -5(2) | -21(2) |
| C(9) | 30(2) | 78(4) | 55(3) | 10(3) | 1(2) | -22(3) |
| C(10) | 28(2) | 67(3) | 45(2) | 19(2) | 8(2) | -10(2) |
| C(11) | 21(2) | 66(3) | 39(2) | 22(2) | 7(2) | 2(2) |
| C(12) | 24(2) | 44(3) | 32(2) | 16(2) | 5(2) | 5(2) |
| C(13) | 22(2) | 54(3) | 28(2) | 10(2) | -2(1) | -2(2) |
| C(14) | 19(2) | 52(3) | 27(2) | 4(2) | -5(1) | -1(2) |
| C(15) | 21(2) | 51(3) | 28(2) | 2(2) | -1(1) | -2(2) |
| C(16) | 23(2) | 56(3) | 30(2) | 2(2) | -6(2) | 1(2) |
| C(17) | 37(2) | 54(3) | 38(2) | -5(2) | -4(2) | 6(2) |
| C(18) | 26(2) | 69(3) | 36(2) | -9(2) | -6(2) | 3(2) |
| C(19) | 31(2) | 61(3) | 36(2) | -8(2) | -7(2) | 11(2) |
| C(20) | 26(2) | 61(3) | 37(2) | -17(2) | -10(2) | 6(2) |
| C(21) | 35(2) | 70(3) | 32(2) | -14(2) | -9(2) | 18(2) |
| C(22) | 35(2) | 67(3) | 36(2) | -16(2) | -3(2) | 18(2) |
| C(23) | 29(2) | 53(3) | 44(2) | -11(2) | 2(2) | 0(2) |
| C(24) | 28(2) | 34(2) | 39(2) | -4(2) | -2(2) | 0(2) |
| C(25) | 27(2) | 44(3) | 27(2) | 15(2) | 7(1) | 7(2) |
| C(26) | 37(2) | 64(3) | 27(2) | 16(2) | 10(2) | 14(2) |
| C(27) | 52(3) | 61(3) | 30(2) | 14(2) | 9(2) | 26(3) |
| C(28) | 88(4) | 60(4) | 41(2) | 6(2) | -8(3) | 30(3) |
| C(29) | 129(7) | 93(5) | 53(3) | -9(3) | -28(4) | 31(5) |
| C(30) | 33(2) | 47(3) | 36(2) | 16(2) | 5(2) | 10(2) |
| C(31) | 29(2) | 50(3) | 27(2) | 15(2) | 5(2) | 8(2) |
| C(32) | 28(2) | 59(4) | 107(4) | 19(3) | -9(3) | 10(2) |
| C(33) | 35(2) | 34(2) | 47(2) | 6(2) | -3(2) | -7(2) |
| C(34) | 39(2) | 45(3) | 51(3) | 7(2) | 4(2) | -2(2) |
| C(35) | 51(3) | 61(4) | 53(3) | 15(3) | 6(2) | -7(3) |
| C(36) | 51(3) | 69(4) | 49(3) | 4(3) | 7(2) | 14(3) |
| C(37) | 88(5) | 101(5) | 44(3) | 11(3) | 6(3) | 29(4) |
| C(38) | 42(3) | 45(3) | 76(3) | -3(3) | 0(2) | -16(2) |
| C(39) | 33(2) | 46(3) | 48(2) | 8(2) | -4(2) | -7(2) |
| C(40) | 34(2) | 53(3) | 89(4) | 9(3) | -11(2) | -12(2) |

**Table S21** Hydrogen coordinates (x $10^4$) and isotropic displacement parameters (Å$^2$ x $10^3$) for sh3137_a_sq (**3**).

| | x | y | z | U(eq) |
|---|---|---|---|---|
| H(14) | 9897 | 3099 | -1499 | 82 |
| H(29) | -38 | 4281 | 5315 | 79 |
| H(3) | 4618 | 3255 | 3074 | 68 |
| H(5) | 7635 | 5066 | 2091 | 95 |
| H(6) | 7794 | 3765 | 1710 | 90 |
| H(7) | 9201 | 4951 | 1312 | 87 |
| H(8) | 9078 | 3701 | 841 | 64 |
| H(9) | 10375 | 4917 | 457 | 65 |
| H(10) | 10409 | 4539 | -329 | 56 |
| H(11A) | 9461 | 3266 | 35 | 50 |
| H(11B) | 10080 | 3426 | -536 | 50 |
| H(12) | 7566 | 4022 | -670 | 40 |
| H(15) | 5587 | 3275 | 912 | 40 |
| H(17) | 2671 | 5365 | 1545 | 52 |
| H(18) | 2625 | 4181 | 2103 | 52 |
| H(19) | 1102 | 5404 | 2360 | 51 |
| H(20) | 1152 | 4204 | 2898 | 49 |
| H(21) | -397 | 5406 | 3176 | 55 |
| H(22) | -838 | 5023 | 3949 | 55 |
| H(23A) | 223 | 3750 | 3665 | 51 |
| H(23B) | -476 | 3953 | 4216 | 51 |
| H(24) | 2129 | 4553 | 4319 | 40 |
| H(26) | 7662 | 3455 | -1452 | 51 |
| H(27) | 6981 | 2065 | -1569 | 57 |
| H(28) | 6119 | 3153 | -2152 | 76 |
| H(29A) | 5879 | 2256 | -2774 | 137 |
| H(29B) | 4277 | 2363 | -2467 | 137 |
| H(29C) | 5439 | 1778 | -2301 | 137 |
| H(30A) | 7116 | 1968 | -641 | 58 |
| H(30B) | 7143 | 2364 | -104 | 58 |
| H(30C) | 8703 | 2299 | -441 | 58 |
| H(32A) | 2858 | 3318 | -1274 | 97 |
| H(32B) | 2972 | 4104 | -1184 | 97 |
| H(32C) | 2713 | 3616 | -703 | 97 |
| H(34) | 2353 | 4274 | 5178 | 54 |
| H(35) | 1712 | 2964 | 5578 | 66 |
| H(36) | 3893 | 3891 | 5867 | 67 |
| H(37A) | 3077 | 2634 | 6330 | 117 |
| H(37B) | 3389 | 3276 | 6680 | 117 |
| H(37C) | 4792 | 2947 | 6362 | 117 |
| H(38A) | 340 | 2959 | 4516 | 81 |
| H(38B) | 1855 | 2562 | 4705 | 81 |
| H(38C) | 1683 | 2773 | 4111 | 81 |
| H(40A) | 6791 | 3772 | 4354 | 88 |
| H(40B) | 6868 | 3688 | 4969 | 88 |
| H(40C) | 6917 | 4415 | 4717 | 88 |

**Table S22** Torsion angles [°] for sh3137_a_sq (**3**).

| | | | |
|---|---|---|---|
| C(24)-O(1)-C(1)-O(2) | -0.3(6) | C(1)-O(1)-C(24)-C(23) | 111.5(4) |
| C(24)-O(1)-C(1)-C(2) | -179.4(3) | C(1)-O(1)-C(24)-C(33) | -125.0(4) |
| C(4)-N(1)-C(2)-C(3) | -0.4(5) | C(22)-C(23)-C(24)-O(1) | -85.5(4) |
| C(4)-N(1)-C(2)-C(1) | -179.9(4) | C(22)-C(23)-C(24)-C(33) | 156.8(4) |
| O(2)-C(1)-C(2)-C(3) | 170.4(5) | O(7)-C(12)-C(25)-C(31) | 48.5(4) |
| O(1)-C(1)-C(2)-C(3) | -10.4(6) | C(11)-C(12)-C(25)-C(31) | 165.8(3) |
| O(2)-C(1)-C(2)-N(1) | -10.3(6) | O(7)-C(12)-C(25)-C(30) | -70.2(4) |
| O(1)-C(1)-C(2)-N(1) | 168.9(3) | C(11)-C(12)-C(25)-C(30) | 47.0(4) |
| N(1)-C(2)-C(3)-O(3) | 0.8(5) | O(7)-C(12)-C(25)-C(26) | 164.5(3) |
| C(1)-C(2)-C(3)-O(3) | -179.9(4) | C(11)-C(12)-C(25)-C(26) | -78.3(4) |
| C(4)-O(3)-C(3)-C(2) | -0.8(5) | C(31)-C(25)-C(26)-O(4) | -168.8(4) |
| C(2)-N(1)-C(4)-O(3) | -0.1(5) | C(12)-C(25)-C(26)-O(4) | 72.7(5) |
| C(2)-N(1)-C(4)-C(5) | 176.3(5) | C(30)-C(25)-C(26)-O(4) | -52.9(5) |
| C(3)-O(3)-C(4)-N(1) | 0.5(5) | C(31)-C(25)-C(26)-C(27) | -45.3(5) |
| C(3)-O(3)-C(4)-C(5) | -176.3(4) | C(12)-C(25)-C(26)-C(27) | -163.8(3) |
| N(1)-C(4)-C(5)-C(6) | -173.0(5) | C(30)-C(25)-C(26)-C(27) | 70.7(4) |
| O(3)-C(4)-C(5)-C(6) | 3.2(8) | O(4)-C(26)-C(27)-C(28) | -118.9(5) |
| C(4)-C(5)-C(6)-C(7) | 176.2(5) | C(25)-C(26)-C(27)-C(28) | 119.0(5) |
| C(5)-C(6)-C(7)-C(8) | -168.7(6) | C(26)-C(27)-C(28)-C(29) | 177.9(6) |
| C(6)-C(7)-C(8)-C(9) | 169.6(5) | C(32)-O(6)-C(31)-O(5) | -0.3(6) |
| C(7)-C(8)-C(9)-C(10) | -161.1(5) | C(32)-O(6)-C(31)-C(25) | 179.5(4) |
| C(8)-C(9)-C(10)-C(11) | -2.7(8) | C(12)-C(25)-C(31)-O(5) | -130.7(4) |
| C(9)-C(10)-C(11)-C(12) | 93.2(5) | C(30)-C(25)-C(31)-O(5) | -8.9(6) |
| C(13)-O(7)-C(12)-C(11) | 103.8(4) | C(26)-C(25)-C(31)-O(5) | 110.1(5) |
| C(13)-O(7)-C(12)-C(25) | -133.2(3) | C(12)-C(25)-C(31)-O(6) | 49.4(4) |
| C(10)-C(11)-C(12)-O(7) | -75.6(4) | C(30)-C(25)-C(31)-O(6) | 171.3(3) |
| C(10)-C(11)-C(12)-C(25) | 168.0(3) | C(26)-C(25)-C(31)-O(6) | -69.7(5) |
| C(12)-O(7)-C(13)-O(8) | 5.6(6) | O(1)-C(24)-C(33)-C(38) | -82.5(4) |
| C(12)-O(7)-C(13)-C(14) | -175.1(3) | C(23)-C(24)-C(33)-C(38) | 35.1(5) |
| C(16)-N(2)-C(14)-C(15) | -0.5(4) | O(1)-C(24)-C(33)-C(39) | 37.5(4) |
| C(16)-N(2)-C(14)-C(13) | -178.6(4) | C(23)-C(24)-C(33)-C(39) | 155.1(4) |
| O(8)-C(13)-C(14)-C(15) | -175.5(4) | O(1)-C(24)-C(33)-C(34) | 153.4(3) |
| O(7)-C(13)-C(14)-C(15) | 5.2(6) | C(23)-C(24)-C(33)-C(34) | -88.9(4) |
| O(8)-C(13)-C(14)-N(2) | 2.3(6) | C(38)-C(33)-C(34)-O(10) | -60.3(5) |
| O(7)-C(13)-C(14)-N(2) | -177.0(3) | C(39)-C(33)-C(34)-O(10) | 178.7(4) |
| N(2)-C(14)-C(15)-O(9) | 0.3(4) | C(24)-C(33)-C(34)-O(10) | 64.3(4) |
| C(13)-C(14)-C(15)-O(9) | 178.4(4) | C(38)-C(33)-C(34)-C(35) | 61.0(5) |
| C(16)-O(9)-C(15)-C(14) | -0.1(4) | C(39)-C(33)-C(34)-C(35) | -60.0(5) |
| C(14)-N(2)-C(16)-O(9) | 0.4(4) | C(24)-C(33)-C(34)-C(35) | -174.5(4) |
| C(14)-N(2)-C(16)-C(17) | -179.9(4) | O(10)-C(34)-C(35)-C(36) | -126.8(6) |
| C(15)-O(9)-C(16)-N(2) | -0.2(4) | C(33)-C(34)-C(35)-C(36) | 112.6(6) |
| C(15)-O(9)-C(16)-C(17) | -179.9(3) | C(34)-C(35)-C(36)-C(37) | 175.6(5) |
| N(2)-C(16)-C(17)-C(18) | 177.7(4) | C(40)-O(12)-C(39)-O(11) | 2.1(7) |
| O(9)-C(16)-C(17)-C(18) | -2.6(6) | C(40)-O(12)-C(39)-C(33) | -176.5(4) |
| C(16)-C(17)-C(18)-C(19) | -179.3(4) | C(38)-C(33)-C(39)-O(11) | 6.5(7) |
| C(17)-C(18)-C(19)-C(20) | -178.3(4) | C(24)-C(33)-C(39)-O(11) | -115.5(5) |
| C(18)-C(19)-C(20)-C(21) | 175.9(4) | C(34)-C(33)-C(39)-O(11) | 128.9(5) |
| C(19)-C(20)-C(21)-C(22) | -167.3(5) | C(38)-C(33)-C(39)-O(12) | -174.8(4) |
| C(20)-C(21)-C(22)-C(23) | -1.0(8) | C(24)-C(33)-C(39)-O(12) | 63.1(5) |
| C(21)-C(22)-C(23)-C(24) | 85.1(6) | C(34)-C(33)-C(39)-O(12) | -52.5(5) |

**Table S23** Hydrogen bonds for sh3137_a_sq (**3**) [Å and °].

| D-H...A | d(D-H) | d(H...A) | d(D...A) | <(DHA) |
|---|---|---|---|---|
| O(10)-H(29)...N(2)#1 | 0.84 | 2.37 | 3.151(5) | 155.2 |

Symmetry transformations used to generate equivalent atoms:

#1 -x+1/2,-y+1,z+1/2

**Table S24** Crystal data and structure refinement for sh3191 (**6**).

| | | |
|---|---|---|
| Identification code | sh3191 | |
| Empirical formula | $C_{44}H_{58}N_2O_{15}$ | |
| Formula weight | 854.92 | |
| Temperature | 152(2) K | |
| Wavelength | 0.71073 Å | |
| Crystal system | Orthorhombic | |
| Space group | $P2_12_12_1$ | |
| Unit cell dimensions | a = 8.6801(6) Å | α = 90° |
| | b = 20.1469(16) Å | β= 90° |
| | c = 26.259(2) Å | γ= 90° |
| Volume | 4592.0(6) $Å^3$ | |
| Z | 4 | |
| Density (calculated) | 1.237 $mg/m^3$ | |
| Absorption coefficient | 0.093 $mm^{-1}$ | |
| F(000) | 1824 | |
| Crystal size | 0.471 x 0.322 x 0.212 $mm^3$ | |
| Theta range for data collection | 1.274 to 27.653° | |
| Index ranges | -11<=h<=11, -26<=k<=26, -34<=l<=34 | |
| Reflections collected | 78838 | |
| Independent reflections | 10656 [R(int) = 0.0474] | |
| Completeness to theta = 25.242° | 99.9 % | |
| Absorption correction | Semi-empirical from equivalents | |
| Max. and min. transmission | 0.9805 and 0.9575 | |
| Refinement method | Full-matrix least-squares on $F^2$ | |
| Data / restraints / parameters | 10656 / 134 / 639 | |
| Goodness-of-fit on $F^2$ | 1.055 | |
| Final R indices [I>2sigma(I)] | R1 = 0.0565, wR2 = 0.1400 | |
| R indices (all data) | R1 = 0.0831, wR2 = 0.1570 | |
| Absolute structure parameter | 0.1(2) | |
| Extinction coefficient | n/a | |
| Largest diff. peak and hole | 0.749 and -0.406 e.$Å^{-3}$ | |

**Table S25** Atomic coordinates (x $10^4$) and equivalent isotropic displacement parameters ($Å^2$ x $10^3$) for sh3191 (**6**). U(eq) is defined as one third of the trace of the orthogonalized $U^{ij}$ tensor.

| | x | y | z | U(eq) |
|---|---|---|---|---|
| N(1) | 5823(4) | 4697(2) | 2835(1) | 38(1) |
| N(2) | 4302(4) | 4772(2) | 834(1) | 37(1) |
| O(1) | 3202(3) | 3991(1) | 3748(1) | 38(1) |
| O(2) | 4211(3) | 5027(1) | 3749(1) | 42(1) |
| O(3) | 6074(4) | 3680(1) | 2517(1) | 49(1) |
| O(4) | 10448(4) | 4038(2) | 1210(1) | 64(1) |
| O(5) | 8989(3) | 2878(2) | -1288(1) | 51(1) |
| O(6) | 4144(3) | 2546(1) | -678(1) | 42(1) |
| O(7) | 4489(3) | 3584(1) | -938(1) | 46(1) |
| O(8) | 6350(3) | 3644(1) | 13(1) | 33(1) |
| O(9) | 5858(4) | 4734(1) | -115(1) | 46(1) |
| O(10) | 4135(3) | 3932(1) | 1384(1) | 42(1) |
| O(11) | 546(4) | 3975(2) | 5141(1) | 56(1) |
| O(12) | 5096(3) | 3999(1) | 4694(1) | 46(1) |
| O(13) | 4899(4) | 2950(2) | 4434(1) | 59(1) |
| C(1) | 4105(4) | 4476(2) | 3574(1) | 34(1) |
| C(2) | 4993(4) | 4247(2) | 3128(2) | 36(1) |
| C(3) | 5138(5) | 3640(2) | 2934(2) | 44(1) |
| C(4) | 6437(5) | 4335(2) | 2482(2) | 40(1) |
| C(5) | 7419(5) | 4554(2) | 2070(2) | 45(1) |
| C(6) | 8025(5) | 4171(2) | 1712(2) | 43(1) |
| C(7) | 9006(5) | 4430(2) | 1305(2) | 47(1) |
| C(8) | 9231(5) | 4091(2) | 823(2) | 41(1) |
| C(9) | 9655(4) | 4470(2) | 372(2) | 42(1) |
| C(10) | 9519(4) | 4262(2) | -100(2) | 40(1) |
| C(11) | 8971(4) | 3595(2) | -277(2) | 36(1) |
| C(12) | 7293(4) | 3602(2) | -451(1) | 33(1) |
| C(13) | 5778(4) | 4233(2) | 140(1) | 34(1) |
| C(14) | 5029(4) | 4218(2) | 639(2) | 33(1) |
| C(15) | 4933(4) | 3707(2) | 975(2) | 37(1) |
| C(16) | 3794(4) | 4581(2) | 1273(2) | 40(1) |
| C(17) | 2911(5) | 4950(3) | 1641(2) | 51(1) |
| C(18) | 2358(5) | 4671(3) | 2067(2) | 58(1) |
| C(19) | 1468(6) | 4990(3) | 2438(2) | 68(2) |
| C(20) | 1002(6) | 4679(3) | 2903(2) | 62(1) |
| C(21) | 297(6) | 5020(3) | 3290(2) | 65(1) |
| C(22) | 112(5) | 4782(3) | 3755(2) | 59(1) |
| C(23) | 551(5) | 4132(2) | 3980(2) | 47(1) |
| C(24) | 2210(4) | 4122(2) | 4188(2) | 36(1) |
| C(25) | 6747(4) | 2976(2) | -739(1) | 31(1) |
| C(26) | 7357(4) | 2998(2) | -1300(2) | 37(1) |
| C(27) | 6615(5) | 2495(2) | -1643(2) | 39(1) |
| C(28) | 5978(6) | 2649(2) | -2081(2) | 54(1) |
| C(29) | 5328(8) | 2157(3) | -2450(2) | 74(2) |
| C(30) | 7214(4) | 2336(2) | -464(2) | 36(1) |
| C(31) | 4992(4) | 2996(2) | -775(1) | 33(1) |
| C(32) | 2832(5) | 3653(3) | -954(3) | 73(2) |
| C(33) | 2566(5) | 3581(2) | 4593(2) | 40(1) |
| C(34) | 2169(5) | 3857(2) | 5128(2) | 46(1) |
| C(35A) | 2494(8) | 3380(3) | 5553(3) | 48(2) |
| C(36A) | 3519(7) | 3488(3) | 5896(2) | 54(2) |
| C(37A) | 3856(11) | 3054(5) | 6338(3) | 78(2) |
| C(35B) | 3120(30) | 3460(15) | 5532(10) | 48(4) |
| C(36B) | 2510(30) | 3139(11) | 5899(8) | 51(4) |
| C(37B) | 3220(40) | 2689(18) | 6285(11) | 68(6) |
| C(38) | 1726(6) | 2929(2) | 4491(2) | 54(1) |
| C(39) | 4300(5) | 3457(2) | 4562(2) | 42(1) |
| C(40) | 6754(5) | 3945(3) | 4646(3) | 67(2) |
| O(14) | 4013(9) | 2231(3) | 3524(2) | 124(2) |
| C(41A) | 4067(13) | 1530(5) | 3585(6) | 140(4) |
| C(42A) | 2977(13) | 1237(5) | 3851(6) | 139(4) |
| C(41B) | 3300(70) | 1960(30) | 3073(16) | 140(4) |
| C(42B) | 2330(70) | 1470(30) | 3210(30) | 140(4) |
| O(15A) | 4947(16) | 6158(6) | 3149(5) | 59(3) |
| C(43A) | 4990(20) | 6308(7) | 2601(7) | 73(4) |
| C(44A) | 4200(40) | 6947(14) | 2540(20) | 128(12) |
| O(15B) | 5414(13) | 6188(5) | 2923(5) | 63(3) |
| C(43B) | 3913(15) | 6222(5) | 2743(4) | 65(3) |
| C(44B) | 3530(30) | 6922(9) | 2652(14) | 97(8) |

**Table S26** Bond lengths [Å] and angles [°] for sh3191 (**6**).

| | |
|---|---|
| N(1)-C(4) | 1.294(5) |
| N(1)-C(2) | 1.390(5) |
| N(2)-C(16) | 1.292(5) |
| N(2)-C(14) | 1.382(5) |
| O(1)-C(1) | 1.334(4) |
| O(1)-C(24) | 1.464(4) |
| O(2)-C(1) | 1.205(4) |
| O(3)-C(4) | 1.360(5) |
| O(3)-C(3) | 1.367(5) |
| O(4)-C(8) | 1.469(5) |
| O(4)-C(7) | 1.500(6) |
| O(5)-C(26) | 1.438(5) |
| O(5)-H(14) | 0.8400 |
| O(6)-C(31) | 1.195(4) |
| O(7)-C(31) | 1.331(4) |
| O(7)-C(32) | 1.445(5) |
| O(8)-C(13) | 1.329(4) |
| O(8)-C(12) | 1.471(4) |
| O(9)-C(13) | 1.213(4) |
| O(10)-C(15) | 1.357(5) |
| O(10)-C(16) | 1.371(5) |
| O(11)-C(34) | 1.429(6) |
| O(11)-H(29) | 0.8400 |
| O(12)-C(39) | 1.338(5) |
| O(12)-C(40) | 1.449(5) |
| O(13)-C(39) | 1.195(5) |
| C(1)-C(2) | 1.476(5) |
| C(2)-C(3) | 1.330(6) |
| C(3)-H(3) | 0.9500 |
| C(4)-C(5) | 1.446(6) |
| C(5)-C(6) | 1.324(6) |
| C(5)-H(5) | 0.9500 |
| C(6)-C(7) | 1.462(6) |
| C(6)-H(6) | 0.9500 |
| C(7)-C(8) | 1.451(6) |
| C(7)-H(7) | 1.0000 |
| C(8)-C(9) | 1.458(6) |
| C(8)-H(8) | 1.0000 |
| C(9)-C(10) | 1.312(6) |
| C(9)-H(9) | 0.9500 |
| C(10)-C(11) | 1.500(6) |
| C(10)-H(10) | 0.9500 |
| C(11)-C(12) | 1.527(5) |
| C(11)-H(11A) | 0.9900 |
| C(11)-H(11B) | 0.9900 |
| C(12)-C(25) | 1.545(5) |
| C(12)-H(12) | 1.0000 |
| C(13)-C(14) | 1.462(5) |
| C(14)-C(15) | 1.359(5) |
| C(15)-H(15) | 0.9500 |
| C(16)-C(17) | 1.440(6) |
| C(17)-C(18) | 1.341(7) |
| C(17)-H(17) | 0.9500 |
| C(18)-C(19) | 1.401(7) |
| C(18)-H(18) | 0.9500 |
| C(19)-C(20) | 1.429(8) |
| C(19)-H(19) | 0.9500 |
| C(20)-C(21) | 1.371(7) |
| C(20)-H(20) | 0.9500 |
| C(21)-C(22) | 1.323(7) |
| C(21)-H(21) | 0.9500 |
| C(22)-C(23) | 1.487(7) |
| C(22)-H(22) | 0.9500 |
| C(23)-C(24) | 1.540(6) |
| C(23)-H(23A) | 0.9900 |
| C(23)-H(23B) | 0.9900 |
| C(24)-C(33) | 1.555(6) |
| C(24)-H(24) | 1.0000 |
| C(25)-C(31) | 1.527(5) |
| C(25)-C(30) | 1.532(5) |
| C(25)-C(26) | 1.567(5) |
| C(26)-C(27) | 1.500(6) |
| C(26)-H(26) | 1.0000 |
| C(27)-C(28) | 1.314(6) |
| C(27)-H(27A) | 0.9500 |
| C(28)-C(29) | 1.497(7) |
| C(28)-H(28) | 0.9500 |
| C(29)-H(29A) | 0.9800 |
| C(29)-H(29B) | 0.9800 |
| C(29)-H(29C) | 0.9800 |
| C(30)-H(30A) | 0.9800 |
| C(30)-H(30B) | 0.9800 |
| C(30)-H(30C) | 0.9800 |
| C(32)-H(32A) | 0.9800 |
| C(32)-H(32B) | 0.9800 |
| C(32)-H(32C) | 0.9800 |
| C(33)-C(38) | 1.527(6) |
| C(33)-C(39) | 1.527(6) |
| C(33)-C(34) | 1.549(6) |
| C(34)-C(35A) | 1.502(7) |
| C(34)-C(35B) | 1.57(2) |
| C(34)-H(34A) | 1.0000 |
| C(34)-H(34B) | 1.0000 |
| C(35A)-C(36A) | 1.284(9) |
| C(35A)-H(35A) | 0.9500 |
| C(36A)-C(37A) | 1.483(9) |
| C(36A)-H(36A) | 0.9500 |
| C(37A)-H(37A) | 0.9800 |
| C(37A)-H(37B) | 0.9800 |
| C(37A)-H(37C) | 0.9800 |
| C(35B)-C(36B) | 1.28(2) |
| C(35B)-H(35B) | 0.9500 |
| C(36B)-C(37B) | 1.49(2) |
| C(36B)-H(36B) | 0.9500 |
| C(37B)-H(37D) | 0.9800 |
| C(37B)-H(37E) | 0.9800 |
| C(37B)-H(37F) | 0.9800 |
| C(38)-H(38A) | 0.9800 |
| C(38)-H(38B) | 0.9800 |
| C(38)-H(38C) | 0.9800 |
| C(40)-H(40A) | 0.9800 |
| C(40)-H(40B) | 0.9800 |
| C(40)-H(40C) | 0.9800 |
| O(14)-C(41A) | 1.422(10) |
| O(14)-C(41B) | 1.44(3) |
| O(14)-H(14A) | 0.8400 |
| C(41A)-C(42A) | 1.315(16) |
| C(41A)-H(41A) | 0.9900 |
| C(41A)-H(41B) | 0.9900 |
| C(42A)-H(42A) | 0.9800 |
| C(42A)-H(42B) | 0.9800 |
| C(42A)-H(42C) | 0.9800 |
| C(41B)-C(42B) | 1.35(3) |
| C(41B)-H(41C) | 0.9900 |
| C(41B)-H(41D) | 0.9900 |
| C(42B)-H(42D) | 0.9800 |
| C(42B)-H(42E) | 0.9800 |
| C(42B)-H(42F) | 0.9800 |
| O(15A)-C(43A) | 1.47(2) |
| O(15A)-H(15A) | 0.8400 |
| C(43A)-C(44A) | 1.47(2) |
| C(43A)-H(43A) | 0.9900 |
| C(43A)-H(43B) | 0.9900 |
| C(44A)-H(44A) | 0.9800 |
| C(44A)-H(44B) | 0.9800 |
| C(44A)-H(44C) | 0.9800 |
| O(15B)-C(43B) | 1.388(17) |
| O(15B)-H(15B) | 0.8400 |
| C(43B)-C(44B) | 1.470(16) |
| C(43B)-H(43C) | 0.9900 |
| C(43B)-H(43D) | 0.9900 |
| C(44B)-H(44D) | 0.9800 |
| C(44B)-H(44E) | 0.9800 |
| C(44B)-H(44F) | 0.9800 |
| | |
| C(4)-N(1)-C(2) | 104.1(3) |

| | |
|---|---|
| C(16)-N(2)-C(14) | 104.2(3) |
| C(1)-O(1)-C(24) | 118.9(3) |
| C(4)-O(3)-C(3) | 104.4(3) |
| C(8)-O(4)-C(7) | 58.5(3) |
| C(26)-O(5)-H(14) | 109.5 |
| C(31)-O(7)-C(32) | 114.9(3) |
| C(13)-O(8)-C(12) | 117.9(3) |
| C(15)-O(10)-C(16) | 105.1(3) |
| C(34)-O(11)-H(29) | 109.5 |
| C(39)-O(12)-C(40) | 115.4(4) |
| O(2)-C(1)-O(1) | 126.0(3) |
| O(2)-C(1)-C(2) | 123.5(3) |
| O(1)-C(1)-C(2) | 110.5(3) |
| C(3)-C(2)-N(1) | 109.7(3) |
| C(3)-C(2)-C(1) | 129.8(4) |
| N(1)-C(2)-C(1) | 120.5(3) |
| C(2)-C(3)-O(3) | 108.0(4) |
| C(2)-C(3)-H(3) | 126.0 |
| O(3)-C(3)-H(3) | 126.0 |
| N(1)-C(4)-O(3) | 113.7(3) |
| N(1)-C(4)-C(5) | 127.4(4) |
| O(3)-C(4)-C(5) | 118.9(3) |
| C(6)-C(5)-C(4) | 126.0(4) |
| C(6)-C(5)-H(5) | 117.0 |
| C(4)-C(5)-H(5) | 117.0 |
| C(5)-C(6)-C(7) | 122.9(4) |
| C(5)-C(6)-H(6) | 118.6 |
| C(7)-C(6)-H(6) | 118.6 |
| C(8)-C(7)-C(6) | 123.2(4) |
| C(8)-C(7)-O(4) | 59.7(3) |
| C(6)-C(7)-O(4) | 114.9(4) |
| C(8)-C(7)-H(7) | 115.6 |
| C(6)-C(7)-H(7) | 115.6 |
| O(4)-C(7)-H(7) | 115.6 |
| C(7)-C(8)-C(9) | 119.7(4) |
| C(7)-C(8)-O(4) | 61.8(3) |
| C(9)-C(8)-O(4) | 114.7(3) |
| C(7)-C(8)-H(8) | 116.4 |
| C(9)-C(8)-H(8) | 116.4 |
| O(4)-C(8)-H(8) | 116.4 |
| C(10)-C(9)-C(8) | 125.2(4) |
| C(10)-C(9)-H(9) | 117.4 |
| C(8)-C(9)-H(9) | 117.4 |
| C(9)-C(10)-C(11) | 127.4(4) |
| C(9)-C(10)-H(10) | 116.3 |
| C(11)-C(10)-H(10) | 116.3 |
| C(10)-C(11)-C(12) | 112.7(3) |
| C(10)-C(11)-H(11A) | 109.0 |
| C(12)-C(11)-H(11A) | 109.0 |
| C(10)-C(11)-H(11B) | 109.0 |
| C(12)-C(11)-H(11B) | 109.0 |
| H(11A)-C(11)-H(11B) | 107.8 |
| O(8)-C(12)-C(11) | 106.4(3) |
| O(8)-C(12)-C(25) | 106.3(3) |
| C(11)-C(12)-C(25) | 115.5(3) |
| O(8)-C(12)-H(12) | 109.5 |
| C(11)-C(12)-H(12) | 109.5 |
| C(25)-C(12)-H(12) | 109.5 |
| O(9)-C(13)-O(8) | 125.6(3) |
| O(9)-C(13)-C(14) | 122.5(3) |
| O(8)-C(13)-C(14) | 111.9(3) |
| C(15)-C(14)-N(2) | 110.1(3) |
| C(15)-C(14)-C(13) | 128.6(3) |
| N(2)-C(14)-C(13) | 121.3(3) |
| O(10)-C(15)-C(14) | 106.9(3) |
| O(10)-C(15)-H(15) | 126.5 |
| C(14)-C(15)-H(15) | 126.5 |
| N(2)-C(16)-O(10) | 113.6(3) |
| N(2)-C(16)-C(17) | 128.7(4) |
| O(10)-C(16)-C(17) | 117.7(4) |
| C(18)-C(17)-C(16) | 122.2(5) |
| C(18)-C(17)-H(17) | 118.9 |
| C(16)-C(17)-H(17) | 118.9 |
| C(17)-C(18)-C(19) | 125.9(6) |
| C(17)-C(18)-H(18) | 117.0 |
| C(19)-C(18)-H(18) | 117.0 |
| C(18)-C(19)-C(20) | 123.3(6) |
| C(18)-C(19)-H(19) | 118.3 |
| C(20)-C(19)-H(19) | 118.3 |
| C(21)-C(20)-C(19) | 122.6(5) |
| C(21)-C(20)-H(20) | 118.7 |
| C(19)-C(20)-H(20) | 118.7 |
| C(22)-C(21)-C(20) | 124.0(6) |
| C(22)-C(21)-H(21) | 118.0 |
| C(20)-C(21)-H(21) | 118.0 |
| C(21)-C(22)-C(23) | 130.9(5) |
| C(21)-C(22)-H(22) | 114.6 |
| C(23)-C(22)-H(22) | 114.6 |
| C(22)-C(23)-C(24) | 113.0(4) |
| C(22)-C(23)-H(23A) | 109.0 |
| C(24)-C(23)-H(23A) | 109.0 |
| C(22)-C(23)-H(23B) | 109.0 |
| C(24)-C(23)-H(23B) | 109.0 |
| H(23A)-C(23)-H(23B) | 107.8 |
| O(1)-C(24)-C(23) | 105.9(3) |
| O(1)-C(24)-C(33) | 107.2(3) |
| C(23)-C(24)-C(33) | 115.9(3) |
| O(1)-C(24)-H(24) | 109.2 |
| C(23)-C(24)-H(24) | 109.2 |
| C(33)-C(24)-H(24) | 109.2 |
| C(31)-C(25)-C(30) | 108.5(3) |
| C(31)-C(25)-C(12) | 108.3(3) |
| C(30)-C(25)-C(12) | 112.1(3) |
| C(31)-C(25)-C(26) | 106.1(3) |
| C(30)-C(25)-C(26) | 112.2(3) |
| C(12)-C(25)-C(26) | 109.4(3) |
| O(5)-C(26)-C(27) | 108.8(3) |
| O(5)-C(26)-C(25) | 107.9(3) |
| C(27)-C(26)-C(25) | 113.6(3) |
| O(5)-C(26)-H(26) | 108.8 |
| C(27)-C(26)-H(26) | 108.8 |
| C(25)-C(26)-H(26) | 108.8 |
| C(28)-C(27)-C(26) | 123.2(4) |
| C(28)-C(27)-H(27A) | 118.4 |
| C(26)-C(27)-H(27A) | 118.4 |
| C(27)-C(28)-C(29) | 124.7(4) |
| C(27)-C(28)-H(28) | 117.7 |
| C(29)-C(28)-H(28) | 117.7 |
| C(28)-C(29)-H(29A) | 109.5 |
| C(28)-C(29)-H(29B) | 109.5 |
| H(29A)-C(29)-H(29B) | 109.5 |
| C(28)-C(29)-H(29C) | 109.5 |
| H(29A)-C(29)-H(29C) | 109.5 |
| H(29B)-C(29)-H(29C) | 109.5 |
| C(25)-C(30)-H(30A) | 109.5 |
| C(25)-C(30)-H(30B) | 109.5 |
| H(30A)-C(30)-H(30B) | 109.5 |
| C(25)-C(30)-H(30C) | 109.5 |
| H(30A)-C(30)-H(30C) | 109.5 |
| H(30B)-C(30)-H(30C) | 109.5 |
| O(6)-C(31)-O(7) | 122.7(3) |
| O(6)-C(31)-C(25) | 125.4(3) |
| O(7)-C(31)-C(25) | 111.8(3) |
| O(7)-C(32)-H(32A) | 109.5 |
| O(7)-C(32)-H(32B) | 109.5 |
| H(32A)-C(32)-H(32B) | 109.5 |
| O(7)-C(32)-H(32C) | 109.5 |
| H(32A)-C(32)-H(32C) | 109.5 |
| H(32B)-C(32)-H(32C) | 109.5 |
| C(38)-C(33)-C(39) | 108.7(3) |
| C(38)-C(33)-C(34) | 111.2(4) |
| C(39)-C(33)-C(34) | 109.0(4) |
| C(38)-C(33)-C(24) | 112.8(4) |
| C(39)-C(33)-C(24) | 105.9(3) |
| C(34)-C(33)-C(24) | 108.9(3) |
| O(11)-C(34)-C(35A) | 105.8(4) |
| O(11)-C(34)-C(33) | 107.6(4) |
| C(35A)-C(34)-C(33) | 113.7(4) |
| O(11)-C(34)-C(35B) | 126.1(10) |
| C(33)-C(34)-C(35B) | 108.2(12) |

| | |
|---|---|
| O(11)-C(34)-H(34A) | 109.9 |
| C(35A)-C(34)-H(34A) | 109.9 |
| C(33)-C(34)-H(34A) | 109.9 |
| O(11)-C(34)-H(34B) | 104.3 |
| C(33)-C(34)-H(34B) | 104.3 |
| C(35B)-C(34)-H(34B) | 104.3 |
| C(36A)-C(35A)-C(34) | 122.9(6) |
| C(36A)-C(35A)-H(35A) | 118.6 |
| C(34)-C(35A)-H(35A) | 118.6 |
| C(35A)-C(36A)-C(37A) | 125.8(7) |
| C(35A)-C(36A)-H(36A) | 117.1 |
| C(37A)-C(36A)-H(36A) | 117.1 |
| C(36A)-C(37A)-H(37A) | 109.5 |
| C(36A)-C(37A)-H(37B) | 109.5 |
| H(37A)-C(37A)-H(37B) | 109.5 |
| C(36A)-C(37A)-H(37C) | 109.5 |
| H(37A)-C(37A)-H(37C) | 109.5 |
| H(37B)-C(37A)-H(37C) | 109.5 |
| C(36B)-C(35B)-C(34) | 123(2) |
| C(36B)-C(35B)-H(35B) | 118.3 |
| C(34)-C(35B)-H(35B) | 118.3 |
| C(35B)-C(36B)-C(37B) | 130(2) |
| C(35B)-C(36B)-H(36B) | 114.8 |
| C(37B)-C(36B)-H(36B) | 114.8 |
| C(36B)-C(37B)-H(37D) | 109.5 |
| C(36B)-C(37B)-H(37E) | 109.5 |
| H(37D)-C(37B)-H(37E) | 109.5 |
| C(36B)-C(37B)-H(37F) | 109.5 |
| H(37D)-C(37B)-H(37F) | 109.5 |
| H(37E)-C(37B)-H(37F) | 109.5 |
| C(33)-C(38)-H(38A) | 109.5 |
| C(33)-C(38)-H(38B) | 109.5 |
| H(38A)-C(38)-H(38B) | 109.5 |
| C(33)-C(38)-H(38C) | 109.5 |
| H(38A)-C(38)-H(38C) | 109.5 |
| H(38B)-C(38)-H(38C) | 109.5 |
| O(13)-C(39)-O(12) | 123.0(4) |
| O(13)-C(39)-C(33) | 125.7(4) |
| O(12)-C(39)-C(33) | 111.2(3) |
| O(12)-C(40)-H(40A) | 109.5 |
| O(12)-C(40)-H(40B) | 109.5 |
| H(40A)-C(40)-H(40B) | 109.5 |
| O(12)-C(40)-H(40C) | 109.5 |
| H(40A)-C(40)-H(40C) | 109.5 |
| H(40B)-C(40)-H(40C) | 109.5 |
| C(41A)-O(14)-H(14A) | 109.5 |
| C(42A)-C(41A)-O(14) | 118.8(11) |
| C(42A)-C(41A)-H(41A) | 107.6 |
| O(14)-C(41A)-H(41A) | 107.6 |
| C(42A)-C(41A)-H(41B) | 107.6 |
| O(14)-C(41A)-H(41B) | 107.6 |
| H(41A)-C(41A)-H(41B) | 107.0 |
| C(41A)-C(42A)-H(42A) | 109.5 |
| C(41A)-C(42A)-H(42B) | 109.5 |
| H(42A)-C(42A)-H(42B) | 109.5 |
| C(41A)-C(42A)-H(42C) | 109.5 |
| H(42A)-C(42A)-H(42C) | 109.5 |
| H(42B)-C(42A)-H(42C) | 109.5 |
| C(42B)-C(41B)-O(14) | 109(3) |
| C(42B)-C(41B)-H(41C) | 109.9 |
| O(14)-C(41B)-H(41C) | 109.9 |
| C(42B)-C(41B)-H(41D) | 109.8 |
| O(14)-C(41B)-H(41D) | 109.9 |
| H(41C)-C(41B)-H(41D) | 108.3 |
| C(41B)-C(42B)-H(42D) | 109.5 |
| C(41B)-C(42B)-H(42E) | 109.5 |
| H(42D)-C(42B)-H(42E) | 109.5 |
| C(41B)-C(42B)-H(42F) | 109.4 |
| H(42D)-C(42B)-H(42F) | 109.5 |
| H(42E)-C(42B)-H(42F) | 109.5 |
| C(43A)-O(15A)-H(15A) | 109.5 |
| O(15A)-C(43A)-C(44A) | 106(2) |
| O(15A)-C(43A)-H(43A) | 110.5 |
| C(44A)-C(43A)-H(43A) | 110.5 |
| O(15A)-C(43A)-H(43B) | 110.5 |
| C(44A)-C(43A)-H(43B) | 110.5 |
| H(43A)-C(43A)-H(43B) | 108.7 |
| C(43A)-C(44A)-H(44A) | 109.5 |
| C(43A)-C(44A)-H(44B) | 109.5 |
| H(44A)-C(44A)-H(44B) | 109.5 |
| C(43A)-C(44A)-H(44C) | 109.5 |
| H(44A)-C(44A)-H(44C) | 109.5 |
| H(44B)-C(44A)-H(44C) | 109.5 |
| C(43B)-O(15B)-H(15B) | 109.5 |
| O(15B)-C(43B)-C(44B) | 108.5(13) |
| O(15B)-C(43B)-H(43C) | 110.0 |
| C(44B)-C(43B)-H(43C) | 110.0 |
| O(15B)-C(43B)-H(43D) | 110.0 |
| C(44B)-C(43B)-H(43D) | 110.0 |
| H(43C)-C(43B)-H(43D) | 108.4 |
| C(43B)-C(44B)-H(44D) | 109.5 |
| C(43B)-C(44B)-H(44E) | 109.5 |
| H(44D)-C(44B)-H(44E) | 109.5 |
| C(43B)-C(44B)-H(44F) | 109.5 |
| H(44D)-C(44B)-H(44F) | 109.5 |
| H(44E)-C(44B)-H(44F) | 109.5 |

**Table S27** Anisotropic displacement parameters ($Å^2 \times 10^3$) for sh3191 (**6**). The anisotropic displacement factor exponent takes the form: $-2\pi^2[ h^2 a^{*2}U^{11} + ... + 2 h k a^* b^* U^{12} ]$.

| | $U^{11}$ | $U^{22}$ | $U^{33}$ | $U^{23}$ | $U^{13}$ | $U^{12}$ |
|---|---|---|---|---|---|---|
| N(1) | 32(2) | 37(2) | 46(2) | -1(1) | 4(2) | 1(1) |
| N(2) | 34(2) | 39(2) | 37(2) | 2(1) | -1(1) | 2(1) |
| O(1) | 35(1) | 35(1) | 43(2) | -6(1) | 9(1) | -1(1) |
| O(2) | 43(2) | 36(1) | 47(2) | -5(1) | 8(1) | 0(1) |
| O(3) | 53(2) | 40(2) | 55(2) | -7(1) | 16(2) | 3(1) |
| O(4) | 44(2) | 78(2) | 70(2) | -8(2) | -2(2) | 4(2) |
| O(5) | 32(1) | 72(2) | 48(2) | -2(2) | 10(1) | -1(1) |
| O(6) | 32(1) | 38(1) | 56(2) | 11(1) | 3(1) | -3(1) |
| O(7) | 25(1) | 39(1) | 73(2) | 16(1) | -3(1) | 7(1) |
| O(8) | 30(1) | 31(1) | 38(1) | 6(1) | 4(1) | 3(1) |
| O(9) | 54(2) | 33(1) | 51(2) | 7(1) | 11(1) | 6(1) |
| O(10) | 34(1) | 53(2) | 39(2) | 6(1) | -2(1) | -4(1) |
| O(11) | 62(2) | 39(2) | 65(2) | -1(2) | 21(2) | 2(1) |
| O(12) | 40(1) | 31(1) | 66(2) | -2(1) | -4(1) | -2(1) |
| O(13) | 56(2) | 33(2) | 89(2) | -10(2) | -3(2) | 9(1) |
| C(1) | 28(2) | 35(2) | 39(2) | -2(2) | 2(2) | 3(2) |
| C(2) | 30(2) | 37(2) | 41(2) | 1(2) | 2(2) | 3(2) |
| C(3) | 46(2) | 40(2) | 46(2) | -2(2) | 11(2) | 2(2) |
| C(4) | 35(2) | 43(2) | 41(2) | -4(2) | 3(2) | 2(2) |
| C(5) | 37(2) | 45(2) | 51(2) | -1(2) | 5(2) | -5(2) |
| C(6) | 36(2) | 51(2) | 42(2) | -1(2) | 1(2) | -6(2) |
| C(7) | 43(2) | 45(2) | 53(2) | -5(2) | 9(2) | -12(2) |
| C(8) | 31(2) | 50(2) | 43(2) | 2(2) | -4(2) | -11(2) |
| C(9) | 31(2) | 41(2) | 54(3) | 5(2) | 0(2) | -10(2) |
| C(10) | 34(2) | 43(2) | 44(2) | 6(2) | 5(2) | -6(2) |
| C(11) | 26(2) | 41(2) | 42(2) | 5(2) | 2(2) | 2(2) |
| C(12) | 26(2) | 34(2) | 38(2) | 6(2) | 5(2) | 2(1) |
| C(13) | 27(2) | 34(2) | 43(2) | 4(2) | -1(2) | -1(2) |
| C(14) | 24(2) | 33(2) | 41(2) | 0(2) | -3(2) | -4(1) |
| C(15) | 30(2) | 41(2) | 40(2) | 2(2) | -4(2) | -3(2) |
| C(16) | 30(2) | 50(2) | 40(2) | -1(2) | -2(2) | 1(2) |
| C(17) | 40(2) | 70(3) | 44(2) | -6(2) | 3(2) | 5(2) |
| C(18) | 35(2) | 100(4) | 39(2) | -5(2) | -4(2) | 1(2) |
| C(19) | 51(3) | 105(4) | 47(3) | -11(3) | -6(2) | 10(3) |
| C(20) | 43(2) | 68(3) | 75(3) | -16(3) | -19(3) | 4(2) |
| C(21) | 49(3) | 82(4) | 63(3) | -7(3) | -6(2) | 14(3) |
| C(22) | 41(2) | 83(3) | 53(3) | 1(3) | -3(2) | 6(2) |
| C(23) | 35(2) | 57(3) | 49(2) | -6(2) | 5(2) | -4(2) |
| C(24) | 32(2) | 36(2) | 42(2) | -3(2) | 7(2) | 0(2) |
| C(25) | 26(2) | 33(2) | 35(2) | 6(2) | 2(1) | 3(1) |
| C(26) | 33(2) | 42(2) | 36(2) | 8(2) | 2(2) | 2(2) |
| C(27) | 39(2) | 40(2) | 38(2) | 4(2) | 5(2) | 2(2) |
| C(28) | 72(3) | 46(2) | 43(2) | 2(2) | -4(2) | 7(2) |
| C(29) | 94(4) | 76(4) | 52(3) | -10(3) | -19(3) | 6(3) |
| C(30) | 33(2) | 33(2) | 43(2) | 5(2) | -2(2) | 4(2) |
| C(31) | 29(2) | 34(2) | 37(2) | 4(2) | 4(2) | 4(2) |
| C(32) | 27(2) | 61(3) | 132(5) | 26(3) | -8(3) | 9(2) |
| C(33) | 44(2) | 28(2) | 49(2) | -2(2) | 4(2) | -1(2) |
| C(34) | 58(3) | 31(2) | 51(2) | 2(2) | 10(2) | 0(2) |
| C(35A) | 53(4) | 36(3) | 54(3) | 8(2) | 19(3) | -2(3) |
| C(36A) | 54(3) | 62(3) | 47(3) | 6(3) | 15(3) | 8(3) |
| C(37A) | 82(6) | 90(6) | 61(4) | 19(4) | 10(4) | 29(5) |
| C(35B) | 52(8) | 49(8) | 43(7) | 8(7) | 22(8) | 4(8) |
| C(36B) | 55(7) | 53(7) | 44(7) | 4(6) | 16(7) | 5(7) |
| C(37B) | 72(13) | 83(14) | 49(11) | 8(12) | 8(11) | 31(11) |
| C(38) | 59(3) | 34(2) | 70(3) | -4(2) | 8(2) | -10(2) |
| C(39) | 49(2) | 28(2) | 48(2) | 1(2) | -3(2) | 2(2) |
| C(40) | 38(2) | 51(3) | 111(5) | -3(3) | -11(3) | -1(2) |
| O(14) | 171(6) | 89(4) | 111(4) | -10(3) | 8(4) | -6(4) |
| C(41A) | 110(6) | 80(5) | 230(11) | -16(6) | -34(7) | 2(4) |
| C(42A) | 109(6) | 76(5) | 231(11) | -11(6) | -37(7) | 0(4) |
| C(41B) | 110(7) | 80(6) | 230(11) | -16(6) | -35(7) | 2(5) |
| C(42B) | 110(7) | 79(6) | 231(11) | -14(6) | -36(7) | 1(5) |
| O(15A) | 66(7) | 45(4) | 68(8) | 12(5) | -4(5) | -8(5) |
| C(43A) | 76(8) | 65(7) | 79(8) | -5(6) | -10(7) | -3(7) |
| C(44A) | 160(30) | 115(13) | 110(20) | 12(12) | -60(20) | 15(15) |
| O(15B) | 61(6) | 36(4) | 92(9) | -9(5) | -18(5) | -8(4) |
| C(43B) | 67(7) | 63(6) | 66(6) | -10(4) | -13(5) | -2(5) |
| C(44B) | 113(17) | 73(8) | 106(17) | 22(7) | -55(16) | 24(9) |

**Table S28** Hydrogen coordinates (x $10^4$) and isotropic displacement parameters ($Å^2$ x $10^3$) for **6.**

| | x | y | z | U(eq) |
|---|---|---|---|---|
| H(14) | 9425 | 3116 | -1507 | 76 |
| H(29) | 378 | 4368 | 5232 | 83 |
| H(3) | 4672 | 3248 | 3063 | 53 |
| H(5) | 7651 | 5014 | 2054 | 54 |
| H(6) | 7814 | 3709 | 1720 | 52 |
| H(7) | 9125 | 4923 | 1295 | 56 |
| H(8) | 8594 | 3684 | 765 | 50 |
| H(9) | 10063 | 4902 | 423 | 51 |
| H(10) | 9799 | 4569 | -358 | 48 |
| H(11A) | 9086 | 3271 | 5 | 44 |
| H(11B) | 9630 | 3444 | -562 | 44 |
| H(12) | 7106 | 4003 | -667 | 39 |
| H(15) | 5347 | 3274 | 932 | 45 |
| H(17) | 2713 | 5407 | 1579 | 61 |
| H(18) | 2592 | 4216 | 2122 | 69 |
| H(19) | 1158 | 5436 | 2379 | 81 |
| H(20) | 1188 | 4218 | 2945 | 75 |
| H(21) | -82 | 5453 | 3219 | 78 |
| H(22) | -385 | 5077 | 3985 | 71 |
| H(23A) | 445 | 3783 | 3717 | 56 |
| H(23B) | -169 | 4025 | 4260 | 56 |
| H(24) | 2466 | 4569 | 4331 | 44 |
| H(26) | 7169 | 3451 | -1442 | 45 |
| H(27A) | 6605 | 2044 | -1538 | 47 |
| H(28) | 5926 | 3106 | -2170 | 64 |
| H(29A) | 4289 | 2294 | -2551 | 111 |
| H(29B) | 5280 | 1718 | -2288 | 111 |
| H(29C) | 5989 | 2134 | -2752 | 111 |
| H(30A) | 6778 | 1953 | -643 | 54 |
| H(30B) | 6824 | 2345 | -114 | 54 |
| H(30C) | 8340 | 2299 | -459 | 54 |
| H(32A) | 2564 | 4085 | -1100 | 110 |
| H(32B) | 2417 | 3623 | -607 | 110 |
| H(32C) | 2392 | 3298 | -1164 | 110 |
| H(34A) | 2736 | 4283 | 5187 | 56 |
| H(34B) | 2635 | 4311 | 5131 | 56 |
| H(35A) | 1917 | 2980 | 5569 | 57 |
| H(36A) | 4114 | 3882 | 5863 | 65 |
| H(37A) | 4964 | 2963 | 6351 | 117 |
| H(37B) | 3291 | 2636 | 6302 | 117 |
| H(37C) | 3537 | 3277 | 6652 | 117 |
| H(35B) | 4214 | 3457 | 5502 | 58 |
| H(36B) | 1429 | 3196 | 5932 | 61 |
| H(37D) | 2778 | 2784 | 6621 | 102 |
| H(37E) | 4334 | 2762 | 6294 | 102 |
| H(37F) | 3006 | 2227 | 6193 | 102 |
| H(38A) | 2157 | 2579 | 4708 | 81 |
| H(38B) | 1854 | 2805 | 4132 | 81 |
| H(38C) | 627 | 2983 | 4567 | 81 |
| H(40A) | 7229 | 4375 | 4721 | 100 |
| H(40B) | 7018 | 3811 | 4298 | 100 |
| H(40C) | 7138 | 3612 | 4887 | 100 |
| H(14A) | 4366 | 2415 | 3786 | 186 |
| H(41A) | 4071 | 1329 | 3241 | 168 |
| H(41B) | 5066 | 1419 | 3746 | 168 |
| H(42A) | 3168 | 758 | 3860 | 208 |
| H(42B) | 2975 | 1413 | 4199 | 208 |
| H(42C) | 1975 | 1322 | 3692 | 208 |
| H(41C) | 2730 | 2311 | 2891 | 168 |
| H(41D) | 4104 | 1782 | 2842 | 168 |
| H(42D) | 1841 | 1280 | 2905 | 210 |
| H(42E) | 2902 | 1118 | 3388 | 210 |
| H(42F) | 1531 | 1645 | 3437 | 210 |
| H(15A) | 5201 | 5761 | 3196 | 89 |
| H(43A) | 4467 | 5956 | 2405 | 88 |
| H(43B) | 6073 | 6341 | 2482 | 88 |
| H(44A) | 4389 | 7229 | 2834 | 192 |
| H(44B) | 3089 | 6870 | 2500 | 192 |
| H(44C) | 4588 | 7169 | 2230 | 192 |
| H(15B) | 5890 | 5885 | 2770 | 95 |
| H(43C) | 3197 | 6029 | 2997 | 78 |
| H(43D) | 3819 | 5966 | 2423 | 78 |
| H(44D) | 4005 | 7198 | 2915 | 146 |
| H(44E) | 2404 | 6978 | 2664 | 146 |
| H(44F) | 3907 | 7055 | 2316 | 146 |

**Table S29** Torsion angles [°] for sh3191 (**6**).

| | |
|---|---|
| C(24)-O(1)-C(1)-O(2) | 1.2(6) |
| C(24)-O(1)-C(1)-C(2) | -179.3(3) |
| C(4)-N(1)-C(2)-C(3) | -0.6(5) |
| C(4)-N(1)-C(2)-C(1) | -179.5(3) |
| O(2)-C(1)-C(2)-C(3) | 170.7(4) |
| O(1)-C(1)-C(2)-C(3) | -8.9(6) |
| O(2)-C(1)-C(2)-N(1) | -10.7(6) |
| O(1)-C(1)-C(2)-N(1) | 169.7(3) |
| N(1)-C(2)-C(3)-O(3) | 0.7(5) |
| C(1)-C(2)-C(3)-O(3) | 179.4(4) |
| C(4)-O(3)-C(3)-C(2) | -0.5(5) |
| C(2)-N(1)-C(4)-O(3) | 0.3(5) |
| C(2)-N(1)-C(4)-C(5) | 179.7(4) |
| C(3)-O(3)-C(4)-N(1) | 0.1(5) |
| C(3)-O(3)-C(4)-C(5) | -179.4(4) |
| N(1)-C(4)-C(5)-C(6) | -179.6(4) |
| O(3)-C(4)-C(5)-C(6) | -0.2(7) |
| C(4)-C(5)-C(6)-C(7) | 179.8(4) |
| C(5)-C(6)-C(7)-C(8) | -157.4(4) |
| C(5)-C(6)-C(7)-O(4) | 133.8(4) |
| C(8)-O(4)-C(7)-C(6) | 115.4(4) |
| C(6)-C(7)-C(8)-C(9) | 154.6(4) |
| O(4)-C(7)-C(8)-C(9) | -103.9(4) |
| C(6)-C(7)-C(8)-O(4) | -101.5(5) |
| C(7)-O(4)-C(8)-C(9) | 111.9(4) |
| C(7)-C(8)-C(9)-C(10) | -162.0(4) |
| O(4)-C(8)-C(9)-C(10) | 127.6(4) |
| C(8)-C(9)-C(10)-C(11) | -2.6(7) |
| C(9)-C(10)-C(11)-C(12) | 100.3(5) |
| C(13)-O(8)-C(12)-C(11) | 100.7(3) |
| C(13)-O(8)-C(12)-C(25) | -135.7(3) |
| C(10)-C(11)-C(12)-O(8) | -74.2(4) |
| C(10)-C(11)-C(12)-C(25) | 168.1(3) |
| C(12)-O(8)-C(13)-O(9) | 6.5(5) |
| C(12)-O(8)-C(13)-C(14) | -173.0(3) |
| C(16)-N(2)-C(14)-C(15) | -0.3(4) |
| C(16)-N(2)-C(14)-C(13) | -179.6(3) |
| O(9)-C(13)-C(14)-C(15) | -175.9(4) |
| O(8)-C(13)-C(14)-C(15) | 3.6(5) |
| O(9)-C(13)-C(14)-N(2) | 3.2(5) |
| O(8)-C(13)-C(14)-N(2) | -177.3(3) |
| C(16)-O(10)-C(15)-C(14) | -0.4(4) |
| N(2)-C(14)-C(15)-O(10) | 0.5(4) |
| C(13)-C(14)-C(15)-O(10) | 179.6(3) |
| C(14)-N(2)-C(16)-O(10) | 0.0(4) |
| C(14)-N(2)-C(16)-C(17) | -178.4(4) |
| C(15)-O(10)-C(16)-N(2) | 0.2(4) |
| C(15)-O(10)-C(16)-C(17) | 178.9(3) |
| N(2)-C(16)-C(17)-C(18) | 174.9(4) |
| O(10)-C(16)-C(17)-C(18) | -3.5(6) |
| C(16)-C(17)-C(18)-C(19) | -178.7(5) |
| C(17)-C(18)-C(19)-C(20) | -175.5(5) |
| C(18)-C(19)-C(20)-C(21) | 171.7(5) |
| C(19)-C(20)-C(21)-C(22) | -166.8(5) |
| C(20)-C(21)-C(22)-C(23) | -0.8(9) |
| C(21)-C(22)-C(23)-C(24) | 86.9(6) |
| C(1)-O(1)-C(24)-C(23) | 109.9(4) |
| C(1)-O(1)-C(24)-C(33) | -125.8(3) |
| C(22)-C(23)-C(24)-O(1) | -82.9(4) |
| C(22)-C(23)-C(24)-C(33) | 158.4(4) |
| O(8)-C(12)-C(25)-C(31) | 49.4(4) |
| C(11)-C(12)-C(25)-C(31) | 167.2(3) |
| O(8)-C(12)-C(25)-C(30) | -70.3(3) |
| C(11)-C(12)-C(25)-C(30) | 47.5(4) |
| O(8)-C(12)-C(25)-C(26) | 164.6(3) |
| C(11)-C(12)-C(25)-C(26) | -77.6(4) |
| C(31)-C(25)-C(26)-O(5) | -171.4(3) |
| C(30)-C(25)-C(26)-O(5) | -53.1(4) |
| C(12)-C(25)-C(26)-O(5) | 72.0(4) |
| C(31)-C(25)-C(26)-C(27) | -50.7(4) |
| C(30)-C(25)-C(26)-C(27) | 67.6(4) |
| C(12)-C(25)-C(26)-C(27) | -167.3(3) |
| O(5)-C(26)-C(27)-C(28) | -112.0(5) |
| C(25)-C(26)-C(27)-C(28) | 127.8(4) |
| C(26)-C(27)-C(28)-C(29) | 175.4(5) |
| C(32)-O(7)-C(31)-O(6) | 4.3(6) |
| C(32)-O(7)-C(31)-C(25) | -176.5(4) |
| C(30)-C(25)-C(31)-O(6) | -10.5(5) |
| C(12)-C(25)-C(31)-O(6) | -132.4(4) |
| C(26)-C(25)-C(31)-O(6) | 110.2(4) |
| C(30)-C(25)-C(31)-O(7) | 170.3(3) |
| C(12)-C(25)-C(31)-O(7) | 48.3(4) |
| C(26)-C(25)-C(31)-O(7) | -69.0(4) |
| O(1)-C(24)-C(33)-C(38) | -81.7(4) |
| C(23)-C(24)-C(33)-C(38) | 36.3(5) |
| O(1)-C(24)-C(33)-C(39) | 37.2(4) |
| C(23)-C(24)-C(33)-C(39) | 155.2(3) |
| O(1)-C(24)-C(33)-C(34) | 154.3(3) |
| C(23)-C(24)-C(33)-C(34) | -87.7(4) |
| C(38)-C(33)-C(34)-O(11) | -60.8(4) |
| C(39)-C(33)-C(34)-O(11) | 179.3(3) |
| C(24)-C(33)-C(34)-O(11) | 64.2(4) |
| C(38)-C(33)-C(34)-C(35A) | 56.1(6) |
| C(39)-C(33)-C(34)-C(35A) | -63.8(5) |
| C(24)-C(33)-C(34)-C(35A) | -179.0(4) |
| C(38)-C(33)-C(34)-C(35B) | 78.2(12) |
| C(39)-C(33)-C(34)-C(35B) | -41.7(12) |
| C(24)-C(33)-C(34)-C(35B) | -156.9(12) |
| O(11)-C(34)-C(35A)-C(36A) | -127.3(7) |
| C(33)-C(34)-C(35A)-C(36A) | 114.9(7) |
| C(34)-C(35A)-C(36A)-C(37A) | 177.2(6) |
| O(11)-C(34)-C(35B)-C(36B) | 7(4) |
| C(33)-C(34)-C(35B)-C(36B) | -122(3) |
| C(34)-C(35B)-C(36B)-C(37B) | 174(3) |
| C(40)-O(12)-C(39)-O(13) | 3.1(7) |
| C(40)-O(12)-C(39)-C(33) | -176.3(4) |
| C(38)-C(33)-C(39)-O(13) | 6.3(6) |
| C(34)-C(33)-C(39)-O(13) | 127.7(5) |
| C(24)-C(33)-C(39)-O(13) | -115.2(5) |
| C(38)-C(33)-C(39)-O(12) | -174.4(4) |
| C(34)-C(33)-C(39)-O(12) | -53.0(4) |
| C(24)-C(33)-C(39)-O(12) | 64.1(4) |

**Table S30** Hydrogen bonds for sh3191 (**6**) [Å and °].

| D-H...A | d(D-H) | d(H...A) | d(D...A) | <(DHA) |
|---|---|---|---|---|
| O(11)-H(29)...N(2)#1 | 0.84 | 2.36 | 3.115(4) | 149.4 |
| O(5)-H(14)...O(15A^a)#2 | 0.84 | 1.80 | 2.609(14) | 160.6 |
| O(5)-H(14)...O(15B^b)#2 | 0.84 | 2.05 | 2.846(11) | 156.9 |
| O(15A^a)-H(15A^a)...N(1) | 0.84 | 2.41 | 3.151(14) | 148.2 |
| O(15B^b)-H(15B^b)...N(1) | 0.84 | 2.40 | 3.034(10) | 132.7 |
| O(14)-H(14A^a)...O(13) | 0.84 | 2.07 | 2.900(7) | 170.7 |

Symmetry transformations used to generate equivalent atoms:

#1 -x+1/2,-y+1,z+1/2 #2 -x+3/2,-y+1,z-1/2

**Table S31** Crystal data and structure refinement for sh3279 (**7**).

| | | |
|---|---|---|
| Identification code | sh3279 | |
| Empirical formula | $C_{44}H_{58}N_2O_{15}$ | |
| Formula weight | 854.92 | |
| Temperature | 133(2) K | |
| Wavelength | 0.71073 Å | |
| Crystal system | Orthorhombic | |
| Space group | $P2_12_12_1$ | |
| Unit cell dimensions | a = 8.7058(6) Å | α= 90° |
| | b = 20.2194(15) Å | β= 90° |
| | c = 26.000(2) Å | γ = 90° |
| Volume | 4576.7(6) Å3 | |
| Z | 4 | |
| Density (calculated) | 1.241 mg/m$^3$ | |
| Absorption coefficient | 0.093 mm$^{-1}$ | |
| F(000) | 1824 | |
| Crystal size | 0.530 x 0.280 x 0.240 mm$^3$ | |
| Theta range for data collection | 1.276 to 27.915° | |
| Index ranges | -6<=h<=11, -23<=k<=26, -33<=l<=34 | |
| Reflections collected | 24427 | |
| Independent reflections | 10915 [R(int) = 0.0400] | |
| Completeness to theta = 25.242° | 99.9 % | |
| Absorption correction | Semi-empirical from equivalents | |
| Max. and min. transmission | 0.9779 and 0.9522 | |
| Refinement method | Full-matrix least-squares on F$^2$ | |
| Data / restraints / parameters | 10915 / 221 / 626 | |
| Goodness-of-fit on F$^2$ | 1.025 | |
| Final R indices [I>2sigma(I)] | R1 = 0.0529, wR2 = 0.1229 | |
| R indices (all data) | R1 = 0.0880, wR2 = 0.1405 | |
| Absolute structure parameter | 0.5(5) | |
| Extinction coefficient | n/a | |
| Largest diff. peak and hole | 0.431 and -0.412 e.Å$^{-3}$ | |

**Table S32** Atomic coordinates (x $10^4$) and equivalent isotropic displacement parameters (Å$^2$ x $10^3$) for sh3279 (**7**). U(eq) is defined as one third of the trace of the orthogonalized $U^{ij}$ tensor.

| | x | y | z | U(eq) |
|---|---|---|---|---|
| N(1) | 5713(3) | 4758(2) | 2831(1) | 30(1) |
| N(2) | 4381(3) | 4753(2) | 831(1) | 26(1) |
| O(1) | 3132(3) | 4016(1) | 3749(1) | 28(1) |
| O(2) | 4012(3) | 5075(1) | 3740(1) | 32(1) |
| O(3) | 6135(3) | 3742(1) | 2531(1) | 37(1) |
| O(4A) | 11256(3) | 4306(2) | 144(1) | 35(1) |
| O(4B) | -1380(13) | 4723(8) | 3491(5) | 63(5) |
| O(5) | 9204(3) | 2882(1) | -1294(1) | 38(1) |
| O(6) | 4725(3) | 3589(1) | -907(1) | 32(1) |
| O(7) | 4422(3) | 2529(1) | -683(1) | 32(1) |
| O(8) | 6599(3) | 3650(1) | 29(1) | 24(1) |
| O(9) | 6237(3) | 4751(1) | -78(1) | 36(1) |
| O(10) | 4115(3) | 3901(1) | 1371(1) | 29(1) |
| O(11) | 562(3) | 4012(1) | 5163(1) | 43(1) |
| O(12) | 4802(3) | 2940(1) | 4428(1) | 45(1) |
| O(13) | 5065(3) | 3992(1) | 4682(1) | 34(1) |
| C(1) | 4001(4) | 4521(2) | 3570(1) | 27(1) |
| C(2) | 4939(4) | 4301(2) | 3131(1) | 28(1) |
| C(3) | 5196(4) | 3689(2) | 2949(2) | 37(1) |
| C(4) | 6396(4) | 4407(2) | 2480(1) | 32(1) |
| C(5) | 7302(4) | 4640(2) | 2055(2) | 36(1) |
| C(6) | 7935(4) | 4267(2) | 1688(1) | 31(1) |
| C(7) | 8729(4) | 4540(2) | 1249(1) | 32(1) |
| C(8) | 9249(4) | 4206(2) | 846(1) | 29(1) |
| C(9) | 9848(4) | 4545(2) | 392(1) | 31(1) |
| C(10) | 9806(4) | 4283(2) | -123(1) | 31(1) |
| C(11) | 9221(4) | 3609(2) | -266(1) | 27(1) |
| C(12) | 7539(4) | 3613(2) | -439(1) | 24(1) |
| C(13) | 6044(4) | 4246(2) | 158(1) | 25(1) |
| C(14) | 5172(4) | 4206(2) | 644(1) | 24(1) |
| C(15) | 5012(4) | 3697(2) | 973(1) | 25(1) |
| C(16) | 3766(4) | 4548(2) | 1259(1) | 26(1) |
| C(17) | 2820(4) | 4903(2) | 1617(1) | 33(1) |
| C(18) | 2275(4) | 4627(2) | 2050(1) | 34(1) |
| C(19) | 1355(4) | 4953(2) | 2429(2) | 40(1) |
| C(20) | 926(4) | 4669(2) | 2878(2) | 36(1) |
| C(21) | 152(4) | 5014(2) | 3282(2) | 40(1) |
| C(22) | -14(4) | 4787(2) | 3779(2) | 41(1) |
| C(23) | 487(4) | 4139(2) | 3991(2) | 36(1) |
| C(24) | 2136(4) | 4138(2) | 4195(1) | 27(1) |
| C(25) | 6996(4) | 2984(2) | -725(1) | 22(1) |
| C(26) | 7578(4) | 3000(2) | -1295(1) | 27(1) |
| C(27) | 6814(4) | 2499(2) | -1634(1) | 31(1) |
| C(28) | 6161(6) | 2643(2) | -2076(2) | 52(1) |
| C(29) | 5496(7) | 2151(3) | -2439(2) | 75(2) |
| C(30) | 7477(4) | 2350(2) | -440(1) | 26(1) |
| C(31) | 5249(4) | 2996(2) | -763(1) | 24(1) |
| C(32) | 3069(4) | 3645(2) | -930(2) | 47(1) |
| C(33) | 2510(4) | 3593(2) | 4599(1) | 29(1) |
| C(34) | 2159(4) | 3873(2) | 5146(1) | 33(1) |
| C(35A) | 2500(6) | 3391(3) | 5570(2) | 36(1) |
| C(36A) | 3575(6) | 3482(3) | 5913(2) | 42(1) |
| C(37A) | 3913(8) | 3033(4) | 6358(2) | 63(2) |
| C(35B) | 3110(40) | 3473(19) | 5541(13) | 36(4) |
| C(36B) | 2380(40) | 3129(16) | 5906(11) | 44(4) |
| C(37B) | 3180(60) | 2680(30) | 6289(17) | 70(7) |
| C(38) | 1638(4) | 2946(2) | 4500(2) | 39(1) |
| C(39) | 4232(4) | 3455(2) | 4562(1) | 31(1) |
| C(40) | 6711(5) | 3918(2) | 4621(2) | 52(1) |
| O(14) | 3978(9) | 2297(3) | 3477(2) | 122(2) |
| C(41) | 4158(10) | 1618(4) | 3475(4) | 118(3) |
| C(42) | 3156(8) | 1310(4) | 3861(4) | 122(3) |
| O(15) | 5121(5) | 6215(2) | 2943(2) | 84(1) |
| C(43A) | 3739(8) | 6270(3) | 2720(3) | 60(2) |
| C(44A) | 3399(12) | 6983(4) | 2657(3) | 97(3) |
| C(43B) | 4840(30) | 6328(12) | 2494(10) | 70(3) |
| C(44B) | 4620(50) | 6950(16) | 2261(13) | 116(6) |

**Table S33** Bond lengths [Å] and angles [°] for sh3279 (**7**).

| | |
|---|---|
| N(1)-C(4) | 1.301(5) |
| N(1)-C(2) | 1.383(5) |
| N(2)-C(16) | 1.304(4) |
| N(2)-C(14) | 1.390(4) |
| O(1)-C(1) | 1.354(4) |
| O(1)-C(24) | 1.468(4) |
| O(2)-C(1) | 1.204(4) |
| O(3)-C(3) | 1.364(4) |
| O(3)-C(4) | 1.369(5) |
| O(4A)-C(10) | 1.442(5) |
| O(4A)-C(9) | 1.467(5) |
| O(4B)-C(22) | 1.411(12) |
| O(4B)-C(21) | 1.557(13) |
| O(5)-C(26) | 1.435(4) |
| O(5)-H(14) | 0.843(14) |
| O(6)-C(31) | 1.337(4) |
| O(6)-C(32) | 1.448(4) |
| O(7)-C(31) | 1.205(4) |
| O(8)-C(13) | 1.340(4) |
| O(8)-C(12) | 1.467(4) |
| O(9)-C(13) | 1.204(4) |
| O(10)-C(15) | 1.360(4) |
| O(10)-C(16) | 1.375(4) |
| O(11)-C(34) | 1.418(5) |
| O(11)-H(29) | 0.843(14) |
| O(12)-C(39) | 1.205(4) |
| O(13)-C(39) | 1.343(4) |
| O(13)-C(40) | 1.450(5) |
| C(1)-C(2) | 1.473(5) |
| C(2)-C(3) | 1.344(5) |
| C(3)-H(3) | 0.9500 |
| C(4)-C(5) | 1.438(5) |
| C(5)-C(6) | 1.335(5) |
| C(5)-H(5) | 0.9500 |
| C(6)-C(7) | 1.445(5) |
| C(6)-H(6) | 0.9500 |
| C(7)-C(8) | 1.326(5) |
| C(7)-H(7) | 0.9500 |
| C(8)-C(9) | 1.462(5) |
| C(8)-H(8) | 0.9500 |
| C(9)-C(10) | 1.440(5) |
| C(9)-H(9A) | 1.0000 |
| C(9)-H(9B) | 0.9500 |
| C(10)-C(11) | 1.503(5) |
| C(10)-H(10A) | 1.0000 |
| C(10)-H(10B) | 0.9500 |
| C(11)-C(12) | 1.531(4) |
| C(11)-H(11A) | 0.9900 |
| C(11)-H(11B) | 0.9900 |
| C(12)-C(25) | 1.548(5) |
| C(12)-H(12) | 1.0000 |
| C(13)-C(14) | 1.476(5) |
| C(14)-C(15) | 1.345(5) |
| C(15)-H(15) | 0.9500 |
| C(16)-C(17) | 1.435(5) |
| C(17)-C(18) | 1.341(5) |
| C(17)-H(17) | 0.9500 |
| C(18)-C(19) | 1.432(5) |
| C(18)-H(18) | 0.9500 |
| C(19)-C(20) | 1.354(6) |
| C(19)-H(19) | 0.9500 |
| C(20)-C(21) | 1.428(5) |
| C(20)-H(20) | 0.9500 |
| C(21)-C(22) | 1.379(6) |
| C(21)-H(21A) | 0.9500 |
| C(21)-H(21B) | 1.0000 |
| C(22)-C(23) | 1.488(6) |
| C(22)-H(22A) | 0.9500 |
| C(22)-H(22B) | 1.0000 |
| C(23)-C(24) | 1.531(5) |
| C(23)-H(23A) | 0.9900 |
| C(23)-H(23B) | 0.9900 |
| C(24)-C(33) | 1.556(5) |
| C(24)-H(24) | 1.0000 |
| C(25)-C(31) | 1.525(4) |
| C(25)-C(30) | 1.538(5) |
| C(25)-C(26) | 1.565(5) |
| C(26)-C(27) | 1.499(5) |
| C(26)-H(26) | 1.0000 |
| C(27)-C(28) | 1.314(5) |
| C(27)-H(27) | 0.9500 |
| C(28)-C(29) | 1.489(6) |
| C(28)-H(28) | 0.9500 |
| C(29)-H(29A) | 0.9800 |
| C(29)-H(29B) | 0.9800 |
| C(29)-H(29C) | 0.9800 |
| C(30)-H(30A) | 0.9800 |
| C(30)-H(30B) | 0.9800 |
| C(30)-H(30C) | 0.9800 |
| C(32)-H(32A) | 0.9800 |
| C(32)-H(32B) | 0.9800 |
| C(32)-H(32C) | 0.9800 |
| C(33)-C(39) | 1.528(5) |
| C(33)-C(38) | 1.536(5) |
| C(33)-C(34) | 1.560(5) |
| C(34)-C(35A) | 1.502(6) |
| C(34)-C(35B) | 1.55(2) |
| C(34)-H(34A) | 1.0000 |
| C(34)-H(34B) | 1.0000 |
| C(35A)-C(36A) | 1.305(7) |
| C(35A)-H(35A) | 0.9500 |
| C(36A)-C(37A) | 1.501(7) |
| C(36A)-H(36A) | 0.9500 |
| C(37A)-H(37A) | 0.9800 |
| C(37A)-H(37B) | 0.9800 |
| C(37A)-H(37C) | 0.9800 |
| C(35B)-C(36B) | 1.34(2) |
| C(35B)-H(35B) | 0.9500 |
| C(36B)-C(37B) | 1.52(2) |
| C(36B)-H(36B) | 0.9500 |
| C(37B)-H(37D) | 0.9800 |
| C(37B)-H(37E) | 0.9800 |
| C(37B)-H(37F) | 0.9800 |
| C(38)-H(38A) | 0.9800 |
| C(38)-H(38B) | 0.9800 |
| C(38)-H(38C) | 0.9800 |
| C(40)-H(40A) | 0.9800 |
| C(40)-H(40B) | 0.9800 |
| C(40)-H(40C) | 0.9800 |
| O(14)-C(41) | 1.380(8) |
| O(14)-H(14A) | 0.864(14) |
| C(41)-C(42) | 1.469(12) |
| C(41)-H(41A) | 0.9900 |
| C(41)-H(41B) | 0.9900 |
| C(42)-H(42A) | 0.9800 |
| C(42)-H(42B) | 0.9800 |
| C(42)-H(42C) | 0.9800 |
| O(15)-C(43B) | 1.22(3) |
| O(15)-C(43A) | 1.340(7) |
| O(15)-H(15A) | 0.841(14) |
| C(43A)-C(44A) | 1.480(10) |
| C(43A)-H(43A) | 0.9900 |
| C(43A)-H(43B) | 0.9900 |
| C(44A)-H(44A) | 0.9800 |
| C(44A)-H(44B) | 0.9800 |
| C(44A)-H(44C) | 0.9800 |
| C(43B)-C(44B) | 1.41(4) |
| C(43B)-H(43C) | 0.9900 |
| C(43B)-H(43D) | 0.9900 |
| C(44B)-H(44D) | 0.9800 |
| C(44B)-H(44E) | 0.9800 |
| C(44B)-H(44F) | 0.9800 |
| | |
| C(4)-N(1)-C(2) | 104.6(3) |
| C(16)-N(2)-C(14) | 104.4(3) |
| C(1)-O(1)-C(24) | 118.4(3) |

| | |
|---|---|
| C(3)-O(3)-C(4) | 104.7(3) |
| C(10)-O(4A)-C(9) | 59.3(2) |
| C(22)-O(4B)-C(21) | 55.1(5) |
| C(26)-O(5)-H(14) | 103(3) |
| C(31)-O(6)-C(32) | 114.9(3) |
| C(13)-O(8)-C(12) | 117.1(2) |
| C(15)-O(10)-C(16) | 104.8(3) |
| C(34)-O(11)-H(29) | 109(3) |
| C(39)-O(13)-C(40) | 115.1(3) |
| O(2)-C(1)-O(1) | 125.4(3) |
| O(2)-C(1)-C(2) | 124.2(3) |
| O(1)-C(1)-C(2) | 110.4(3) |
| C(3)-C(2)-N(1) | 109.7(3) |
| C(3)-C(2)-C(1) | 130.0(3) |
| N(1)-C(2)-C(1) | 120.3(3) |
| C(2)-C(3)-O(3) | 107.9(3) |
| C(2)-C(3)-H(3) | 126.1 |
| O(3)-C(3)-H(3) | 126.1 |
| N(1)-C(4)-O(3) | 113.2(3) |
| N(1)-C(4)-C(5) | 127.7(3) |
| O(3)-C(4)-C(5) | 119.1(3) |
| C(6)-C(5)-C(4) | 126.2(4) |
| C(6)-C(5)-H(5) | 116.9 |
| C(4)-C(5)-H(5) | 116.9 |
| C(5)-C(6)-C(7) | 123.1(4) |
| C(5)-C(6)-H(6) | 118.5 |
| C(7)-C(6)-H(6) | 118.5 |
| C(8)-C(7)-C(6) | 126.3(4) |
| C(8)-C(7)-H(7) | 116.9 |
| C(6)-C(7)-H(7) | 116.9 |
| C(7)-C(8)-C(9) | 121.3(3) |
| C(7)-C(8)-H(8) | 119.3 |
| C(9)-C(8)-H(8) | 119.3 |
| C(10)-C(9)-C(8) | 124.7(3) |
| C(10)-C(9)-O(4A) | 59.5(2) |
| C(8)-C(9)-O(4A) | 119.8(3) |
| C(10)-C(9)-H(9A) | 114.0 |
| C(8)-C(9)-H(9A) | 114.0 |
| O(4A)-C(9)-H(9A) | 114.0 |
| C(10)-C(9)-H(9B) | 117.7 |
| C(8)-C(9)-H(9B) | 117.7 |
| C(9)-C(10)-O(4A) | 61.2(2) |
| C(9)-C(10)-C(11) | 124.9(3) |
| O(4A)-C(10)-C(11) | 116.4(3) |
| C(9)-C(10)-H(10A) | 114.5 |
| O(4A)-C(10)-H(10A) | 114.5 |
| C(11)-C(10)-H(10A) | 114.5 |
| C(9)-C(10)-H(10B) | 117.5 |
| C(11)-C(10)-H(10B) | 117.5 |
| C(10)-C(11)-C(12) | 113.1(3) |
| C(10)-C(11)-H(11A) | 109.0 |
| C(12)-C(11)-H(11A) | 109.0 |
| C(10)-C(11)-H(11B) | 109.0 |
| C(12)-C(11)-H(11B) | 109.0 |
| H(11A)-C(11)-H(11B) | 107.8 |
| O(8)-C(12)-C(11) | 106.9(3) |
| O(8)-C(12)-C(25) | 105.7(2) |
| C(11)-C(12)-C(25) | 115.4(3) |
| O(8)-C(12)-H(12) | 109.5 |
| C(11)-C(12)-H(12) | 109.5 |
| C(25)-C(12)-H(12) | 109.5 |
| O(9)-C(13)-O(8) | 125.7(3) |
| O(9)-C(13)-C(14) | 123.7(3) |
| O(8)-C(13)-C(14) | 110.6(3) |
| C(15)-C(14)-N(2) | 109.7(3) |
| C(15)-C(14)-C(13) | 129.7(3) |
| N(2)-C(14)-C(13) | 120.7(3) |
| C(14)-C(15)-O(10) | 108.1(3) |
| C(14)-C(15)-H(15) | 126.0 |
| O(10)-C(15)-H(15) | 126.0 |
| N(2)-C(16)-O(10) | 113.1(3) |
| N(2)-C(16)-C(17) | 129.2(3) |
| O(10)-C(16)-C(17) | 117.7(3) |
| C(18)-C(17)-C(16) | 122.6(4) |
| C(18)-C(17)-H(17) | 118.7 |

| | |
|---|---|
| C(16)-C(17)-H(17) | 118.7 |
| C(17)-C(18)-C(19) | 125.7(4) |
| C(17)-C(18)-H(18) | 117.1 |
| C(19)-C(18)-H(18) | 117.1 |
| C(20)-C(19)-C(18) | 123.7(4) |
| C(20)-C(19)-H(19) | 118.2 |
| C(18)-C(19)-H(19) | 118.2 |
| C(19)-C(20)-C(21) | 123.9(4) |
| C(19)-C(20)-H(20) | 118.1 |
| C(21)-C(20)-H(20) | 118.1 |
| C(22)-C(21)-C(20) | 125.1(4) |
| C(22)-C(21)-O(4B) | 57.1(5) |
| C(20)-C(21)-O(4B) | 118.4(7) |
| C(22)-C(21)-H(21A) | 117.5 |
| C(20)-C(21)-H(21A) | 117.5 |
| C(22)-C(21)-H(21B) | 114.6 |
| C(20)-C(21)-H(21B) | 114.6 |
| O(4B)-C(21)-H(21B) | 114.6 |
| C(21)-C(22)-O(4B) | 67.8(6) |
| C(21)-C(22)-C(23) | 127.6(4) |
| O(4B)-C(22)-C(23) | 111.3(7) |
| C(21)-C(22)-H(22A) | 116.2 |
| C(23)-C(22)-H(22A) | 116.2 |
| C(21)-C(22)-H(22B) | 113.6 |
| O(4B)-C(22)-H(22B) | 113.6 |
| C(23)-C(22)-H(22B) | 113.6 |
| C(22)-C(23)-C(24) | 113.9(3) |
| C(22)-C(23)-H(23A) | 108.8 |
| C(24)-C(23)-H(23A) | 108.8 |
| C(22)-C(23)-H(23B) | 108.8 |
| C(24)-C(23)-H(23B) | 108.8 |
| H(23A)-C(23)-H(23B) | 107.7 |
| O(1)-C(24)-C(23) | 106.3(3) |
| O(1)-C(24)-C(33) | 106.9(3) |
| C(23)-C(24)-C(33) | 115.5(3) |
| O(1)-C(24)-H(24) | 109.3 |
| C(23)-C(24)-H(24) | 109.3 |
| C(33)-C(24)-H(24) | 109.3 |
| C(31)-C(25)-C(30) | 108.4(3) |
| C(31)-C(25)-C(12) | 108.8(3) |
| C(30)-C(25)-C(12) | 111.7(3) |
| C(31)-C(25)-C(26) | 105.2(3) |
| C(30)-C(25)-C(26) | 112.6(3) |
| C(12)-C(25)-C(26) | 109.8(3) |
| O(5)-C(26)-C(27) | 109.1(3) |
| O(5)-C(26)-C(25) | 108.3(3) |
| C(27)-C(26)-C(25) | 113.5(3) |
| O(5)-C(26)-H(26) | 108.6 |
| C(27)-C(26)-H(26) | 108.6 |
| C(25)-C(26)-H(26) | 108.6 |
| C(28)-C(27)-C(26) | 123.8(4) |
| C(28)-C(27)-H(27) | 118.1 |
| C(26)-C(27)-H(27) | 118.1 |
| C(27)-C(28)-C(29) | 125.1(4) |
| C(27)-C(28)-H(28) | 117.4 |
| C(29)-C(28)-H(28) | 117.4 |
| C(28)-C(29)-H(29A) | 109.5 |
| C(28)-C(29)-H(29B) | 109.5 |
| H(29A)-C(29)-H(29B) | 109.5 |
| C(28)-C(29)-H(29C) | 109.5 |
| H(29A)-C(29)-H(29C) | 109.5 |
| H(29B)-C(29)-H(29C) | 109.5 |
| C(25)-C(30)-H(30A) | 109.5 |
| C(25)-C(30)-H(30B) | 109.5 |
| H(30A)-C(30)-H(30B) | 109.5 |
| C(25)-C(30)-H(30C) | 109.5 |
| H(30A)-C(30)-H(30C) | 109.5 |
| H(30B)-C(30)-H(30C) | 109.5 |
| O(7)-C(31)-O(6) | 123.2(3) |
| O(7)-C(31)-C(25) | 124.9(3) |
| O(6)-C(31)-C(25) | 111.9(3) |
| O(6)-C(32)-H(32A) | 109.5 |
| O(6)-C(32)-H(32B) | 109.5 |
| H(32A)-C(32)-H(32B) | 109.5 |
| O(6)-C(32)-H(32C) | 109.5 |

| | |
|---|---|
| H(32A)-C(32)-H(32C) | 109.5 |
| H(32B)-C(32)-H(32C) | 109.5 |
| C(39)-C(33)-C(38) | 108.5(3) |
| C(39)-C(33)-C(24) | 107.0(3) |
| C(38)-C(33)-C(24) | 112.8(3) |
| C(39)-C(33)-C(34) | 108.5(3) |
| C(38)-C(33)-C(34) | 111.4(3) |
| C(24)-C(33)-C(34) | 108.5(3) |
| O(11)-C(34)-C(35A) | 107.4(3) |
| O(11)-C(34)-C(35B) | 127.4(12) |
| O(11)-C(34)-C(33) | 107.0(3) |
| C(35A)-C(34)-C(33) | 113.3(3) |
| C(35B)-C(34)-C(33) | 108.1(16) |
| O(11)-C(34)-H(34A) | 109.7 |
| C(35A)-C(34)-H(34A) | 109.7 |
| C(33)-C(34)-H(34A) | 109.7 |
| O(11)-C(34)-H(34B) | 104.0 |
| C(35B)-C(34)-H(34B) | 104.0 |
| C(33)-C(34)-H(34B) | 104.0 |
| C(36A)-C(35A)-C(34) | 123.6(5) |
| C(36A)-C(35A)-H(35A) | 118.2 |
| C(34)-C(35A)-H(35A) | 118.2 |
| C(35A)-C(36A)-C(37A) | 125.7(5) |
| C(35A)-C(36A)-H(36A) | 117.2 |
| C(37A)-C(36A)-H(36A) | 117.2 |
| C(36A)-C(37A)-H(37A) | 109.5 |
| C(36A)-C(37A)-H(37B) | 109.5 |
| H(37A)-C(37A)-H(37B) | 109.5 |
| C(36A)-C(37A)-H(37C) | 109.5 |
| H(37A)-C(37A)-H(37C) | 109.5 |
| H(37B)-C(37A)-H(37C) | 109.5 |
| C(36B)-C(35B)-C(34) | 119(3) |
| C(36B)-C(35B)-H(35B) | 120.4 |
| C(34)-C(35B)-H(35B) | 120.4 |
| C(35B)-C(36B)-C(37B) | 124(3) |
| C(35B)-C(36B)-H(36B) | 118.0 |
| C(37B)-C(36B)-H(36B) | 118.0 |
| C(36B)-C(37B)-H(37D) | 109.5 |
| C(36B)-C(37B)-H(37E) | 109.5 |
| H(37D)-C(37B)-H(37E) | 109.5 |
| C(36B)-C(37B)-H(37F) | 109.5 |
| H(37D)-C(37B)-H(37F) | 109.5 |
| H(37E)-C(37B)-H(37F) | 109.5 |
| C(33)-C(38)-H(38A) | 109.5 |
| C(33)-C(38)-H(38B) | 109.5 |
| H(38A)-C(38)-H(38B) | 109.5 |
| C(33)-C(38)-H(38C) | 109.5 |
| H(38A)-C(38)-H(38C) | 109.5 |
| H(38B)-C(38)-H(38C) | 109.5 |
| O(12)-C(39)-O(13) | 122.9(3) |
| O(12)-C(39)-C(33) | 125.5(3) |
| O(13)-C(39)-C(33) | 111.5(3) |
| O(13)-C(40)-H(40A) | 109.5 |
| O(13)-C(40)-H(40B) | 109.5 |
| H(40A)-C(40)-H(40B) | 109.5 |
| O(13)-C(40)-H(40C) | 109.5 |
| H(40A)-C(40)-H(40C) | 109.5 |
| H(40B)-C(40)-H(40C) | 109.5 |
| C(41)-O(14)-H(14A) | 112(7) |
| O(14)-C(41)-C(42) | 110.6(7) |
| O(14)-C(41)-H(41A) | 109.5 |
| C(42)-C(41)-H(41A) | 109.5 |
| O(14)-C(41)-H(41B) | 109.5 |
| C(42)-C(41)-H(41B) | 109.5 |
| H(41A)-C(41)-H(41B) | 108.1 |
| C(41)-C(42)-H(42A) | 109.5 |
| C(41)-C(42)-H(42B) | 109.5 |
| H(42A)-C(42)-H(42B) | 109.5 |
| C(41)-C(42)-H(42C) | 109.5 |
| H(42A)-C(42)-H(42C) | 109.5 |
| H(42B)-C(42)-H(42C) | 109.5 |
| C(43B)-O(15)-H(15A) | 122(6) |
| C(43A)-O(15)-H(15A) | 108(5) |
| O(15)-C(43A)-C(44A) | 108.0(6) |
| O(15)-C(43A)-H(43A) | 110.1 |
| C(44A)-C(43A)-H(43A) | 110.1 |
| O(15)-C(43A)-H(43B) | 110.1 |
| C(44A)-C(43A)-H(43B) | 110.1 |
| H(43A)-C(43A)-H(43B) | 108.4 |
| C(43A)-C(44A)-H(44A) | 109.5 |
| C(43A)-C(44A)-H(44B) | 109.5 |
| H(44A)-C(44A)-H(44B) | 109.5 |
| C(43A)-C(44A)-H(44C) | 109.5 |
| H(44A)-C(44A)-H(44C) | 109.5 |
| H(44B)-C(44A)-H(44C) | 109.5 |
| O(15)-C(43B)-C(44B) | 128(2) |
| O(15)-C(43B)-H(43C) | 105.4 |
| C(44B)-C(43B)-H(43C) | 105.4 |
| O(15)-C(43B)-H(43D) | 105.4 |
| C(44B)-C(43B)-H(43D) | 105.4 |
| H(43C)-C(43B)-H(43D) | 106.0 |
| C(43B)-C(44B)-H(44D) | 109.5 |
| C(43B)-C(44B)-H(44E) | 109.5 |
| H(44D)-C(44B)-H(44E) | 109.5 |
| C(43B)-C(44B)-H(44F) | 109.5 |
| H(44D)-C(44B)-H(44F) | 109.5 |
| H(44E)-C(44B)-H(44F) | 109.5 |

**Table S34** Anisotropic displacement parameters (Å$^2$x 10$^3$) for sh3279 (**7**). The anisotropic displacement factor exponent takes the form: $-2\pi^2[ h^2 a^{*2}U^{11} + ... + 2 h k a^* b^* U^{12} ]$.

| | $U^{11}$ | $U^{22}$ | $U^{33}$ | $U^{23}$ | $U^{13}$ | $U^{12}$ |
|---|---|---|---|---|---|---|
| N(1) | 28(1) | 33(2) | 28(2) | -3(1) | 0(1) | -2(1) |
| N(2) | 23(1) | 29(2) | 25(1) | -2(1) | -1(1) | 3(1) |
| O(1) | 27(1) | 28(1) | 28(1) | -5(1) | 6(1) | -2(1) |
| O(2) | 36(1) | 29(1) | 33(1) | -5(1) | 5(1) | -2(1) |
| O(3) | 43(2) | 33(1) | 36(1) | -2(1) | 15(1) | 3(1) |
| O(4A) | 23(2) | 45(2) | 37(2) | -4(2) | 6(1) | -4(2) |
| O(4B) | 30(6) | 124(13) | 36(7) | 17(7) | 1(5) | 16(7) |
| O(5) | 27(1) | 55(2) | 32(1) | -1(1) | 10(1) | 0(1) |
| O(6) | 23(1) | 29(1) | 44(2) | 7(1) | 1(1) | 4(1) |
| O(7) | 28(1) | 31(1) | 37(1) | 7(1) | 2(1) | -3(1) |
| O(8) | 27(1) | 24(1) | 21(1) | 3(1) | 3(1) | 3(1) |
| O(9) | 50(2) | 25(1) | 32(1) | 6(1) | 8(1) | 4(1) |
| O(10) | 29(1) | 32(1) | 26(1) | 0(1) | 3(1) | -1(1) |
| O(11) | 46(2) | 35(2) | 47(2) | -3(1) | 15(1) | 5(1) |
| O(12) | 41(2) | 23(1) | 71(2) | -7(1) | 1(1) | 6(1) |
| O(13) | 29(1) | 26(1) | 49(2) | -4(1) | -4(1) | -2(1) |
| C(1) | 24(2) | 31(2) | 26(2) | 1(2) | -2(1) | 2(2) |
| C(2) | 24(2) | 33(2) | 27(2) | 0(2) | 0(1) | 2(2) |
| C(3) | 39(2) | 38(2) | 33(2) | 2(2) | 12(2) | 4(2) |
| C(4) | 28(2) | 33(2) | 34(2) | -2(2) | -1(2) | 0(2) |
| C(5) | 32(2) | 40(2) | 35(2) | -4(2) | 6(2) | -4(2) |
| C(6) | 25(2) | 37(2) | 30(2) | 0(2) | -3(2) | -2(2) |
| C(7) | 28(2) | 33(2) | 34(2) | -1(2) | 0(2) | -7(2) |
| C(8) | 28(2) | 30(2) | 28(2) | 0(2) | -2(2) | -2(2) |
| C(9) | 30(2) | 30(2) | 34(2) | 0(2) | 3(2) | -3(2) |
| C(10) | 26(2) | 33(2) | 33(2) | 4(2) | -1(2) | -3(2) |
| C(11) | 26(2) | 30(2) | 26(2) | 1(2) | -1(1) | 1(2) |
| C(12) | 23(2) | 26(2) | 22(2) | 4(2) | 5(1) | 2(1) |
| C(13) | 24(2) | 27(2) | 25(2) | 1(2) | -2(1) | 2(1) |
| C(14) | 23(2) | 25(2) | 24(2) | -2(1) | -7(1) | -2(1) |
| C(15) | 23(2) | 27(2) | 25(2) | -4(2) | 0(1) | -2(1) |
| C(16) | 20(2) | 33(2) | 26(2) | -2(2) | -2(1) | -1(1) |
| C(17) | 27(2) | 41(2) | 31(2) | 0(2) | 1(2) | 6(2) |
| C(18) | 26(2) | 48(3) | 30(2) | -6(2) | -4(2) | 2(2) |
| C(19) | 32(2) | 51(3) | 37(2) | -8(2) | 1(2) | 7(2) |
| C(20) | 31(2) | 43(2) | 36(2) | -10(2) | -6(2) | 6(2) |
| C(21) | 31(2) | 52(3) | 36(2) | -7(2) | -4(2) | 12(2) |
| C(22) | 29(2) | 54(3) | 39(2) | -6(2) | -1(2) | 9(2) |
| C(23) | 26(2) | 47(2) | 34(2) | -3(2) | 4(2) | -1(2) |
| C(24) | 26(2) | 28(2) | 27(2) | -5(2) | 5(1) | 1(1) |
| C(25) | 21(1) | 23(2) | 22(2) | 2(1) | 1(1) | 2(1) |
| C(26) | 26(2) | 29(2) | 27(2) | 4(2) | 5(1) | 4(2) |
| C(27) | 34(2) | 29(2) | 28(2) | 0(2) | 5(2) | 5(2) |
| C(28) | 80(3) | 42(3) | 33(2) | -2(2) | -14(2) | 7(2) |
| C(29) | 109(5) | 64(3) | 52(3) | -14(3) | -34(3) | 9(3) |
| C(30) | 26(2) | 26(2) | 27(2) | 4(2) | 0(2) | 4(1) |
| C(31) | 26(2) | 26(2) | 20(2) | 2(1) | 0(1) | 3(1) |
| C(32) | 23(2) | 47(2) | 73(3) | 18(2) | 1(2) | 11(2) |
| C(33) | 33(2) | 21(2) | 34(2) | -2(2) | 3(2) | -2(2) |
| C(34) | 43(2) | 23(2) | 35(2) | 0(2) | 4(2) | 0(2) |
| C(35A) | 42(3) | 32(3) | 36(2) | 5(2) | 15(2) | 0(2) |
| C(36A) | 46(3) | 47(3) | 34(2) | 3(2) | 11(2) | 11(2) |
| C(37A) | 68(4) | 78(5) | 43(3) | 17(3) | 10(3) | 30(4) |
| C(35B) | 41(7) | 33(7) | 33(6) | 1(6) | 13(7) | -2(7) |
| C(36B) | 49(7) | 49(7) | 35(6) | 8(6) | 12(6) | 9(6) |
| C(37B) | 82(14) | 85(14) | 44(11) | 21(11) | 7(12) | 35(12) |
| C(38) | 38(2) | 30(2) | 49(2) | -7(2) | 7(2) | -7(2) |
| C(39) | 35(2) | 22(2) | 35(2) | 1(2) | -4(2) | -1(2) |
| C(40) | 31(2) | 43(3) | 81(3) | -5(2) | -7(2) | -1(2) |
| O(14) | 189(6) | 73(3) | 103(4) | -10(3) | 7(4) | -9(4) |
| C(41) | 121(6) | 79(5) | 156(8) | -39(5) | -32(6) | 1(4) |
| C(42) | 81(5) | 68(4) | 218(10) | 20(5) | -43(5) | -1(4) |
| O(15) | 99(3) | 30(2) | 122(3) | -11(2) | -61(3) | -1(2) |
| C(43A) | 59(4) | 64(4) | 57(4) | -10(3) | -14(3) | -10(3) |
| C(44A) | 133(8) | 80(5) | 78(5) | -6(5) | -62(5) | 45(5) |
| C(43B) | 64(7) | 65(6) | 81(6) | -9(6) | -12(6) | -8(7) |
| C(44B) | 158(15) | 89(10) | 101(12) | 1(9) | -42(12) | 41(11) |

**Table S35** Hydrogen coordinates (x $10^4$) and isotropic displacement parameters ($Å^2$ x $10^3$) for **7**.

| | x | y | z | U(eq) |
|---|---|---|---|---|
| H(14) | 9520(50) | 3130(20) | -1534(13) | 58 |
| H(29) | 430(60) | 4371(14) | 5321(18) | 64 |
| H(3) | 4796 | 3289 | 3087 | 44 |
| H(5) | 7466 | 5103 | 2033 | 43 |
| H(6) | 7859 | 3799 | 1717 | 37 |
| H(7) | 8895 | 5005 | 1249 | 38 |
| H(8) | 9233 | 3736 | 853 | 34 |
| H(9A) | 9768 | 5038 | 409 | 38 |
| H(9B) | 10296 | 4969 | 440 | 38 |
| H(10A) | 9688 | 4625 | -399 | 37 |
| H(10B) | 10177 | 4557 | -393 | 37 |
| H(11A) | 9330 | 3311 | 33 | 33 |
| H(11B) | 9863 | 3429 | -548 | 33 |
| H(12) | 7343 | 4010 | -658 | 28 |
| H(15) | 5448 | 3269 | 933 | 30 |
| H(17) | 2570 | 5350 | 1546 | 39 |
| H(18) | 2521 | 4175 | 2109 | 41 |
| H(19) | 1028 | 5392 | 2362 | 48 |
| H(20) | 1152 | 4214 | 2928 | 44 |
| H(21A) | -281 | 5433 | 3201 | 47 |
| H(21B) | 163 | 5507 | 3247 | 47 |
| H(22A) | -507 | 5079 | 4013 | 49 |
| H(22B) | -54 | 5148 | 4041 | 49 |
| H(23A) | 397 | 3799 | 3718 | 43 |
| H(23B) | -215 | 4012 | 4274 | 43 |
| H(24) | 2379 | 4582 | 4344 | 32 |
| H(26) | 7383 | 3450 | -1439 | 33 |
| H(27) | 6800 | 2051 | -1523 | 37 |
| H(28) | 6110 | 3096 | -2171 | 62 |
| H(29A) | 5080 | 2382 | -2740 | 112 |
| H(29B) | 6300 | 1842 | -2549 | 112 |
| H(29C) | 4672 | 1906 | -2268 | 112 |
| H(30A) | 7047 | 1964 | -617 | 40 |
| H(30B) | 7089 | 2364 | -87 | 40 |
| H(30C) | 8600 | 2318 | -436 | 40 |
| H(32A) | 2787 | 4083 | -1059 | 71 |
| H(32B) | 2639 | 3584 | -584 | 71 |
| H(32C) | 2658 | 3305 | -1160 | 71 |
| H(34A) | 2754 | 4290 | 5202 | 40 |
| H(34B) | 2652 | 4320 | 5145 | 40 |
| H(35A) | 1902 | 3000 | 5590 | 44 |
| H(36A) | 4196 | 3865 | 5878 | 51 |
| H(37A) | 3752 | 3273 | 6681 | 94 |
| H(37B) | 4982 | 2883 | 6339 | 94 |
| H(37C) | 3226 | 2649 | 6346 | 94 |
| H(35B) | 4203 | 3472 | 5525 | 43 |
| H(36B) | 1299 | 3169 | 5927 | 53 |
| H(37D) | 2570 | 2278 | 6339 | 105 |
| H(37E) | 3297 | 2910 | 6618 | 105 |
| H(37F) | 4201 | 2559 | 6157 | 105 |
| H(38A) | 2021 | 2602 | 4733 | 59 |
| H(38B) | 1798 | 2807 | 4143 | 59 |
| H(38C) | 539 | 3015 | 4561 | 59 |
| H(40A) | 7218 | 4340 | 4697 | 78 |
| H(40B) | 6942 | 3786 | 4267 | 78 |
| H(40C) | 7086 | 3578 | 4859 | 78 |
| H(14A) | 4210(120) | 2470(50) | 3771(19) | 183 |
| H(41A) | 5242 | 1507 | 3551 | 142 |
| H(41B) | 3904 | 1442 | 3130 | 142 |
| H(42A) | 3481 | 852 | 3918 | 183 |
| H(42B) | 2092 | 1316 | 3738 | 183 |
| H(42C) | 3227 | 1557 | 4185 | 183 |
| H(15A) | 5210(80) | 5828(16) | 3060(30) | 126 |
| H(43A) | 2944 | 6059 | 2938 | 72 |
| H(43B) | 3744 | 6048 | 2381 | 72 |
| H(44A) | 2392 | 7036 | 2494 | 145 |
| H(44B) | 3392 | 7198 | 2995 | 145 |
| H(44C) | 4189 | 7187 | 2440 | 145 |
| H(43C) | 3898 | 6073 | 2416 | 84 |
| H(43D) | 5677 | 6115 | 2297 | 84 |
| H(44D) | 4411 | 6889 | 1894 | 174 |
| H(44E) | 3751 | 7175 | 2423 | 174 |
| H(44F) | 5551 | 7217 | 2304 | 174 |

**Table S36** Torsion angles [°] for sh3279 (**7**).

| | | | |
|---|---|---|---|
| C(24)-O(1)-C(1)-O(2) | -1.5(5) | C(21)-O(4B)-C(22)-C(23) | -123.3(5) |
| C(24)-O(1)-C(1)-C(2) | 178.9(3) | C(21)-C(22)-C(23)-C(24) | 88.4(5) |
| C(4)-N(1)-C(2)-C(3) | 0.4(4) | O(4B)-C(22)-C(23)-C(24) | 166.2(6) |
| C(4)-N(1)-C(2)-C(1) | -179.2(3) | C(1)-O(1)-C(24)-C(23) | 108.7(3) |
| O(2)-C(1)-C(2)-C(3) | 169.8(4) | C(1)-O(1)-C(24)-C(33) | -127.4(3) |
| O(1)-C(1)-C(2)-C(3) | -10.6(5) | C(22)-C(23)-C(24)-O(1) | -84.4(4) |
| O(2)-C(1)-C(2)-N(1) | -10.8(5) | C(22)-C(23)-C(24)-C(33) | 157.3(3) |
| O(1)-C(1)-C(2)-N(1) | 168.8(3) | O(8)-C(12)-C(25)-C(31) | 49.6(3) |
| N(1)-C(2)-C(3)-O(3) | -0.1(4) | C(11)-C(12)-C(25)-C(31) | 167.5(3) |
| C(1)-C(2)-C(3)-O(3) | 179.4(3) | O(8)-C(12)-C(25)-C(30) | -70.1(3) |
| C(4)-O(3)-C(3)-C(2) | -0.3(4) | C(11)-C(12)-C(25)-C(30) | 47.8(4) |
| C(2)-N(1)-C(4)-O(3) | -0.6(4) | O(8)-C(12)-C(25)-C(26) | 164.2(2) |
| C(2)-N(1)-C(4)-C(5) | 176.9(4) | C(11)-C(12)-C(25)-C(26) | -77.9(3) |
| C(3)-O(3)-C(4)-N(1) | 0.6(4) | C(31)-C(25)-C(26)-O(5) | -170.8(3) |
| C(3)-O(3)-C(4)-C(5) | -177.2(3) | C(30)-C(25)-C(26)-O(5) | -52.9(4) |
| N(1)-C(4)-C(5)-C(6) | -176.9(4) | C(12)-C(25)-C(26)-O(5) | 72.2(3) |
| O(3)-C(4)-C(5)-C(6) | 0.4(6) | C(31)-C(25)-C(26)-C(27) | -49.5(4) |
| C(4)-C(5)-C(6)-C(7) | 174.9(3) | C(30)-C(25)-C(26)-C(27) | 68.4(4) |
| C(5)-C(6)-C(7)-C(8) | -172.8(4) | C(12)-C(25)-C(26)-C(27) | -166.5(3) |
| C(6)-C(7)-C(8)-C(9) | 171.4(3) | O(5)-C(26)-C(27)-C(28) | -111.1(4) |
| C(7)-C(8)-C(9)-C(10) | -151.5(4) | C(25)-C(26)-C(27)-C(28) | 128.0(4) |
| C(7)-C(8)-C(9)-O(4A) | 136.9(4) | C(26)-C(27)-C(28)-C(29) | 175.2(5) |
| C(10)-O(4A)-C(9)-C(8) | 115.1(4) | C(32)-O(6)-C(31)-O(7) | 3.7(5) |
| C(8)-C(9)-C(10)-O(4A) | -107.0(4) | C(32)-O(6)-C(31)-C(25) | -177.5(3) |
| C(8)-C(9)-C(10)-C(11) | -3.4(6) | C(30)-C(25)-C(31)-O(7) | -14.3(5) |
| O(4A)-C(9)-C(10)-C(11) | 103.6(4) | C(12)-C(25)-C(31)-O(7) | -136.0(3) |
| C(9)-O(4A)-C(10)-C(11) | -117.2(4) | C(26)-C(25)-C(31)-O(7) | 106.4(4) |
| C(9)-C(10)-C(11)-C(12) | 95.3(4) | C(30)-C(25)-C(31)-O(6) | 166.9(3) |
| O(4A)-C(10)-C(11)-C(12) | 167.3(3) | C(12)-C(25)-C(31)-O(6) | 45.2(4) |
| C(13)-O(8)-C(12)-C(11) | 99.7(3) | C(26)-C(25)-C(31)-O(6) | -72.4(3) |
| C(13)-O(8)-C(12)-C(25) | -136.8(3) | O(1)-C(24)-C(33)-C(39) | 35.5(4) |
| C(10)-C(11)-C(12)-O(8) | -78.4(3) | C(23)-C(24)-C(33)-C(39) | 153.5(3) |
| C(10)-C(11)-C(12)-C(25) | 164.4(3) | O(1)-C(24)-C(33)-C(38) | -83.7(3) |
| C(12)-O(8)-C(13)-O(9) | 0.7(5) | C(23)-C(24)-C(33)-C(38) | 34.3(4) |
| C(12)-O(8)-C(13)-C(14) | -178.1(3) | O(1)-C(24)-C(33)-C(34) | 152.4(3) |
| C(16)-N(2)-C(14)-C(15) | -0.7(4) | C(23)-C(24)-C(33)-C(34) | -89.6(4) |
| C(16)-N(2)-C(14)-C(13) | -179.5(3) | C(39)-C(33)-C(34)-O(11) | 178.5(3) |
| O(9)-C(13)-C(14)-C(15) | -171.9(3) | C(38)-C(33)-C(34)-O(11) | -62.1(4) |
| O(8)-C(13)-C(14)-C(15) | 6.9(5) | C(24)-C(33)-C(34)-O(11) | 62.6(4) |
| O(9)-C(13)-C(14)-N(2) | 6.7(5) | C(39)-C(33)-C(34)-C(35A) | -63.2(4) |
| O(8)-C(13)-C(14)-N(2) | -174.5(3) | C(38)-C(33)-C(34)-C(35A) | 56.2(5) |
| N(2)-C(14)-C(15)-O(10) | 0.4(4) | C(24)-C(33)-C(34)-C(35A) | -179.1(3) |
| C(13)-C(14)-C(15)-O(10) | 179.2(3) | C(39)-C(33)-C(34)-C(35B) | -41.3(14) |
| C(16)-O(10)-C(15)-C(14) | 0.0(3) | C(38)-C(33)-C(34)-C(35B) | 78.1(14) |
| C(14)-N(2)-C(16)-O(10) | 0.7(4) | C(24)-C(33)-C(34)-C(35B) | -157.2(14) |
| C(14)-N(2)-C(16)-C(17) | 180.0(3) | O(11)-C(34)-C(35A)-C(36A) | -127.8(5) |
| C(15)-O(10)-C(16)-N(2) | -0.4(4) | C(33)-C(34)-C(35A)-C(36A) | 114.2(5) |
| C(15)-O(10)-C(16)-C(17) | -179.8(3) | C(34)-C(35A)-C(36A)-C(37A) | 177.0(5) |
| N(2)-C(16)-C(17)-C(18) | -179.3(3) | O(11)-C(34)-C(35B)-C(36B) | 11(5) |
| O(10)-C(16)-C(17)-C(18) | 0.0(5) | C(33)-C(34)-C(35B)-C(36B) | -119(3) |
| C(16)-C(17)-C(18)-C(19) | 178.6(3) | C(34)-C(35B)-C(36B)-C(37B) | 175(4) |
| C(17)-C(18)-C(19)-C(20) | -174.9(4) | C(40)-O(13)-C(39)-O(12) | 2.2(6) |
| C(18)-C(19)-C(20)-C(21) | 172.9(4) | C(40)-O(13)-C(39)-C(33) | -175.8(3) |
| C(19)-C(20)-C(21)-C(22) | -165.7(4) | C(38)-C(33)-C(39)-O(12) | 6.6(5) |
| C(19)-C(20)-C(21)-O(4B) | 126.4(7) | C(24)-C(33)-C(39)-O(12) | -115.4(4) |
| C(22)-O(4B)-C(21)-C(20) | 115.4(5) | C(34)-C(33)-C(39)-O(12) | 127.8(4) |
| C(20)-C(21)-C(22)-O(4B) | -103.7(8) | C(38)-C(33)-C(39)-O(13) | -175.5(3) |
| C(20)-C(21)-C(22)-C(23) | -3.3(7) | C(24)-C(33)-C(39)-O(13) | 62.6(4) |
| O(4B)-C(21)-C(22)-C(23) | 100.4(8) | C(34)-C(33)-C(39)-O(13) | -54.3(4) |

**Table S37** Hydrogen bonds for sh3279 (**7**) [Å and °].

| D-H...A | d(D-H) | d(H...A) | d(D...A) | <(DHA) |
|---|---|---|---|---|
| O(11)-H(29)...N(2)#1 | 0.843(14) | 2.218(19) | 3.042(4) | 166(5) |
| O(5)-H(14)...O(15)#2 | 0.843(14) | 1.928(17) | 2.760(5) | 169(5) |
| O(14)-H(14A)...O(12) | 0.864(14) | 2.023(17) | 2.885(6) | 175(10) |
| O(15)-H(15A)...N(1) | 0.841(14) | 2.28(5) | 3.004(5) | 144(7) |

Symmetry transformations used to generate equivalent atoms:

#1 -x+1/2,-y+1,z+1/2 #2 -x+3/2,-y+1,z-1/2

## 3. Supplemental figures

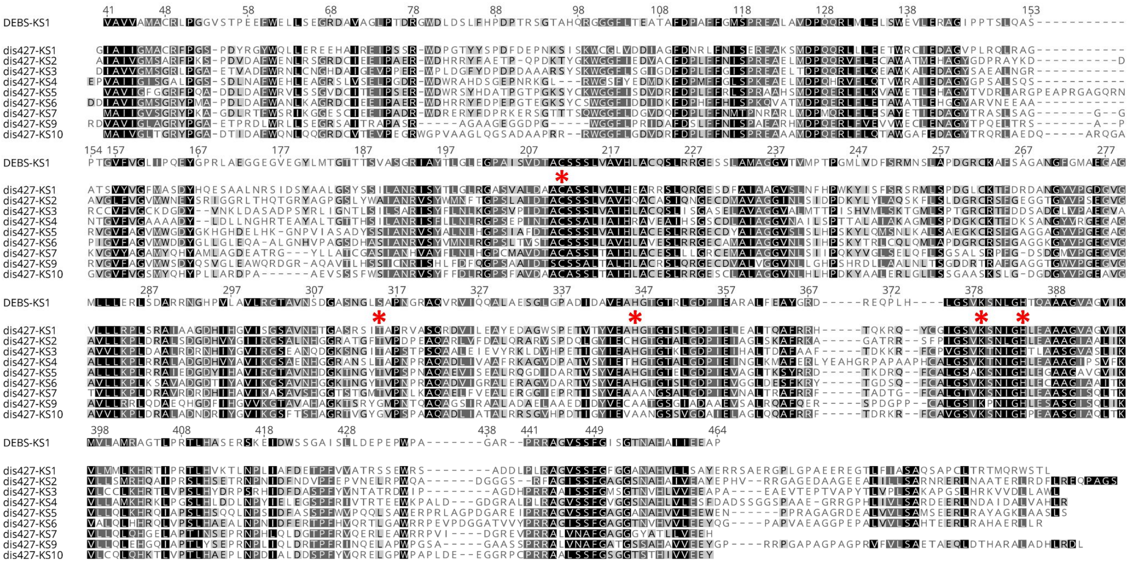

**Figure S7** The aligned KS domains. DEBS-KS1 is the first KS domain of 6-deoxyerythronolide B synthase.(6) dis427-KS with corresponding module numbers are KS domains from the *S. cellulosum* So ce427 disorazole Z biosynthetic pathway. The conserved active site are marked by red asterisks.

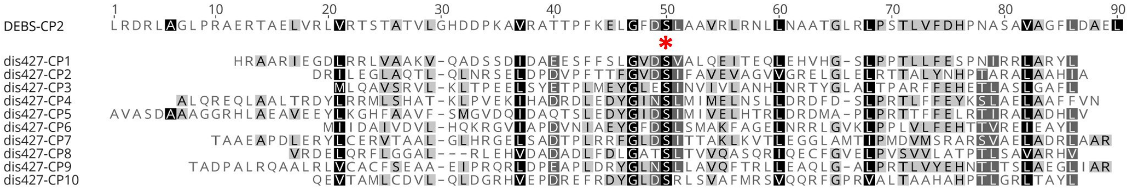

**Figure S8** The aligned CP domains. DEBS-CP2 is the CP domain from the second module of 6-deoxyerythronolide B synthase.(7) dis427-CP with corresponding module numbers are CP domains from the *S. cellulosum* So ce427 disorazole Z biosynthetic pathway. The serine for phosphopantetheinyl arm attachment is marked by red asterisk.

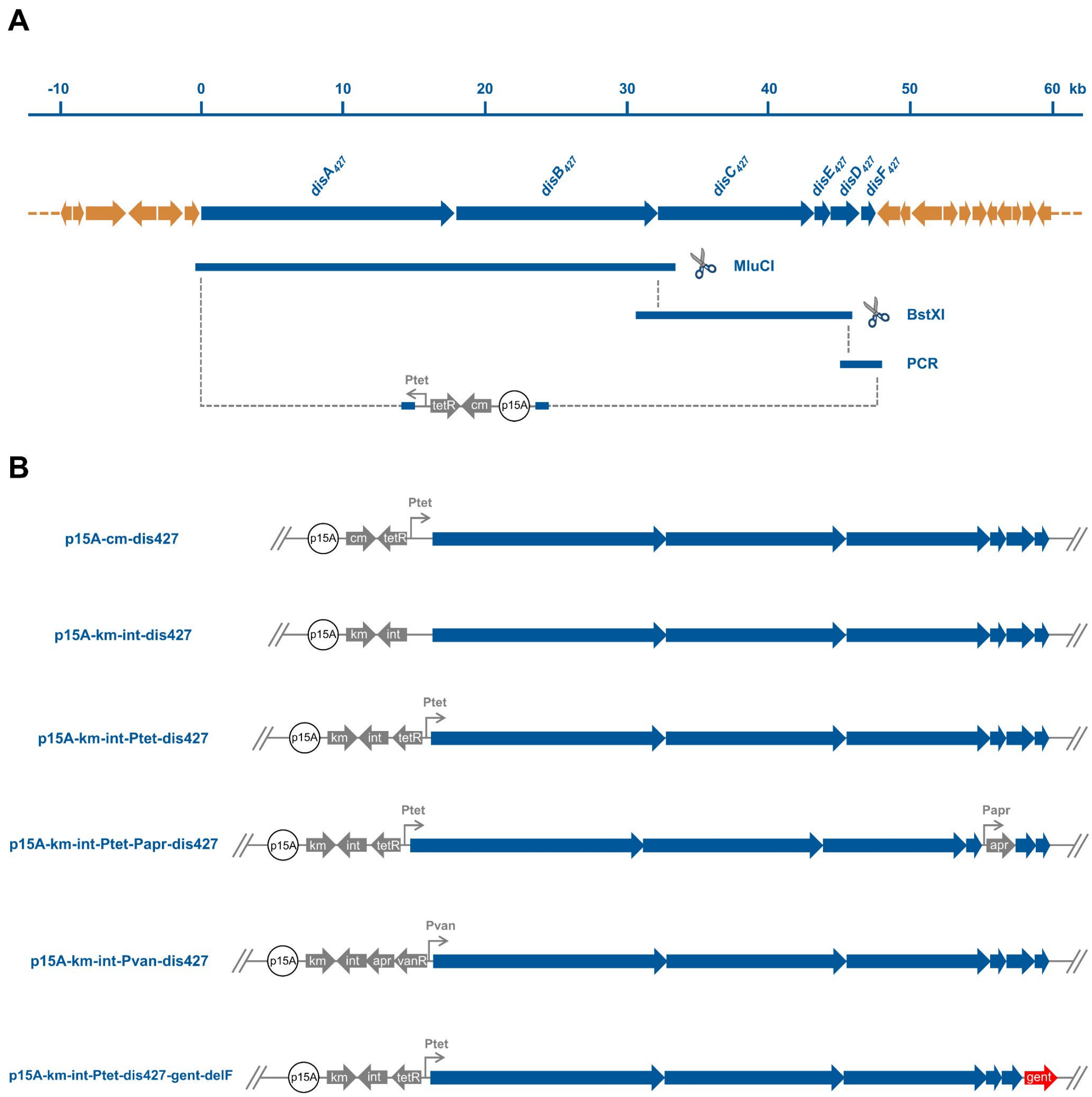


**Figure S9** Cloning and engineering of the *dis427* gene cluster for heterologous expression. A: the disorazole Z biosynthesis gene cluster was cloned from the genomic DNA of *S. cellulosum* So ce427 using LLHR as described in methods section. B: scheme of plasmids containing *dis427* gene cluster generated in this study, the expression constructs are created using LCHR as described in methods section.

**A**

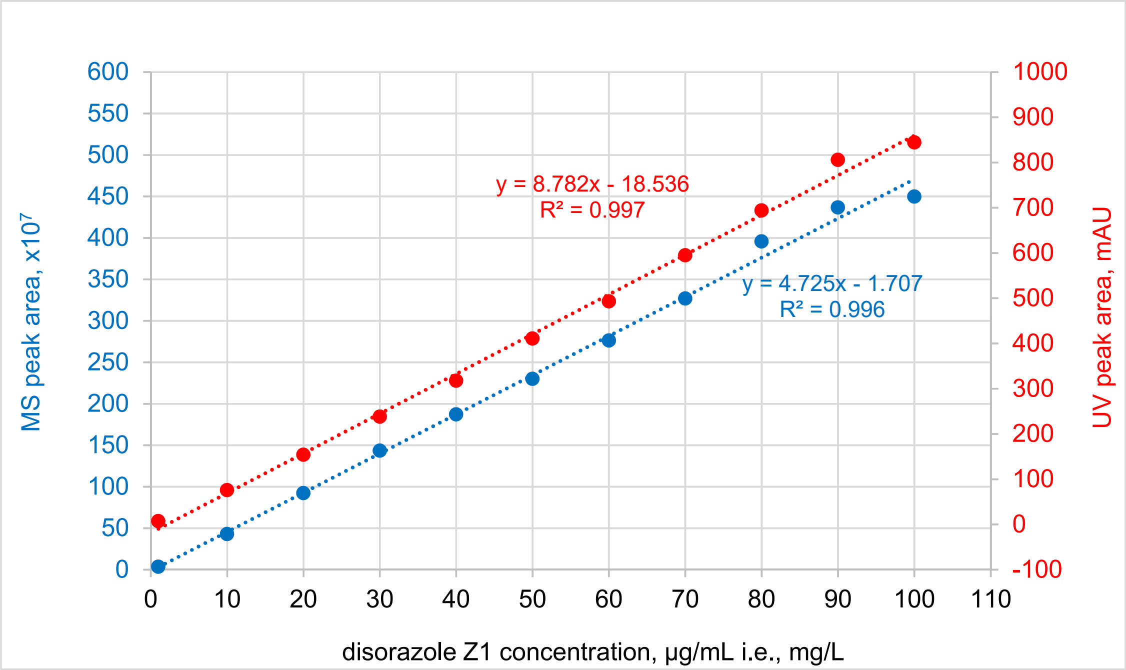


**B**

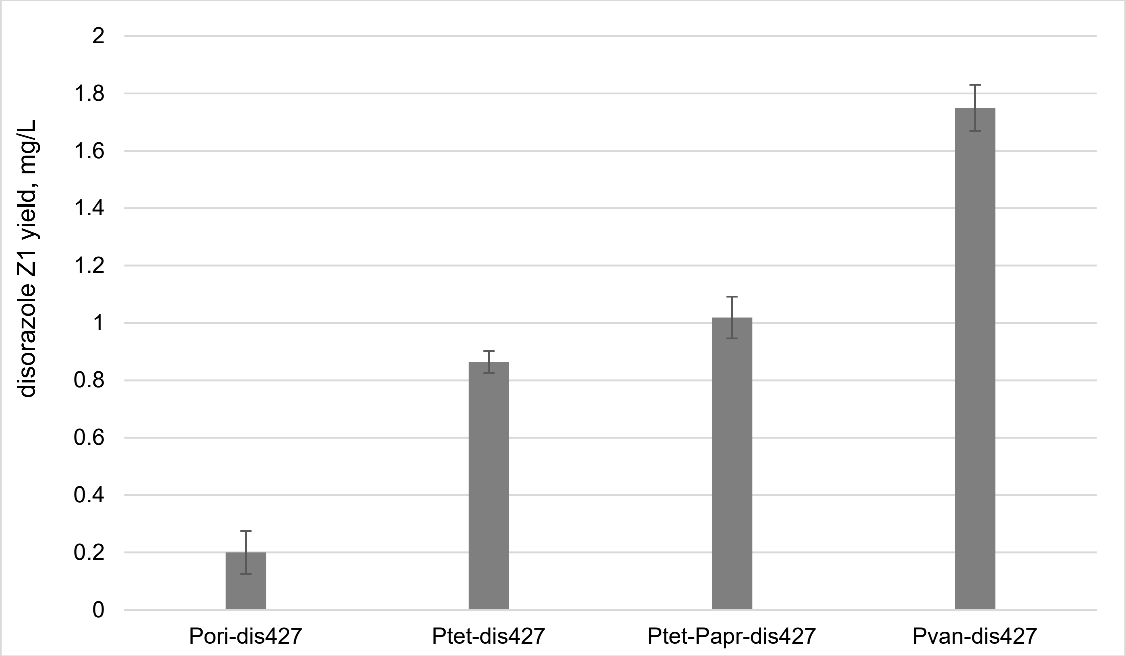


**Figure S10** Quantitative analysis of disorazole Z1 (**3**) using HPLC-UV-MS. A: the standard curve of **3**. B: heterologous production yields of **3** in *M. xanthus* DK1622 before and after promoter engineering.

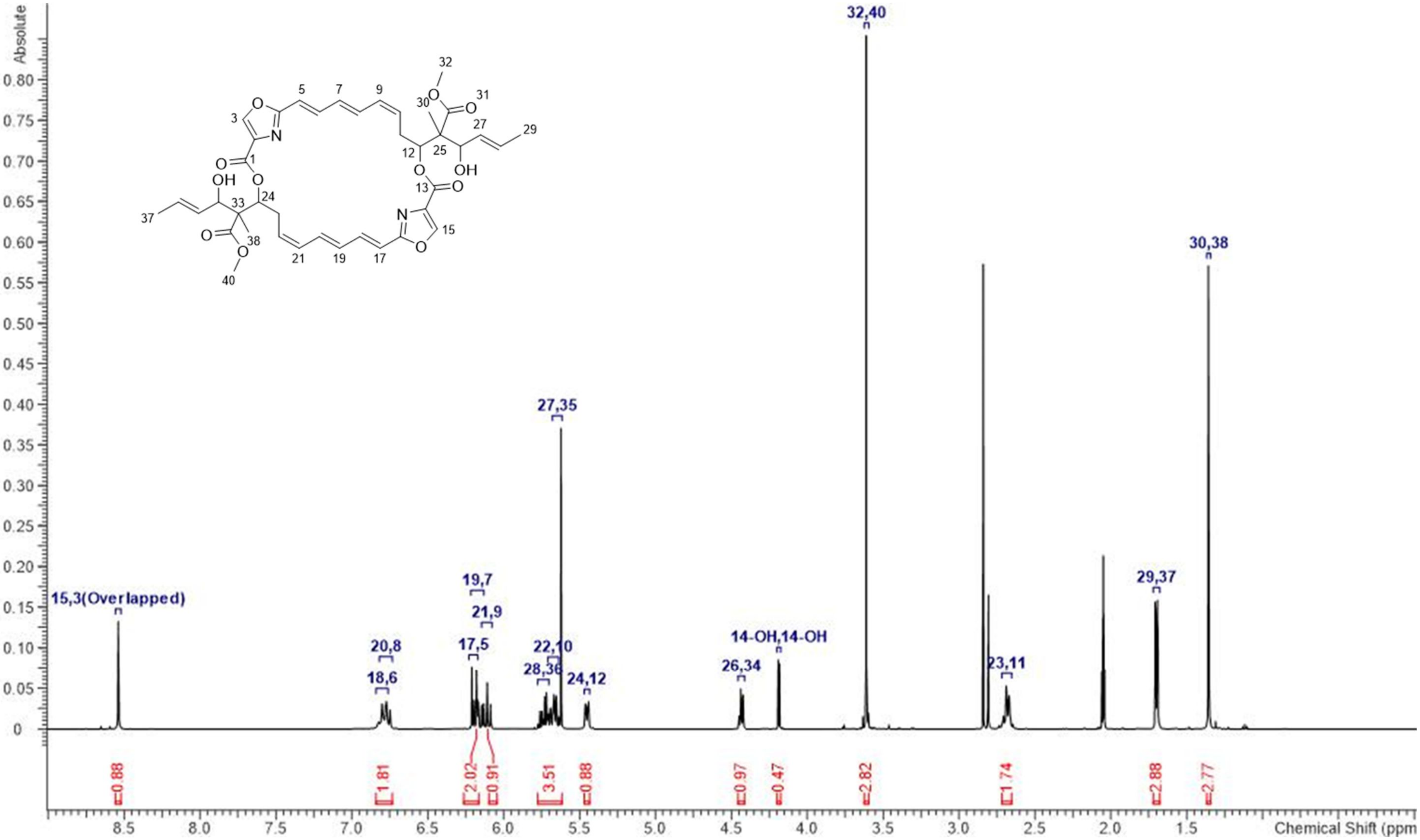


**Figure S11** $^{1}$H NMR spectrum of disorazole Z1 (**3**) in acetone-$d_6$ (500 MHz).

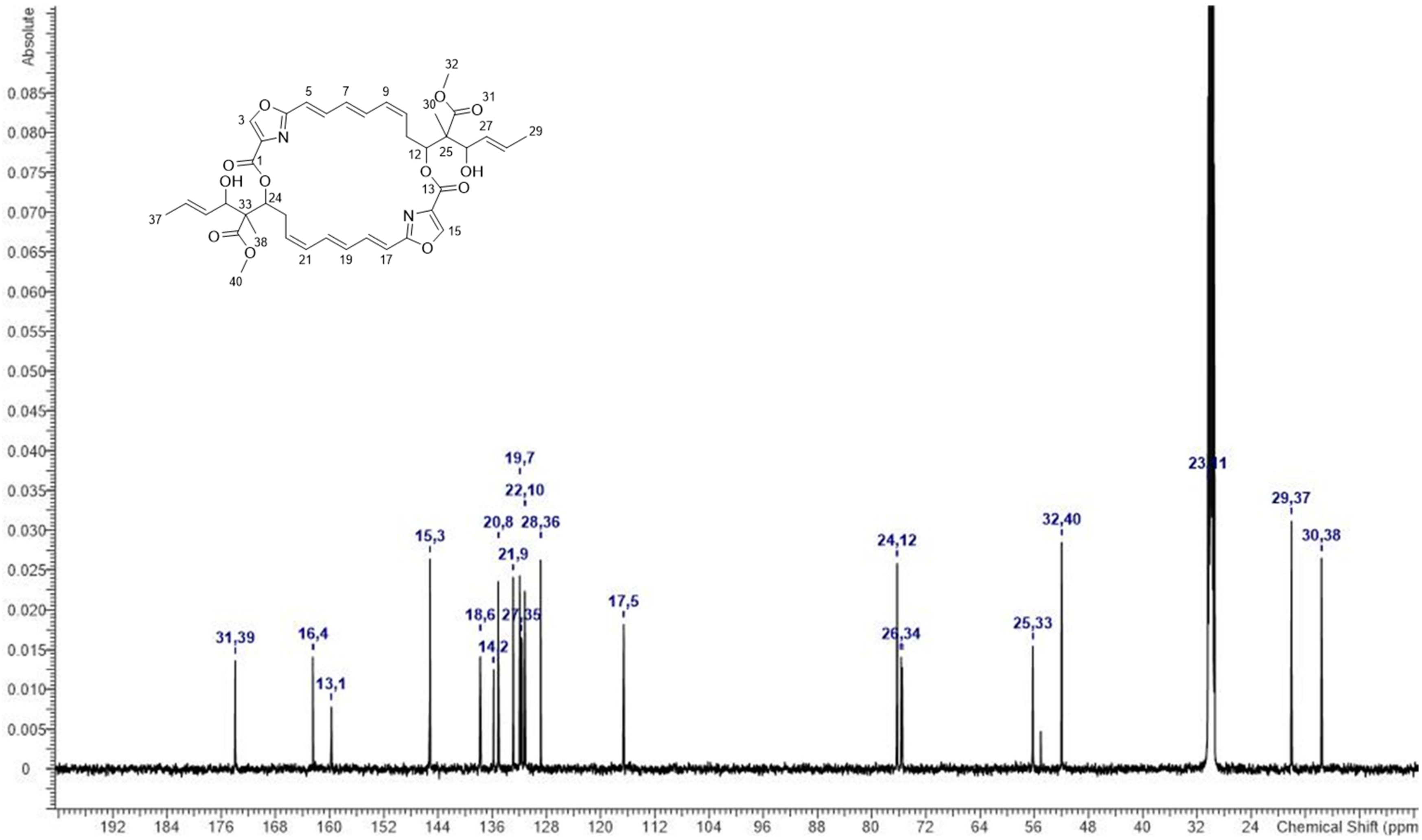


**Figure S12** $^{13}C$ NMR spectrum of disorazole Z1 (**3**) in acetone-$d_6$ (100 MHz).

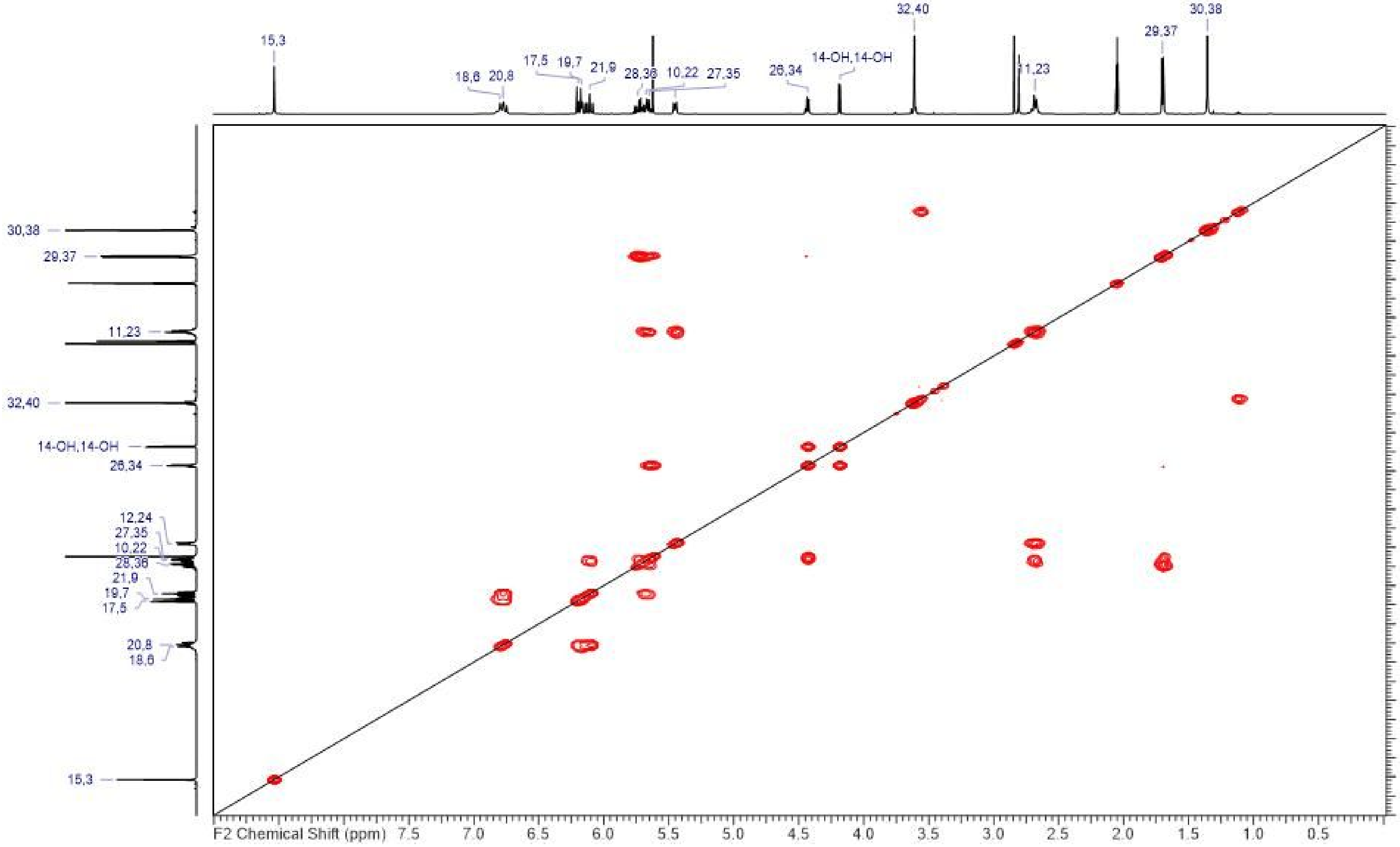


**Figure S13** $^{1}$H,$^{1}$H-COSY NMR spectrum of disorazole Z1 (**3**) in acetone-$d_6$.

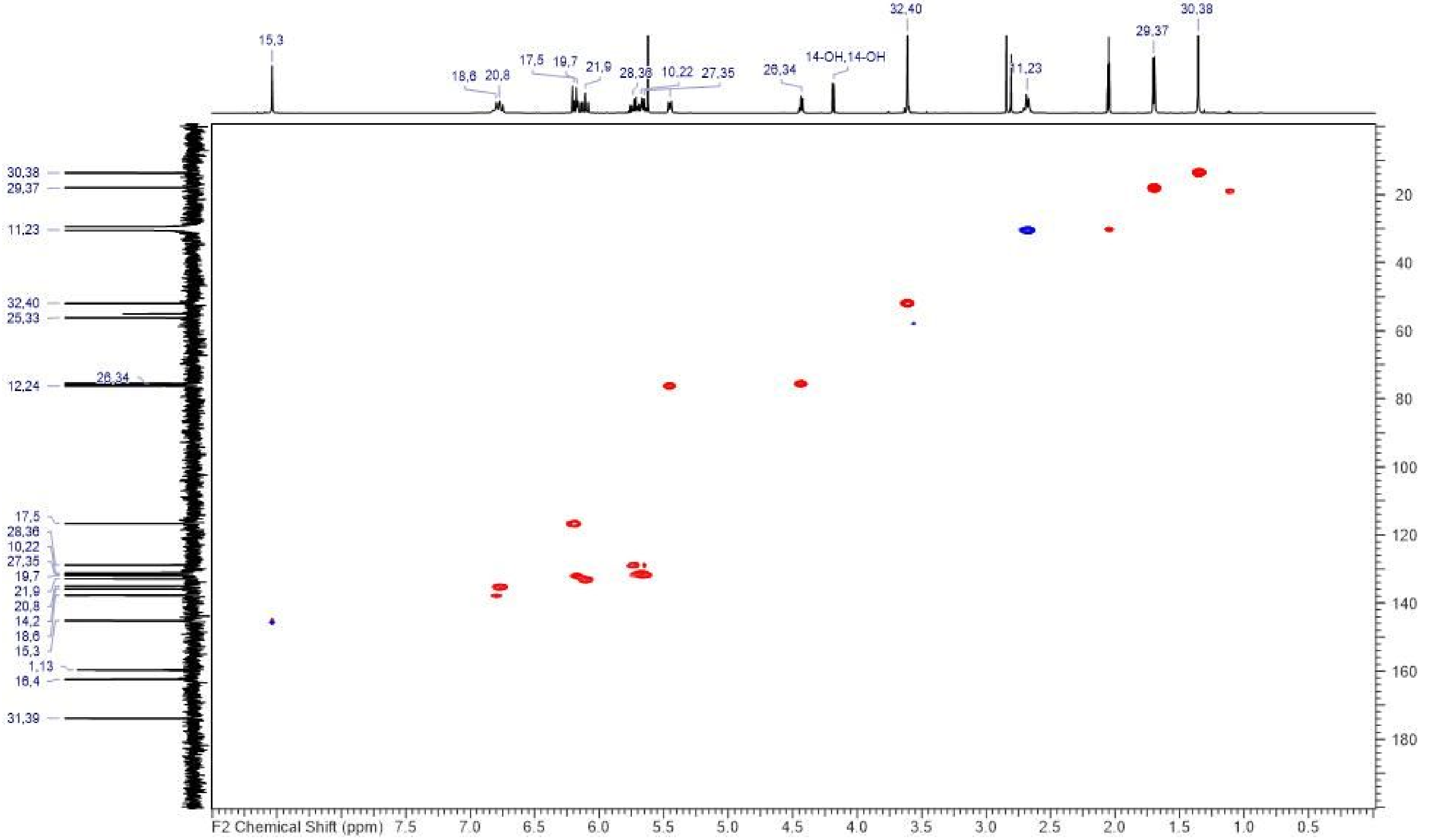


**Figure S14** HSQC-NMR spectrum of disorazole Z1 (**3**) in acetone-$d_6$.

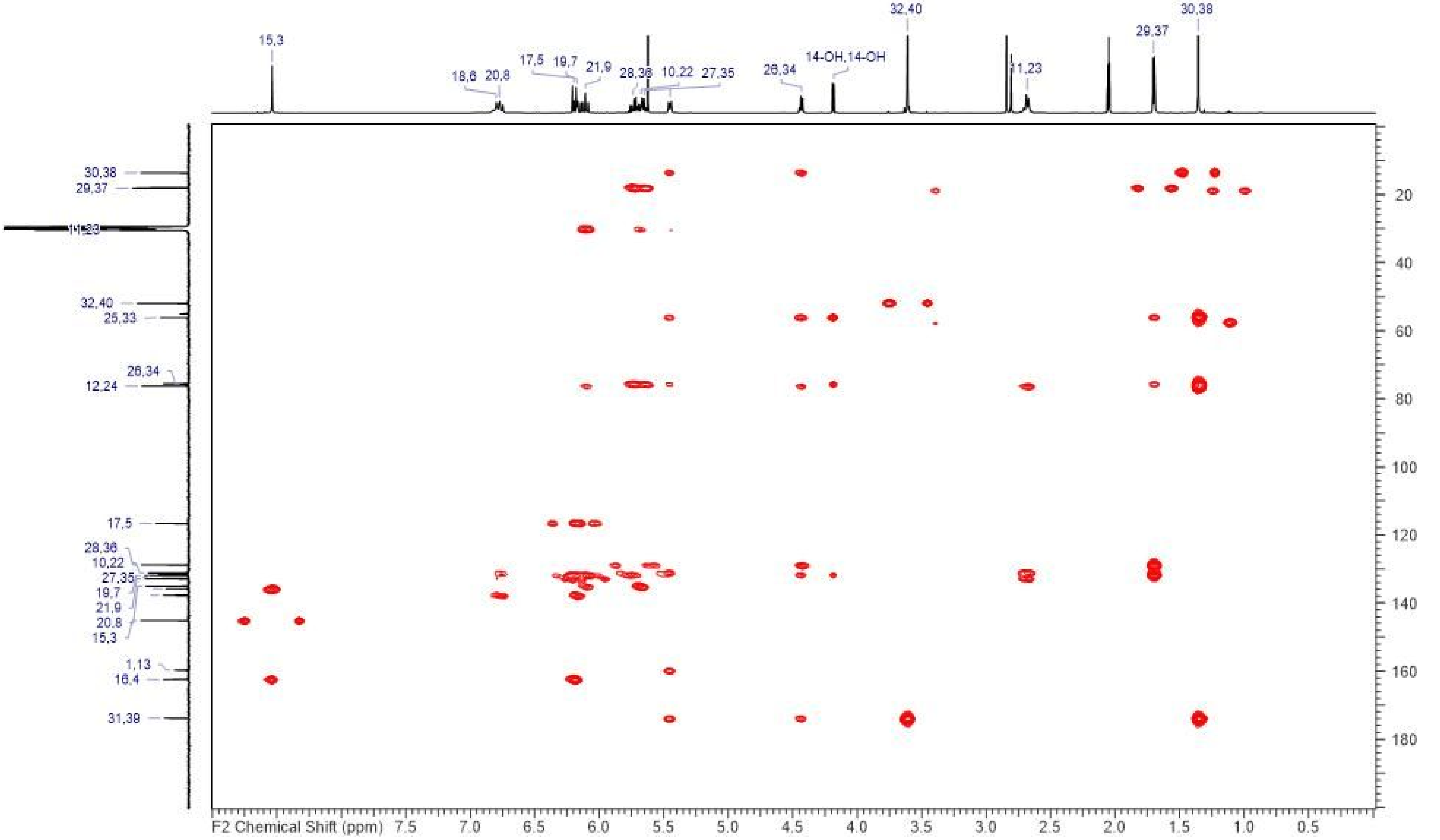


**Figure S15** HMBC NMR spectrum of disorazole Z1 (**3**) in acetone-$d_6$.

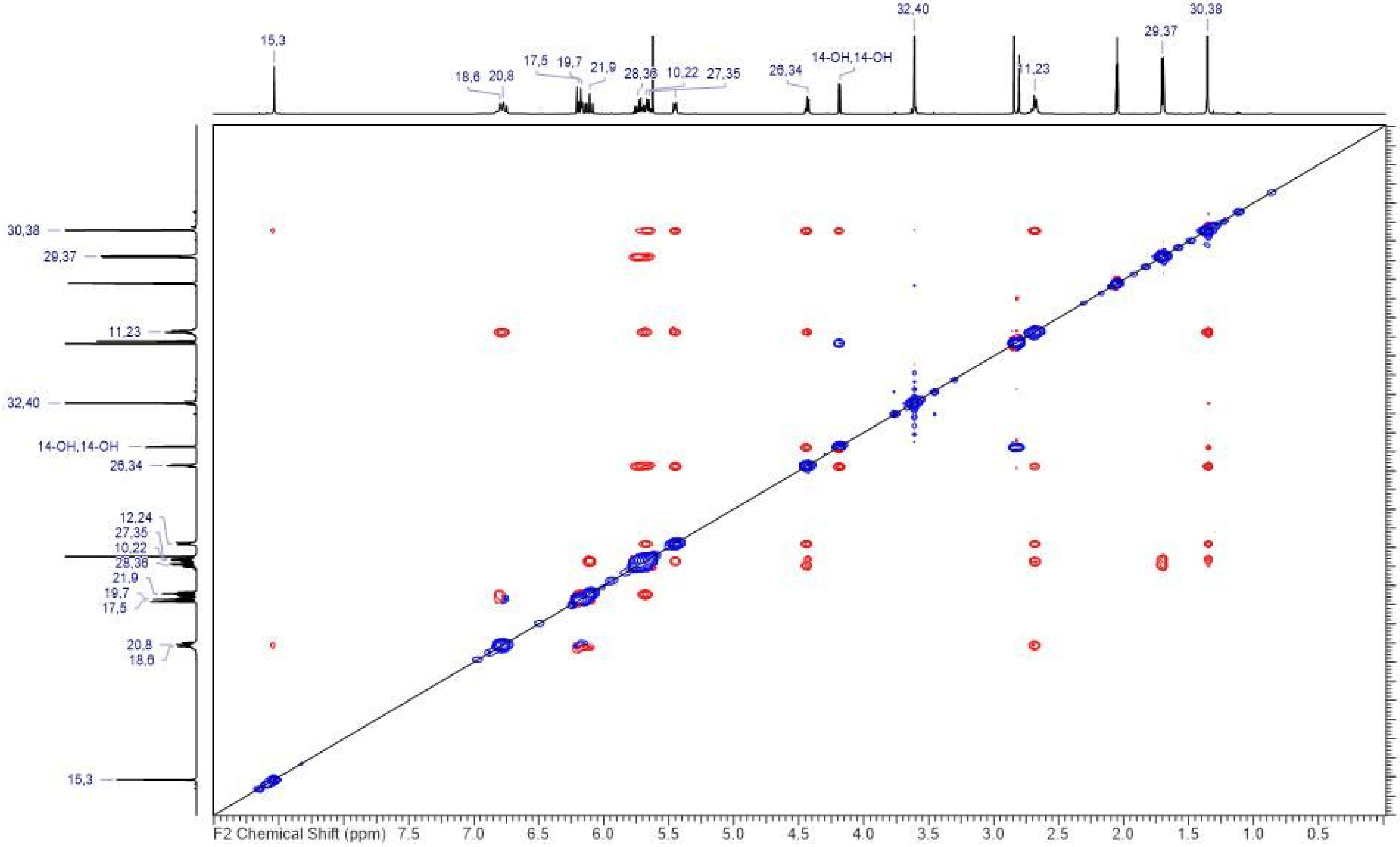


**Figure S16** $^{1}H$,$^{1}H$-ROESY NMR spectrum of disorazole Z1 (**3**) in acetone-$d_6$.

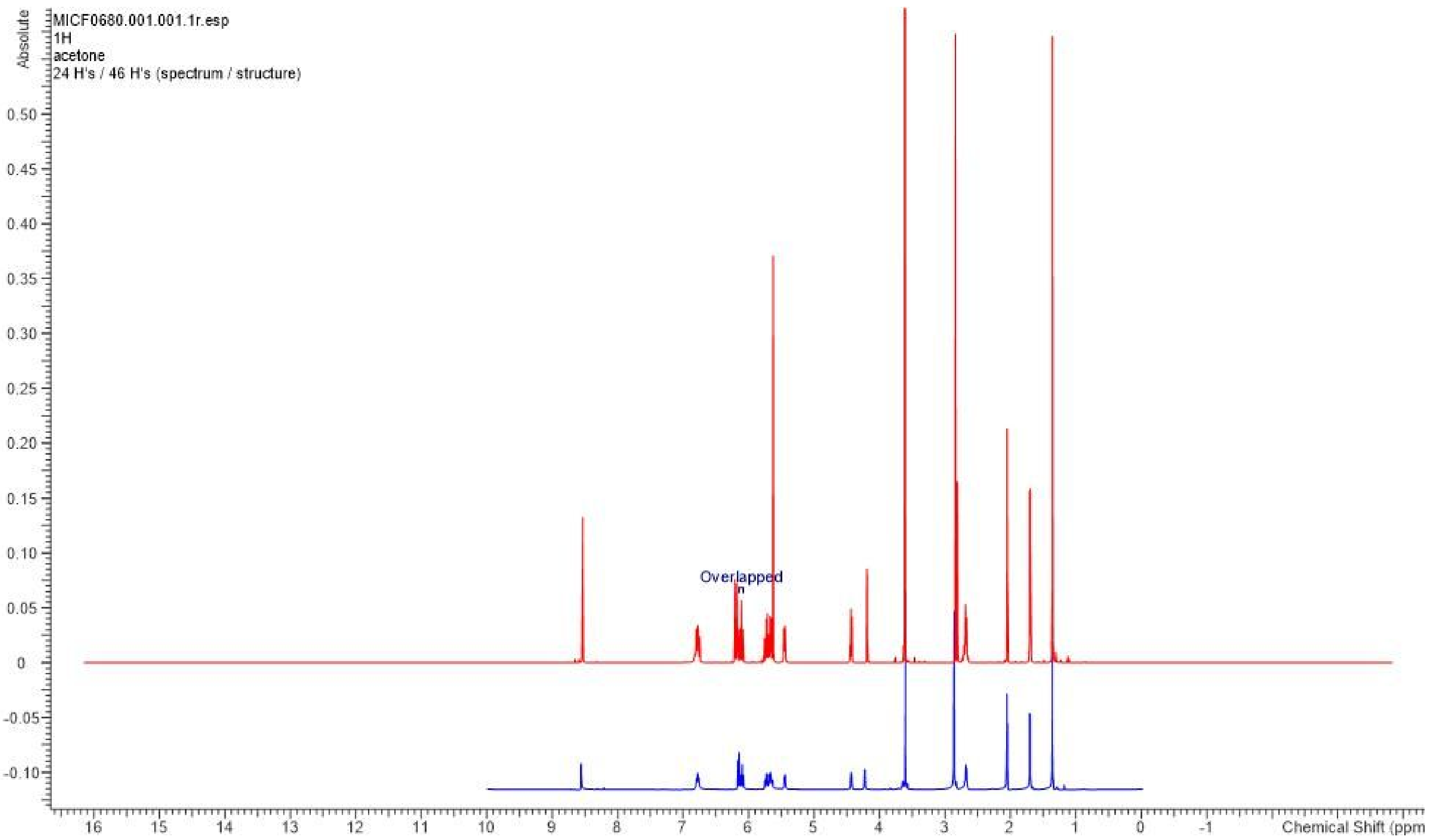


**Figure S17** Comparison of $^{1}$H NMR spectra of **3** in acetone-$d_6$ isolated from *S. cellulosum* and *M. xanthus*. Top/red: isolated from *S. cellulosum* So ce1875; Bottom/blue: isolated from *M. xanthus* DK1622::km-int-Ptet-dis427.

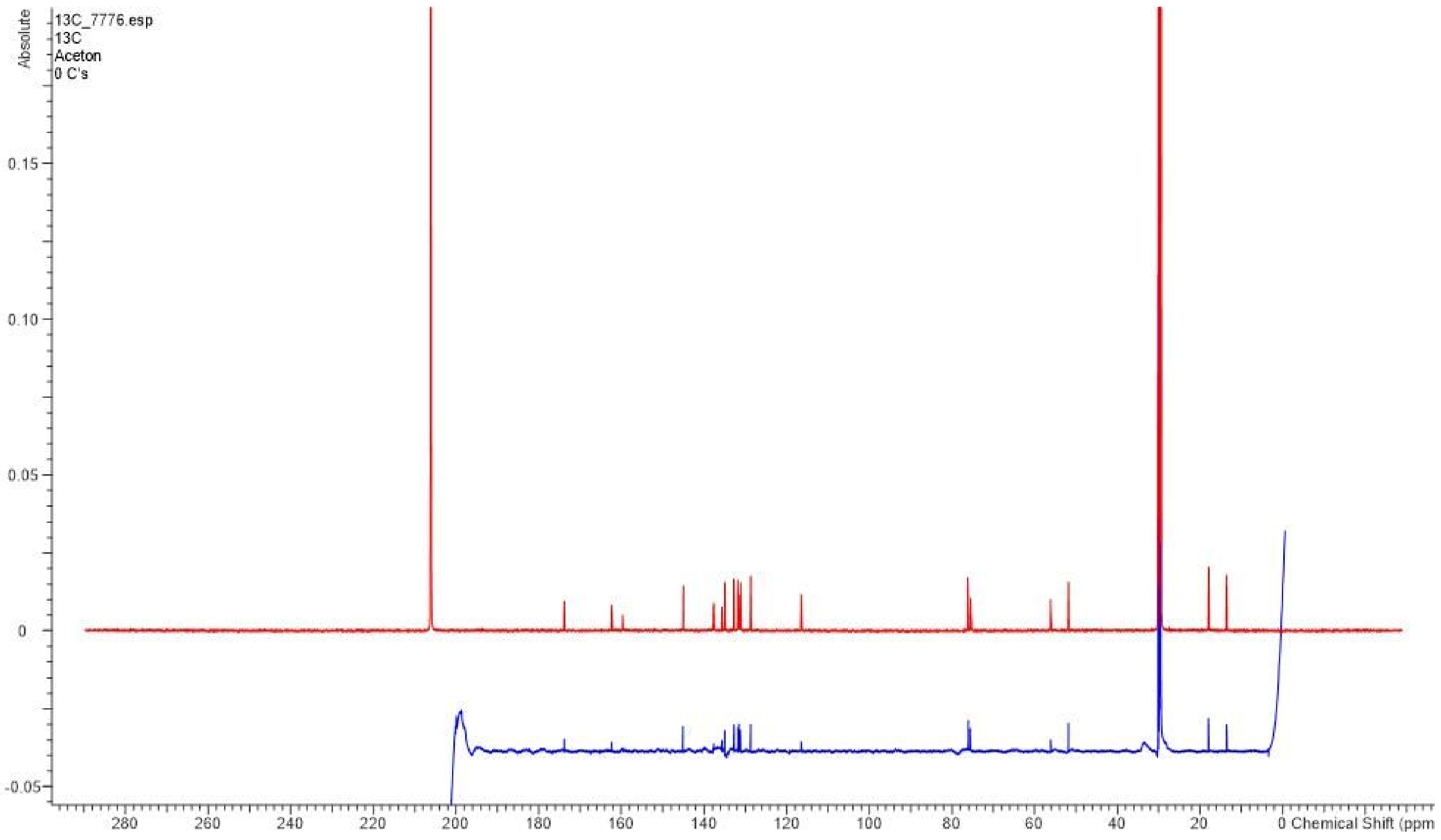


**Figure S18** Comparison of $^{13}C$ NMR spectra of **3** in acetone-$d_6$ isolated from *S. cellulosum* and *M. xanthus*. Top/red: isolated from *S. cellulosum* So ce1875; Bottom/blue: isolated from *M. xanthus* DK1622::km-int-Ptet-dis427.

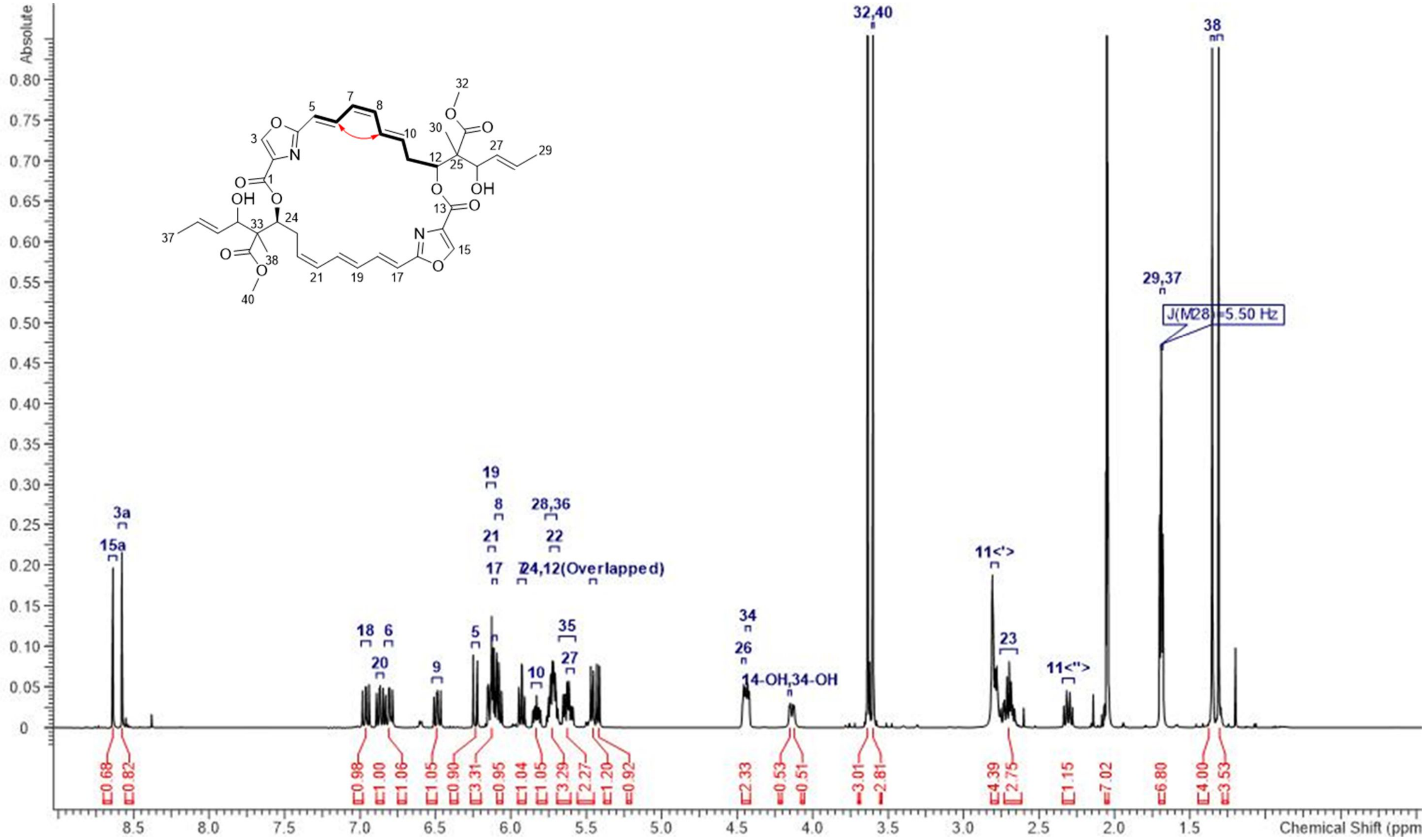

**Figure S19** $^1$H NMR spectrum of *Δ*$^{7,8}$-*cis*-disorazole Z (**4**) in acetone-$d_6$ (500 MHz).

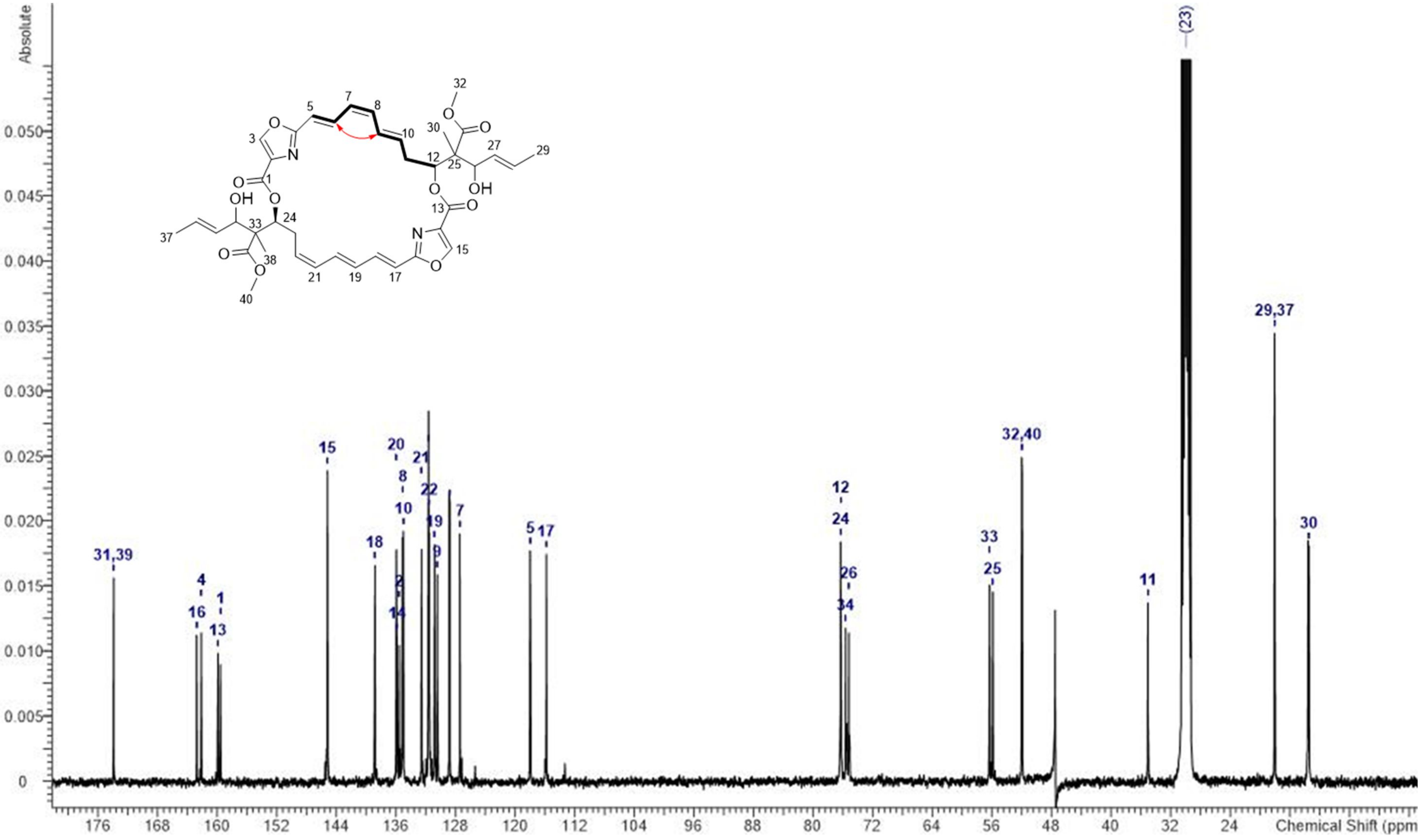


**Figure S20** $^{13}$C NMR spectrum of *Δ*$^{7,8}$-*cis*-disorazole Z (**4**) in acetone-$d_6$ (100 MHz).

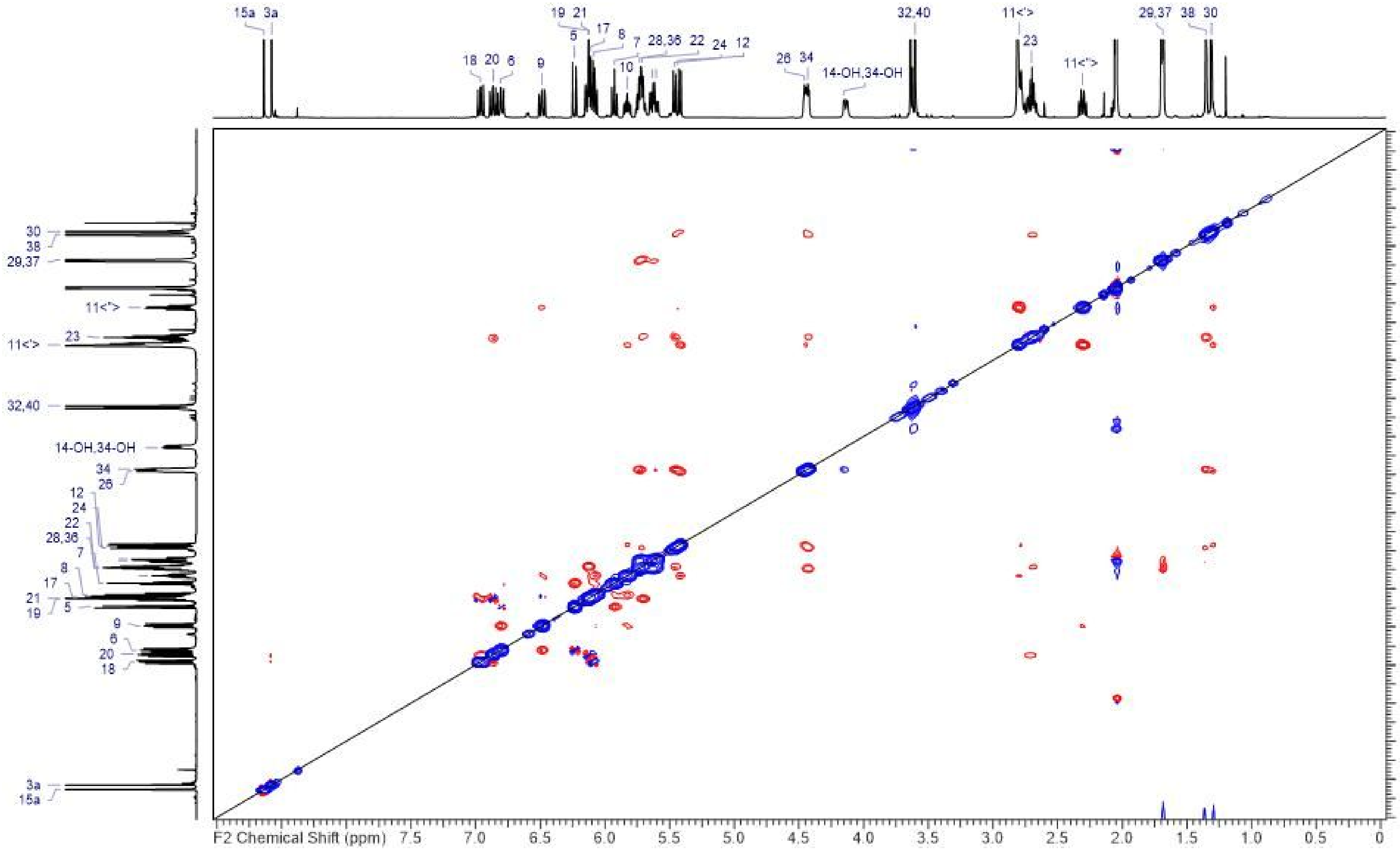


**Figure S21** $^1H,^1H$-ROESY NMR spectrum of $\Delta^{7,8}$-*cis*-disorazole Z (**4**) in acetone-$d_6$.

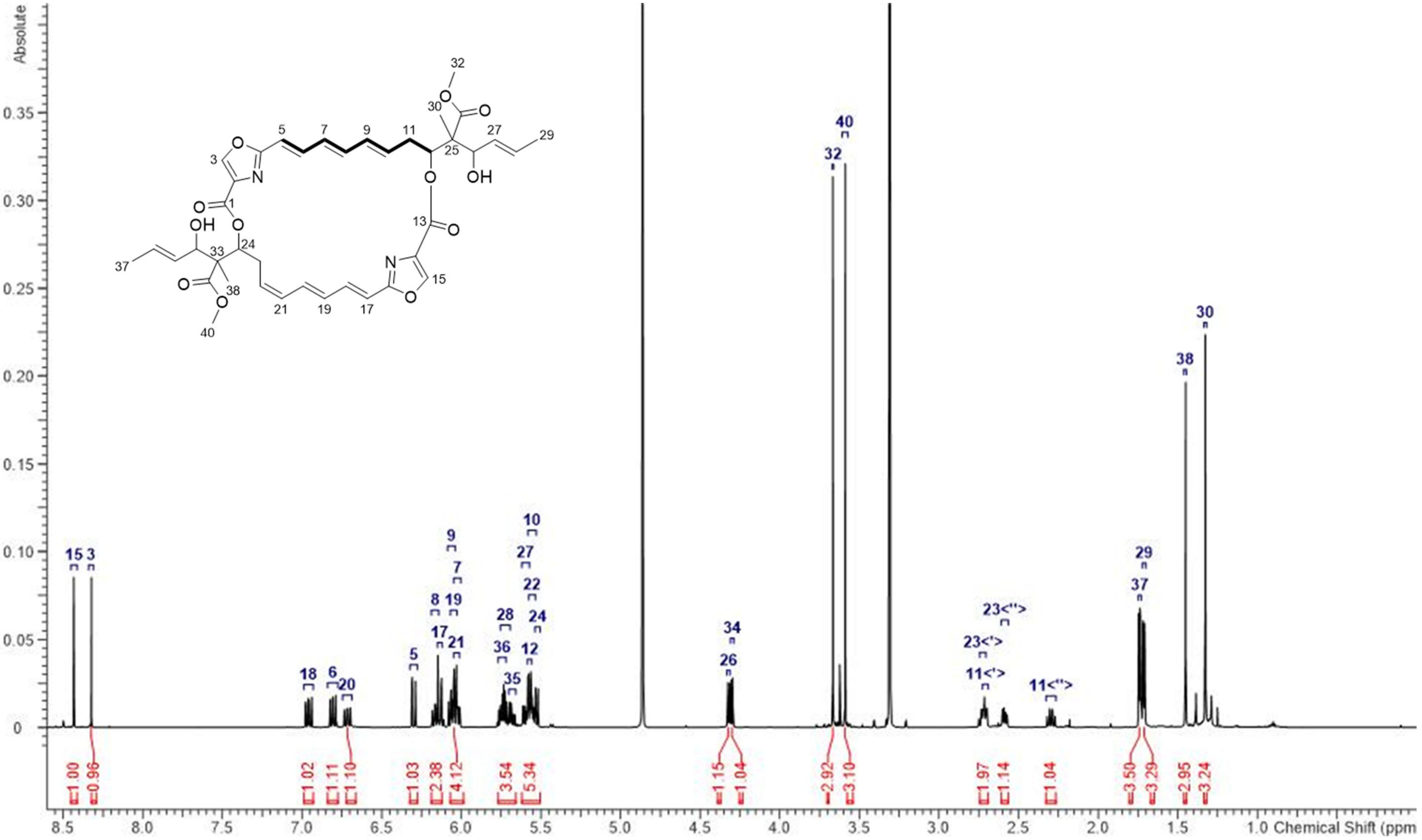


**Figure S22** $^{1}$H NMR spectrum of *Δ*$^{9,10}$-*trans*-disorazole Z (**5**) in methanol-$d_4$ (700 MHz).

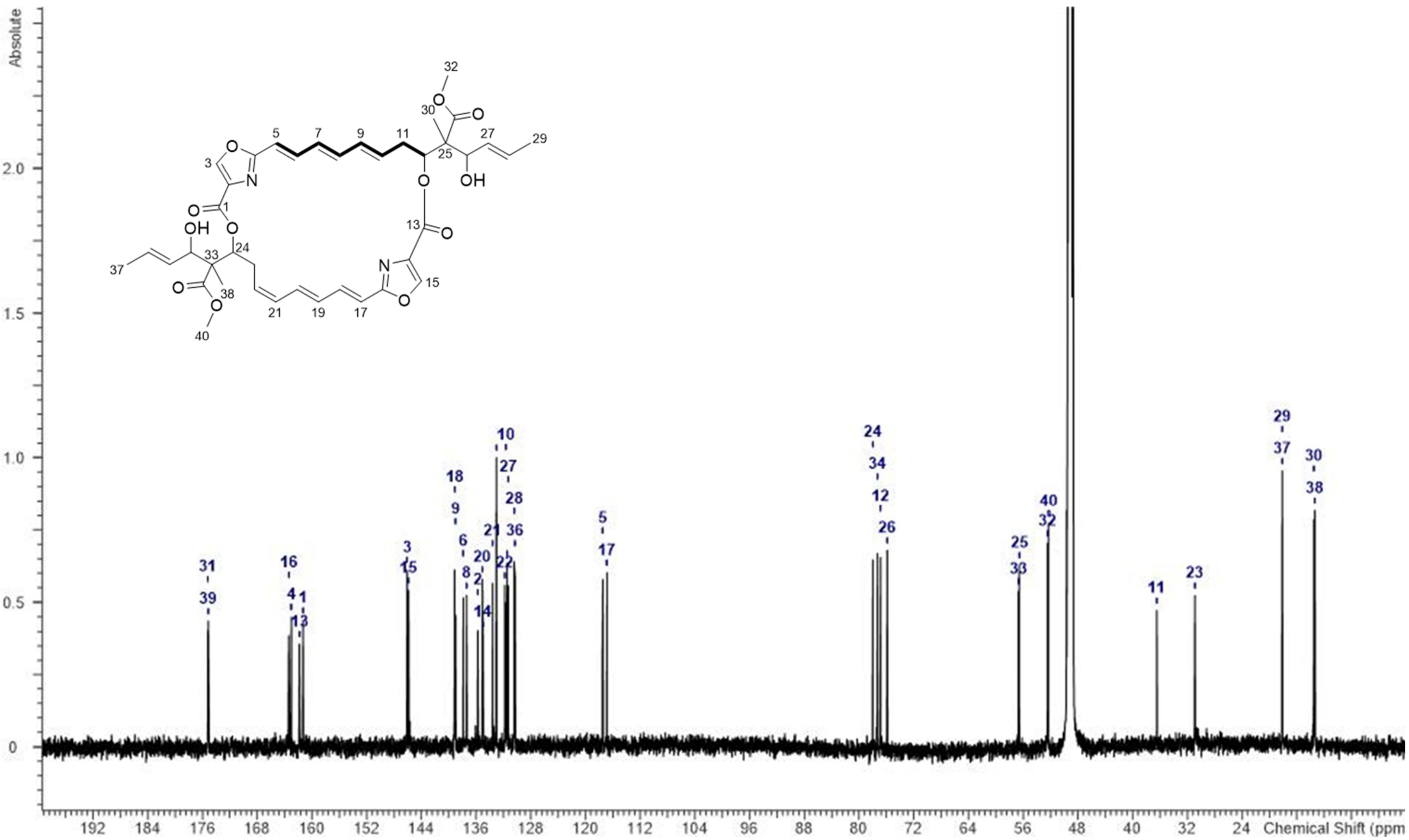

**Figure S23** $^{13}$C NMR spectrum of *Δ*$^{9,10}$-*trans*-disorazole Z (**5**) in in methanol-$d_4$ (175 MHz).

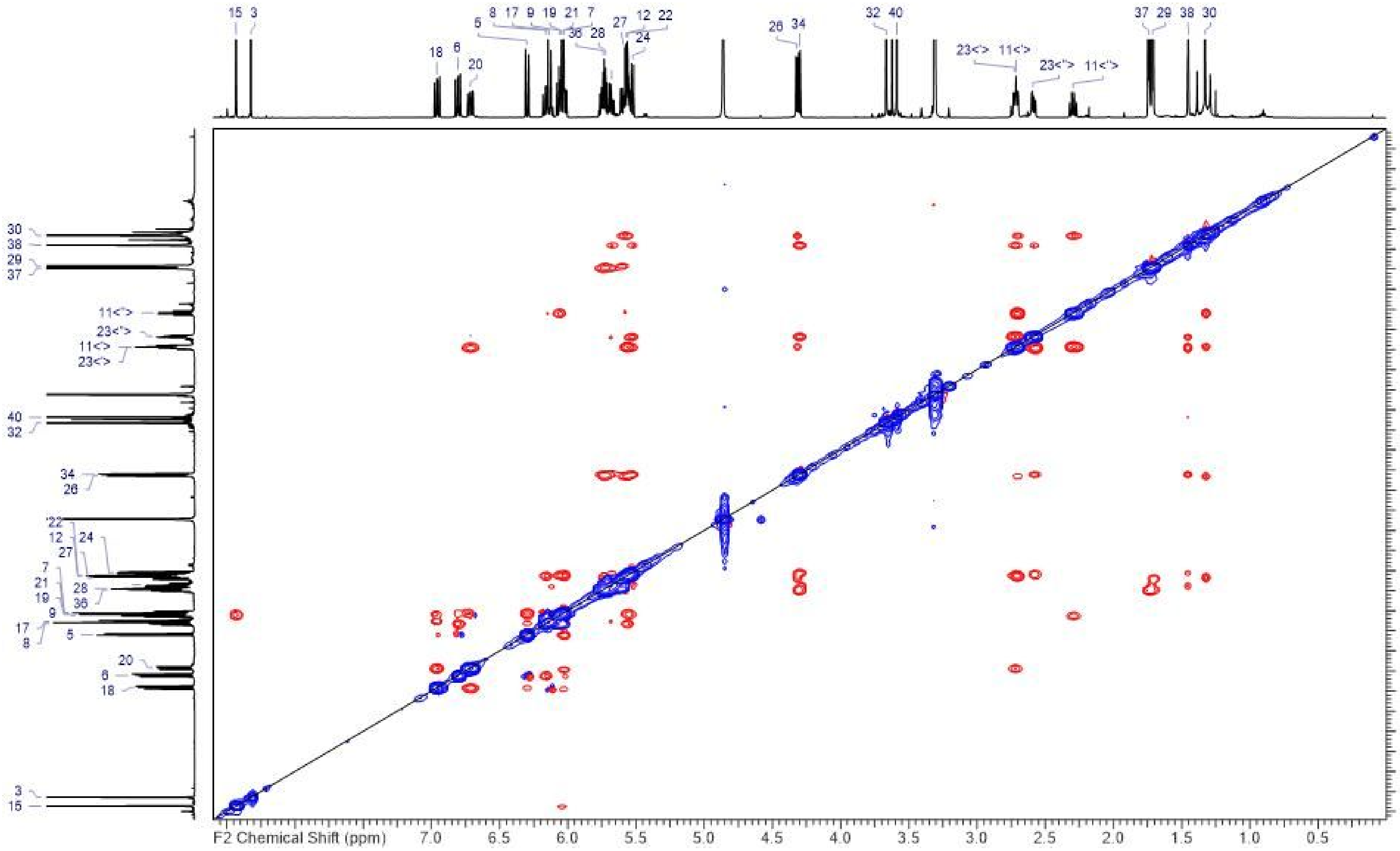

**Figure S24** $^{1}H$,$^{1}H$-ROESY NMR spectrum of $\Delta^{9,10}$-*trans*-disorazole Z (**5**) in methanol-$d_4$.

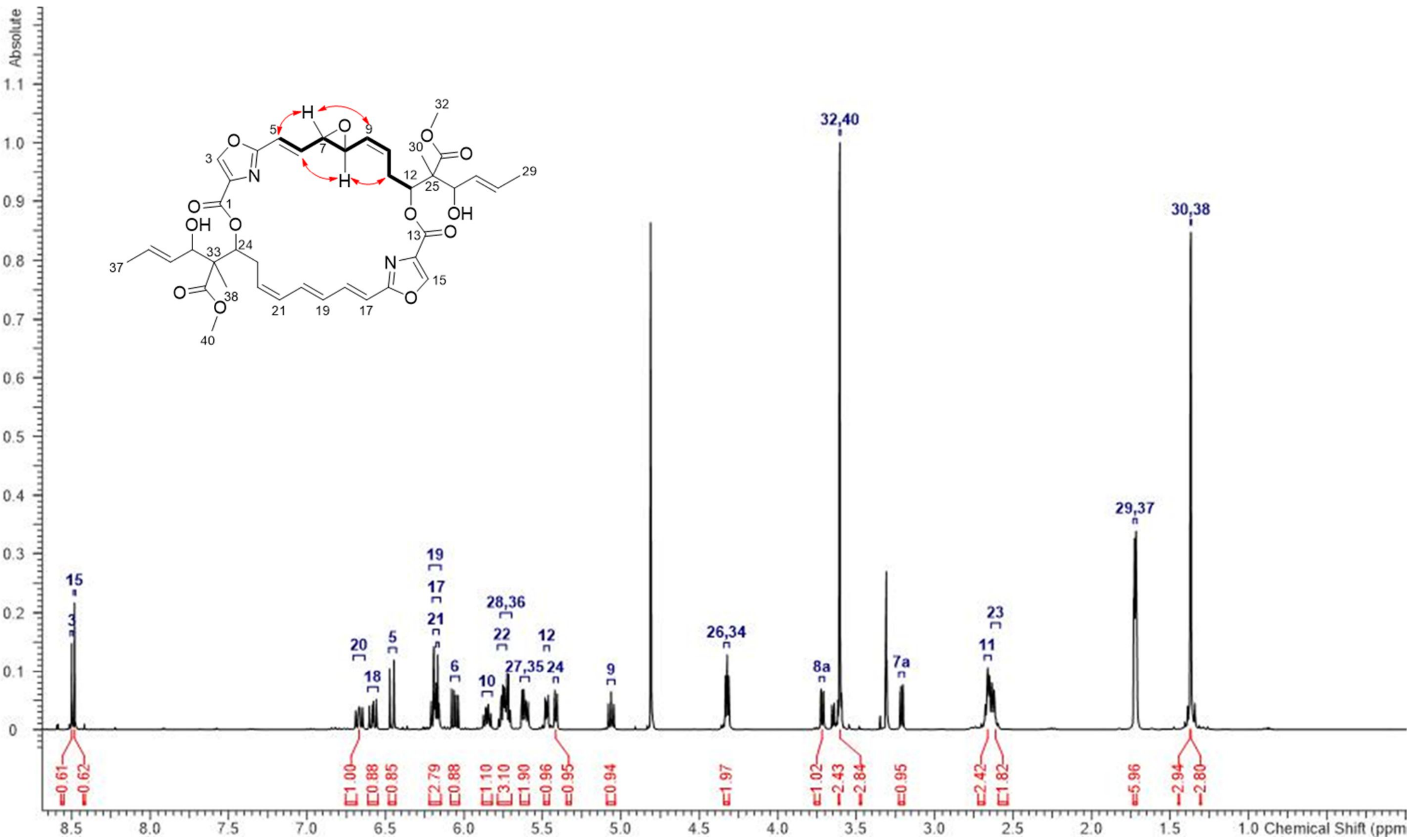

**Figure S25** $^{1}$H NMR spectrum of 7,8-epoxy-disorazole Z (**6**) in methanol-$d_4$ (600 MHz).

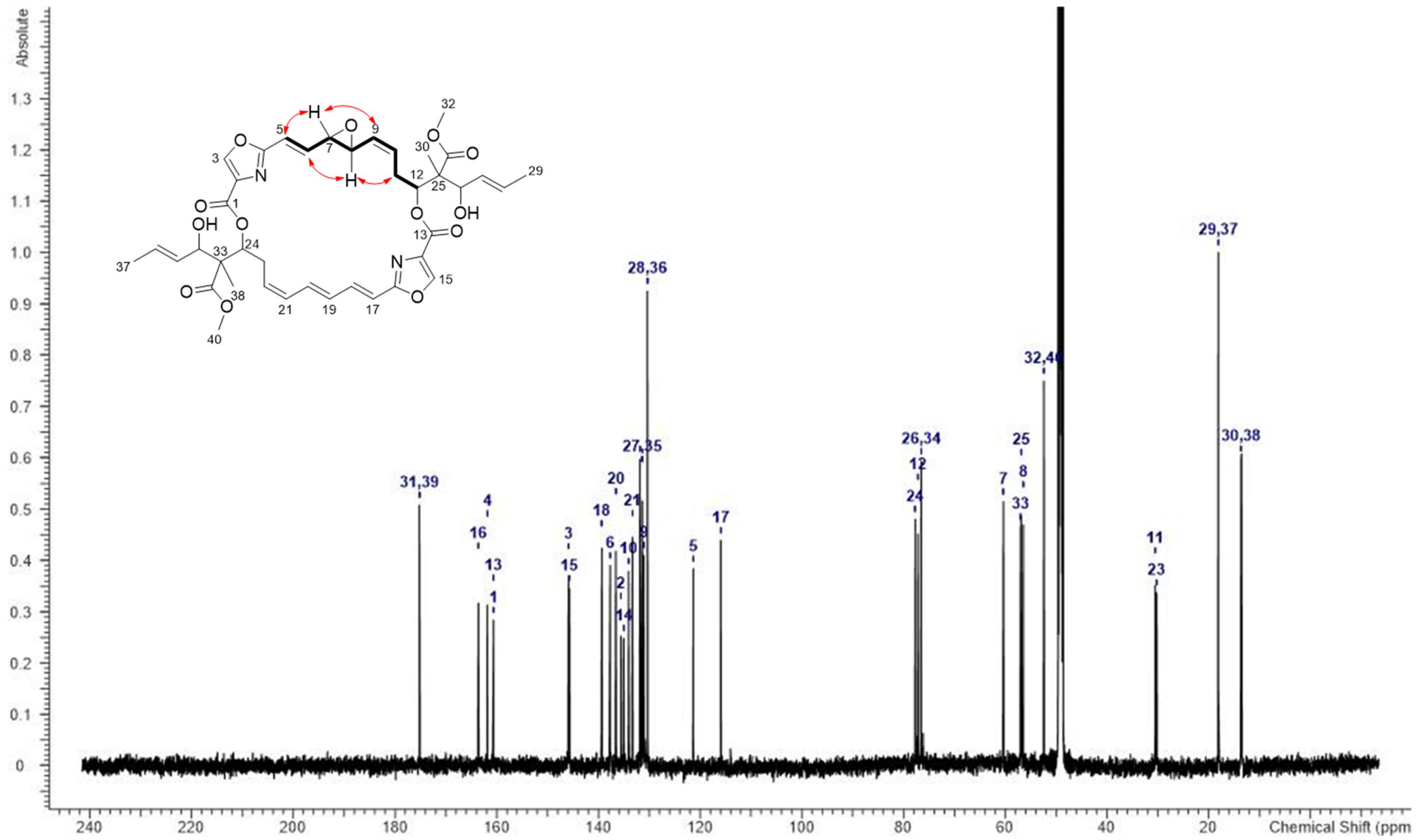


**Figure S26** $^{13}C$ NMR spectrum of 7,8-epoxy-disorazole Z (**6**) in in methanol-$d_4$ (150 MHz).

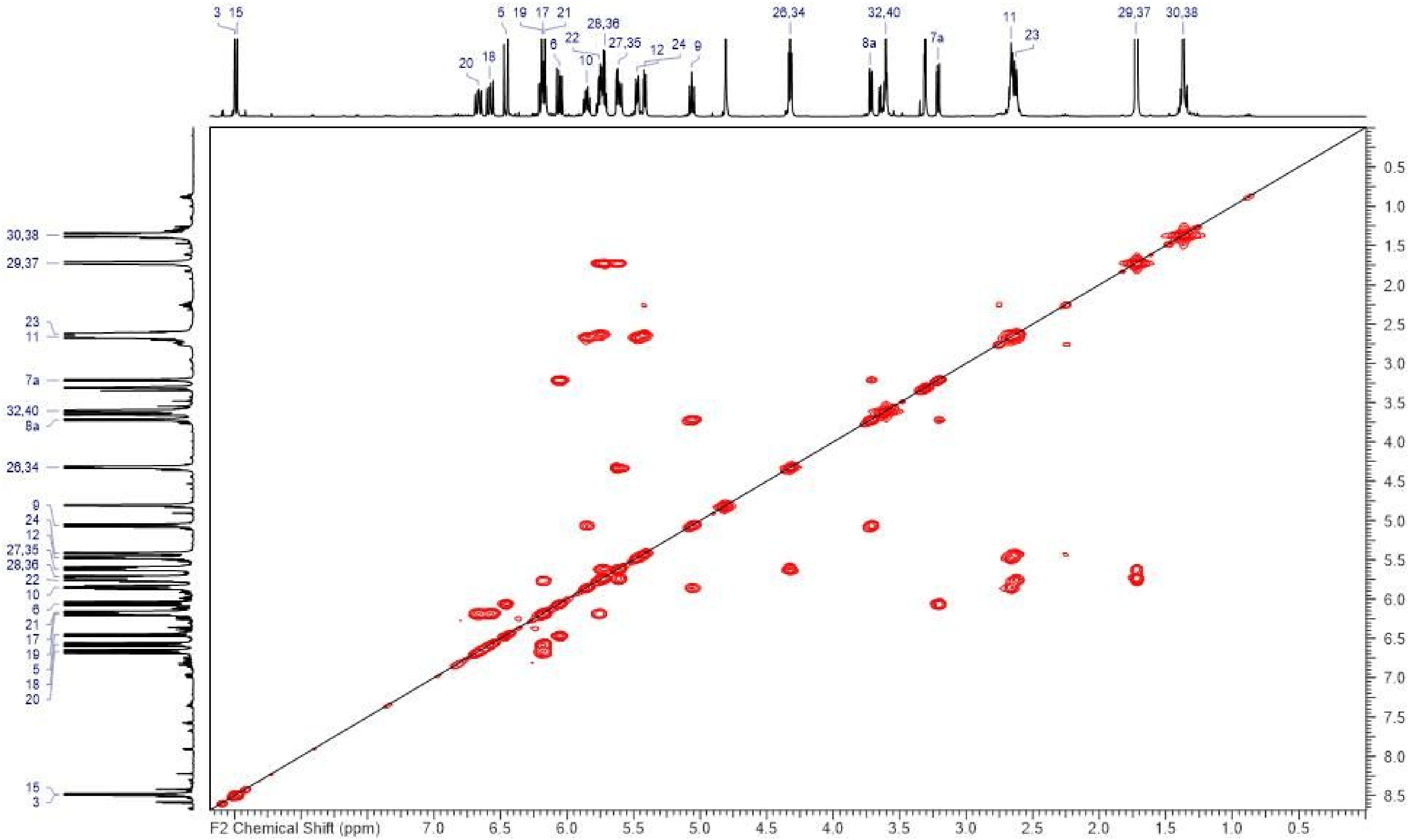


**Figure S27** $^1$H,$^1$H-COSY NMR spectrum of 7,8-epoxy-disorazole Z (**6**) in in methanol-$d_4$.

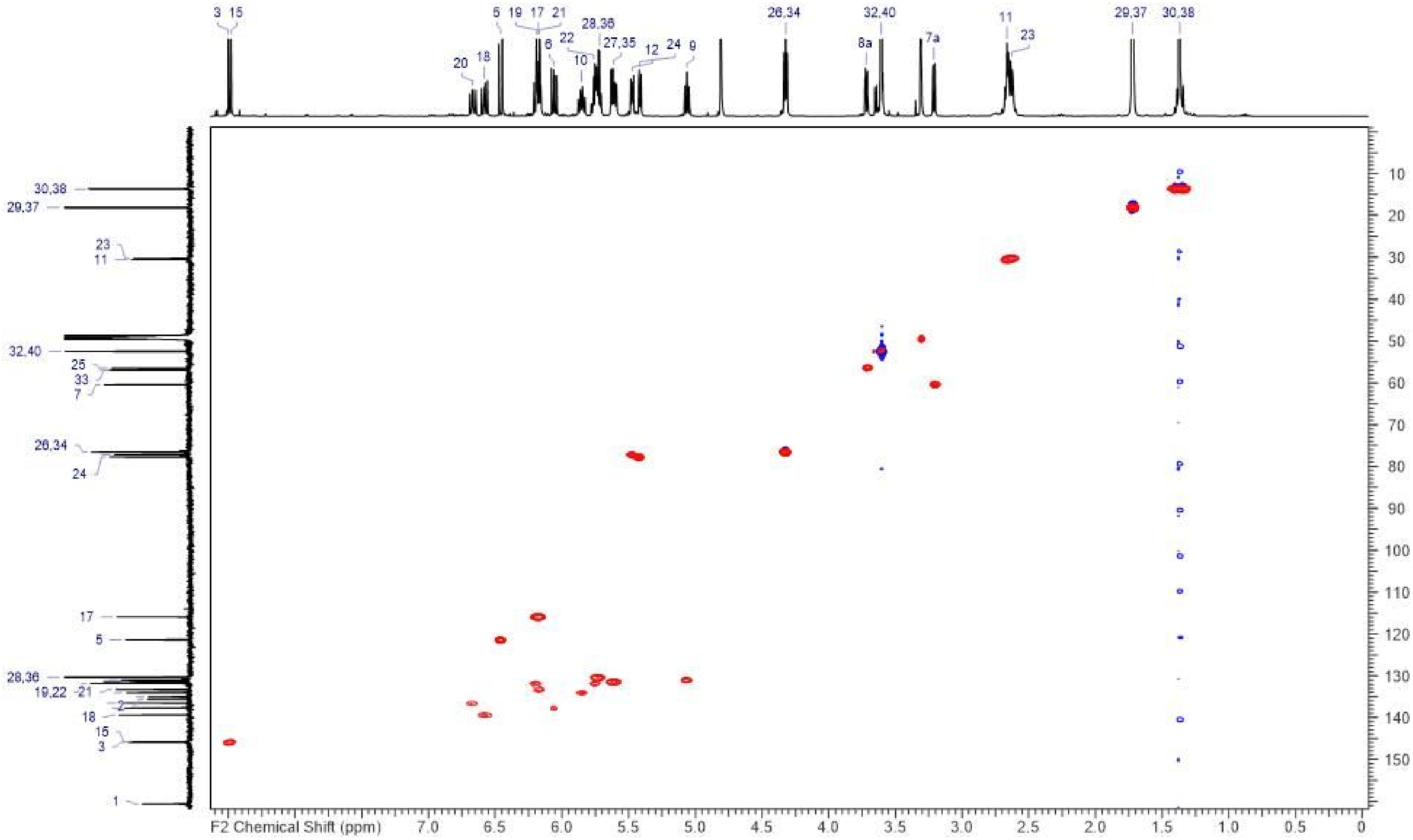


**Figure S28** HMQC-NMR spectrum of 7,8-epoxy-disorazole Z (**6**) in in methanol-*d*4.

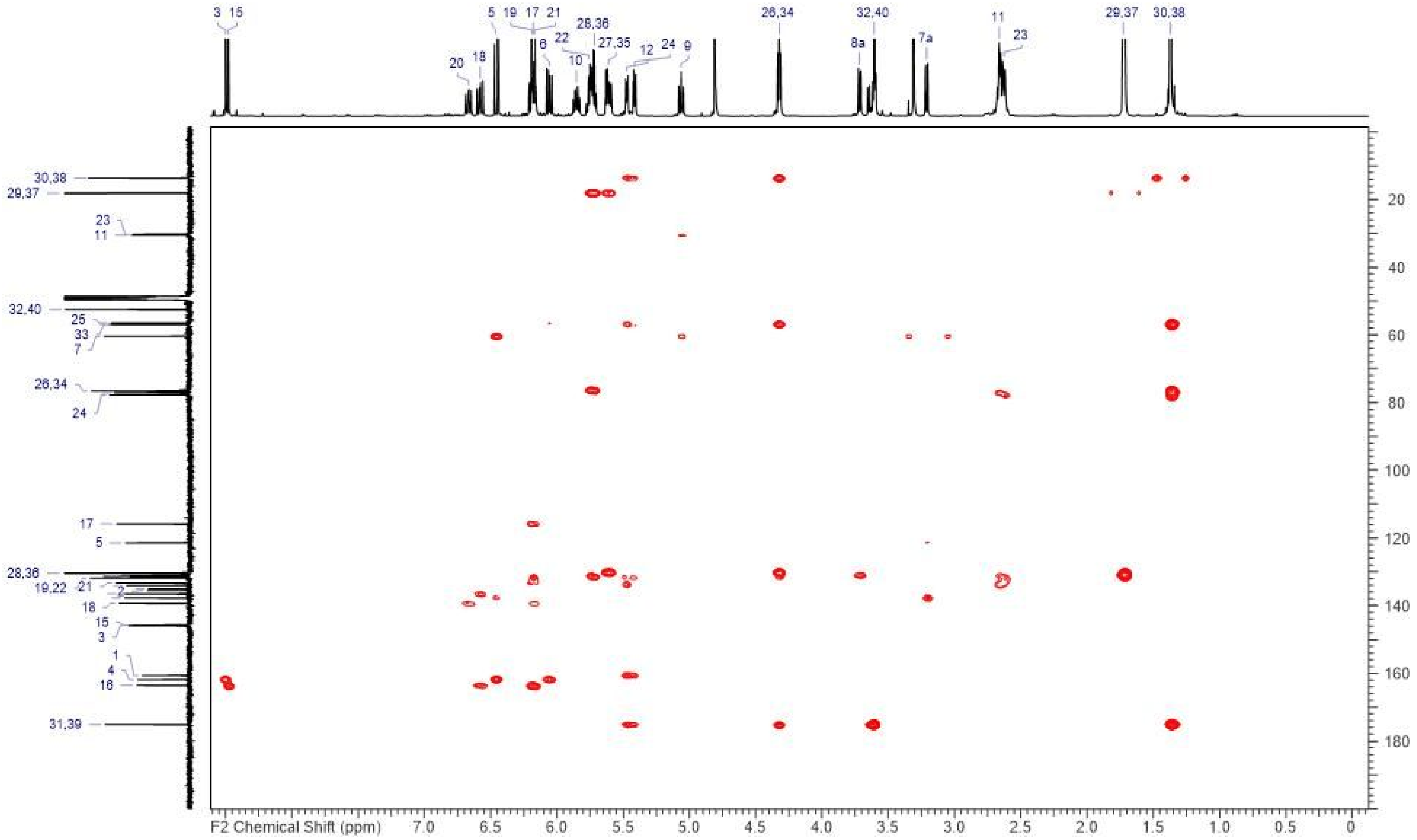

**Figure S29** HMBC NMR spectrum of 7,8-epoxy-disorazole Z (**6**) in in methanol-$d_4$.

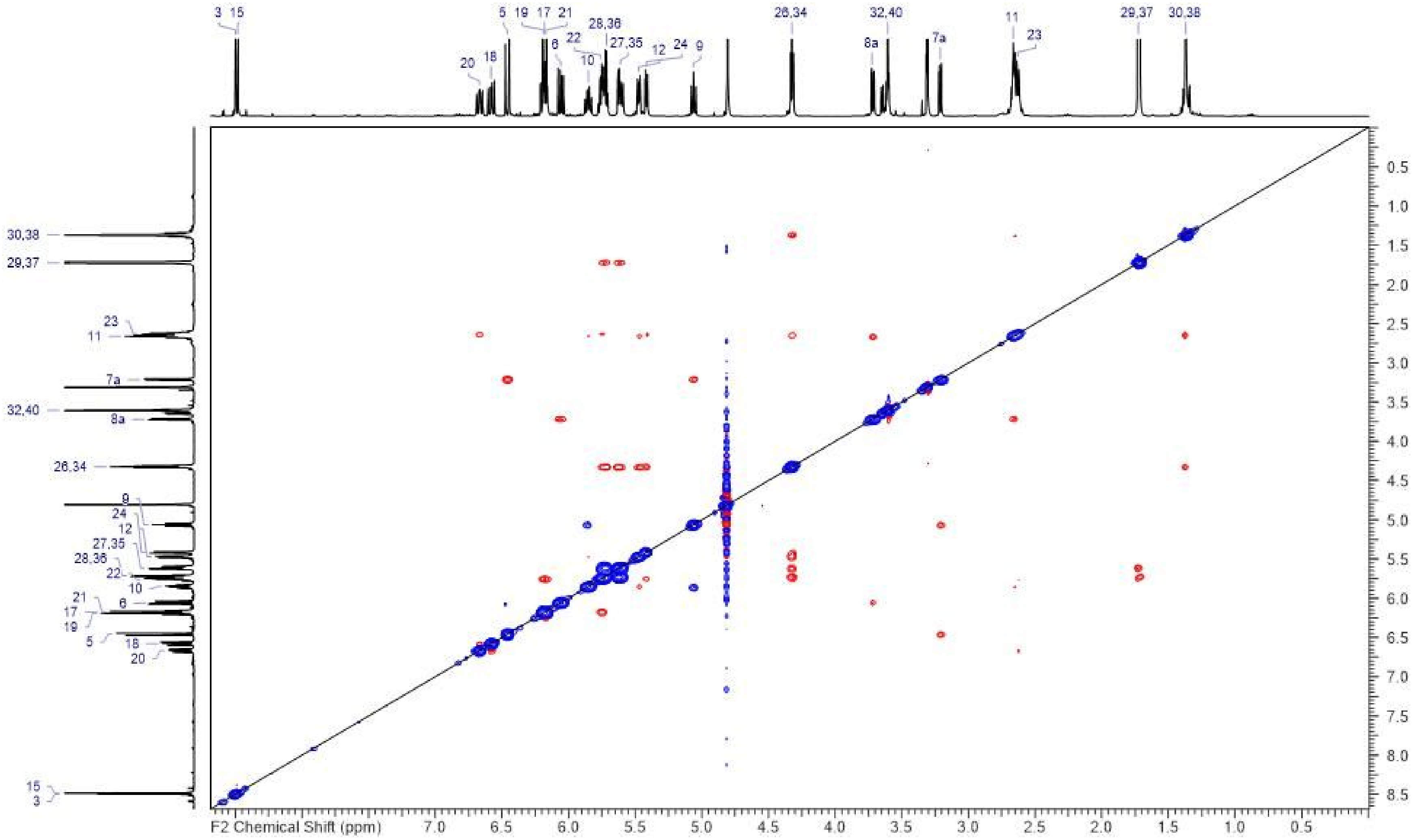


**Figure S30** $^{1}H,^{1}H$-ROESY NMR spectrum of 7,8-epoxy-disorazole Z (**6**) in methanol-$d_4$.

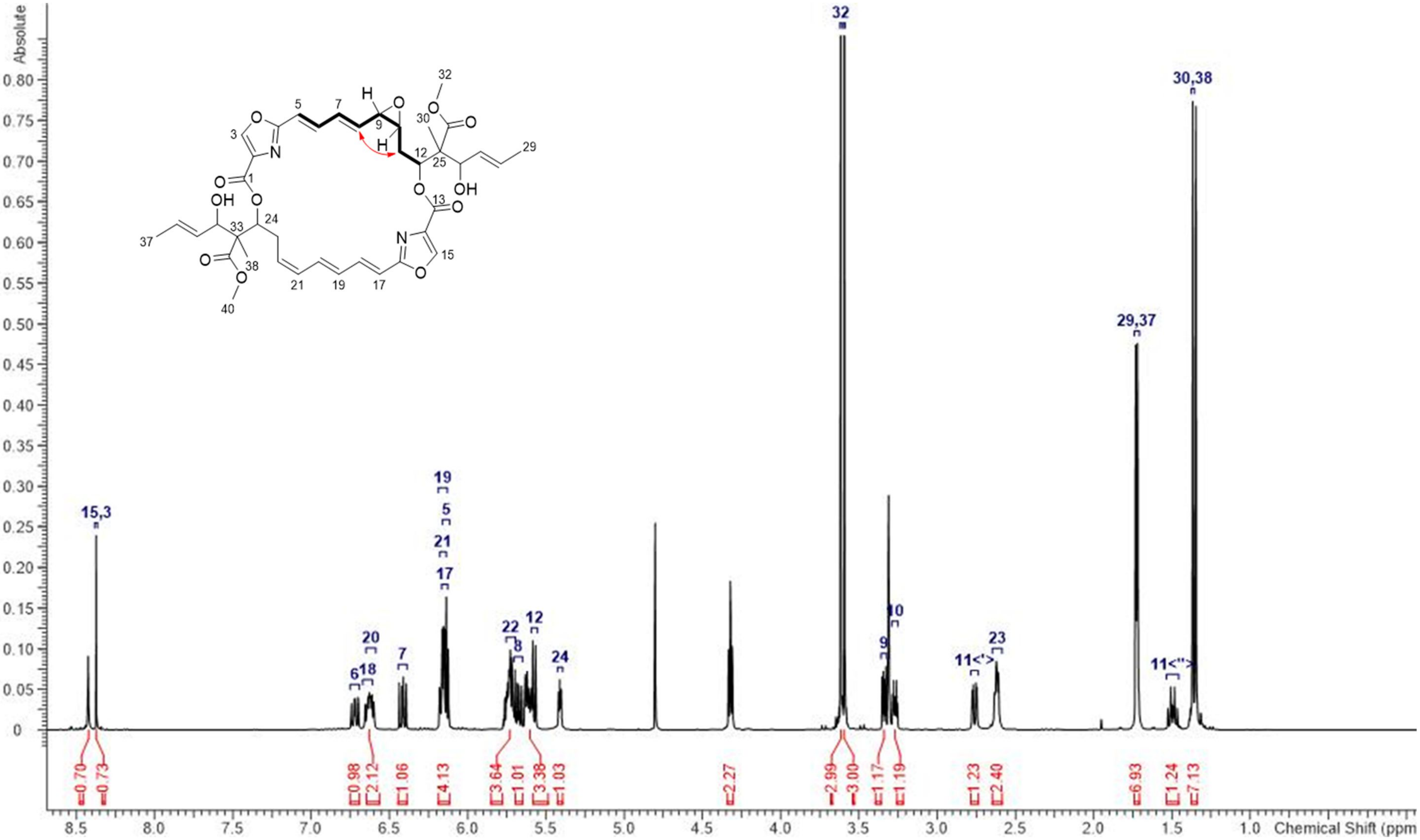


**Figure S31** $^{1}$H NMR spectrum of 9,10-epoxy-disorazole Z (**7**) in methanol-$d_4$ (600 MHz).

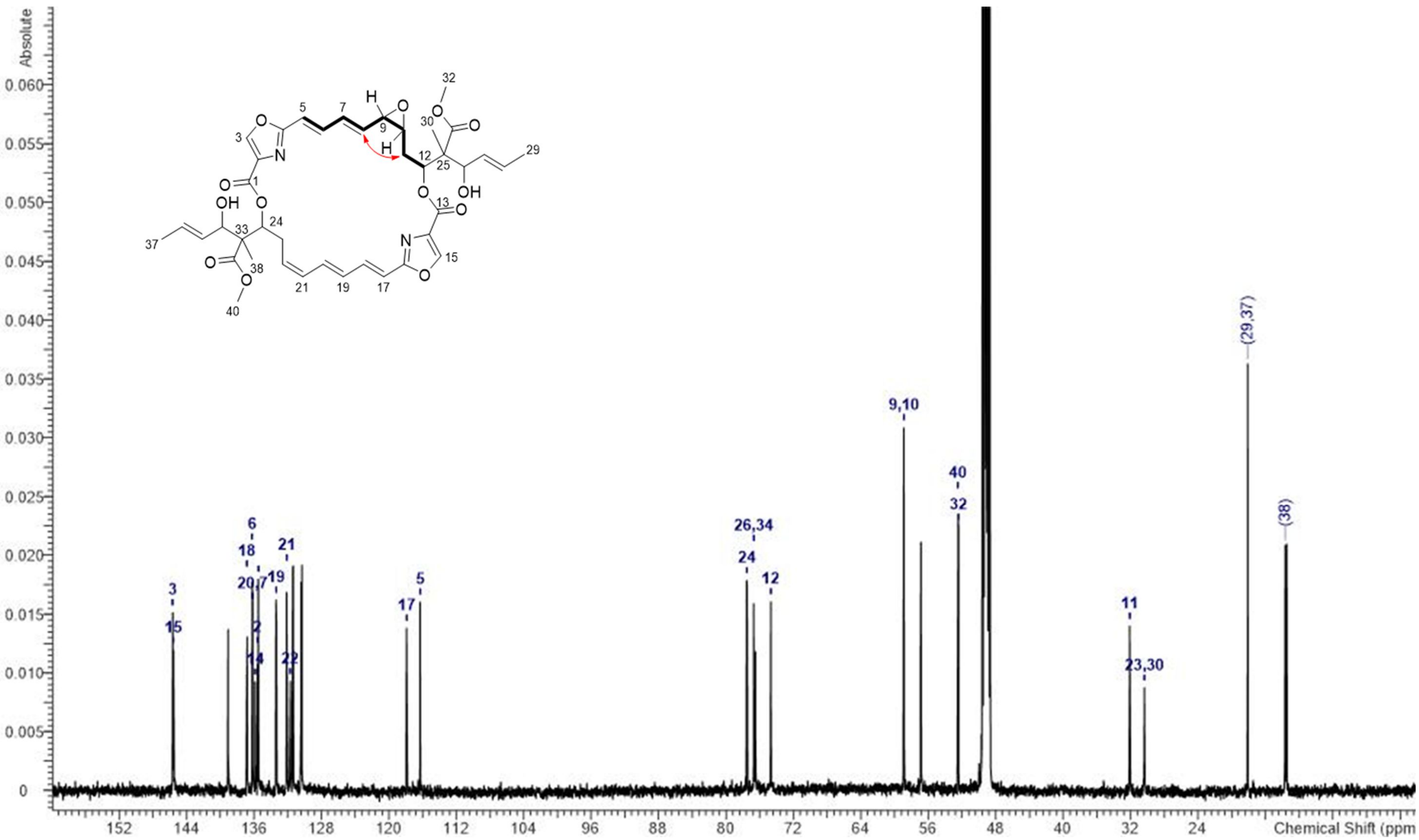


**Figure S32** $^{13}$C NMR spectrum of 9,10-epoxy-disorazole Z (**7**) in in methanol-$d_4$ (150 MHz).

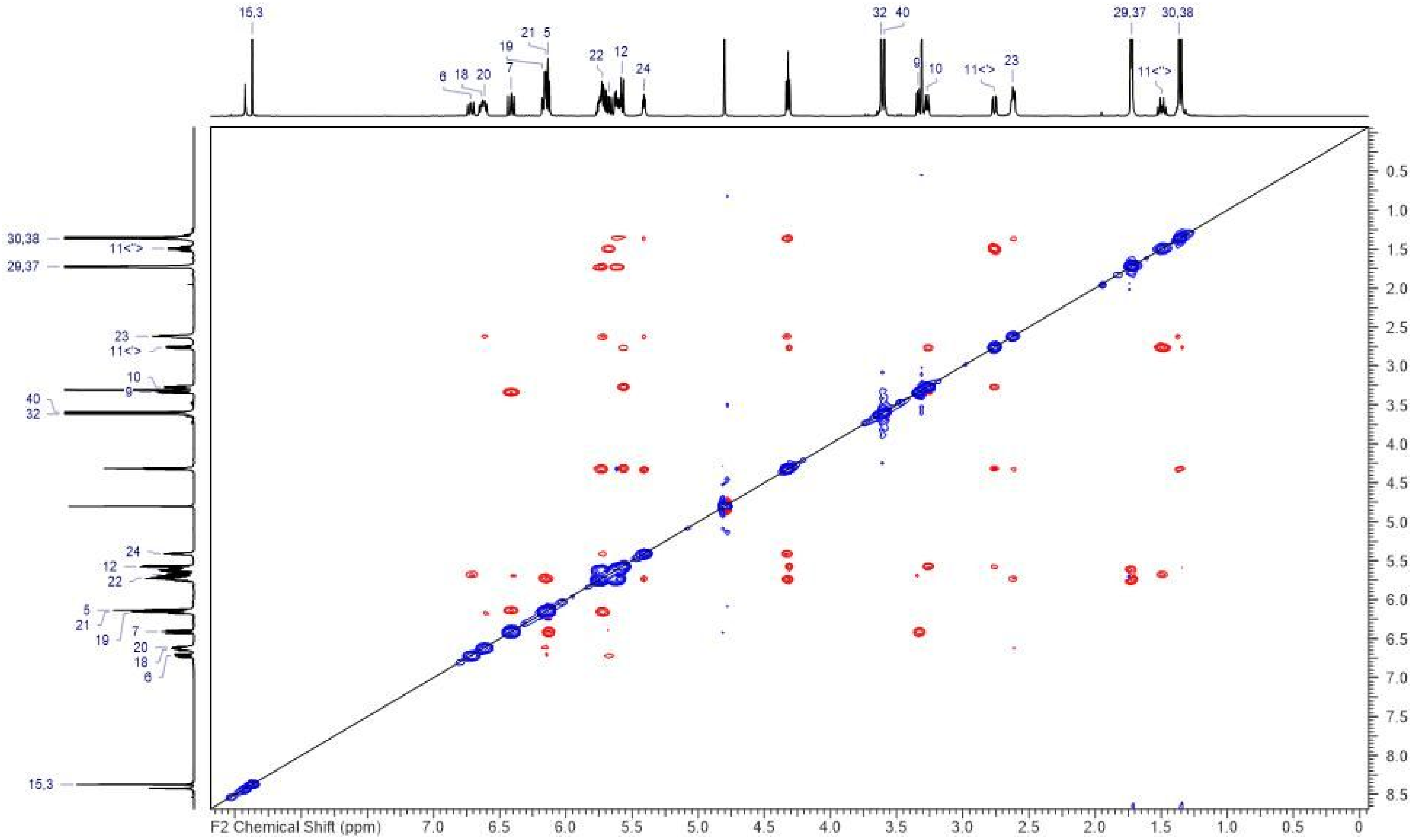


**Figure S33** $^{1}H,^{1}H$-ROESY NMR spectrum of 9,10-epoxy-disorazole Z (**7**) in methanol-$d_4$.

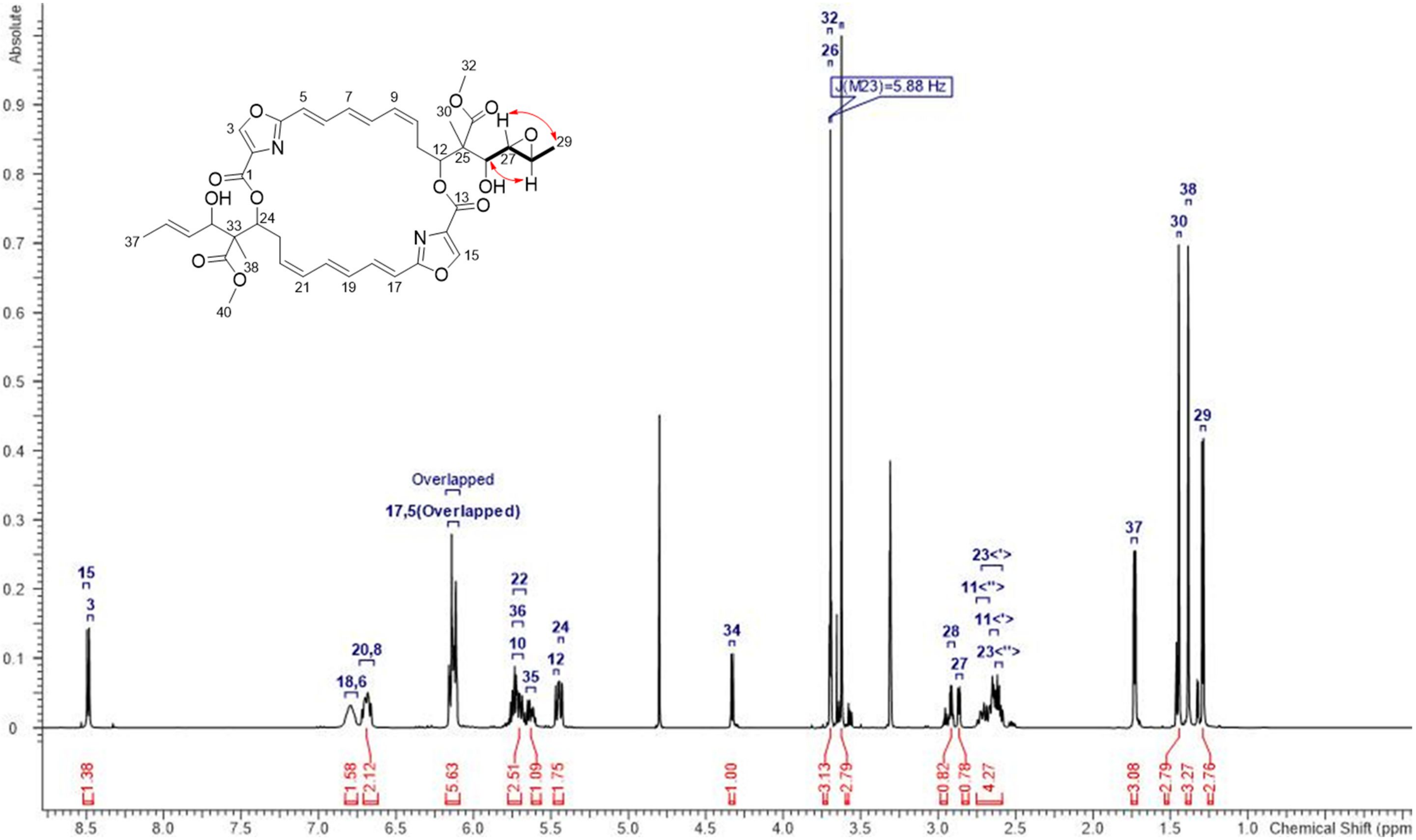


**Figure S34** $^{1}H$ NMR spectrum of 27,28-epoxy-disorazole Z (**8**) in methanol-*d*$_4$ (600 MHz).

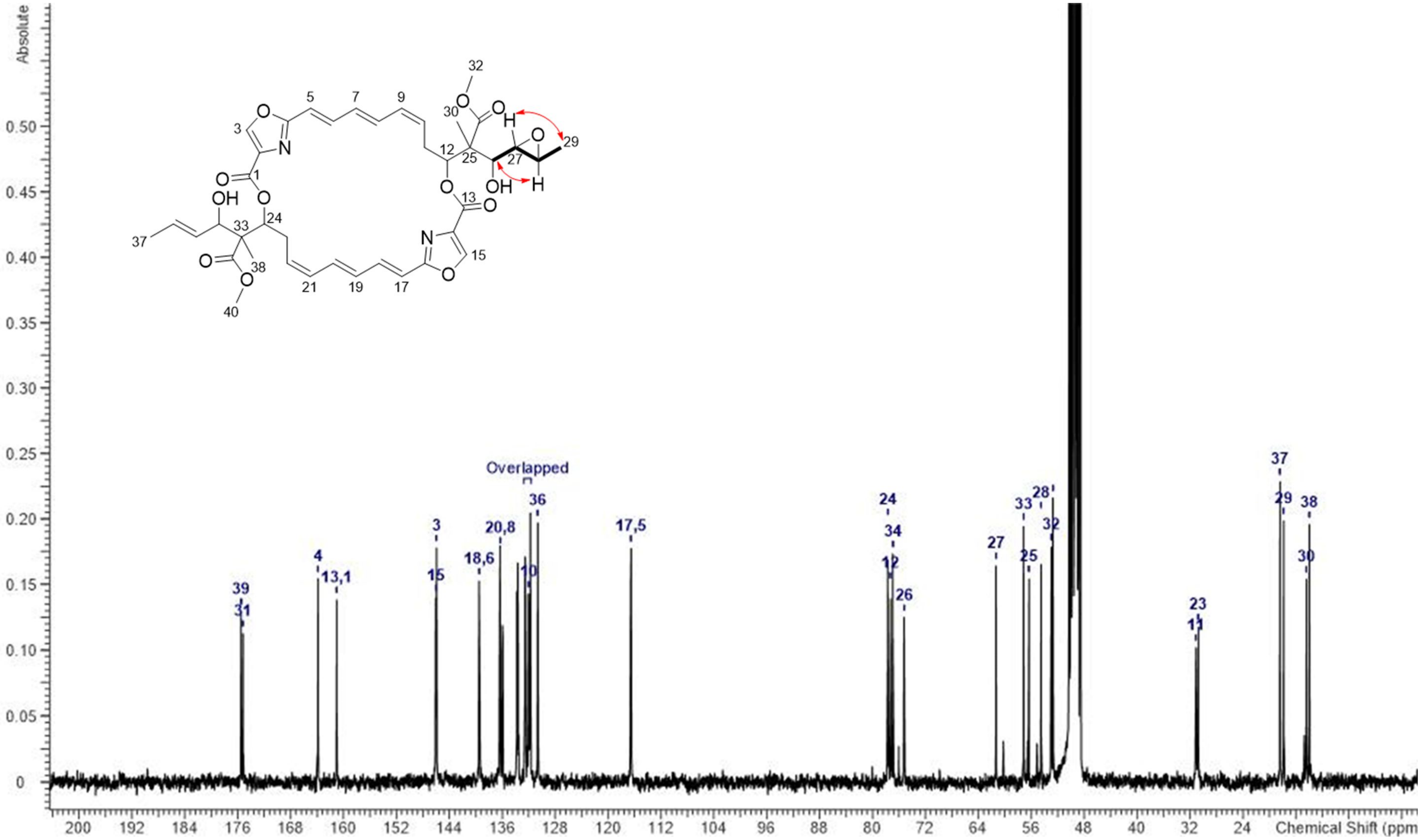

**Figure S35** $^{13}C$ NMR spectrum of 27,28-epoxy-disorazole Z (**8**) in in methanol-$d_4$ (150 MHz).

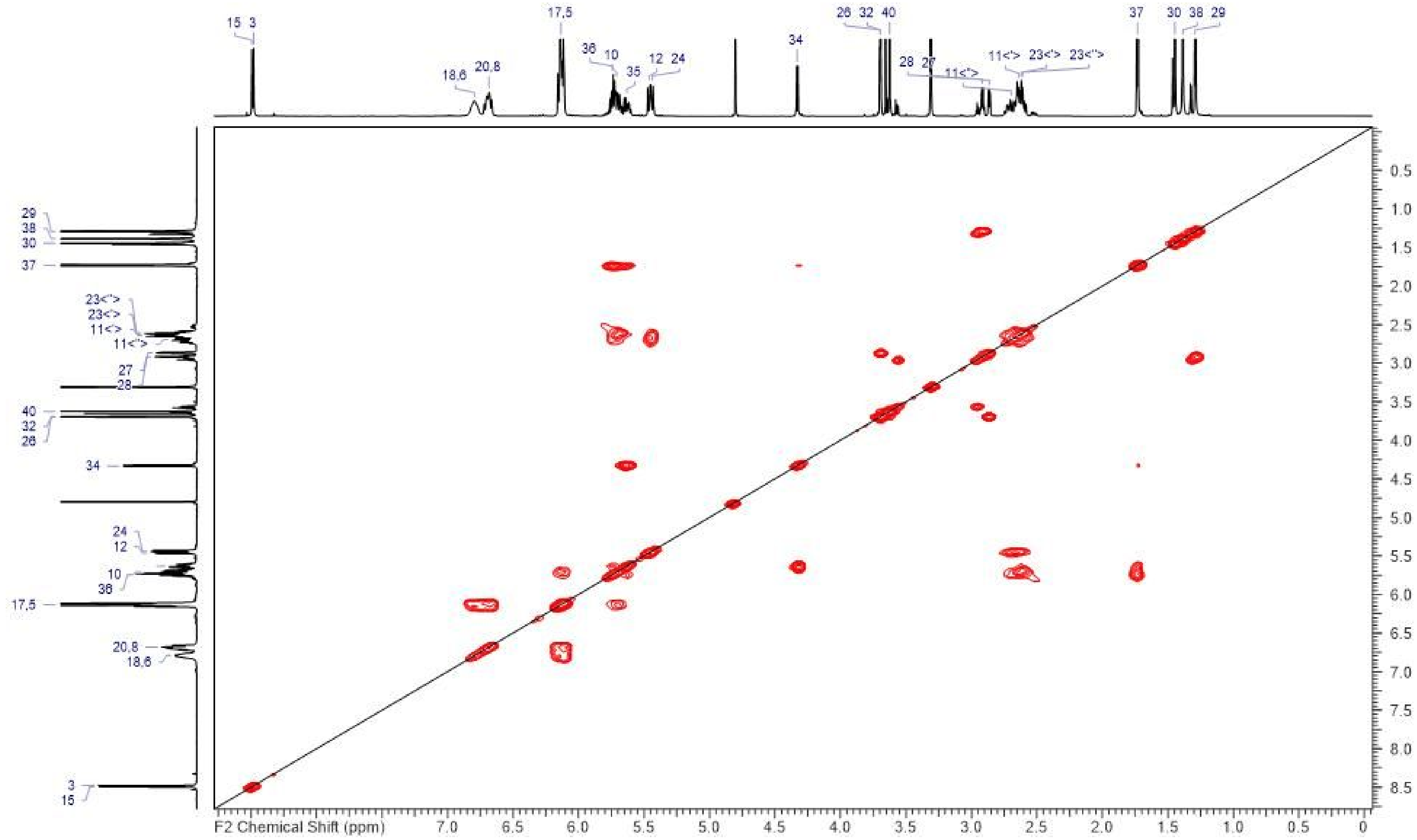


**Figure S36** $^{1}H,^{1}H$-COSY NMR spectrum of 27,28-epoxy-disorazole Z (**8**) in in methanol-$d_4$.

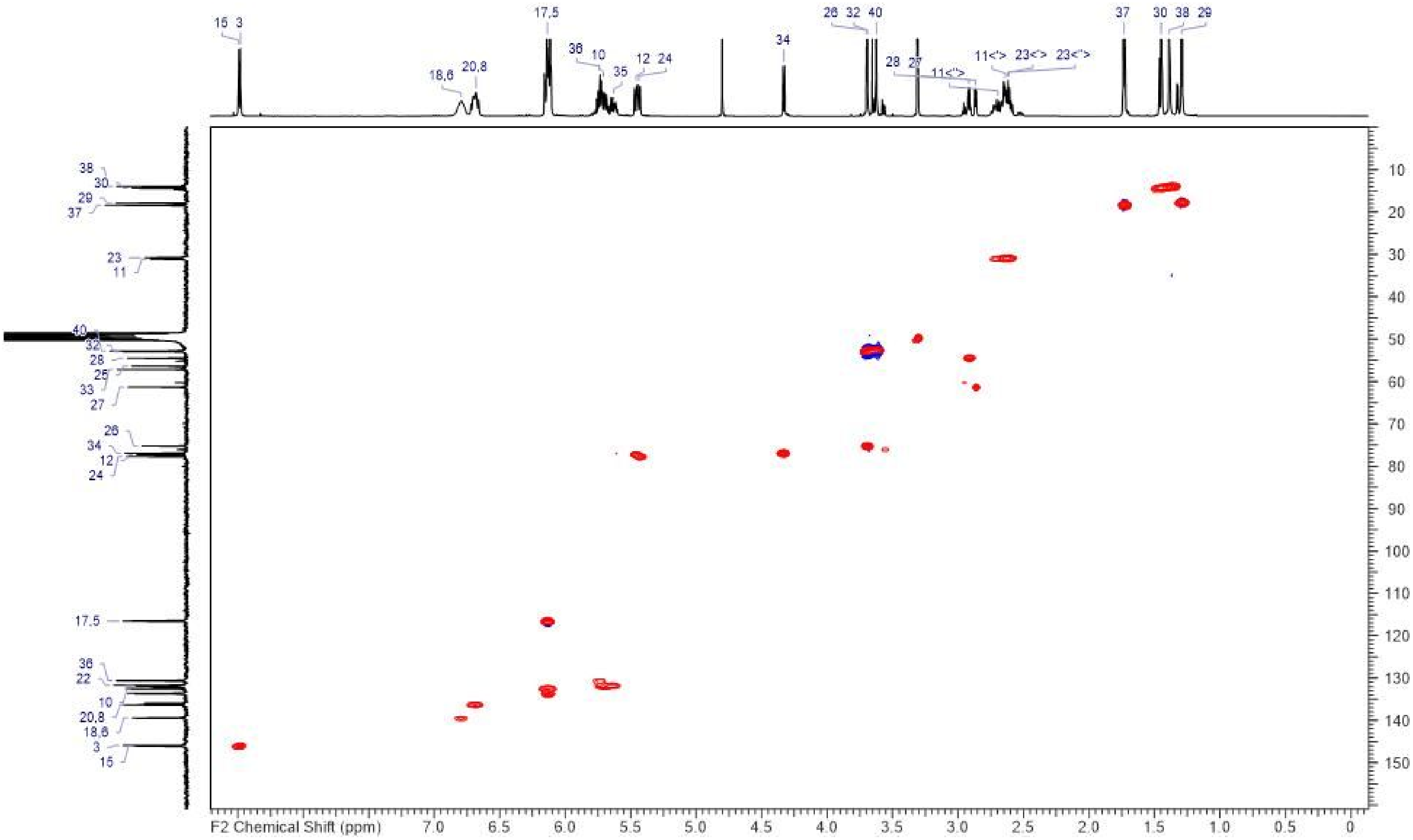


**Figure S37** HMQC-NMR spectrum of 27,28-epoxy-disorazole Z (**8**) in in methanol-$d_4$.

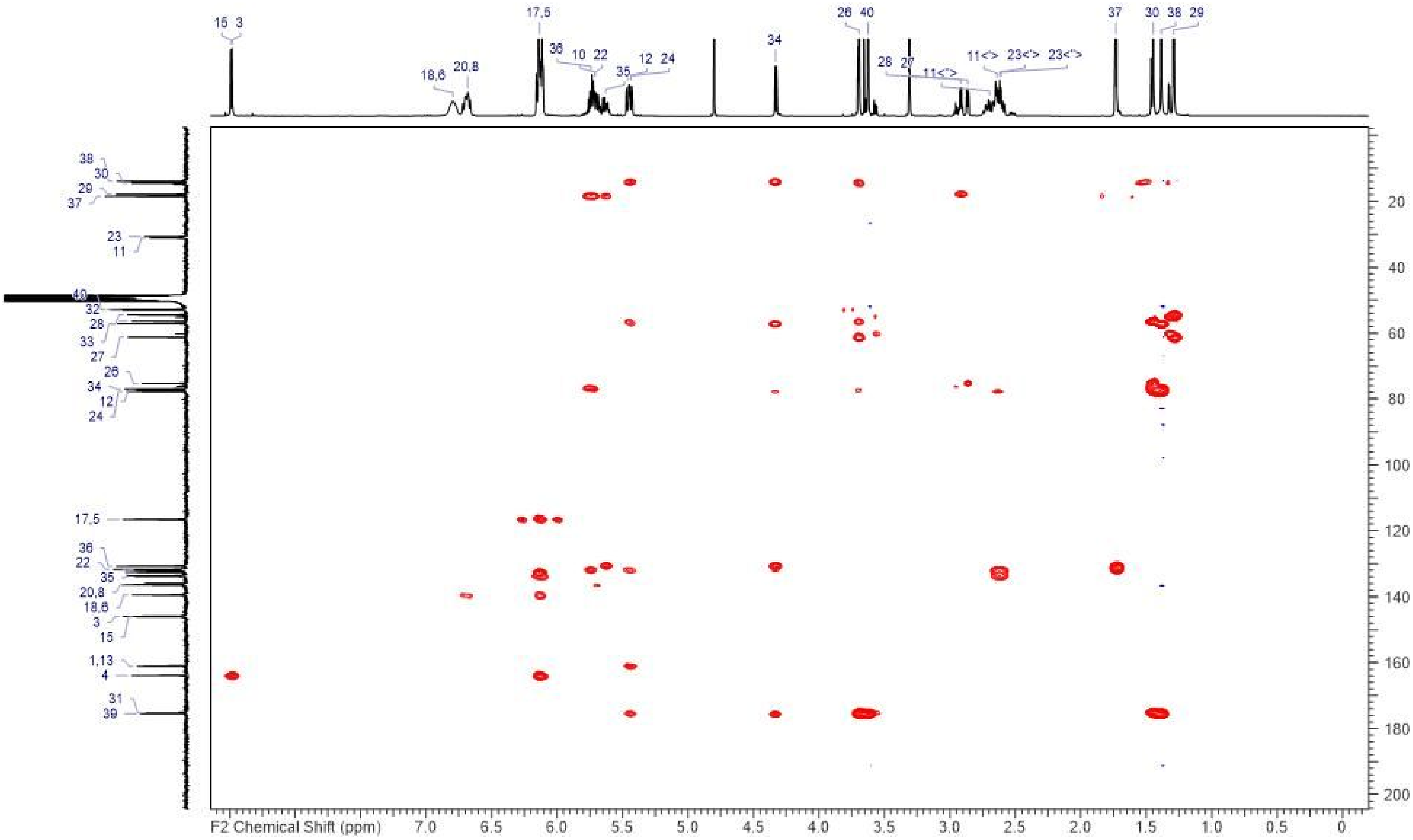


**Figure S38** HMBC NMR spectrum of 27,28-epoxy-disorazole Z (**8**) in in methanol-$d_4$.

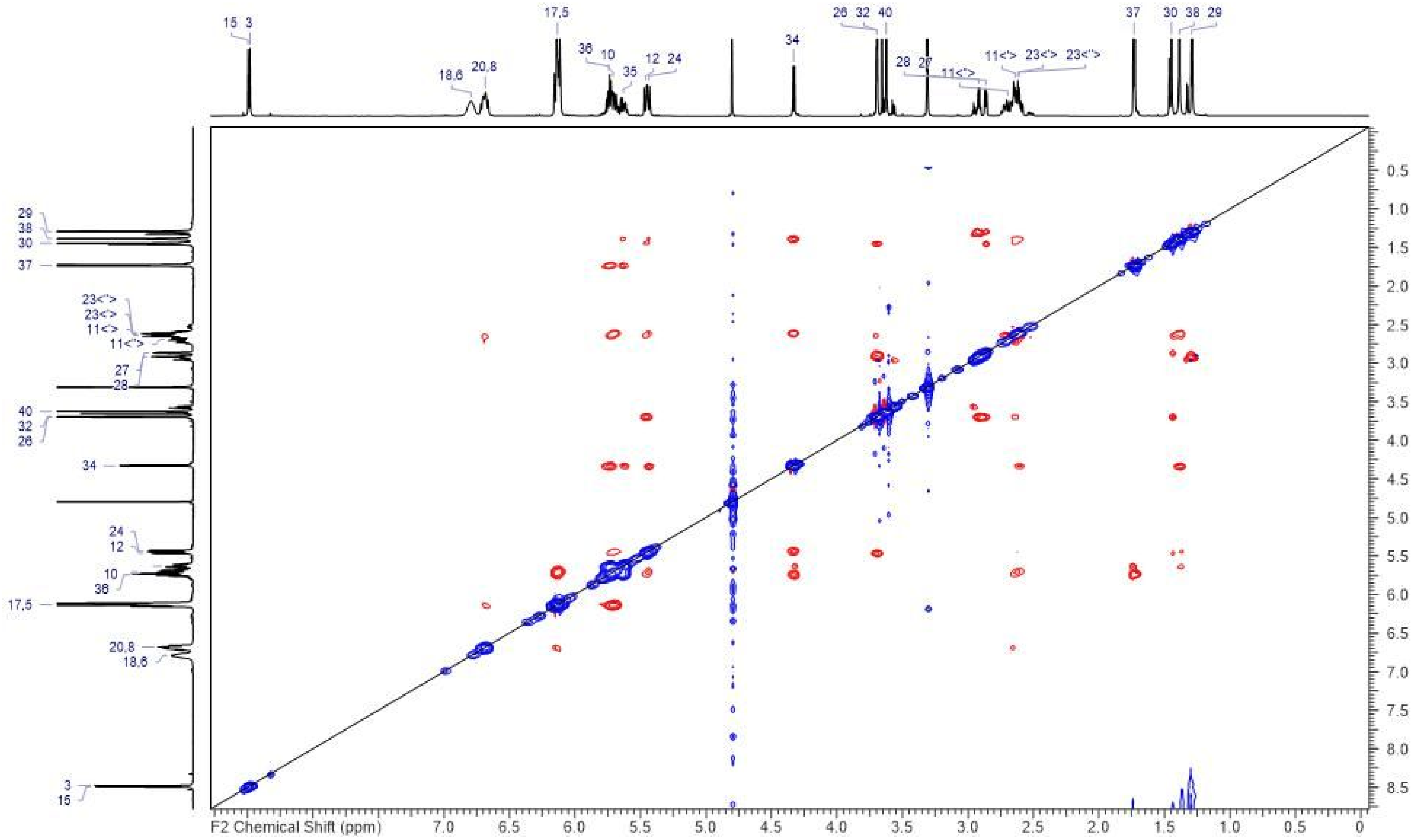


**Figure S39** $^{1}$H,$^{1}$H-ROESY NMR spectrum of 27,28-epoxy-disorazole Z (**8**) in methanol-$d_4$.

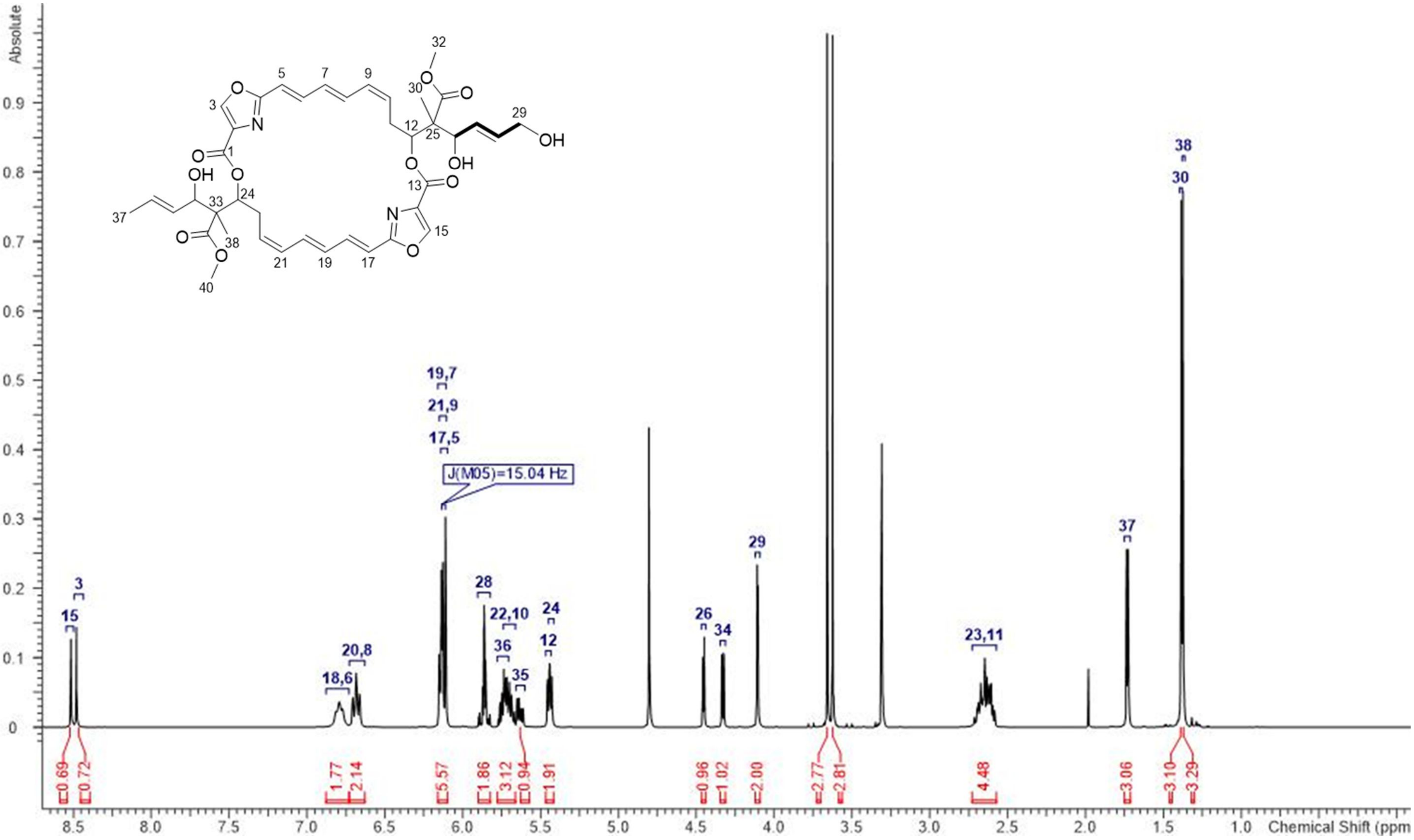


**Figure S40** $^{1}H$ NMR spectrum of 29-hydroxy-disorazole Z (**9**) in methanol-$d_4$ (600 MHz).

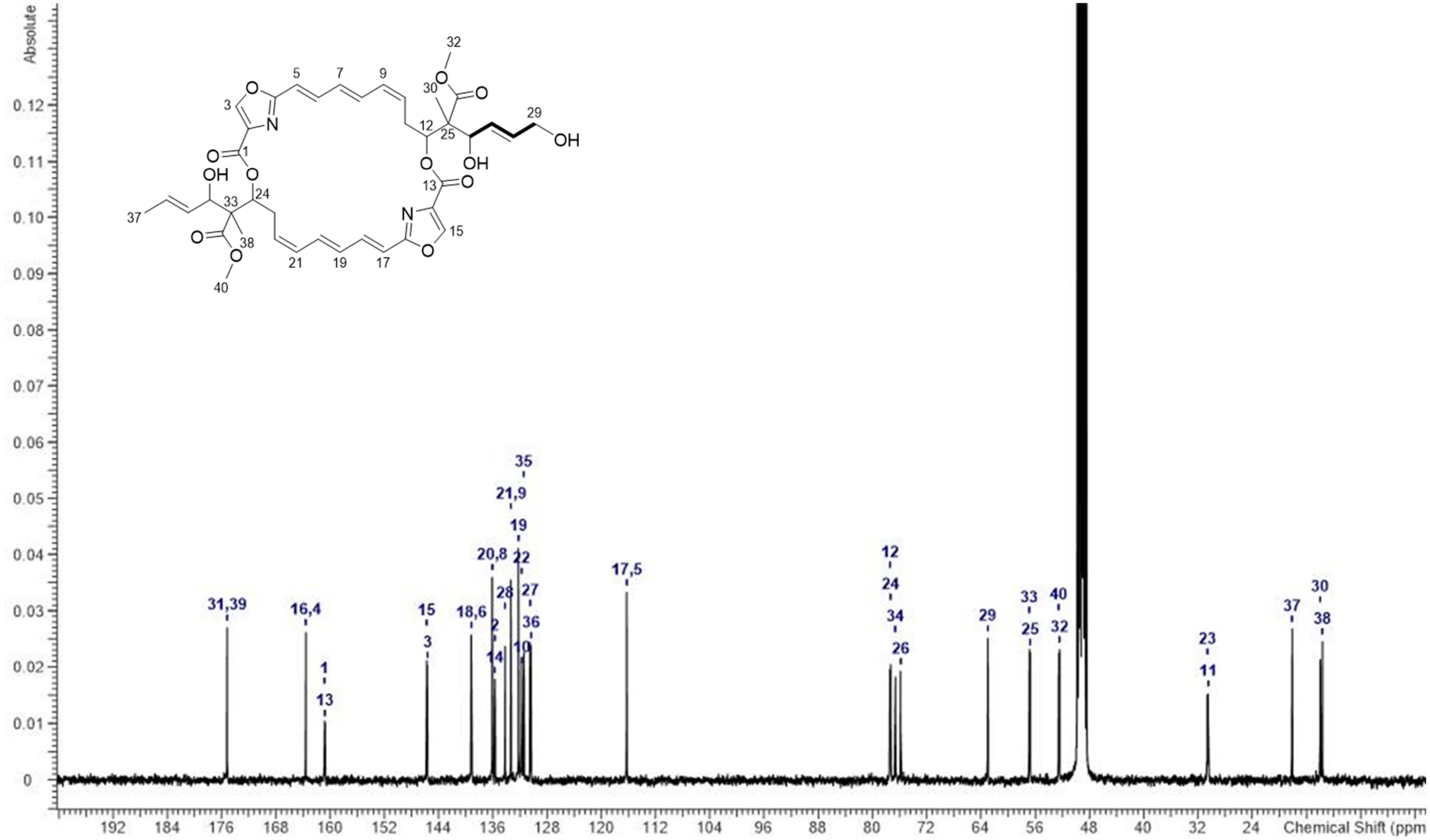

**Figure S41** $^{13}C$ NMR spectrum of 29-hydroxy-disorazole Z (**9**) in in methanol-$d_4$ (150 MHz).

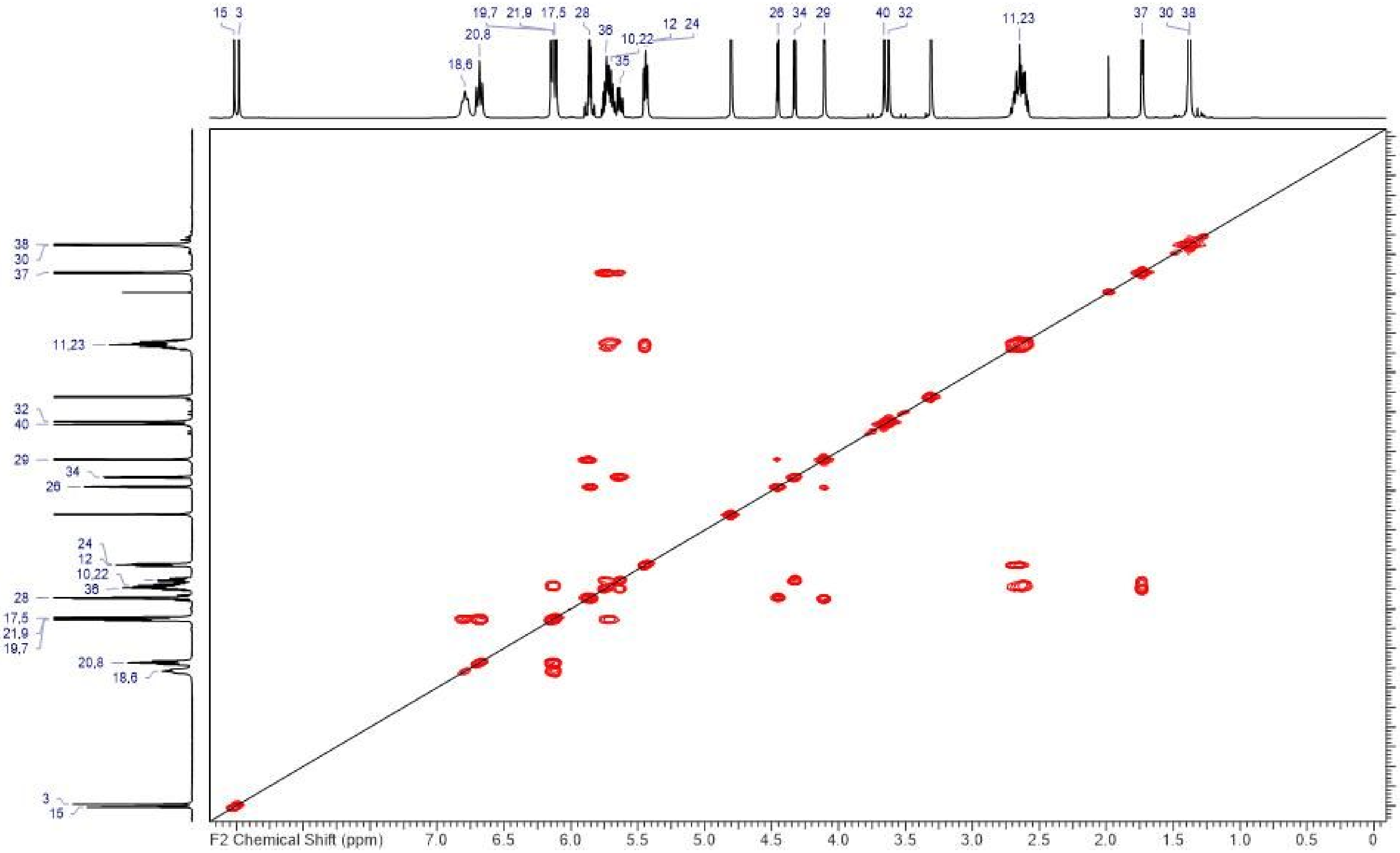


**Figure S42** $^{1}$H,$^{1}$H-COSY NMR spectrum of 29-hydroxy-disorazole Z (**9**) in in methanol-*d*$_4$.

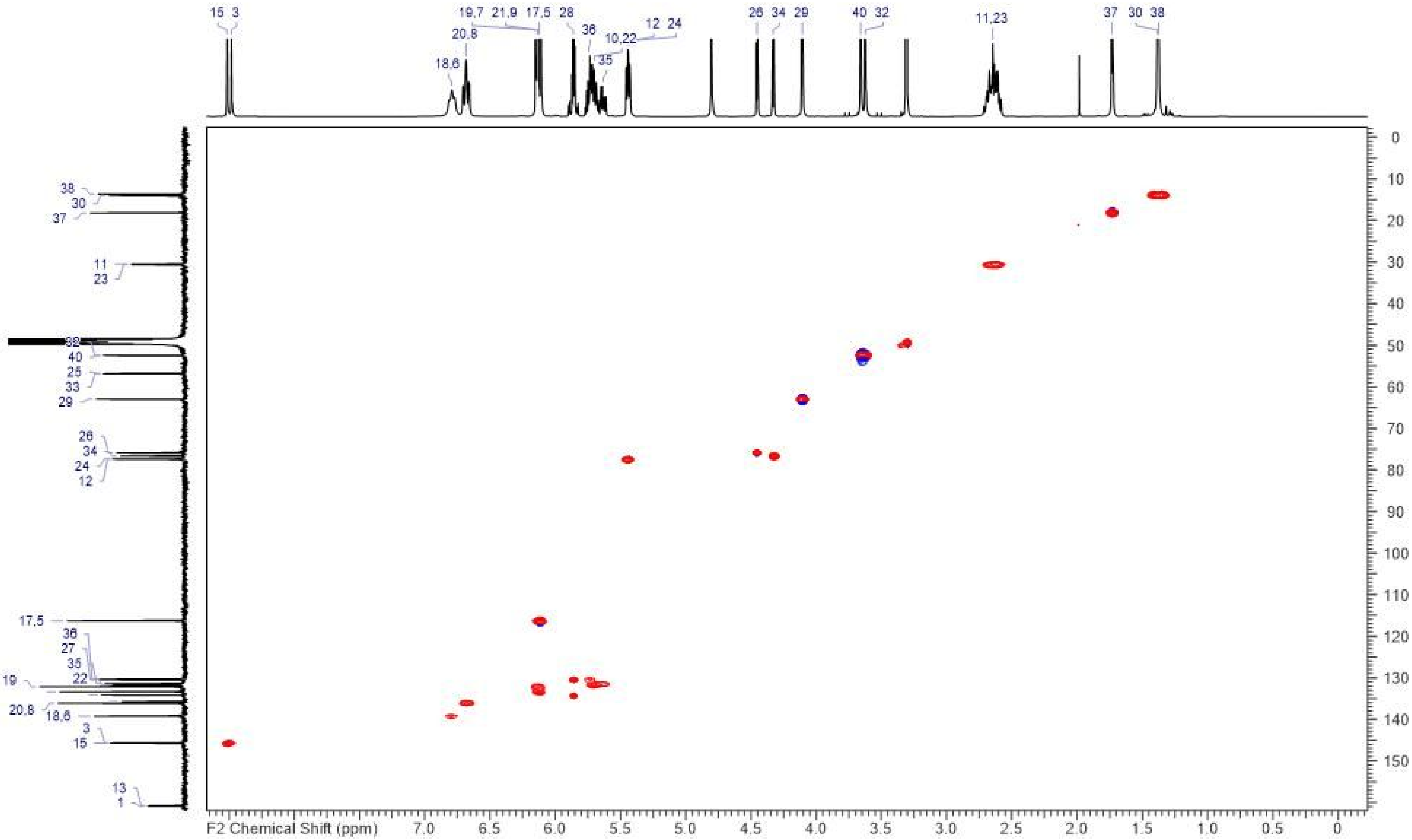


**Figure S43** HMQC-NMR spectrum of 29-hydroxy-disorazole Z (**9**) in in methanol-$d_4$.

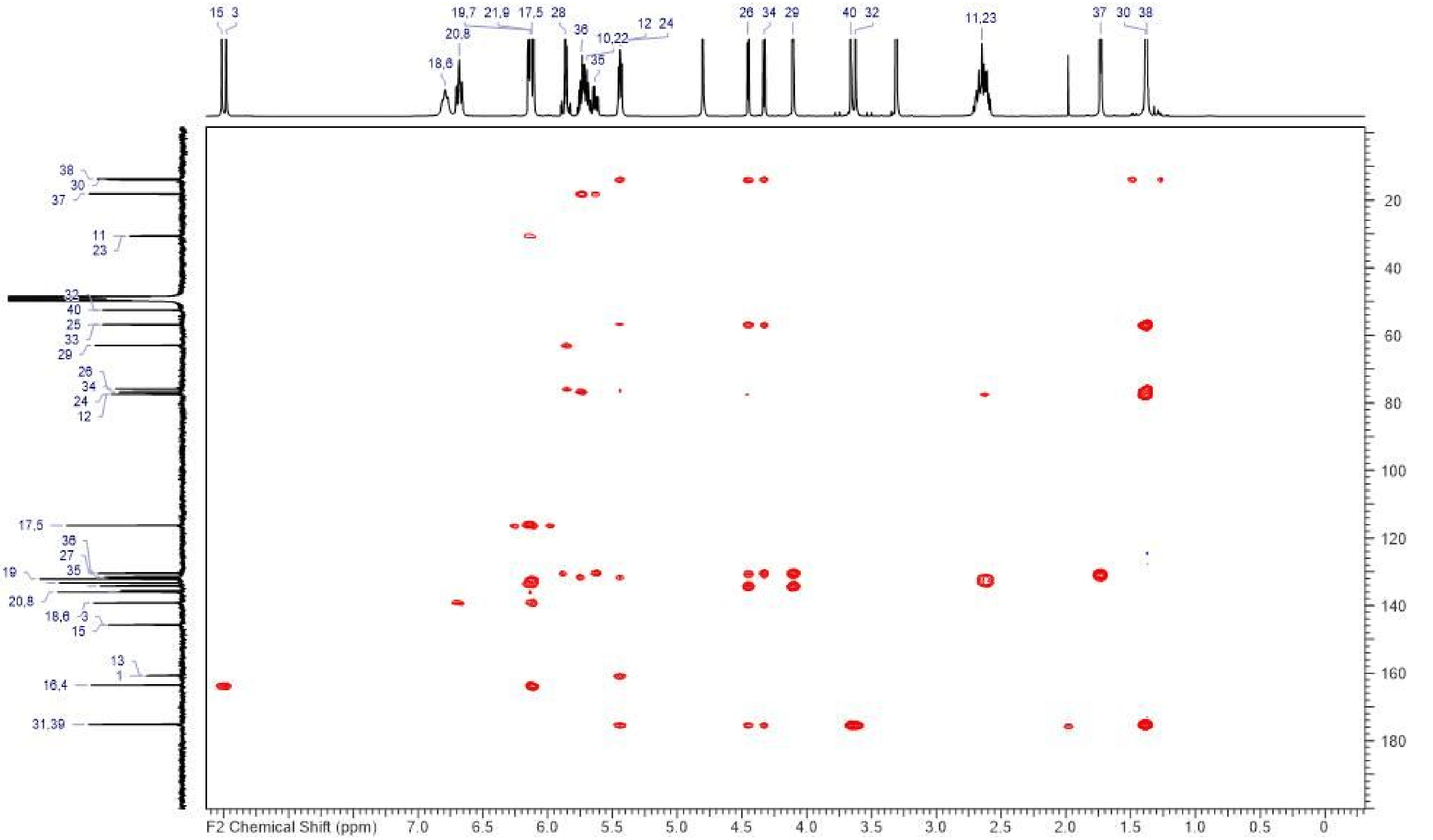


**Figure S44** HMBC NMR spectrum of 29-hydroxy-disorazole Z (**9**) in in methanol-$d_4$.

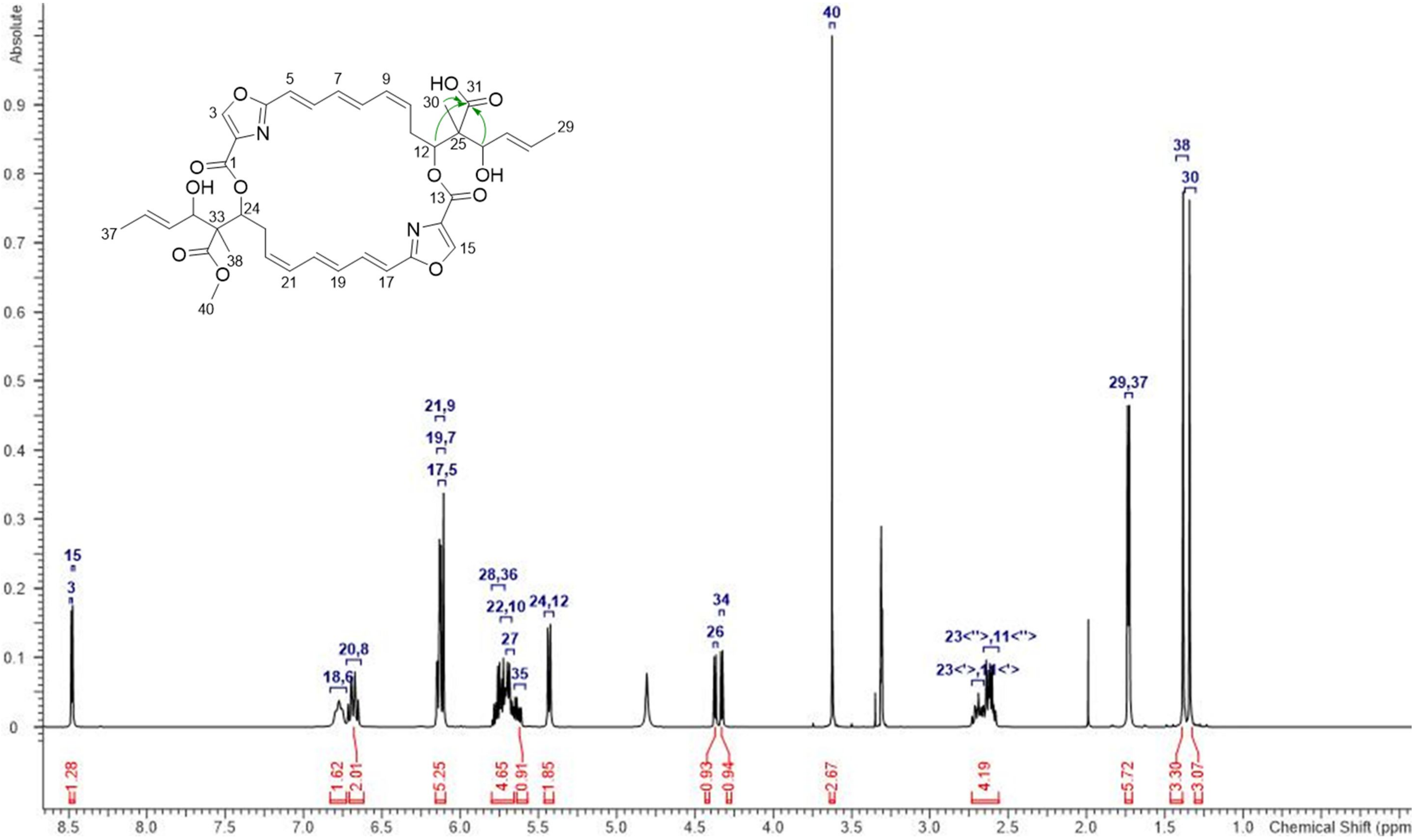


**Figure S45** $^{1}$H NMR spectrum of 31-*O*-desmethyl-disorazole Z (**10**) in methanol-$d_4$ (600 MHz).

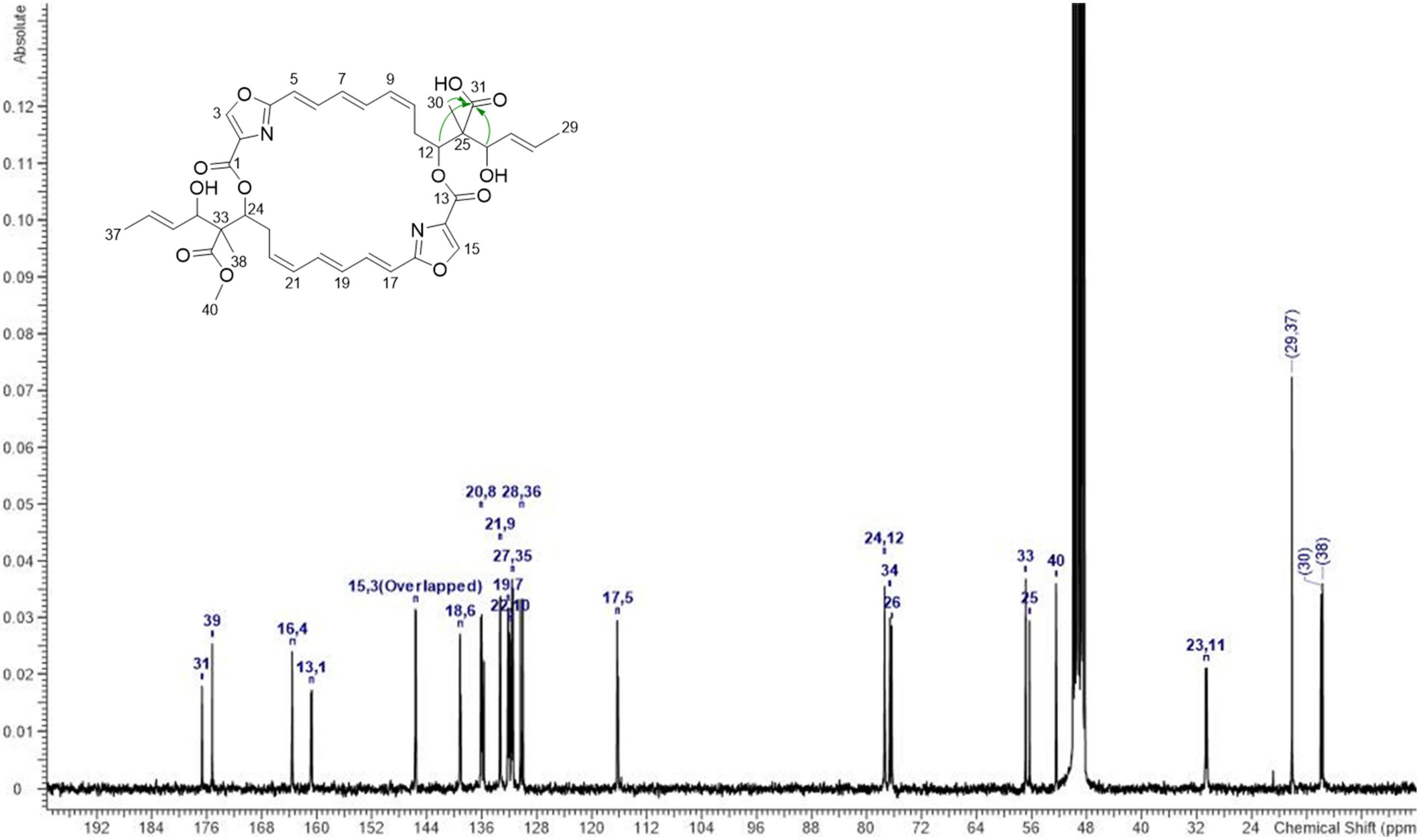


**Figure S46** $^{13}C$ NMR spectrum of 31-*O*-desmethyl-disorazole Z (**10**) in methanol-$d_4$ (150 MHz).

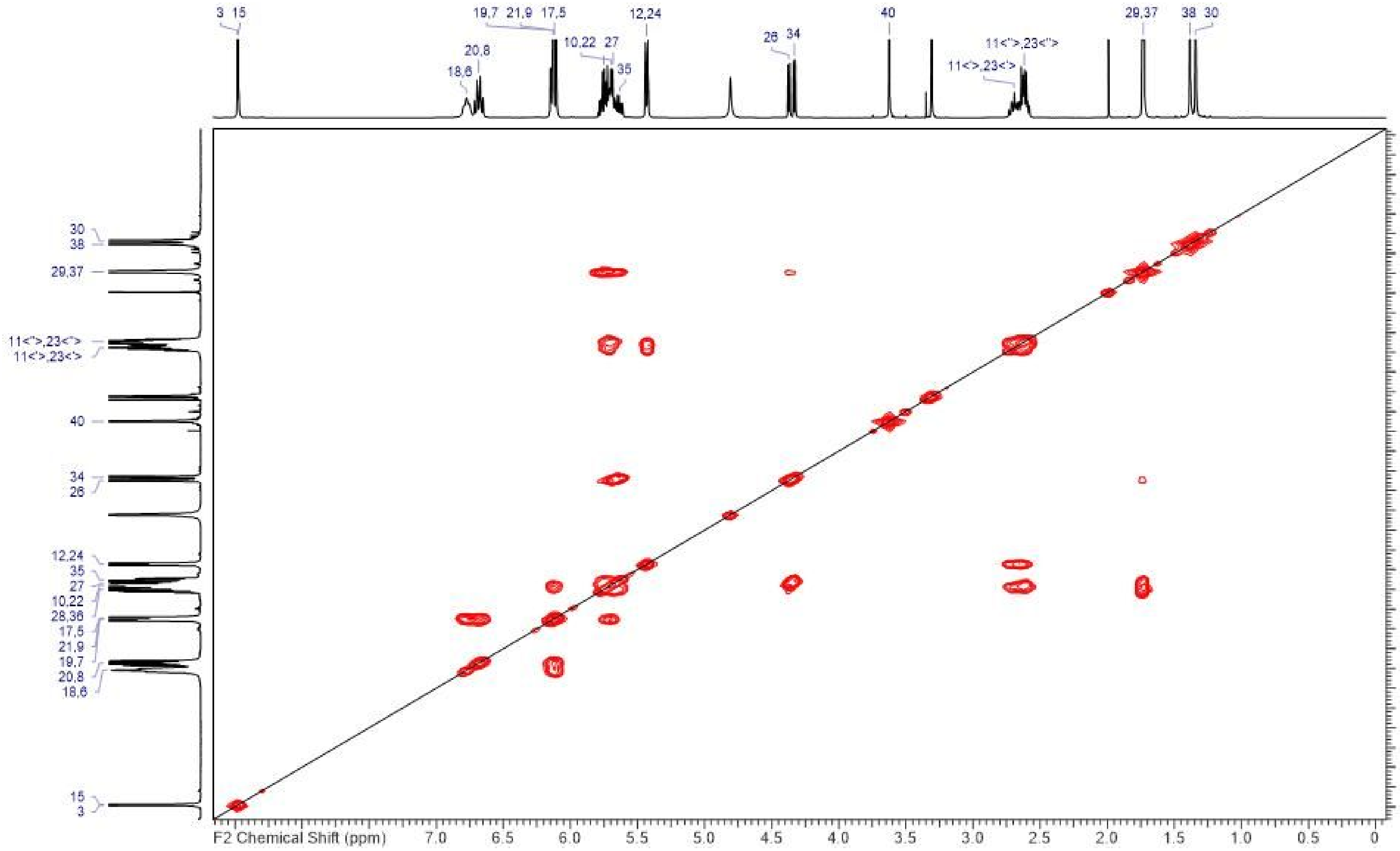


**Figure S47** $^{1}$H,$^{1}$H-COSY NMR spectrum of 31-*O*-desmethyl-disorazole Z (**10**) in in methanol-$d_4$.

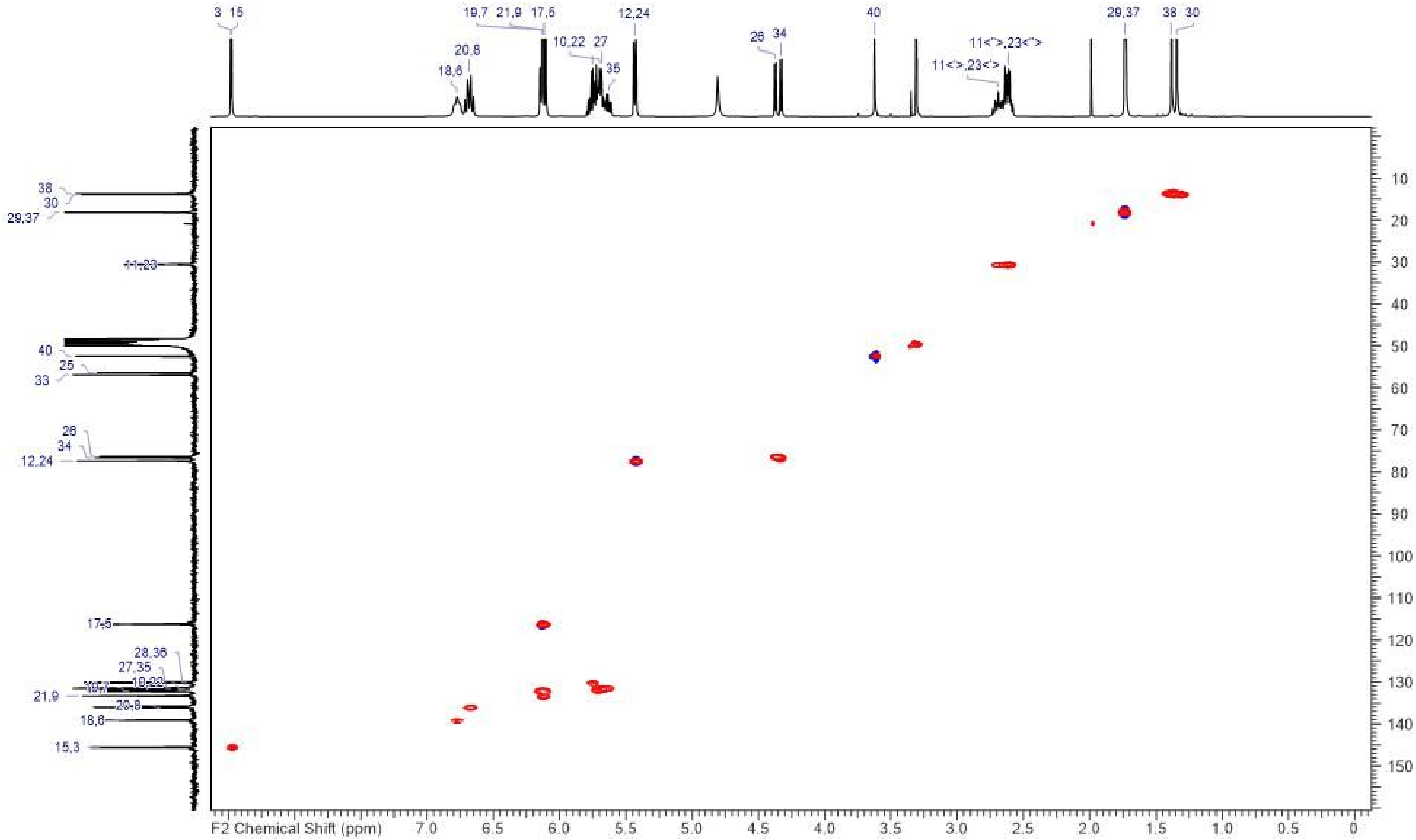


**Figure S48** HMQC-NMR spectrum of 31-*O*-desmethyl-disorazole Z (**10**) in in methanol-$d_4$.

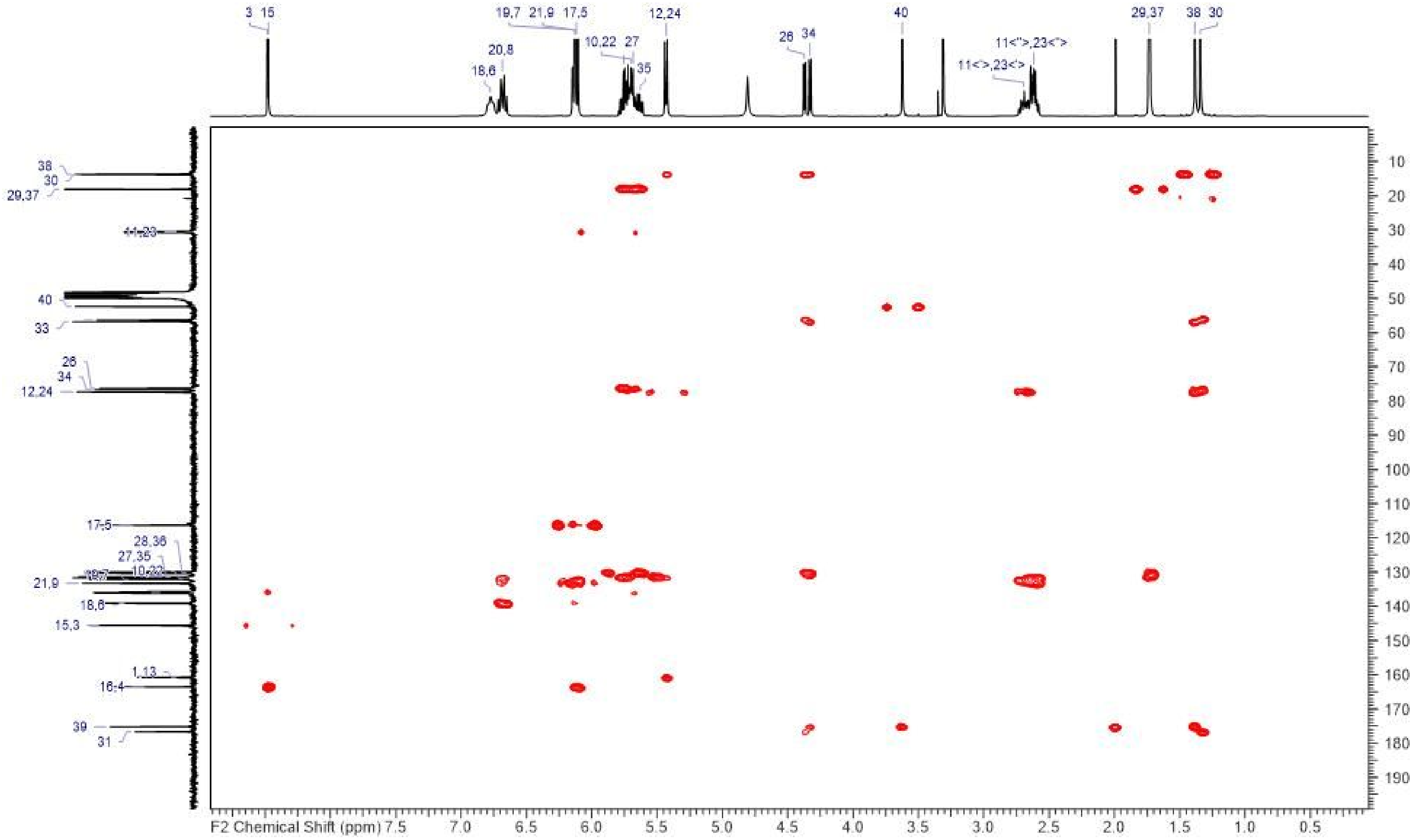


**Figure S49** HMBC NMR spectrum of 31-*O*-desmethyl-disorazole Z (**10**) in in methanol-*d*$_4$.

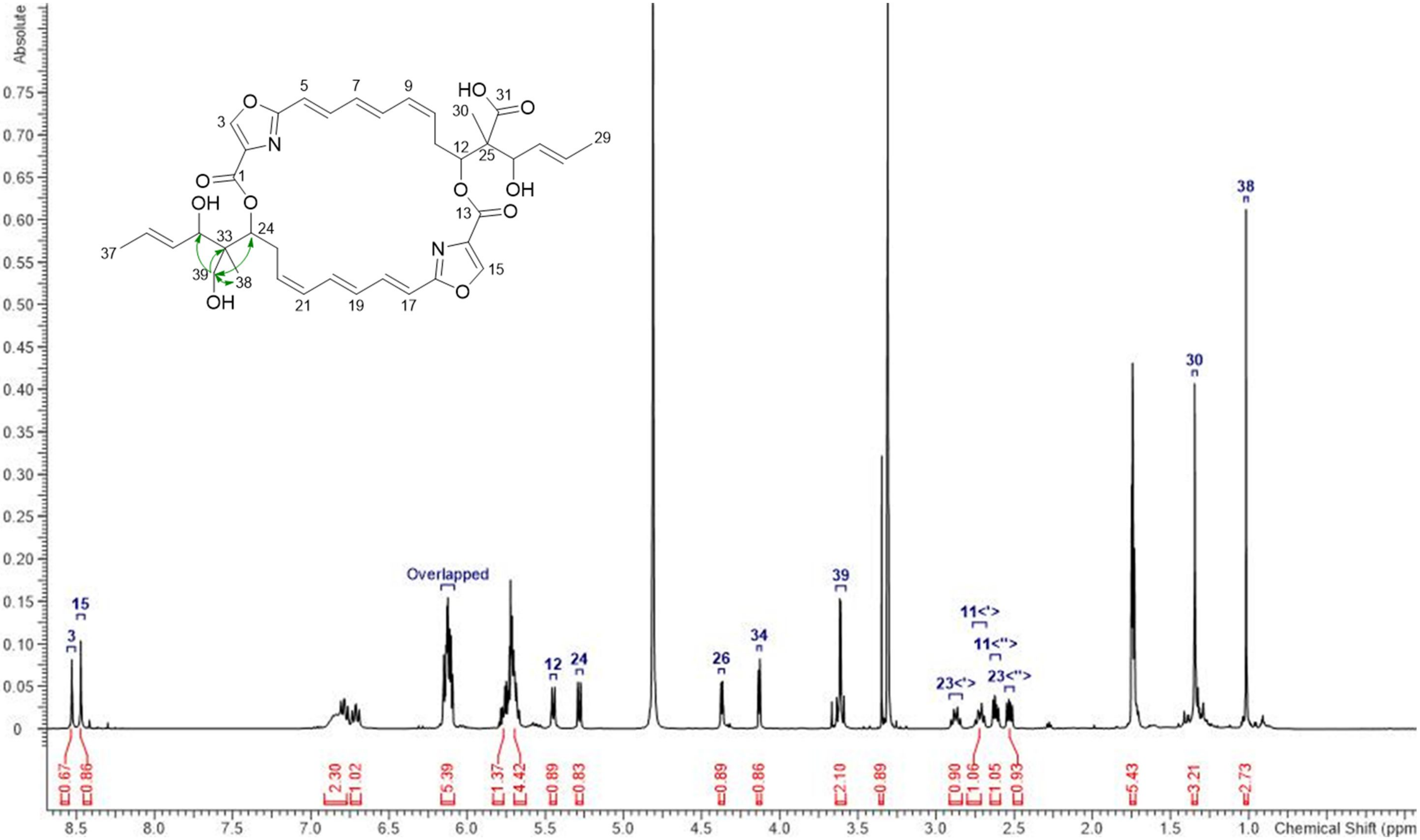


**Figure S50** $^{1}$H NMR spectrum of 31-*O*-desmethyl-39-hydroxy-disorazole Z (**11**) in methanol-$d_4$ (600 MHz).

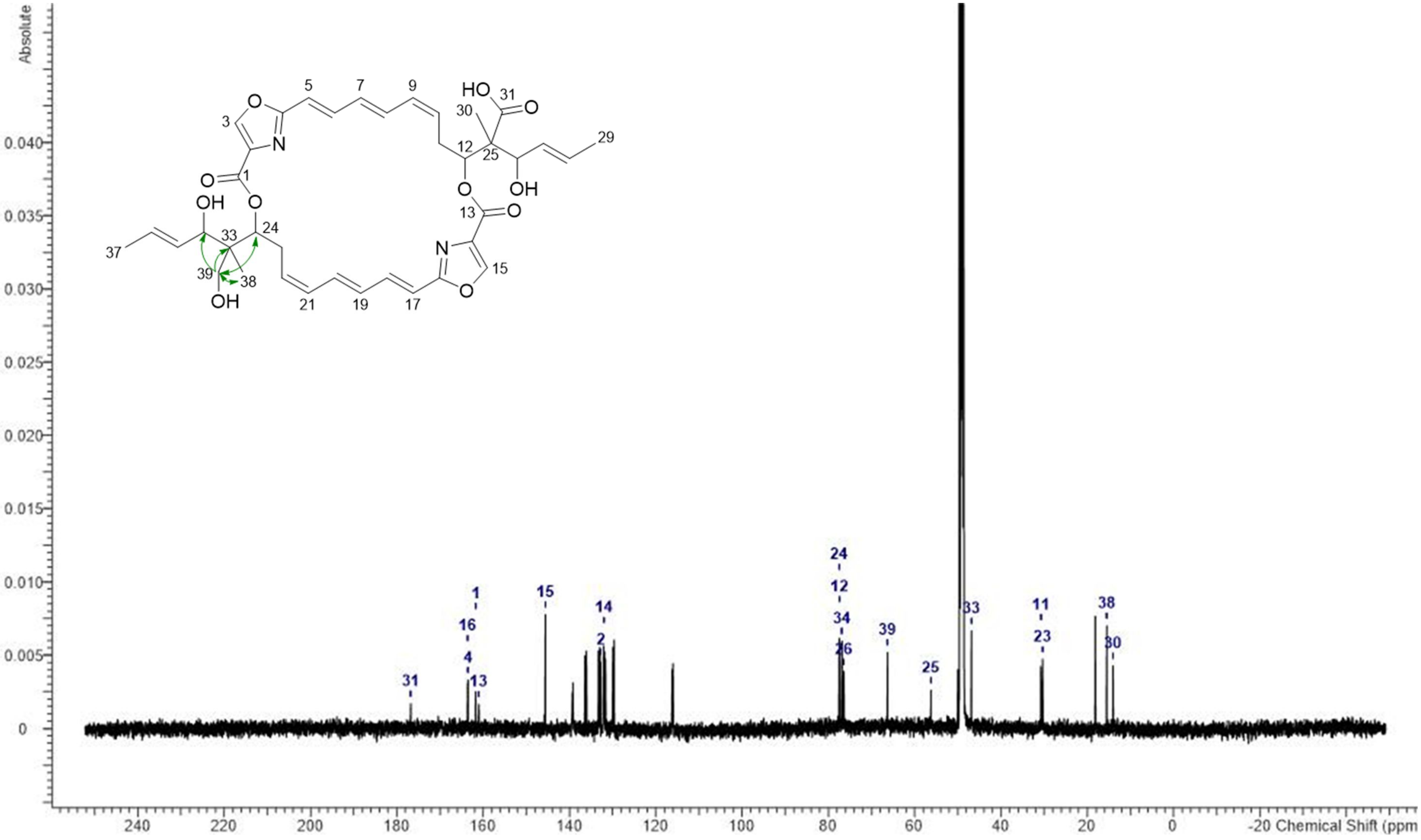

**Figure S51** $^{13}C$ NMR spectrum of 31-*O*-desmethyl-39-hydroxy-disorazole Z (**11**) in methanol-$d_4$ (150 MHz).

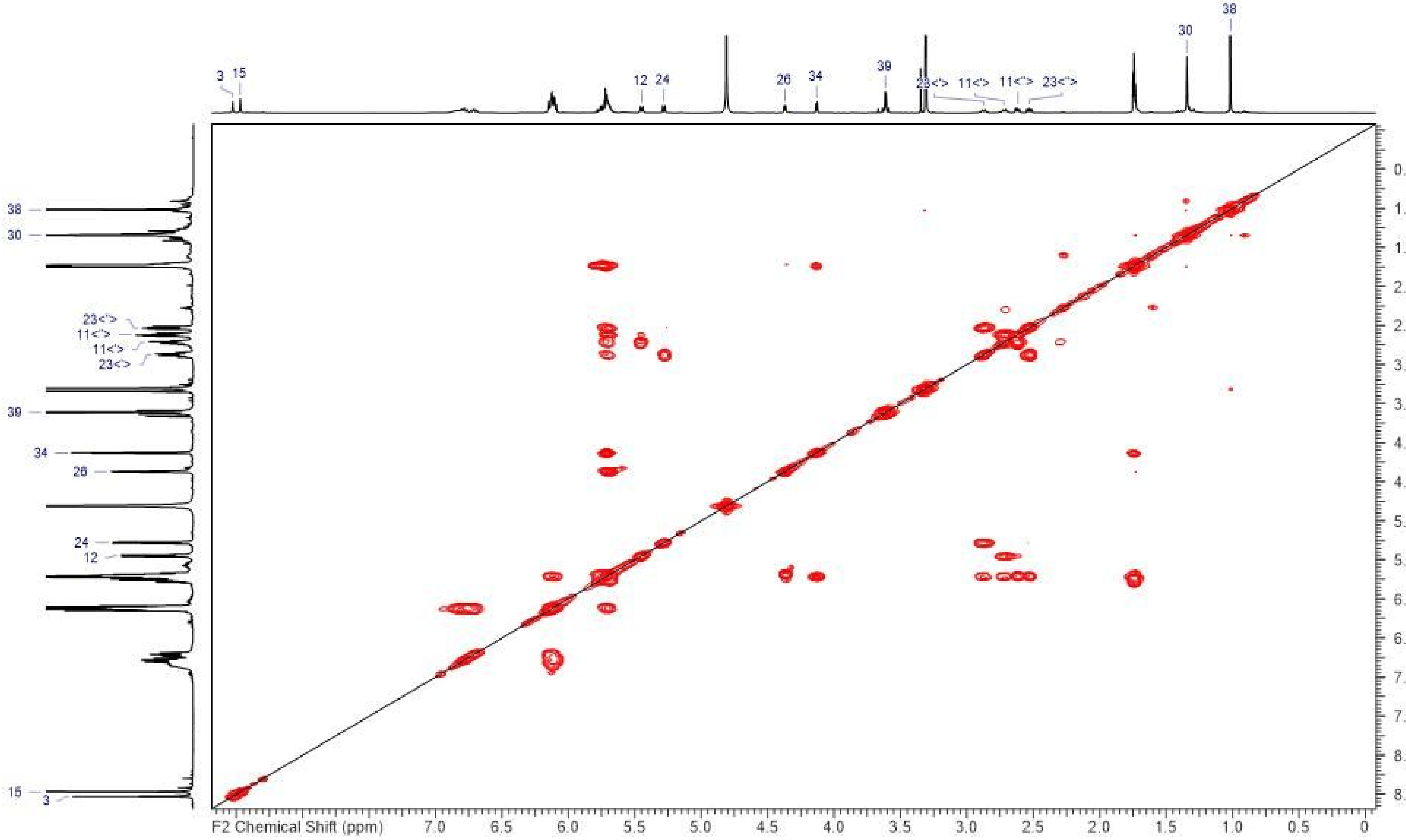


**Figure S52** $^{1}$H,$^{1}$H-COSY NMR spectrum of 31-*O*-desmethyl-39-hydroxy-disorazole Z (**11**) in in methanol-$d_4$.

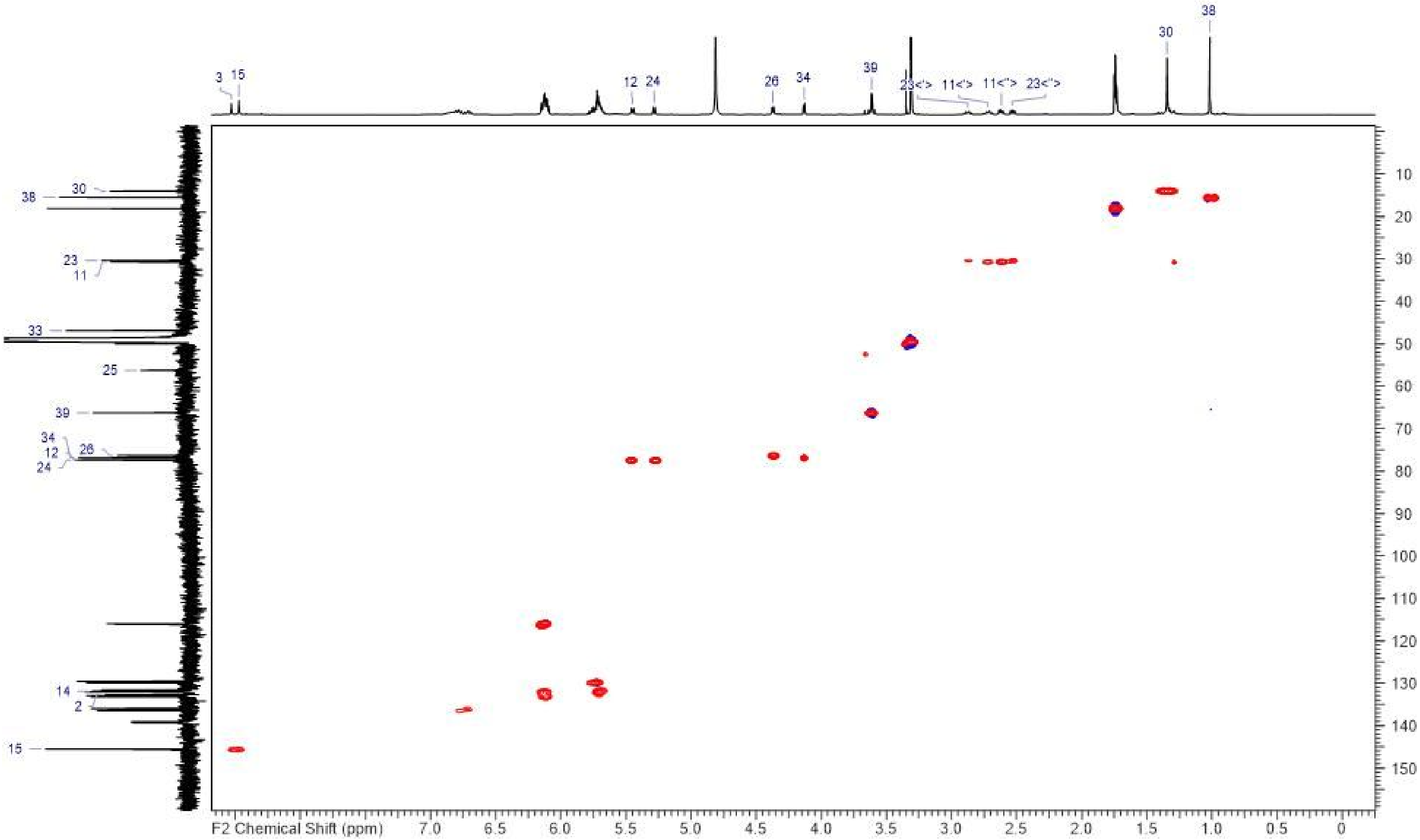


**Figure S53** HMQC-NMR spectrum of 31-*O*-desmethyl-39-hydroxy-disorazole Z (**11**) in in methanol-$d_4$.

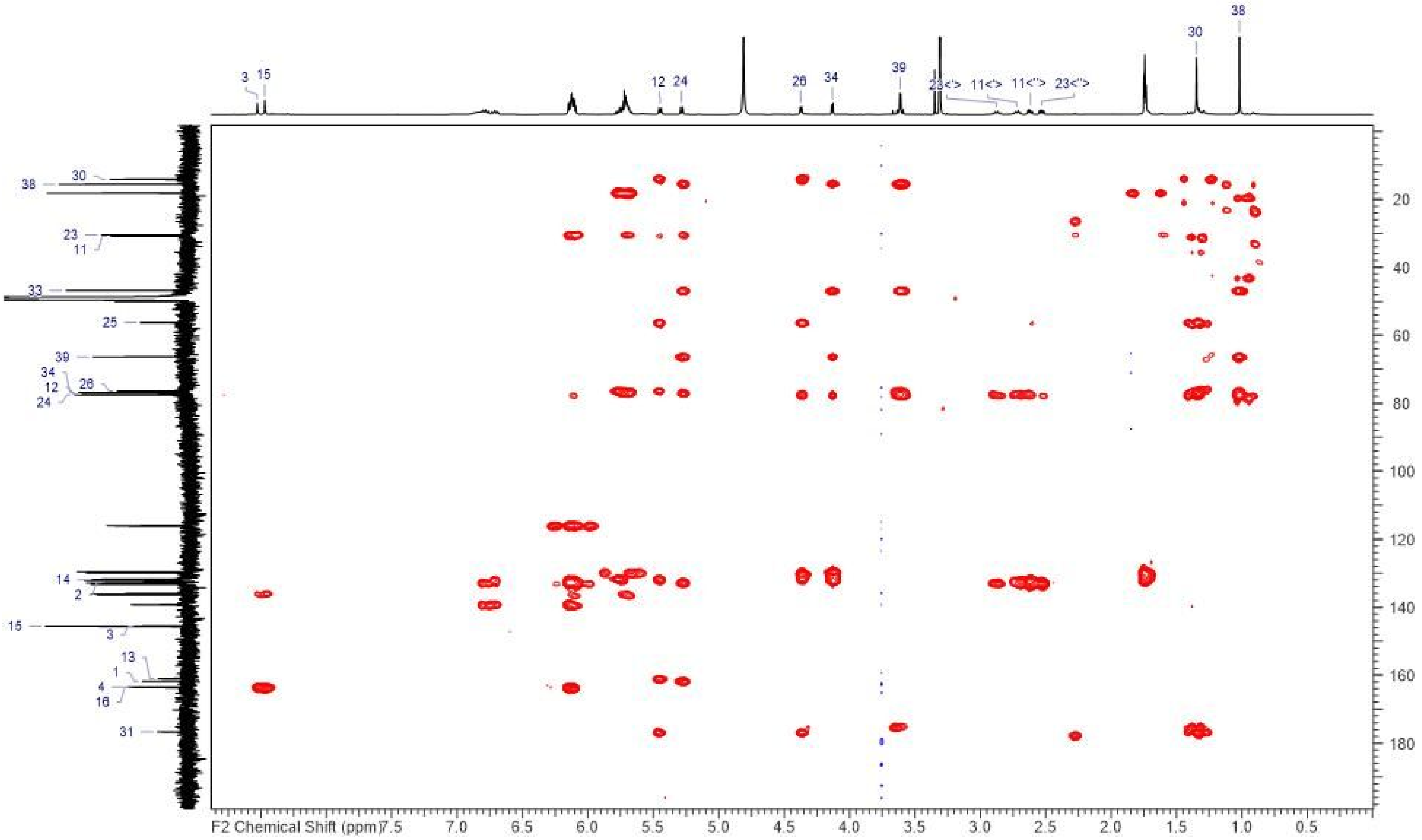


**Figure S54** HMBC NMR spectrum of 31-*O*-desmethyl-39-hydroxy-disorazole Z (**11**) in in methanol-$d_4$.

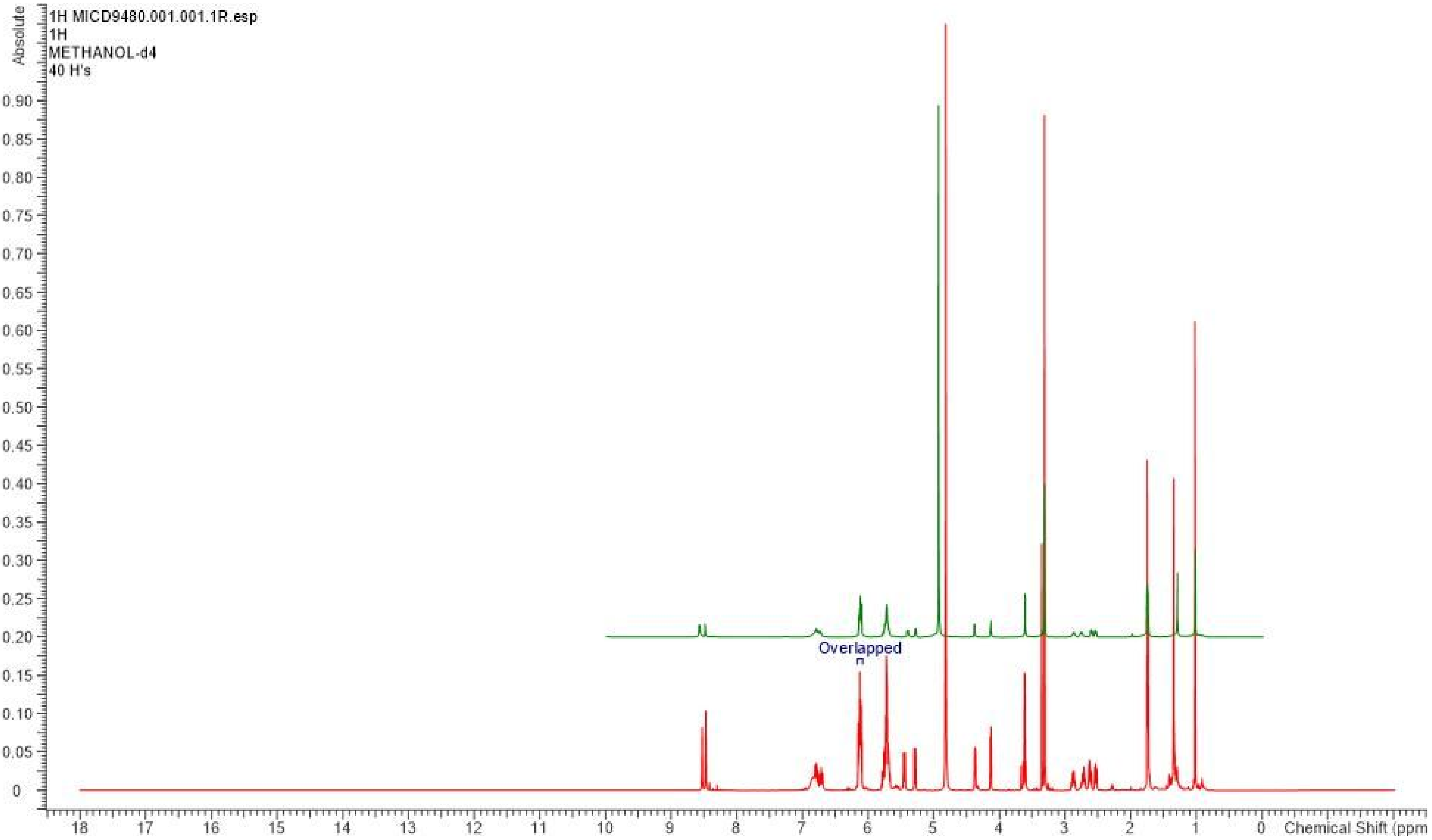


**Figure S55** Comparison of $^{1}$H NMR spectra of **11** methanol-$d_4$ isolated from *S. cellulosum* and *M. xanthus*. Bottom/red: isolated from *S. cellulosum* So ce1875; Top/green: isolated from *M. xanthus* DK1622::km-int-Ptet-dis427-gent-delF.

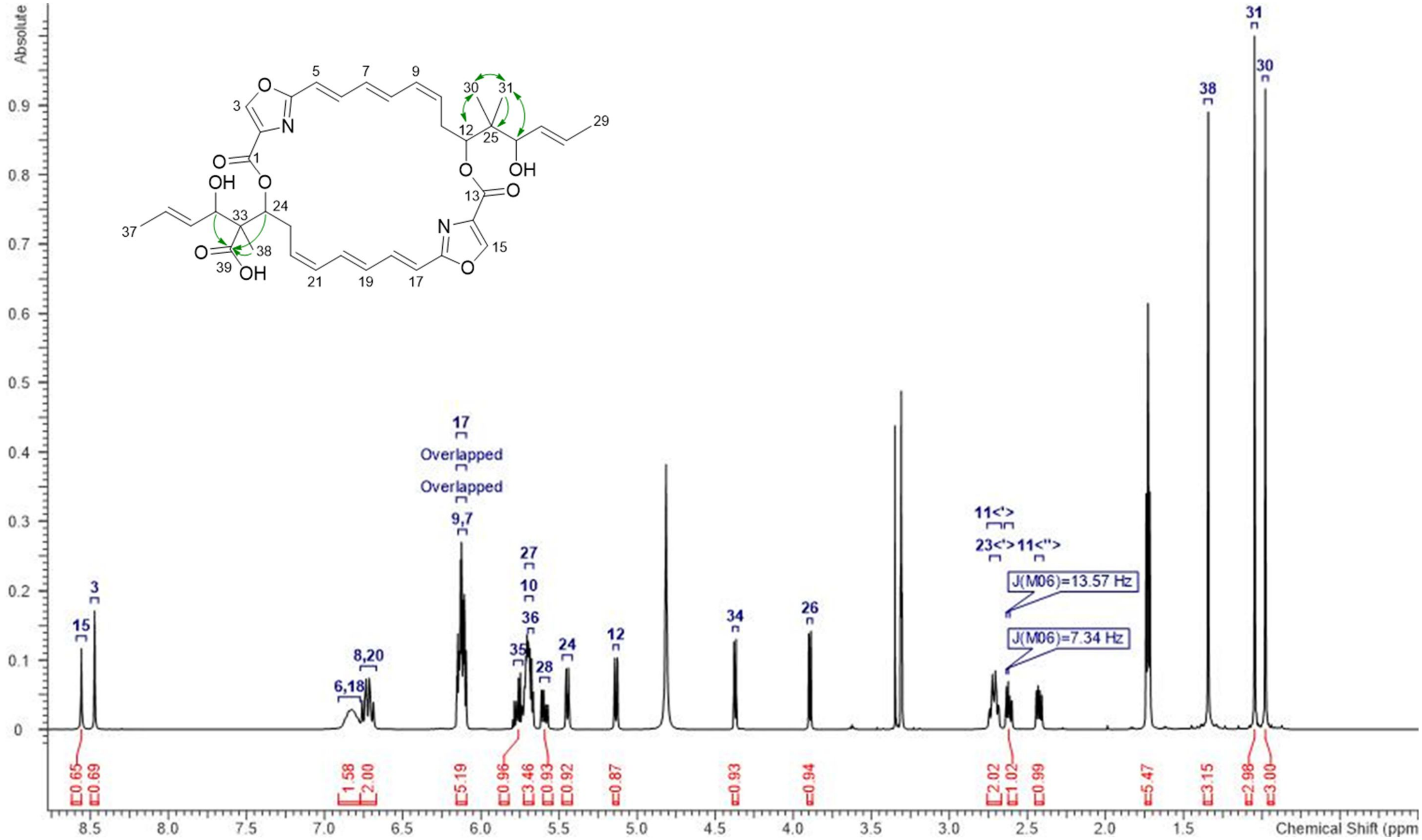


**Figure S56** $^{1}$H NMR spectrum of *O*-desmethyl-dimethyl-disorazole Z (**12**) in methanol-$d_4$ (600 MHz).

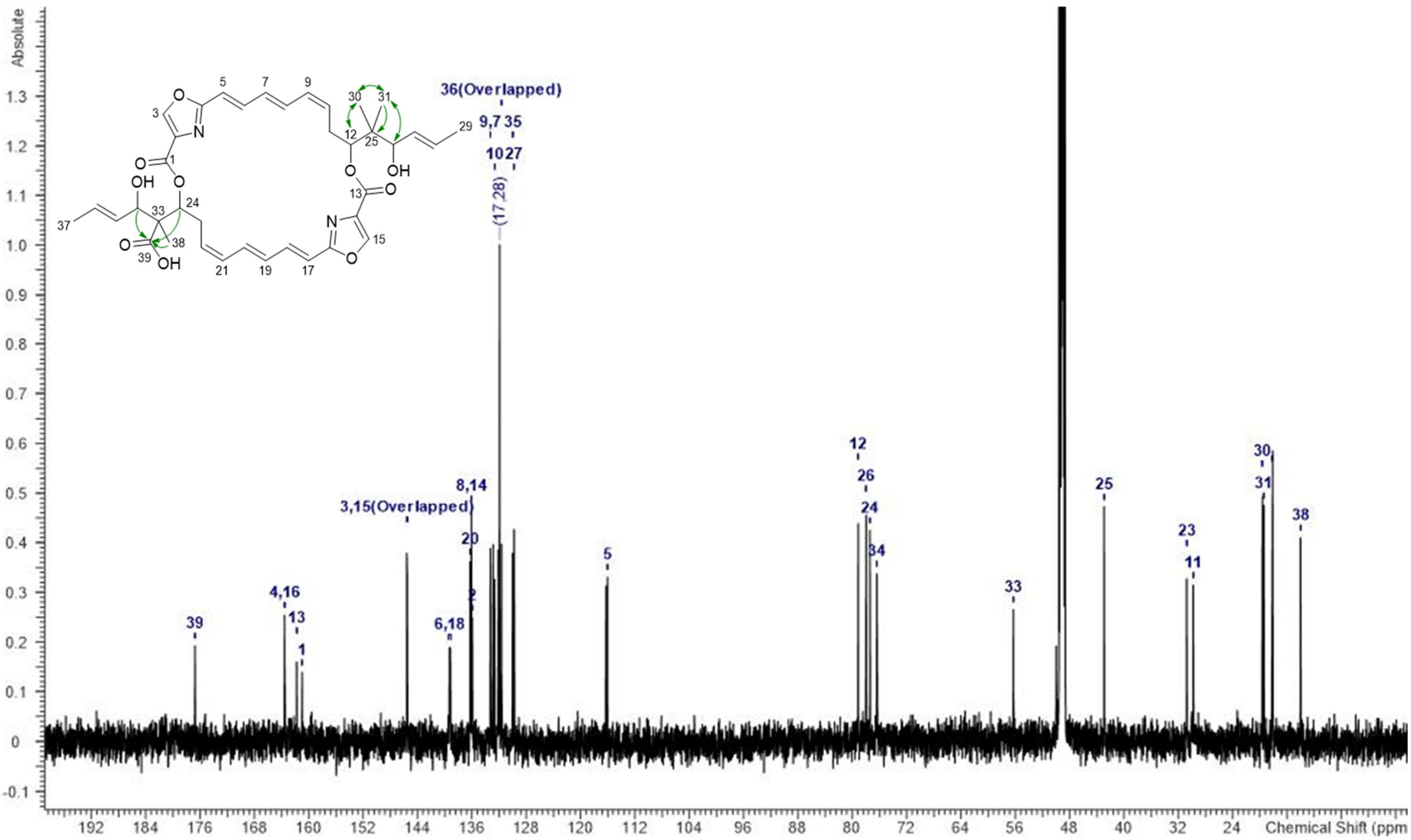


**Figure S57** $^{13}$C NMR spectrum of *O*-desmethyl-dimethyl-disorazole Z (**12**) in methanol-$d_4$ (150 MHz).

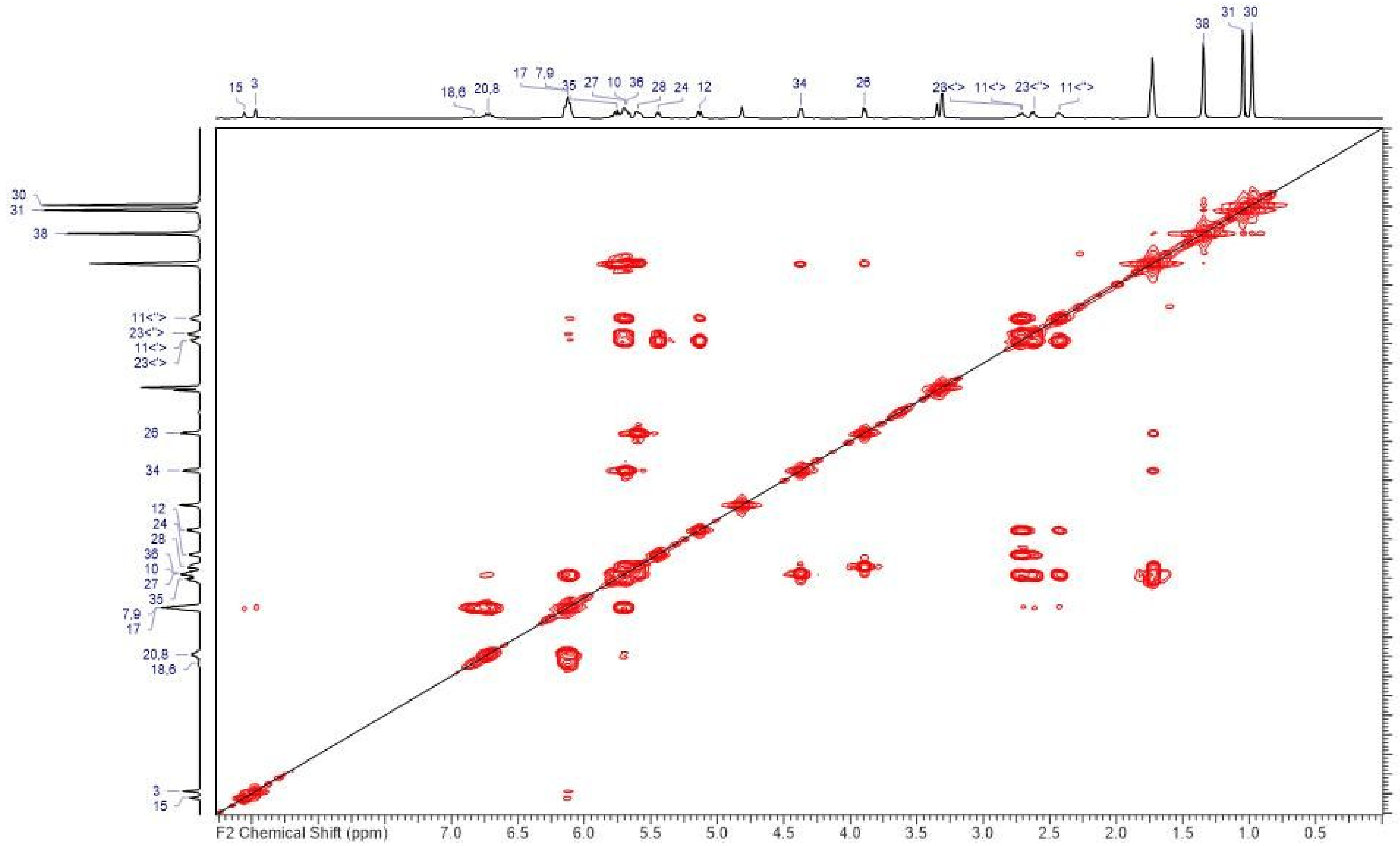


**Figure S58** $^{1}$H,$^{1}$H-COSY NMR spectrum of *O*-desmethyl-dimethyl-disorazole Z (**12**) in in methanol-$d_4$.

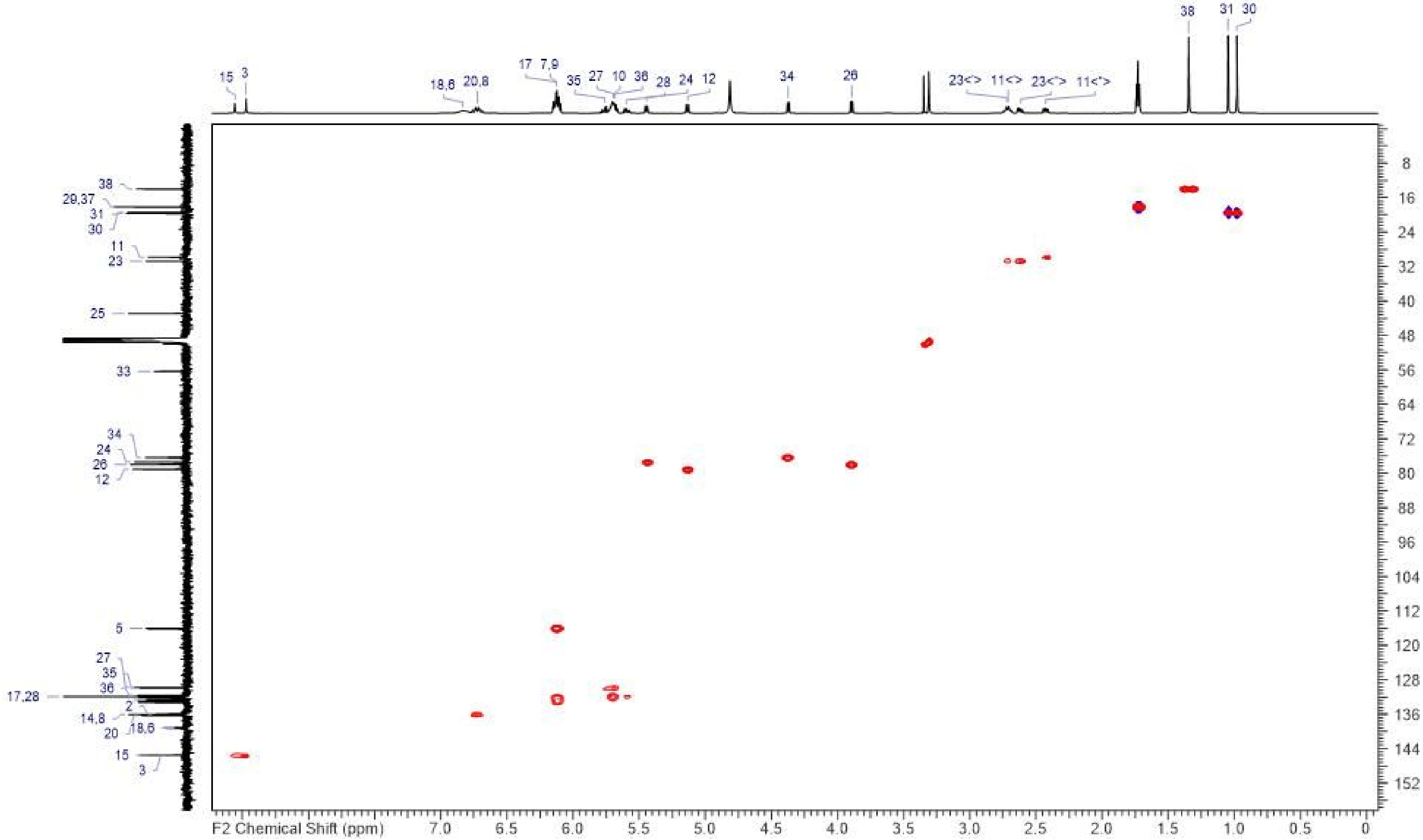


**Figure S59** HMQC-NMR spectrum of *O*-desmethyl-dimethyl-disorazole Z (**12**) in in methanol-$d_4$.

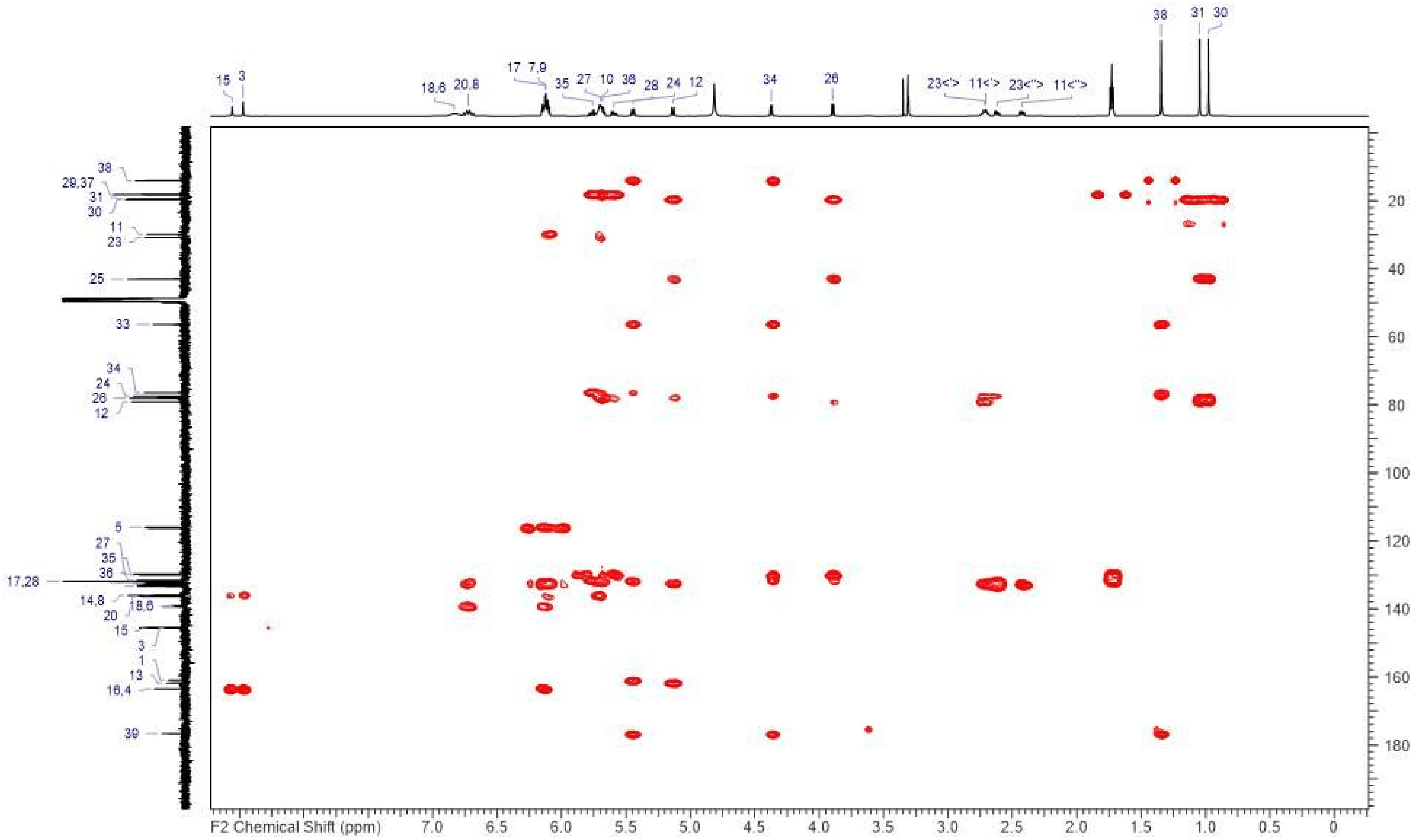


**Figure S60** HMBC NMR spectrum of *O*-desmethyl-dimethyl-disorazole Z (**12**) in in methanol-$d_4$.

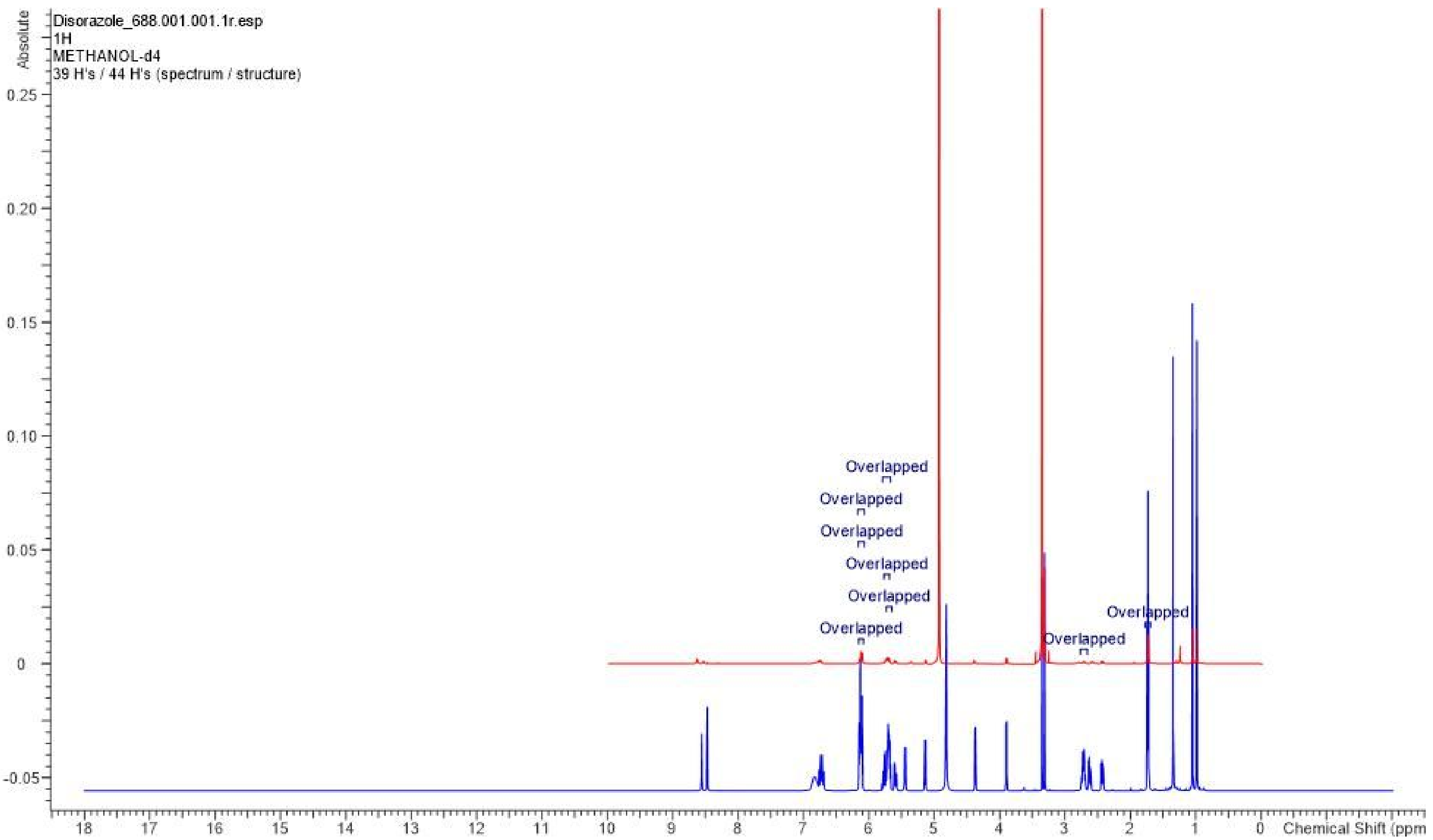


**Figure S61** Comparison of $^{1}$H NMR spectra of **12** in methanol-$d_4$ isolated from *S. cellulosum* and *M. xanthus***.** Bottom/blue: isolated from *S. cellulosum* So ce1875; Top/red: isolated from *M. xanthus* DK1622::km-int-Ptet-dis427-gent-delF.

## 4. References


1. Dale JA, Dull DL, Mosher HS. 1969. α-Methoxy-α-trifluoromethylphenylacetic acid, a versa-tile reagent for the determination of enantiomeric composition of alcohols and amines. J Org Chem 34:2543–2549. doi:10.1021/jo01261a013.

2. Lemière GL, Willaert JJ, Dommisse RA, Lepoivre JA, Alderweireldt FC. 1990. Determination of the absolute configuration and enantiomeric purity of alcohols from the $^{13}$C-NMR spectra of the corresponding MTPA esters. Chirality 2:175–184. doi:10.1002/chir.530020309.

3. Pehk T, Lippmaa E, Lopp M, Paju A, Borer BC, Taylor R. 1993. Determination of the absolute configuration of chiral secondary alcohols; new advances using $^{13}$C- and 2D-NMR spectroscopy. Tetrahedron: Asymmetry 4:1527–1532. doi:10.1016/S0957-4166(00)80354-7.

4. Hoye TR, Jeffrey CS, Shao F. 2007. Mosher ester analysis for the determination of absolute configuration of stereogenic (chiral) carbinol carbons. Nat Protoc 2:2451–2458. doi:10.1038/nprot.2007.354.

5. Jansen R, Irschik H, Reichenbach H, Wray V, Höfle G. 1994. Disorazoles, Highly Cytotoxic Metabolites from the Sorangicin-Producing Bacterium *Sorangium Cellulosum*, Strain So ce12. Liebigs Ann Chem 1994:759–773. doi:10.1002/jlac.199419940802.

6. Robbins T, Kapilivsky J, Cane DE, Khosla C. 2016. Roles of Conserved Active Site Residues in the Ketosynthase Domain of an Assembly Line Polyketide Synthase. Biochemistry 55:4476–4484. doi:10.1021/acs.biochem.6b00639.

7. Alekseyev VY, Liu CW, Cane DE, Puglisi JD, Khosla C. 2007. Solution structure and proposed domain domain recognition interface of an acyl carrier protein domain from a modular polyketide synthase. Protein Sci 16:2093–2107. doi:10.1110/ps.073011407.
